# Oxidative additions of alkynyl/vinyl iodides to gold and gold-catalyzed vinylation reactions triggered by the MeDalphos ligand†

**DOI:** 10.1039/d1sc01483h

**Published:** 2021-04-28

**Authors:** Jessica Rodriguez, Alexis Tabey, Sonia Mallet-Ladeira, Didier Bourissou

**Affiliations:** Laboratoire Hétérochimie Fondamentale et Appliquée (UMR 5069), Université de Toulouse (UPS), CNRS 118 route de Narbonne F-31062 Toulouse France dbouriss@chimie.ups-tlse.fr; Institut de Chimie de Toulouse (FR 2599) 118 Route de Narbonne 31062 Toulouse Cedex 09 France

## Abstract

The hemilabile Ad_2_P(*o*-C_6_H_4_)NMe_2_ ligand promotes fast, quantitative and irreversible oxidative addition of alkynyl and vinyl iodides to gold. The reaction is general. It works with a broad range of substrates of various electronic bias and steric demand, and proceeds with complete retention of stereochemistry from *Z* and *E* vinyl iodides. Both alkynyl and vinyl iodides react faster than aryl iodides. The elementary step is amenable to catalysis. Oxidative addition of vinyl iodides to gold and π-activation of alkenols (and *N*-alkenyl amines) at gold have been combined to achieve hetero-vinylation reactions. A number of functionalized heterocycles, *i.e.* tetrahydrofuranes, tetrahydropyranes, oxepanes and pyrrolidines were obtained thereby (24 examples, 87% average yield). Taking advantage of the chemoselectivity for vinyl iodides over aryl iodides, sequential transformations involving first a hetero-vinylation step and then a C–N coupling, a C–C coupling or an heteroarylation were achieved from a vinyl/aryl bis-iodide substrate.

## Abstract 2

The hemilabile Ad_2_P(*o*-C_6_H_4_)NMe_2_ ligand promotes fast, quantitative and irreversible oxidative addition of alkynyl and vinyl iodides to gold.

## Graphical abstract

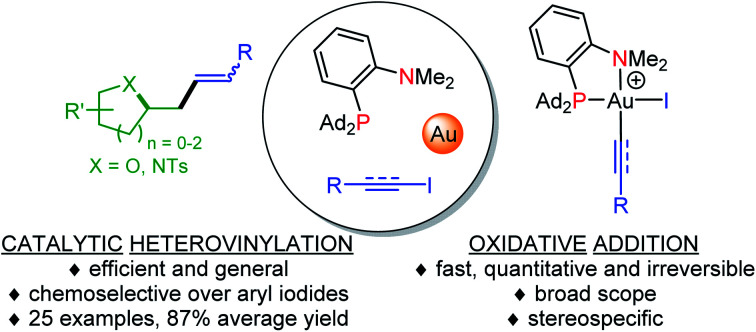

## Introduction

**Fig. 1:**
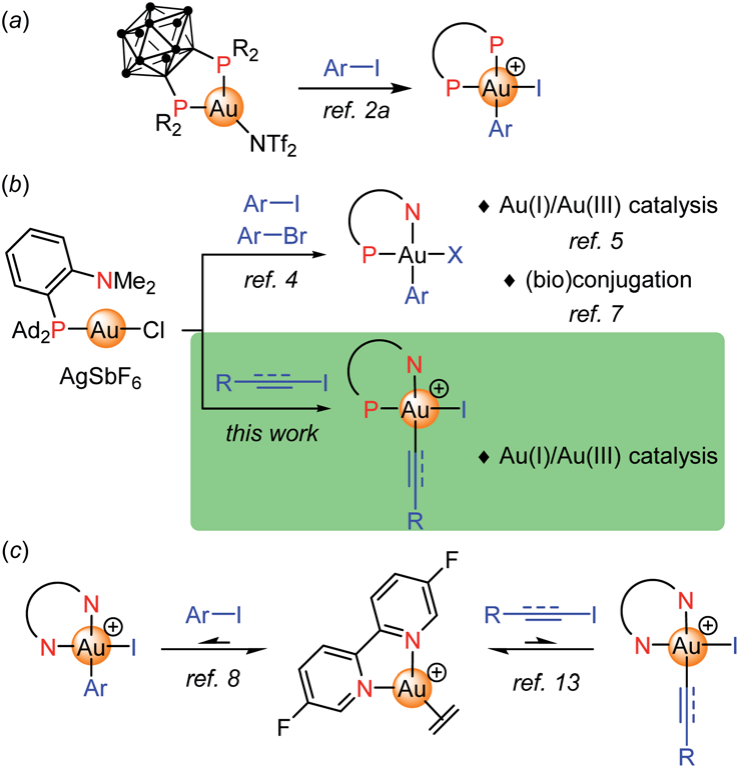
Ligand-enabled oxidative additions of C–I/C–Br bonds to gold.

Judicious ligand design has been recently shown to overcome the reluctance of gold(i) complexes to undergo oxidative addition, providing a valuable mean to achieve Au(i)/Au(iii) cycles, including 2-electron redox catalysis.^1^ Our group first reported a bending strategy. Thanks to their unique chelating properties, *o*-carboranyl diphosphines promote oxidative addition of aryl iodides and strained C–C bonds to gold under mild conditions (Fig. 1a).^2^ Then, (P,N) ligands, in particular MeDalphos^3^ proved to also prepare and activate gold(i) towards oxidative addition due to the hemilabile and hard character of the N center. The (P,N) gold complex reacts readily with a variety of aryl iodides and bromides (Fig. 1b).^4^ The resulting aryl gold(iii) complexes are reactive and open perspectives both in catalysis and (bio)conjugation. The ligand-enabled oxidative addition of aryl iodides has been used as entry point to achieve catalytic C–C and C–N cross-couplings such as the arylation of indoles, of anilines and (sulfon)amides.^5*a*–*c*^ It has also been successfully merged with π activation, enabling the difunctionalization of alkenes (heteroarylation, diarylation).^5*d*–*g*,6^ In addition, the (P,N) aryl Au(iii) proved very powerful and chemoselective to arylate cysteine residues of peptides and proteins, as well as to derivatize B-cages and access well-defined hybrid nanoclusters.^7^

Apart from P-based ligands, McGrady, Bower and Russell employed a simple bipyridine to trigger oxidative addition to gold.^8,9^ The corresponding ethylene gold(i) complex reacts with aryl iodides (20 eq.) at 50 °C, quantitative conversions are achieved upon removal of ethylene (Fig. 1c). The oxidative addition is in fact reversible in this case. The feasibility of a Negishi-type biaryl coupling was substantiated by performing the respective elementary steps sequentially in stoichiometric conditions.

Besides aryl substrates, there are also a few precedents of activation and coupling of electrophilic alkynyl and vinyl compounds at gold. Most developed is the use of alkynyl hypervalent iodine reagents, as pioneered by Waser in the late 2000's^10^ and further extended by Liu, Patil and Hashmi.^11^ Ethynylbenzoiodoxolone reagents (EBX) proved to be very efficient alkynylating reagents and they were engaged in a number of C(sp^2^)–C(sp) coupling reactions. Much less is known from alkynyl halides, with only two gold-catalyzed cyclization–alkynylation sequences reported in 2019 by Hashmi with *tert*-butylallenoates and alkynyl bromides (under thermal conditions),^12*a*^ and by Ollivier and Fensterbank with *o*-alkynylphenols and alkynyl iodides (under Ir-photosensitized conditions).^12*b*^ In addition, Bower and Russell have reported very recently first examples of oxidative addition of alkynyl and vinyl iodides to gold from the (bipyridine)Au(H_2_C=CH_2_)^+^ complex (Fig. 1c).^13^ As for aryl iodides, the reaction is reversible and requires an excess of substrate to isolate the respective Au(iii) complexes.

Given the high reactivity and versatility that the (P,N) ligand confers to gold in the oxidative addition of aryl halides, we were intrigued about the possibility for the (P,N) gold complex to also activate alkynyl and vinyl iodides. The results we obtained along this line are reported herein. The reaction is shown to proceed readily and quantitatively with a variety of substrates, used in stoichiometric amounts. Chemoselectivity over aryl iodides has been investigated. The elementary step has been leveraged into catalysis. Hetero-vinylation reactions were found to be efficiently catalyzed by the (P,N) gold complex. The scope of the transformation has been explored and it has been applied to the synthesis of various polyfunctional products featuring tetrahydrofurane moieties starting from a vinyl/aryl bis-iodide substrate.

## Results and discussion

**Scheme 1:**
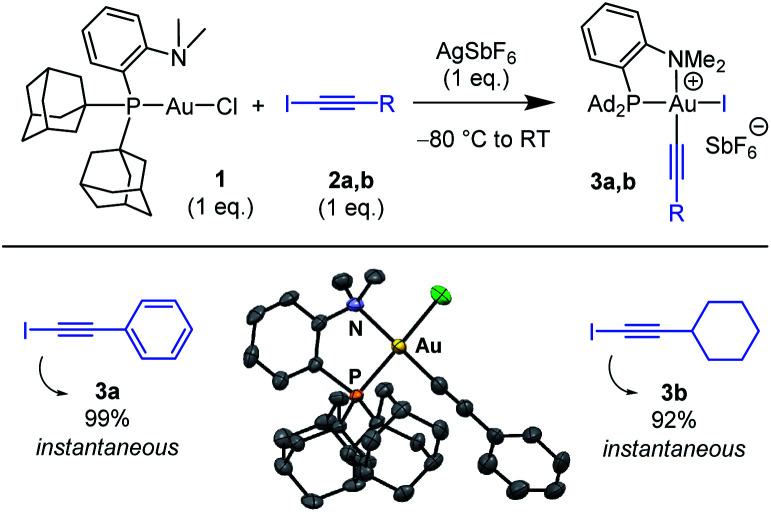
Oxidative addition of alkynyl iodides to the (P,N)AuCl complex **1**. Reaction times and spectroscopic yields based on ^31^P NMR. Molecular structure of the (P,N)Au(iii) alkynyl complex **3a** (hydrogen atoms and counter anion omitted for clarity, ellipsoids shown at 50% probability).

The ability of the MeDalphos ligand to promote oxidative addition of alkynyl iodides to gold was first investigated (Scheme 1).^14^ The (P,N)AuCl complex **1** was reacted with iodo phenylacetylene **2a** in the presence of silver hexafluoroantimonate. NMR monitoring showed instantaneous and quantitative formation of the Au(iii) alkynyl complex **3a**. The ^31^P NMR signal appears at *δ* 103 ppm, deshielded by 46 ppm with respect to (P,N)AuCl. The ^1^H NMR signal for the NMe_2_ group resonates at *δ* 3.68 ppm, shifted downfield by *ca.* 1 ppm compared to that of the gold(i) precursor **1**. This is reminiscent of that observed for the (P,N)Au(iii) aryl complexes deriving from oxidative addition of aryl halides^4,5*a*^ and suggests coordination of the nitrogen atom to gold. Most diagnostic for the oxidative addition of PhCC–I is the disappareance of the ≡C–I signal of **2a** at *δ* 6.6 ppm in ^13^C NMR spectroscopy. In complex **3a**, the respective alkynyl carbon atom is found at *δ* 59.1 ppm. This signal resonates as a doublet with a small *J*_PC_ coupling constant (6.5 Hz), indicating that the alkynyl sits *cis* to phosphorus. Crystals of **3a** suitable for X-ray diffraction analysis were obtained by vapor diffusion of pentane into a dichloromethane solution at 4 °C (Scheme 1).^15^ The gold center adopts square-planar geometry, with tight coordination of the NMe_2_ group (the Au–N distance is short at 2.121(8) Å). The alkynyl group sits indeed *cis* to phosphorus. The Au–C≡C–Ph framework only marginally deviates from linearity, despite the proximity of the sterically demanding PAd_2_ group.

Oxidative addition of C(sp)–I bonds to gold was then generalized to substrate **2b** featuring a cyclohexyl instead of phenyl substituent. Here also the reaction was complete within the time of mixing. The ensuing (P,N)Au(iii) alkynyl complex **3b** (obtained in 92% yield) was characterized by multi-nuclear NMR spectroscopy and mass spectrometry. Besides extending the reaction to simpler and commercially available Au(i) precursors, the MeDalphos ligand imparts higher reactivity compared to bipyridine. The (P,N)-ligated complex **1** reacts readily with alkynyl iodides **2a,b** (instantaneous reactions with only 1 eq. of substrate) and forms monometallic complexes. For comparison, the (bipyridine)Au(i) (H_2_C=CH_2_)^+^ complex requires large excess of substrate (20 eq.) along with extended reaction times (1 h), and tends to form Au(i)/Au(iii) bimetallic complexes.^13^

**Scheme 2:**
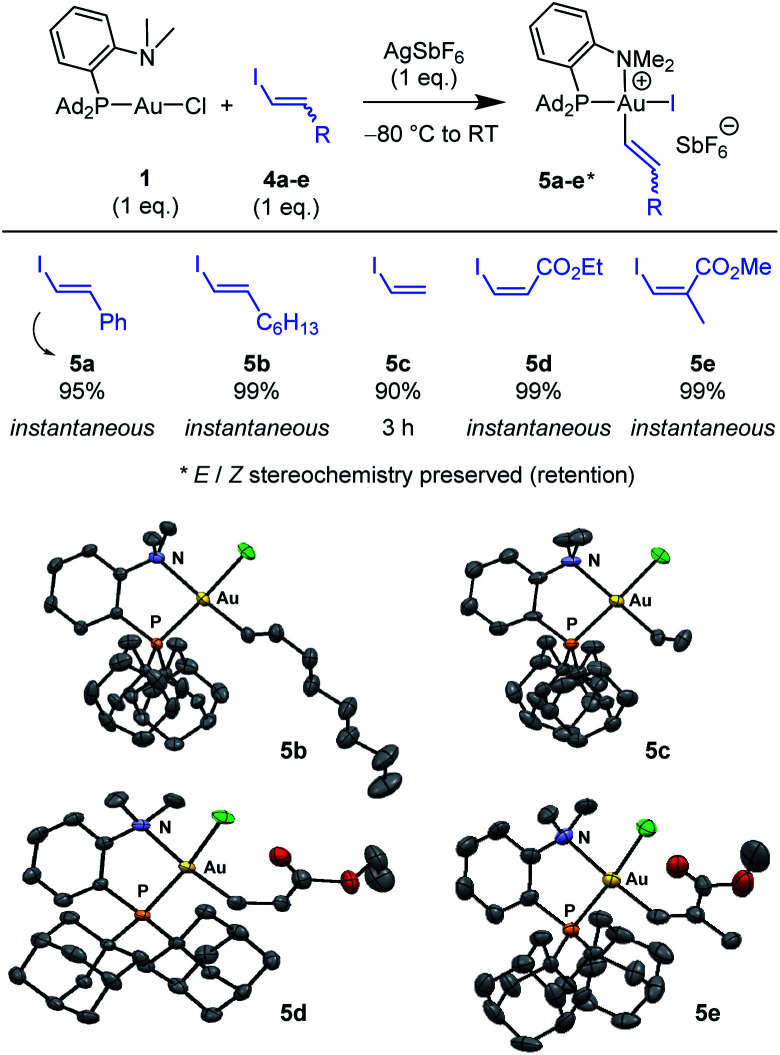
Oxidative addition of vinyl iodides to the (P,N)AuCl complex **1**. Reaction times and spectroscopic yields based on ^31^P NMR. Molecular structures of the (P,N)Au(iii) vinyl complexes **5b–e** (hydrogen atoms, counter anions and solvate molecules omitted for clarity, ellipsoids shown at 50% probability).

Next, the feasibility of oxidative addition of vinyl iodides to gold was explored (Scheme 2).^16^ First, the (P,N)AuCl complex **1** was activated by AgSbF_6_ and reacted with *trans* iodo styrene **4a**. The corresponding (P,N)Au(iii) vinyl complex **5a** was immediately and cleanly formed, as apparent from NMR spectroscopy. Besides the typical pattern for the MeDalphos ligand chelating a gold(iii) center (deshielded ^31^P NMR signal at *δ* 75.0 ppm and deshielded ^1^H NMR signal for the NMe_2_ group at *δ* 3.51 ppm), the ^1^H NMR spectrum shows two new signals characteristic of a CH=CH moiety (*δ* 6.96 and 6.50 ppm), with associated ^13^C NMR signals at *δ* 140.7 and 113.2 ppm. The absence of *J*_PC_ coupling supports *cis* relationship between the vinyl and PAd_2_ groups at gold while the large ^3^*J*_HH_ coupling constant (14.5 Hz) indicates that the *trans* geometry of the C=C bond is retained. Given that only styrenyl substrates undergo oxidative addition to the (bipyridine)Au(H_2_C=CH_2_)^+^ complex,^13^ we were eager to examine the scope of vinyl iodides prone to react with the (P,N)AuCl complex **1**. Gratifyingly, instantaneous and quantitative reactions were observed with the alkyl-substituted substrate **4b**, the parent vinyl iodide **4c** (oxidative addition takes 3 h in this case), as well as the ester-substituted substrates **4d,e**. The transformation thus tolerates substituents with different electronic bias at the C=C bond and it works with both *Z* and *E* substrates, including trisubstituted ones. In all cases, the *Z*/*E* stereochemistry of the substrate is preserved, oxidative addition to gold proceeds with retention.

X-ray diffraction analyses were carried out on complexes **5b–e** (Scheme 2), confirming the square-planar geometry of gold, the chelation of the (P,N) ligand with strong N → Au coordination, the *cis* relationship of the vinyl and PAd_2_ groups and the *Z*/*E* stereochemistry of the C=C double bond. Of note, the vinyl group is oriented perpendicularly to the gold coordination plane in all complexes to minimize steric constraints.

**Scheme 3:**
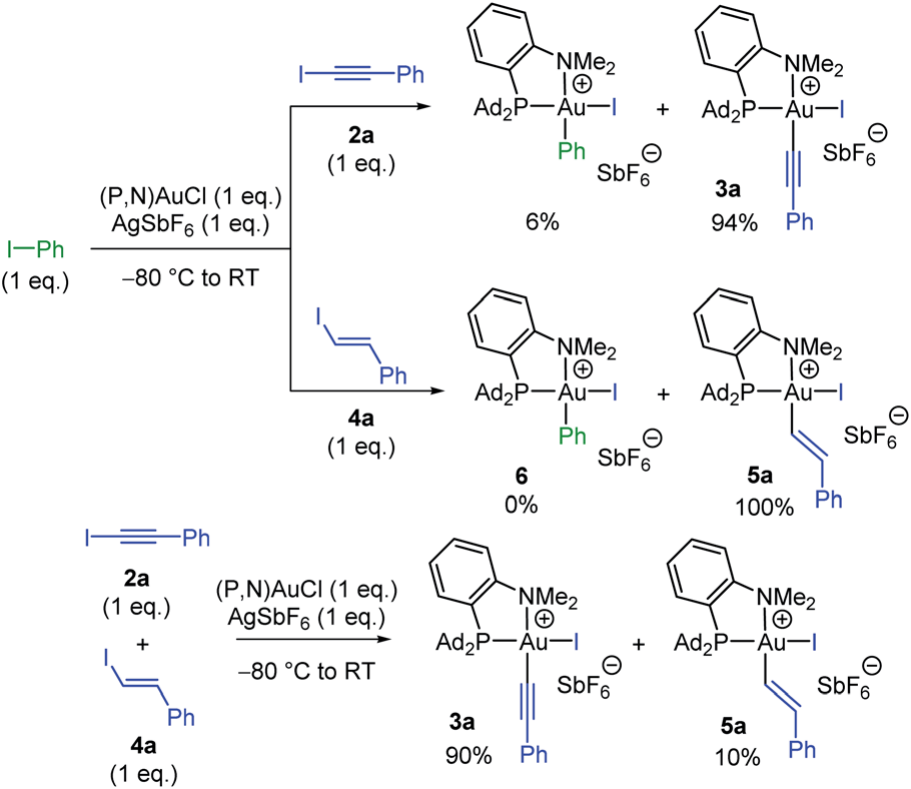
Competitive reactions between iodo benzene, iodo phenylacetylene **2a** and *trans* iodo styrene **4a** in the oxidative addition to the (P,N)AuCl complex **1**.

Having substantiated that oxidative addition of alkynyl and vinyl iodides takes place easily with the (P,N)AuCl complex **1**, we were then keen to assess the relative reactivity of these substrates compared to aryl iodides. To this end, competitive experiments were performed between iodo benzene, iodo phenylacetylene **2a** and *trans* iodo styrene **4a** (Scheme 3). In all cases, equimolar amounts of the two iodides were combined with 1 eq. of the (P,N)AuCl complex **1** and 1 eq. of AgSbF_6_. The proportion of the ensuing Au(iii) complexes was determined by ^31^P and ^1^H NMR spectroscopy at complete consumption of the Au(i) complex.^15^ The competition between Ph–I and Ph–C≡C–I revealed strong preference for C(sp)–I oxidative addition, the Au(iii) alkynyl and aryl complexes being obtained in 94/6 ratio, while complete selectivity for oxidative addition of the vinyl iodide was observed between Ph–I and Ph–CH=CH–I. Thus, both alkynyl and vinyl iodides react faster than aryl iodides with the (P,N)AuCl complex **1**.^17,18^ Furthermore, C(sp)–I prevailed over C(sp^2^)–I oxidative addition in the competition between Ph–C≡C–I and Ph–CH=CH–I.

Note that the (P,N) ligand and silver salt are needed for the C(sp)/C(sp^2^)–I oxidative addition to take place at gold. No reaction occurs with the (P,N)AuCl complex alone, with PhC≡C–I as well as H_13_C_6_–CH=CH–I. In the case of N-heterocyclic carbene complex (IPr)AuCl, only the π-complex was formed, while (Ph_3_P)AuCl gave mainly the inactive (Ph_3_P)_2_Au^+^ species.^15^

Our next goal was to demonstrate that the (P,N) gold complex not only undergoes oxidative addition of alkynyl and vinyl iodides, but also enables catalytic cross-coupling transformations to be achieved.^19^ Given that the (P,N) ligand triggers fast, quantitative and irreversible addition of C(sp)/C(sp^2^)–I bonds to gold, we were interested in combining oxidative addition of alkynyl/vinyl iodides and π-activation at gold. To this end, the coupling of alkenols with alkynyl and vinyl iodides was investigated as an attractive extension of the recently reported hetero-arylation reactions of alkenes.^20^ Unfortunately, no hetero-alkynylation product could be achieved from iodo phenylacetylene and 4-penten-1-ol. In fact, no oxidative addition occurred and only the Au(i) π-complex with 4-penten-1-ol was observed under these conditions. However, positive results were obtained with vinyl iodides, as detailed hereafter. The coupling of *trans* iodo oct-1-ene with 4-penten-1-ol was used to optimize the reaction conditions. The silver salt, base, solvent, concentration, temperature and catalytic loading were varied (Table 1). The hetero-vinylation product **7a** was obtained in 99% yield within 2 h using 5 mol% of (P,N)AuCl, AgSbF_6_ (1.05 eq.) and K_3_PO_4_ (1 eq.) in DCE at 0.4 M and 80 °C (entry 1). The use of other halide scavengers and bases results in lower yields (entries 2–9). The reaction proceeds equally well at 45 °C and can even be performed at room temperature if prolonged for 16 h (entries 10–11). Working in other solvents and lowering the concentration (0.1 M) also results in somewhat decreased efficiency. However, the catalytic loading can be reduced to 1 mol% (entry 17) and the reaction can be performed in air with technical grade solvents^15^ without noticeable loss of catalytic activity, demonstrating good robustness.

**Table 1:** Optimization of the catalytic conditions^a^

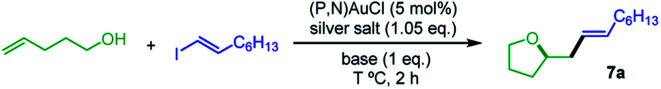					
Entry	Ag salt	Base	Solvent	*T* (°C)	Yield^b^ (%)
**1**	**AgOTf**	**K**_**3**_ **PO**_**4**_	**DCE**	**80**	**99**
2	AgSbF_6_	K_3_PO_4_	DCE	80	50
3	AgPF_6_	K_3_PO_4_	DCE	80	77
4	AgBF_4_	K_3_PO_4_	DCE	80	88
5	AgOTf	DTBP	DCE	80	92
6	AgOTf	Cs_2_CO_3_	DCE	80	58
7	AgOTf	NaOAc	DCE	80	50
8	AgOTf	DIEA	DCE	80	21
9	AgOTf	-	DCE	80	6
**10**	**AgOTf**	**K**_**3**_ **PO**_**4**_	**DCE**	**45**	**99**
11^c^	AgOTf	K_3_PO_4_	DCE	25	84
12	AgOTf	K_3_PO_4_	*o*-DCB	80	95
13	AgOTf	K_3_PO_4_	toluene	80	88
14	AgOTf	K_3_PO_4_	DCM	45	89
15	AgOTf	K_3_PO_4_	chloroform	45	84
16^d^	AgOTf	K_3_PO_4_	DCE	80	90
**17** e	**AgOTf**	**K**_**3**_ **PO**_**4**_	**DCE**	**80**	**98**

a Reactions performed at 0.4 M concentration with equimolar amounts of alkenol and vinyl iodide (0.4 mmol scale).

b Yield determined by ^1^H NMR using dimethyl terephthalate as internal standard.

c 16 h.

d Reaction performed at 0.1 M concentration.

e Reaction performed with 1 mol% of (P,N)AuCI and 1.01 eq. of AgOTf. DTBP = 2,6-di-*tert*-butylpyridine.

**Scheme 4:**
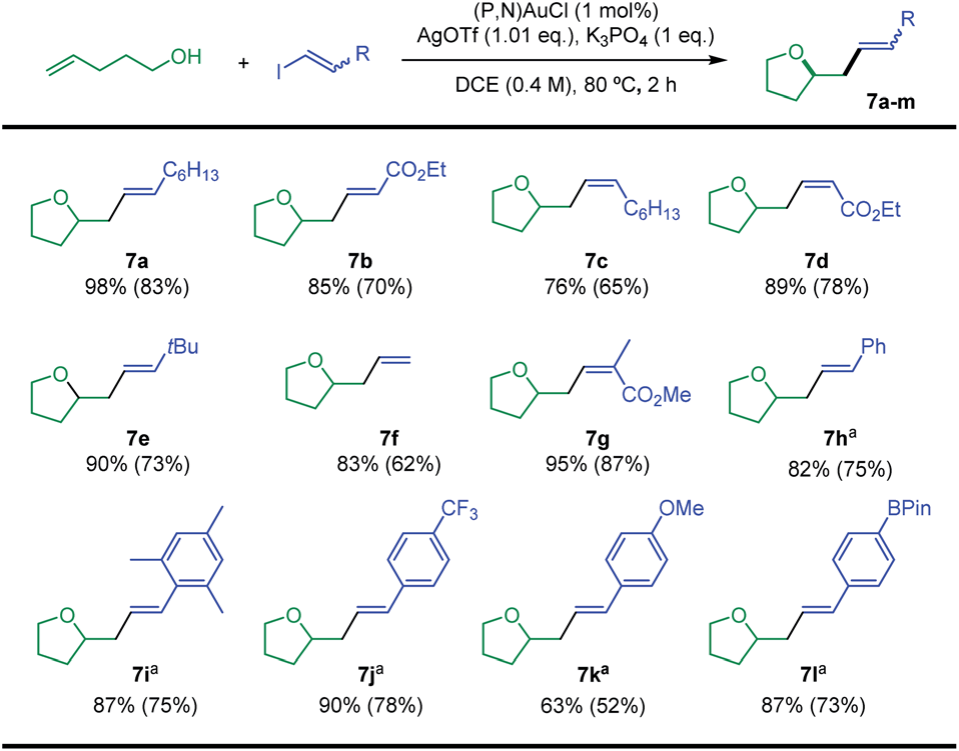
Gold-catalyzed oxy-vinylation, scope of vinyl iodides. Yields determined by ^1^H NMR spectroscopy with dimethyl terephthalate as internal standard. Isolated yields in parentheses. ^a^25 °C, 16 h.

With the optimized conditions in hands, the reaction of 4-penten-1-ol with different vinyl iodides was studied. Good to excellent yields were observed in all cases (Scheme 4), illustrating the generality and efficiency of the transformation. *Trans* iodo ethyl acrylate gave the corresponding oxy-vinylation product **7b** in good yield. The reaction works well and with complete retention of stereochemistry from *cis* vinyl iodides, as substantiated by the formation of **7c** and **7d**. Good results were also obtained when preparing **7e–g** from the parent, the *t*Bu and the tri-substituted substrates, indicating that the reaction is not very sensitive to the steric demand of the vinyl iodide. With *trans* iodo styrenes, the transformation was most conveniently achieved at 25 °C over 16 h (to minimize degradation of the vinyl iodide). The corresponding oxy-vinylation products **7h–l** were obtained in good yields whatever the steric demand and electronic bias of the substrate. The efficient preparation of **7l** is particularly noteworthy, no interference was observed with the Bpin moiety.

**Scheme 5:**
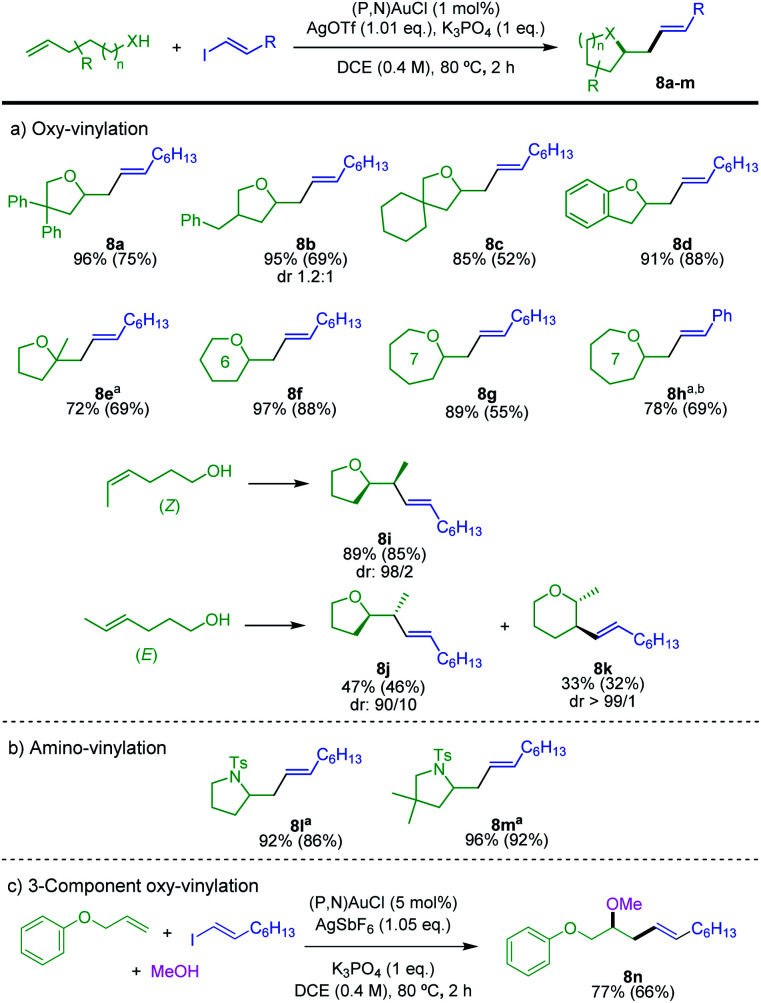
Gold-catalyzed hetero-vinylation, scope of alkenols and *N*-tosyl alkenamines, as well as 3-component oxy-vinylation. Yields determined by ^1^H NMR spectroscopy with dimethyl terephthalate as internal standard. Isolated yields in parentheses. ^a^5 mol% (P,N)AuCl, 1.05 eq. AgOTf. ^b^25 °C, 16 h.

The scope of alkenols was then assessed (Scheme 5a). The oxy-vinylation tolerates aryl and alkyl substitution on 4-penten-1-ol, as illustrated by the preparation of the tetrahydrofurane derivatives **8a–c**. It works well with *ortho*-allyl phenol to give the benzo-fused product **8d**. It is also efficient with *gem*-disubstituted alkenols and enables the formation of quaternary centers as in **8e**. In addition, six and seven-membered ring products **8f–h** are formed in high yields from 5-hexen-1-ol and 6-hepten-1-ol. In all cases, the terminal alkenols undergo *exo* cyclization with complete regioselectivity. Internal alkenols are also suitable substrates, as substantiated by the oxy-vinylation of 4-hexen-1-ols. Of note, the reaction of the *Z* isomer is fully regioselective for 5-*exo* cyclization, while 5-*exo* and 6-*endo* cyclizations compete for the *E* isomer. The corresponding tetrahydrofurane/tetrahydropyrane products **8j**/**8k** are obtained in 47 and 33% yields, respectively. A similar tendency was observed for the oxy-arylation, with in the latter case complete switch of regioselectivity from 5-*exo* to 6-*endo* between the *Z* and *E* isomers of the alkenol.^5*d*^ The transformation is applicable to *N*-tosyl alkenamines as well. Amino-vinylation proceeds nicely to give the pyrrolidine products **8l** and **8m** in >92% yields (Scheme 5b). The feasibility of 3-component oxy-vinylation of alkenes^5*e*^ was also substantiated by the preparation of product **8n** (Scheme 5c).

**Scheme 6:**
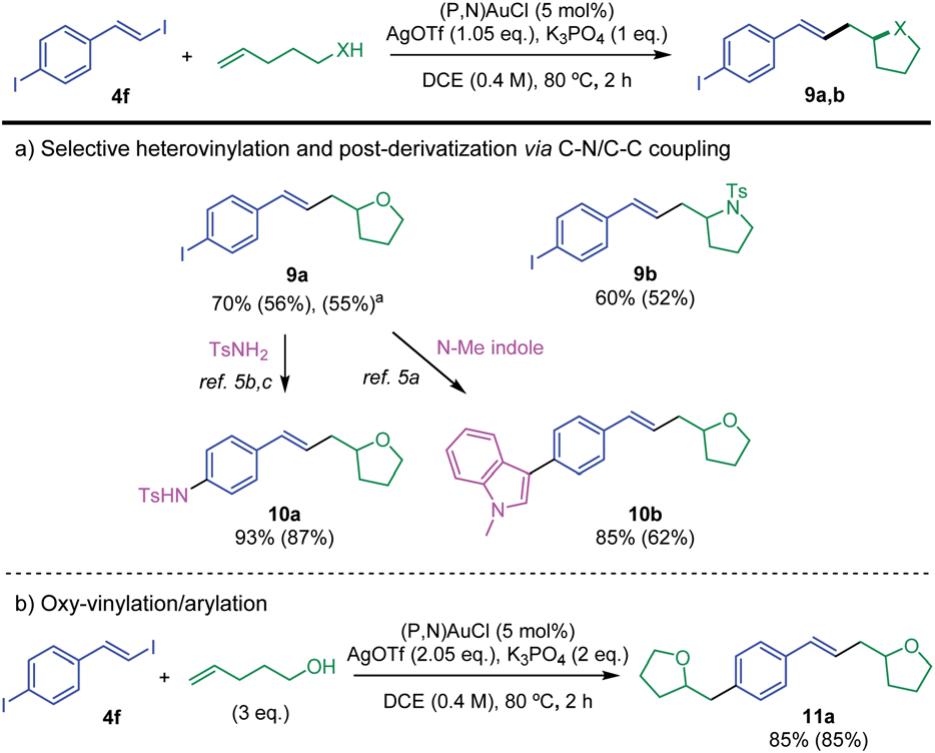
Selective and sequential transformation of the bifunctional substrate **4f**. Yields determined by ^1^H NMR spectroscopy with dimethyl terephthalate as internal standard. Isolated yields in parentheses. ^a^Gram scale reaction (4 mmol).

Then, the preference observed for oxidative addition of vinyl–I over Ar–I bonds to gold prompted us to explore the possibility to achieve chemoselective catalytic transformation of the difunctional substrate **4f** featuring both vinyl–I and aryl–I moieties.^21^ Pleasingly, using 1 eq. of 4-penten-1-ol or *N*-tosyl 4-penten-1-amine, the oxy- and amino-vinylations are largely predominant. The tetrahydrofurane and pyrrolidine products **9a,b** featuring iodo styryl arms were obtained in good yields (Scheme 6a). ^1^H NMR analysis of the crude reaction mixture shows no oxy-arylation product and only a small amount of oxy-arylation/oxy-vinylation product resulting from the transformation of both C(sp^2^)–I bonds of **4f** (7% with respect to **9a**). The synthesis of **9a** was scale up to 4 mmol, demonstrating the robustness and practical interest of the transformation. Further derivatization of **9a** was achieved using the ability of the (P,N) gold(i) complex to catalyze C–N and C–C coupling.^5*a*–*c*^ Using *N*-tosyl amine and *N*-methyl indole, the functionalized tetrahydrofuranes **10a,b** were obtained in high yields. In addition, using 3 eq. of 4-penten-1-ol, the oxy-vinylation and oxy-arylation reactions were found to readily proceed giving the styryl-bridged bis-tetrahydrofurane **11a** in 85% yield (Scheme 6b).

**Scheme 7:**
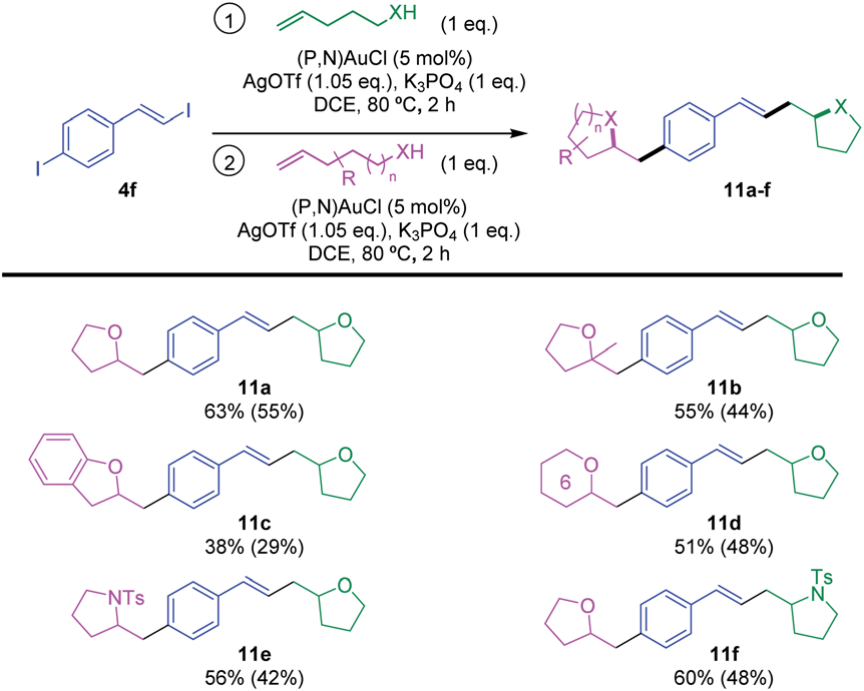
Sequential gold-catalyzed hetero-vinylation/arylation transformations of the bifunctional substrate **4f**. Yields over the two steps determined by ^1^H NMR spectroscopy with dimethyl terephthalate as internal standard. Isolated yields in parentheses.

Finally, we aimed to take advantage of the reactivity of **4f** to achieve sequential hetero-vinylation/hetero-arylation catalytic transformations in one-pot and obtain thereby original heterocyclic products (Scheme 7). To start with, the sequential reaction of **4f** with 2 eq. of 4-penten-1-ol was studied. Once the oxy-vinylation reaction achieved with the first eq. of alkenol, another load of gold complex and silver salt was required for the oxy-arylation to proceed when adding the second eq. of 4-penten-1-ol. The bis-tetrahydrofurane **11a** was obtained in good yield (63% over two steps) under these conditions. With this procedure in hands, different alkenols were then used for the oxy-vinylation and oxy-arylation steps. Unsymmetrical products **11b–d** were thereby obtained in moderate to good yields (38–55% over two steps), combining 4-penten-1-ol with 4-methyl-4-penten-1-ol, *ortho*-allyl phenol and 5-hexen-1-ol, respectively. This reaction sequence also works with *N*-tosyl alkenamines, allowing for example the access to **11e** and **11f** combining tetrahydrofurane and pyrrolidine moieties. Thanks to the vinyl–I/aryl–I chemoselectivity, it is straightforward to obtain these two constitutional isomers by simply changing the order of addition of the alkenol and *N*-tosyl alkenamine.

**Fig. 2:**
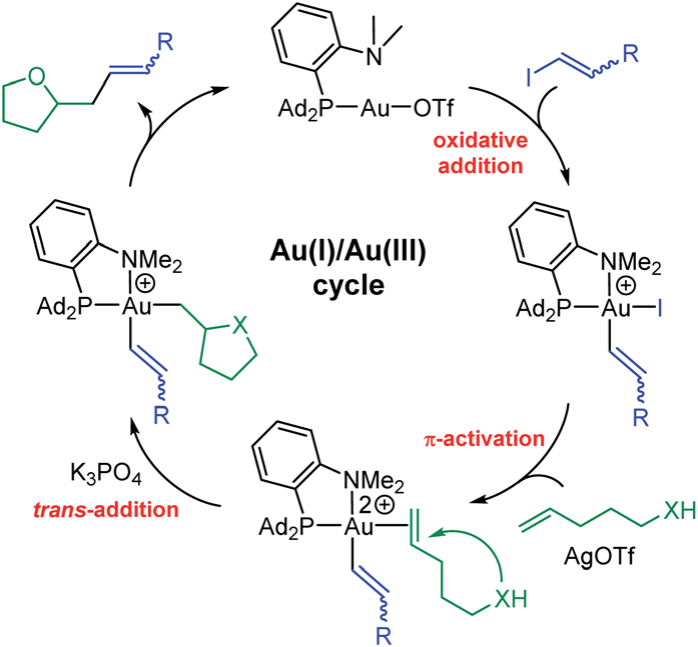
Catalytic cycle proposed to account for oxy-/amino–vinylation reactions catalyzed by the (P,N)Au(i) complex.

From a mechanistic viewpoint, the hetero-vinylation reactions catalyzed by the (P,N)Au(i) complex are proposed to occur *via* the catalytic cycle displayed in Fig. 2. As for the corresponding hetero-arylation, it starts by oxidative addition of the vinyl iodide to gold, then involves π-activation and cyclization of the alkenol at gold, and the product is finally released upon C(sp^2^)–C(sp^3^) reductive elimination. In line with this picture, similar results were obtained using the gold(iii) vinyl complex **5b** resulting from oxidative addition of *trans* iodo oct-1-ene as catalyst.^15^ In addition, NMR monitoring of a catalytic run identified the alkenol/gold(i) π-complex as resting state of the transformation.^22^ The proposed mechanism is consistent with the complete *trans* selectivity observed experimentally upon addition of the vinyl group and O atom to the C=C bond, as apparent in the reaction of the internal alkenols leading to **8i,j,k**.

## Conclusions

The MeDalphos ligand triggers oxidative addition of alkynyl and vinyl iodides to gold under mild conditions. The corresponding (P,N)-chelated organo Au(iii) complexes form quickly, quantitatively and irreversibly. They are readily isolated and have been fully characterized. The reaction works with a broad range of substrates of various electronic bias and steric demand, including vinyl iodide itself and iodoacrylates. *Z* and *E* vinyl iodides are activated and the oxidative addition proceeds with complete retention of stereochemistry.

In the case of vinyl iodides, this elementary step could be used as an entry point for a catalytic transformation merging Au(i)/Au(iii) redox cycling and π-activation at gold. The (P,N)Au complex proved efficient and general in promoting the hetero-vinylation of alkenols and *N*-tosyl alkenamines.

The relative reactivity of vinyl and aryl iodides has been investigated *via* competitive experiments. This revealed high chemoselectivity of both the oxidative addition and catalytic coupling in favour of the vinyl substrates. This chemoselectivity was exploited synthetically with a vinyl/aryl bis-iodide substrate. Several polyfunctional tetrahydrofurane products were obtained straightforwardly by sequential hetero-vinylation/arylation.

These results further demonstrate the interest and synthetic value of “non-innocent” ligands such as MeDalphos in 2-electron redox gold chemistry. Future work will aim to expand the variety of suitable substrates and achievable catalytic transformations, as well as to develop new ligand frameworks.

## Author contributions

J. R. and A. T. performed the experiments; J. R., A. T. and D. B. analyzed the experimental data; and S. M.-L. collected and refined the X-ray diffraction data. All the authors contributed to scientific discussion and participated in writing the manuscript. D. B. guided the research.

## Conflicts of interest

There are no conflicts to declare.

## Supplementary Material

SC-012-D1SC01483H-s001

SC-012-D1SC01483H-s002
