# Supplementary material for: Oxidative additions of alkynyl/vinyl iodides to gold and gold-catalyzed vinylation reactions triggered by the MeDalphos ligand
Source: Chem Sci. 2021 Apr 28;12(22):7706–12. doi: 10.1039/d1sc01483h (PMC8188461; doi:10.1039/d1sc01483h)
Supplement: SC-012-D1SC01483H-s001 [file SC-012-D1SC01483H-s001.pdf]

Electronic Supplementary Material (ESI) for Chemical Science.
This journal is © The Royal Society of Chemistry 2021

Supporting Information

Oxidative additions of alkynyl/vinyl iodides to gold and gold-catalyzed vinylation reactions triggered by the MeDalphos Ligand

Jessica Rodriguez,[‡a] Alexis Tabey,[‡a] Sonia Mallet-Ladeira,[b] and Didier Bourissou*[a]

Table of contents

1. Materials and methods S3
2. Experimental procedures and analytical data S3
2.1 General procedures for vinyl and alkynyl iodides S3
2.1.1 Synthesis of vinyl iodide substrates S3
2.1.2 Synthesis of alkynyl iodide substrates S4
2.2 Oxidative addition reactions and competition experiments S4
2.2.1 Oxidative addition reactions S4
2.2.1.1 Reaction of (P,N)AuCl with alkynyl iodides S4
2.2.1.2 Reaction of (P,N)AuCl with vinyl iodides S5
2.2.1.3 Reaction of (IPr)AuCl with $H_{13}C_6$–CH=CH–I S7
2.2.2 Competition experiments S9
2.2.2.1 Competition reaction between (iodoethynyl)benzene and iodobenzene S9
2.2.2.2 Competition reaction between trans-styryl iodide and iodobenzene S10
2.2.2.3 Competition reaction between trans-styryl iodide and (iodoethynyl)benzene S12
2.2.3 Oxidative addition of (iodoethynyl)benzene in the presence of 4-penten-1-ol S13
2.3 Gold-catalyzed coupling of vinyl iodide and 4-penten-1-ol S14
2.4 Gold-catalyzed oxy-vinylation of alkenes S14
2.4.1 Synthesis of alkenol substrates S14
2.4.2 General procedure for the gold-catalyzed oxy-vinylation of alkenes S15
2.4.3 Catalytic oxy-vinylation under air with reagent-grade chemicals S19
2.4.4 Catalytic oxy-vinylation catalyzed by gold(III) complex S19
2.4.5 Monitoring catalytic oxy-vinylation catalyzed (MeDalphos)AuCl S20
2.5 Gold-catalyzed Amino-vinylation of alkenes S20
2.5.1 Synthesis of substrates S20
2.5.2 General procedure for the gold-catalyzed Amino-vinylation of alkenes S20
2.6 Gold-catalyzed 3-component oxyvinylation S21
2.7 Gold-catalyzed heterovinylation/heteroarylation of alkenes S22
2.7.1 General procedure for the gold-catalyzed heterovinylation from bifunctional substrate S22
2.7.2 Synthetic application from **9a** S22
2.7.3 General procedure for the gold-catalyzed oxy-vinylation/oxy-arylation from bifunctional substrate S23
2.7.4 General procedure for the gold-catalyzed heterovinylation/heteroarylation from bifunctional substrate S24
3. Crystallographic Data S25
4. References S29
5. NMR spectra S30
5.1 NMR spectra of complexes S30
5.2 NMR spectra of vinyl iodides S40
5.3 NMR spectra of hetero-vinylation products S42
5.4 NMR spectra of heterovinylation/heteroarylation products S66

1. Materials and methods

Unless otherwise stated, all reactions and manipulations were carried out under an atmosphere of dry argon using standard Schlenk techniques or in a glovebox under an inert atmosphere. Dry, oxygen-free solvents were employed. Solution ^{1}H, ^{13}C, ^{31}P, ^{19}F NMR spectra were recorded on Bruker Avance 300, 400 or 500 spectrometers at 298K unless otherwise stated. Chemical shifts are expressed with a positive sign, in parts per million, calibrated to residual ^{1}H and ^{13}C solvent signals. The following abbreviations and their combinations are used: br, broad; s, singlet; d, doublet; t, triplet; q, quartet; quin, quintuplet; m, multiplet. GC-MS analyses were performed on a MS Perkin Elmer Clarus MS560 and GC PerkinElmer Clarus 500. Mass spectra were recorded on a Waters LCT apparatus. Melting points were determined on a Stuart SMP-42 automatic melting-point apparatus. All starting materials were purchased from Aldrich, Fluorochem and used as received unless otherwise stated.

2. Experimental procedures and analytical data

2.1 General procedures for vinyl and alkynyl iodides

2.1.1 Synthesis of vinyl iodide substrates

(*E*)-(2-iodovinyl)benzene,[1] (*E*)-1-(2-iodovinyl)-4-(trifluoromethyl)benzene,[1] (*E*)-1-iodo-4-(2-iodovinyl)benzene,[2] (*E*)-1-(2-iodovinyl)-4-methoxybenzene,[3] (*E*)-1-iodo-3,3-dimethylbut-1-ene,[3] (*Z*)-(2-iodovinyl)benzene,[4] methyl (*Z*)-3-iodo-2-methylacrylate,[5] (*Z*)-1-iodooct-1-ene[6] were prepared according to reported procedures.

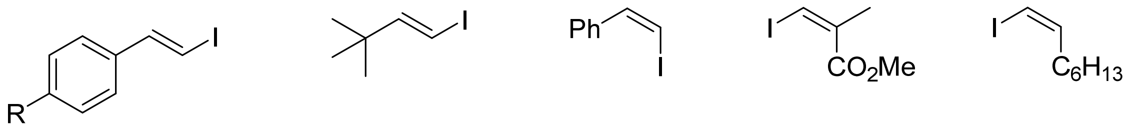

R = H, CF_3, I, OMe

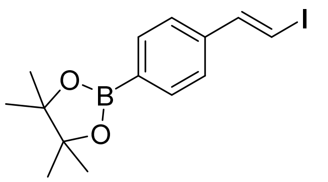

(*E*)-2-(4-(2-iodovinyl)phenyl)-4,4,5,5-tetramethyl-1,3,2-dioxaborolane: Following literature procedure,[1] a solution of CH_2I_2 (0.32 mL, 4.0 mmol) in THF (1.0 mL) was added dropwise to a −78 °C solution of LiHMDS (670 mg, 4.0 mmol) in THF (4mL) and ether (4 mL) in the dark. After 20 min, a solution of 4-(4,4,5,5-tetramethyl-1,3,2-dioxaborolan-2-yl)benzyl bromide (594 mg, 2.0 mmol) in THF (1.5 mL) was added dropwise. The reaction mixture was allowed to slowly warm to rt. After 16 h, DBU (0.6 mL, 4.0 mmol) was added dropwise, stirred for 1 h, and then diluted with Et_2O (25 mL). The mixture was filtered through a plug of celite/silica (approximately 3 cm celite over 3 cm silica) and the solvent removed under reduced pressure. The filtrate was concentrated under reduced pressure, and the residue was purified by SiO_2 chromatography using pentane/ethyl acetate to give (*E*)-2-(4-(2-iodovinyl)phenyl)-4,4,5,5-tetramethyl-1,3,2-dioxaborolane (101 mg, 15%,) as an yellow oil. (pentane/ethyl acetate: 96/4, R_f: 0.5); **^{1}H NMR (300 MHz, CDCl$_3$) δ** 7.76 (d, *J* = 8.1 Hz, 2H), 7.44 (d, *J* = 14.9 Hz, 1H), 7.29 (d, *J* = 7.8 Hz, 2H), 6.92 (d, *J* = 14.9 Hz, 1H), 1.34 (s, 12H). **^{13}C NMR (75 MHz, CDCl$_3$) δ** 145.2, 135.4, 135.3 (2 C_{ar}), 128.2, 125.4 (2 C_{ar}), 84.0 (2 C_q), 78.2, 25.0 (4 CH_3). **EI-MS:** Calculated for $C_{14}H_{18}BIO_2$: 356.0445, Found: 355.9172.

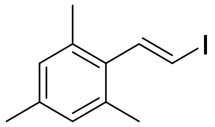

(*E*)-2-(2-iodovinyl)-1,3,5-trimethylbenzene: Following literature procedure,[1] a solution of CH_2I_2 (0.32 mL, 4.0 mmol) in THF (1.0 mL) was added dropwise to a −78 °C solution of LiHMDS (670 mg, 4.0 mmol) in THF (4mL) and ether (4 mL) in the dark. After 20 min, a solution of 2-(bromomethyl)-1,3,5-trimethylbenzene (426 mg, 2.0 mmol) in THF (1.5 mL) was added dropwise. The reaction mixture was allowed to slowly warm to rt. After 16 h, DBU (0.6 mL, 4.0 mmol) was added dropwise, stirred for 16 h, and then diluted with Et_2O (25 mL). The mixture was filtered

through a plug of celite/silica (approximately 3 cm celite over 3 cm silica) and the solvent removed under reduced pressure. The filtrate was concentrated under reduced pressure, and the residue was purified by SiO_2 chromatography using 100% pentane to give (*E*)-2-(2-iodovinyl)-1,3,5-trimethylbenzene (327 mg, 60%) as an oil. TLC: Rf = 0.8 (100% pentane); **^{1}H NMR (300 MHz, $CDCl_3$) δ** 7.43 (d, *J* = 15.1 Hz, 1H), 6.86 (s, 2H), 6.28 (d, *J* = 15.1 Hz, 1H), 2.25 (s, 9H). **^{13}C NMR (75 MHz, $CDCl_3$) δ** 143.6, 137.3, 135.7, 134.9, 128.9, 80.0, 21.1, 20.9. **EI-MS:** Calculated for $C_{11}H_{13}I$: 272.0062, Found: 271.9172.

2.1.2 Synthesis of alkynyl iodide substrates

(iodoethynyl)benzene and (iodoethynyl)cyclohexane were prepared according to reported procedures.[7]

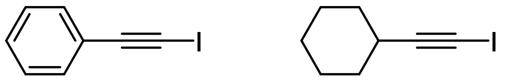

2.2 Oxidative addition reactions and competition experiments

2.2.1 Oxidative addition reactions

2.2.1.1 Reaction of (P,N)AuCl with alkynyl iodides

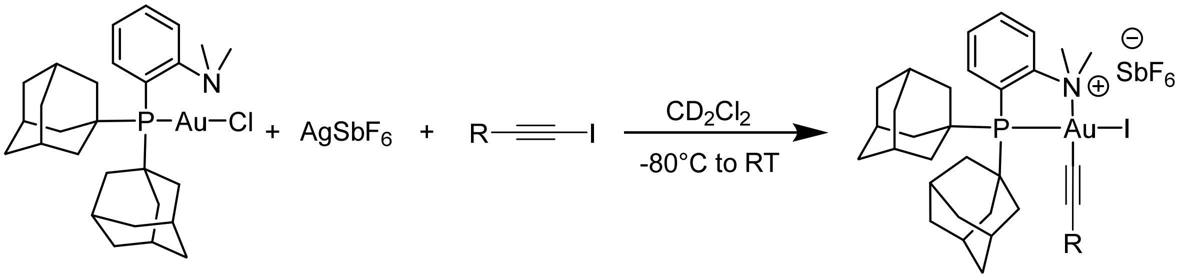

(1 eq.) (1 eq.) (1 eq.)

In a glovebox, a screw-cap NMR tube was charged with silver hexafluoroantimonate (8.0 mg, 0.023 mmol) in CD_2Cl_2 (0.3 mL). (MeDalphos)AuCl complex (15 mg, 0.023 mmol, 1 eq.) was transferred into a small glass vial and dissolved in CD_2Cl_2 (0.3 mL). The alkynyl iodide (0.023 mmol, 1 eq.) was added to the solution of (MeDalphos)AuCl complex. The prepared solution was loaded into a plastic syringe equipped with stainless steel needle. The syringe was closed by blocking the needle with a septum. Outside the glovebox, the NMR tube was cooled down to -80°C (Ethanol/N_2 cold bath). At this temperature, the solution of (MeDalphos)AuCl complex and alkynyl iodide was added. The tube was gently shaken and allowed to warm to RT. The formation of the Au(III) complex was confirmed by ^{1}H and ^{31}P NMR spectroscopy as well as high-resolution mass spectrometry (Electrospray ionization, positive mode). Iodine to chloride exchange reaction from Au(III) complexes was systematically observed with all substrates. In the following examples, only the mass peak corresponding to the Au(III) chloride derivatives is indicated. The solution was filtered over celite and the solvent evaporated under vacuum. Crystals suitable for X-ray diffraction analysis were obtained by layering a DCM solution with pentane or diethyl ether.

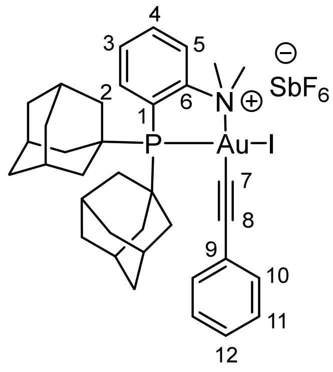

Complex **3a** was prepared using (iodoethynyl)benzene and obtained instantaneous in 99% yield. **^{1}H NMR (500 MHz, CD_2Cl_2): δ** 8.09 (dddd, J_{HH} = 8.7 Hz, J_{HH} = 7.0 Hz, J_{HH} = 1.7 Hz, 1H, H_2), 8.05 – 7.96 (m, 2H, H_4 & H_5), 7.86 (dddd, J_{HH} = 8.1 Hz, J_{HH} = 7.1 Hz, J_{HP} = 2.6 Hz, J_{HH} = 1.1 Hz, 1H, H_3), 7.49 – 7.45 (m, 2H, H_{10}), 7.43 (m, 3H, H_{11} & H_{12}), 3.68 (s, 6H, $N(CH_3)_2$), 2.43 (dd, J_{HP} = 6.3 Hz, J_{HH} = 3.0 Hz, 12H, H_{Ad}), 2.27 – 2.13 (m, 6H, H_{Ad}), 1.82 (t, J_{HH} = 3.0 Hz, 12H, H_{Ad}). **^{31}P{^{1}H} NMR (162 MHz, CD_2Cl_2): δ** 102.7 (s). **^{13}C NMR (125 MHz, CD_2Cl_2): δ** 159.2 (d, J_{PC} = 6.5 Hz, C_6), 137.5 (d, J_{PC} = 2.3 Hz, C_2), 135.5 (d, J_{PC} = 1.9 Hz, C_4), 131.5 (d, J_{PC} = 7.6 Hz, C_3), 131.3 (s, C_{10}), 129.0 (s, C_{12}), 128.6 (s, C_{11}), 125.0 (d, J_{PC} = 8.2 Hz, C_5), 123.1 (s, C_9), 117.6 (d, J_{PC} = 43.7 Hz, C_1), 108.6 (s, C_8), 59.1 (d, J_{PC} = 6.5 Hz, C_7), 55.3 (s, $N(CH_3)_2$), 48.6 (d, J_{PC} = 11.0 Hz, C_{qAd}), 40.6 (d, J_{PC} = 2.4 Hz, CH_{2Ad}), 35.3 (d, J_{PC} = 1.8 Hz, CH_{2Ad}), 28.7 (d, J_{PC} = 10.0 Hz, CH_{Ad}). **HRMS (ESI$^+$)**: calculated for [M^+] = $C_{36}H_{45}NPClAu^+$: 754.2644. Found: 754.2650.

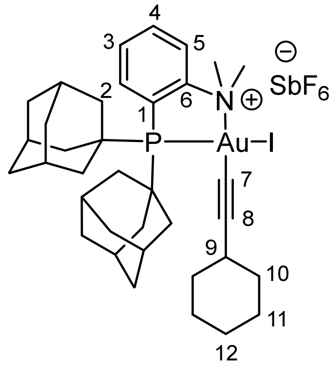

Complex **3b** was prepared using (iodoethynyl)cyclohexane and obtained instantaneous in 99% yield. **^{1}H NMR (500 MHz, CD_2Cl_2): δ** 8.06 (m, 1H, H_2), 8.02 – 7.94 (m, 2H, H_4 & H_5), 7.93 – 7.79 (m, 1H, H_3), 3.62 (s, 6H, $N(CH_3)_2$), 2.49 – 2.29 (m, 12H, H_{Ad}), 2.29 – 2.02 (m, 9H, $6H_{Ad}$ & H_9 & $2H_{Chx}$), 2.02 – 1.90 (m, 1H, H_{Chx}), 1.90 – 1.67 (m, 16H, $12H_{Ad}$ & $4H_{Chx}$), 1.68 – 1.29 (m, 3H, H_{Chx}). **^{31}P{^{1}H} NMR (162 MHz, CD_2Cl_2): δ** 98.8 (s). **^{13}C NMR (125 MHz, CD_2Cl_2): δ** 159.1 (d, J_{PC} = 6.4 Hz, C_6), 137.3 (s, C_2), 135.4 (s, C_4), 131.3 (d, J_{PC} = 7.5 Hz, C_3), 124.9 (d, J_{PC} = 8.1 Hz, C_5), 117.7 (d, J_{PC} = 43.8 Hz, C_1), 55.1 (s, $N(CH_3)_2$), 48.5 (d, J_{PC} = 11.5 Hz, C_{qAd}), 42.6 (s, CH_{2Chx}), 40.6 (s, CH_{2Ad}), 35.7 (s, CH_{2Chx}), 35.3 (s, CH_{2Ad}), 32.8 (s, CH_{2Chx}), 28.8 (s, C_9), 28.6 (d, J_{PC} = 10.0 Hz, CH_{Ad}), 25.7 (s, CH_{2Chx}), 25.1 (s, CH_{2Chx}). C_7 and C_8 not observed. **HRMS (ESI$^+$)**: calculated for [M^+] = $C_{36}H_{51}NPClAu^+$: 760.3113. Found: 760.3117.

2.2.1.2 Reaction of (P,N)AuCl with vinyl iodides

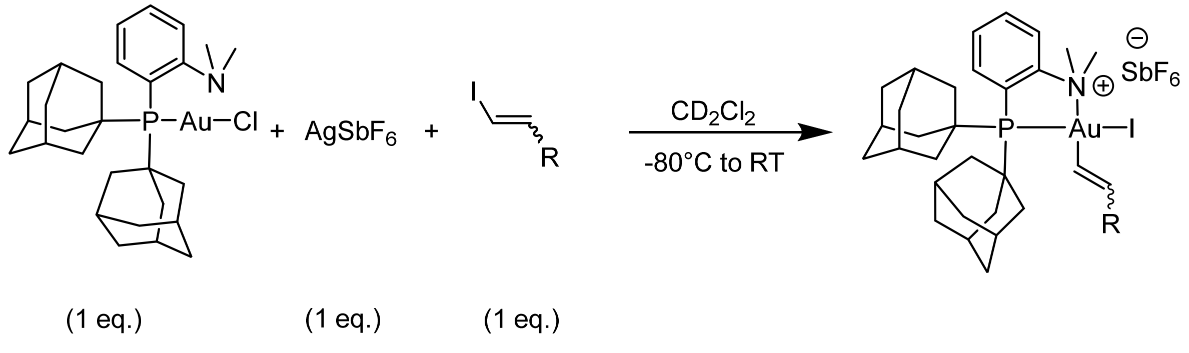

In a glovebox, a screw-cap NMR tube was charged with silver hexafluoroantimonate (8.0 mg, 0.023 mmol) in CD_2Cl_2 (0.3 mL). (MeDalphos)AuCl complex (15 mg, 0.023 mmol, 1 eq.) was transferred into a small glass vial and dissolved in CD_2Cl_2 (0.3 mL). The vinyl iodide (0.023 mmol, 1 eq.) was added to the solution of (MeDalphos)AuCl complex. The prepared solution was loaded into a plastic syringe equipped with stainless steel needle. The syringe was closed by blocking the needle with a septum. Outside the glovebox, the NMR tube was cooled down to -80°C (Ethanol/N_2 cold bath). At this temperature, the solution of (MeDalphos)AuCl complex and vinyl iodide was added. The tube was gently shaken and allowed to warm to RT. The formation of the Au(III) complex was confirmed by ^{1}H and ^{31}P NMR spectroscopy as well as high-resolution mass spectrometry (Electrospray ionization, positive mode). Iodine to chloride exchange reaction from Au(III) complexes was systematically observed with all substrates. In the following examples, only the

mass peak corresponding to the Au(III) chloride derivatives is indicated. The solution was filtered over celite and the solvent evaporated under vacuum. Crystals suitable for X-ray diffraction analysis were obtained by layering a DCM solution with pentane or diethyl ether in fridge.

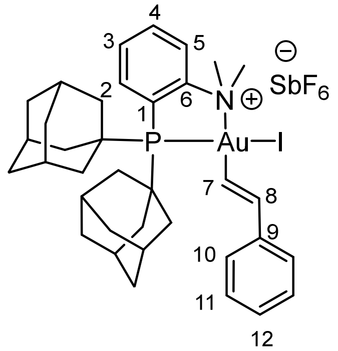

Complex **5a** was prepared using *trans*-styryl iodide and obtained instantaneous in 95% yield. **^{1}H NMR (500 MHz, CD_2Cl_2): δ** 8.02 (ddd, J_{HH} = 7.2 Hz, J_{HH} = 1.5 Hz, 1H, H_2), 7.98 – 7.93 (m, 2H, H_4 & H_3), 7.85 – 7.74 (m, 1H, H_5), 7.49 – 7.32 (m, 5H, H_{10} & H_{11} & H_{12}), 6.96 (d, J_{HH} = 14.3 Hz, 1H, H_8), 6.50 (dd, J_{HH} = 14.5 Hz, J_{HP} = 6.9 Hz, 1H, H_7), 3.51 (s, 6H, N(CH_3)$_2$), 2.53 – 2.29 (m, 6H, H_{Ad}), 2.29 – 2.07 (m, 12H, H_{Ad}), 1.79 (m, 12H, H_{Ad}). **^{31}P{^{1}H} NMR (162 MHz, CD_2Cl_2): δ** 75.0. **^{13}C NMR (125 MHz, CD_2Cl_2): δ** 158.4 (d, J_{PC} = 6.9 Hz, C_6), 140.7 (s, C_8), 136.7 (d, J_{PC} = 2.2 Hz, C_2), 136.5 (s, C_9), 135.6 (d, J_{PC} = 7.5 Hz, C_4), 130.5 (d, J_{PC} = 7.5 Hz, C_5), 129.0 (s, C_{11}), 128.6 (s, C_{12}), 126.3 (s, C_{10}), 125.1 (d, J_{PC} = 7.6 Hz, C_3), 118.6 (d, J_{PC} = 45.2 Hz, C_1), 113.2 (s, C_7), 53.4 (s, $N(CH_3)_2$), 47.6 (d, J_{PC} = 14.6 Hz, C_{qAd}), 40.4 (s, CH_{2Ad}), 35.3 (s, CH_{2Ad}), 28.5 (d, J_{PC} = 9.8 Hz, CH_{Ad}). **HRMS (ESI$^+$)**: calculated for [M^+] = $C_{36}H_{47}NPClAu^+$: 756.2800. Found: 756.2807.

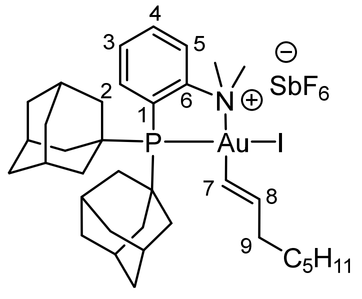

Complex **5b** was prepared using *trans*-1-Iodo-1-octene and obtained instantaneous in 99% yield. **^{1}H NMR (400 MHz, CD_2Cl_2): δ** 7.95 – 7.75 (m, 3H, H_2 & H_4 & H_5), 7.71 – 7.59 (m, 1H, H_3), 5.99 – 5.77 (m, 1H, H_8), 5.43 (ddt, J_{HH} = 13.6 Hz, J_{HP} = 7.5 Hz, J_{HH} = 1.4 Hz, 1H, H_7), 3.31 (s, 6H, N(CH_3)$_2$), 2.44 – 1.82 (m, 20H, H_{Ad} & H_9), 1.82 – 1.54 (m, 12H, H_{Ad}), 1.54 – 1.09 (m, 8H, H_{alk}), 0.95 – 0.67 (m, 3H, H_{alk}). **^{31}P{^{1}H} NMR (162 MHz, CD_2Cl_2): δ** 72.2 (s). **^{13}C NMR (125 MHz, CD_2Cl_2): δ** 158.3 (d, J_{PC} = 7.0 Hz, C_6), 142.8 (s, C_8), 136.5 (d, J_{PC} = 2.4 Hz, C_2), 135.7 (d, J_{PC} = 1.9 Hz, C_4), 130.3 (d, J_{PC} = 7.5 Hz, C_3), 125.1 (d, J_{PC} = 7.5 Hz, C_5), 118.8 (d, J_{PC} = 45.2 Hz, C_1), 109.8 (d, J_{PC} = 1.7 Hz, C_7), 53.1 (s, $N(CH_3)_2$), 47.3 (d, J_{PC} = 15.1 Hz, C_{qAd}), 40.4 (s, CH_{2Ad}), 36.9 (s, C_9), 35.4 (s, CH_{2Ad}), 31.6 (s, CH_{2alk}), 29.0 (s, CH_{2alk}), 28.8 (s, CH_{2alk}), 28.5 (d, J_{PC} = 9.8 Hz, CH_{Ad}), 22.6 (s, CH_{2alk}), 13.9 (s, CH_{3alk}). **HRMS (ESI$^+$)**: calculated for [M^+] = $C_{36}H_{55}NPClAu^+$: 764.3426. Found: 764.3419. **Mp (°C):** 124.1.

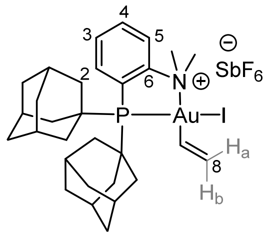

Complex **5c** was prepared using vinyl iodide and obtained in 90% yield in 3 hours. **^{1}H NMR (500 MHz, CD_2Cl_2): δ** 8.00 (dddd, J_{HH} = 8.6 Hz, J_{HH} = 7.0 Hz, J_{HH} = 1.6 Hz, 1H, H_2), 7.97 – 7.91 (m, 2H, H_4 & H_3), 7.78 (dddd, J_{HH} = 8.2 Hz, J_{HH} = 7.1 Hz, J_{HP} = 2.3 Hz, J_{HH} = 1.2 Hz, 1H, H_5), 6.24 (ddd, J_{HH} = 7.5 Hz, J_{HH} = 3.8 Hz, 1H, H_{8b}), 6.18 (dt, J_{HH} = 14.9 Hz, J_{HH} = 7.0 Hz, 1H, H_7), 5.73 (ddd, J_{HH} = 14.9 Hz, J_{HH} = 3.6 Hz, J_{HP} = 1.5 Hz, 1H, H_{8a}), 3.45 (s, 6H, N(CH_3)$_2$), 2.39 – 2.31 (m, 6H, H_{Ad}), 2.24 – 2.15 (m, 12H, H_{Ad}), 1.83 – 1.75 (m, 12H, H_{Ad}). **^{31}P{^{1}H} NMR (162 MHz, CD_2Cl_2): δ** 73.8. **^{13}C NMR (125 MHz, CD_2Cl_2): δ** 158.3 (d, J_{PC} = 7.0 Hz, C_6), 136.6 (d, J_{PC} = 2.4 Hz, C_2), 135.7 (d, J_{PC} = 1.8 Hz, C_4), 130.4 (d, J_{PC} = 7.5 Hz, C_5), 127.7 (s, C_8), 125.1 (d, J_{PC} = 7.6 Hz, C_3), 121.2 (s, C_7), 118.7 (d, J_{PC} = 45.1 Hz, C_1), 53.2 (s, $N(CH_3)_2$), 47.4 (d, J_{PC} = 14.8 Hz, C_{qAd}), 40.4 (s, CH_{2Ad}), 35.4 (d, J_{PC} = 1.9 Hz, CH_{2Ad}), 28.5 (d, J_{PC} = 9.8 Hz, CH_{Ad}). **HRMS (ESI$^+$)**: calculated for [M^+] = $C_{30}H_{43}NPClAu^+$: 680.2510. Found: 680.2504. **Mp (°C):** 124.2.

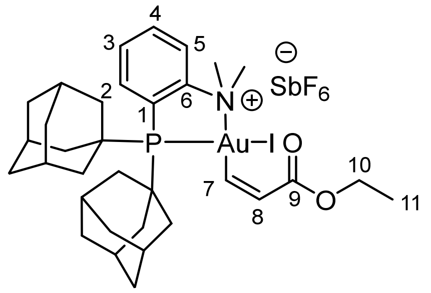

Complex **5d** was prepared using Ethyl *cis*-3-iodoacrylate and obtained instantaneous in 99% yield. **^{1}H NMR (500 MHz, CD_2Cl_2): δ** 8.02 (dddd, J_{HH} = 8.6 Hz, J_{HH} = 7.1 Hz, J_{HH} = 1.6 Hz, 1H, H_2), 7.99 – 7.92 (m, 2H, H_4 & H_5), 7.79 (dddd, J_{HH} = 8.1 Hz, J_{HH} = 7.1 Hz, J_{HP} = 2.4 Hz, J_{HH} = 1.1 Hz, 1H, H_3), 6.99 (dd, J_{HH} = 7.7 Hz, J_{HP} = 5.3 Hz, 1H, H_7), 6.79 (dd, J_{HH} = 7.7 Hz, J_{HP} =2.3 Hz, 1H, H_8), 4.29 (q, J_{HH} = 7.1 Hz, 2H, H_{10}), 3.55 (s, 6H, N(CH_3)$_2$), 2.53 – 1.60 (m, 30H, H_{Ad}), 1.35 (t, J_{HH} = 7.1 Hz, 3H, H_{11}). **^{31}P{^{1}H} NMR (162 MHz, CD_2Cl_2): δ** 79.9 (s). **^{13}C NMR (125 MHz, CD_2Cl_2): δ** 167.34 (s, C_9), 158.53 (d, J_{PC} = 6.9 Hz, C_6), 136.65 (s, C_2), 135.28 (d, J_{PC} = 1.7 Hz, C_4), 132.73 (s, C_7), 130.80 (s, C_8), 130.58 (d, J_{PC} = 7.5 Hz, C_3), 124.94 (d, J_{PC} = 7.7 Hz, C_5), 118.94 (d, J_{PC} = 45.1 Hz, C_1), 61.62 (s, C_{10}), 53.2 (s, N(CH_3)$_2$), 35.30 (s, CH_{2Ad}), 28.46 (d, J_{PC} = 9.9 Hz, CH_{Ad}), 13.97 (s, C_{11}). C_{qAd} not observed. **HRMS (ESI$^+$)**: calculated for [M^+] = $C_{33}H_{47}NO_2PClAu^+$: 752.2698. Found: 752.2696. **Mp (°C):** 124.3.

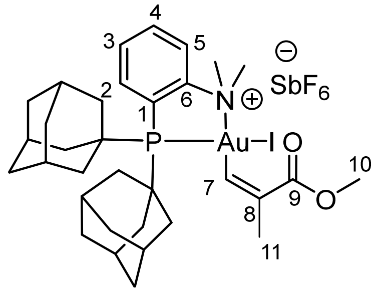

Complex **5e** was prepared using methyl (*Z*)-3-iodo-2-methylacrylate and obtained instantaneous in 99% yield. **^{1}H NMR (400 MHz, CD_2Cl_2): δ** 7.99-7.88 (m, 3H, H_2, H_4 & H_5), 7.74 (dddd, J_{HH} = 8.1 Hz, J_{HH} = 7.1 Hz, J_{HP} = 2.4 Hz, J_{HH} = 1.1 Hz, 1H, H_3), 6.41 (dq, J_{HP} = 5.9, J_{HH} = 1.5 Hz, 1H, H_7), 3.80 (s, 1H, H_{10}), 3.49 (s, 6H, N(CH_3)$_2$), 2.39 - 2.34 (m, 6H, H_{Ad}), 2.23 (dd, J_{HH} = 1.5, J_{HP} = 0.8 Hz, 3H, H_{11}) 2.11 – 1.99 (m, 12H, H_{Ad}), 1.81 – 1.69 (m, 18H, H_{Ad}). **^{31}P{^{1}H} NMR (162 MHz, CD_2Cl_2): δ** 77.1 (s). **^{13}C NMR (100 MHz, CD_2Cl_2): δ** 169.3 (s, C_9), 159.2 (d, J_{PC} = 7.0 Hz, C_6), 138.5 (s, C_2), 137.1 (d, J_{PC} = 2.5 Hz, C_4), 135.9 (d, J_{PC} = 2.0 Hz, C_8), 131.1 (d, J_{PC} = 7.6 Hz, C_3), 125.5 (d, J_{PC} = 7.6 Hz, C_5), 124.7 (s, C_7), 119.7 (d, J_{PC} = 45.0 Hz, C_1), 53.2 (s, N(CH_3)$_2$), 35.90 (d, J_{PC} = 2.1 Hz, CH_{2Ad}), 29.1 (d, J_{PC} = 9.8 Hz, CH_{Ad}), 21.9 (s, C_{10}), 14.4 (s, C_{11}). C_{qAd} not observed. **HRMS (ESI$^+$)**: calculated for [M^+] = $C_{33}H_{47}NO_2PClAu^+$: 752.2698. Found: 752.2698. **Mp (°C):** 124.1.

2.2.1.3 Reaction of (IPr)AuCl with $H_{13}C_6$–CH=CH–I

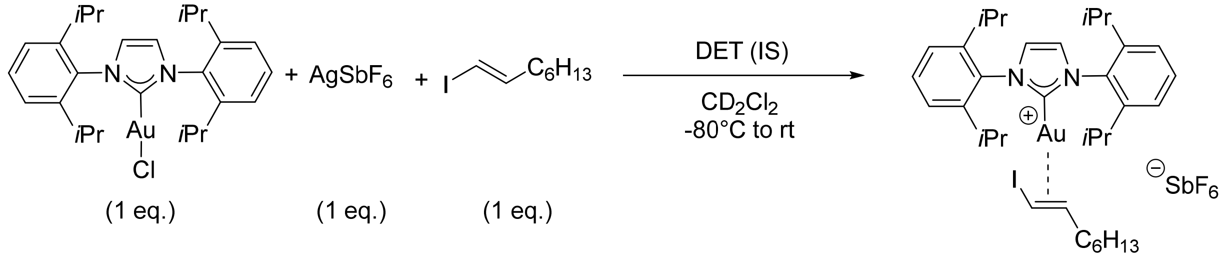

In a glovebox, a screw-cap NMR tube was charged with silver hexafluoroantimonate (8.0 mg, 0.023 mmol) in CD_2Cl_2 (0.3 mL). (IPr)AuCl complex (14.2 mg, 0.023 mmol, 1 eq.) was transferred into a small glass vial and dissolved in CD_2Cl_2 (0.3 mL). The vinyl iodide (5.5 mg, 0.023 mmol, 1 eq.) was added to the solution of (IPr)AuCl complex. The prepared solution was loaded into a plastic syringe equipped with stainless steel needle. The syringe was closed by blocking the needle with a septum. Outside the glovebox, the NMR tube was cooled down to -80°C (Ethanol/N_2 cold bath). At this temperature, the solution of (IPr)AuCl complex and vinyl iodide was added. The tube was gently shaken and allowed to warm to RT. The reaction was repeated two times. The formation of π-complex was quantitative according to ^{1}H NMR and HSQC ^{1}H-^{13}C NMR experiments.

^{1}H NMR (400 MHz, CD_2Cl_2): δ 7.59 (dd, *J* = 8.2, 7.5 Hz, 2H), 7.49 (s, 2H), 7.38 (d, *J* = 7.8 Hz, 4H), 6.45 (dt, *J* = 14.4, 6.3 Hz, 1H), 5.74 (dt, J = 14.4, 1.1 Hz, 1H), 2.47 (hept, *J* = 6.9 Hz, 4H), 1.92-1.87 (m, 2H), 1.28 (dd, *J* = 9.2, 6.9 Hz, 24H), 1.28-1.25 (m, 2H), 1.17-1.01 (m, 6H), 0.90 (t, *J* = 7.3 Hz, 3H).

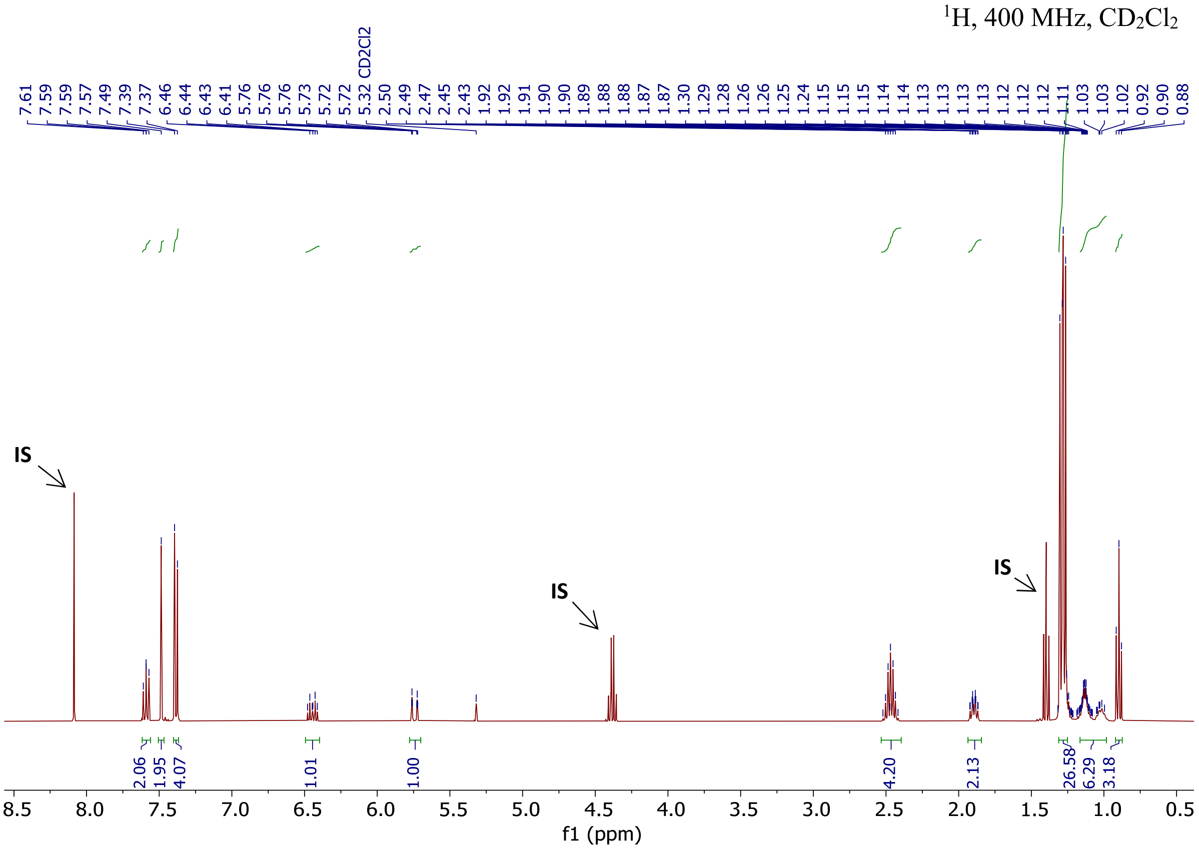

^{1}H, 400 MHz, CD_2Cl_2
IS
IS
IS
2.06
1.95
4.07
1.01
1.00
4.20
2.13
26.58
6.29
3.18
f1 (ppm)

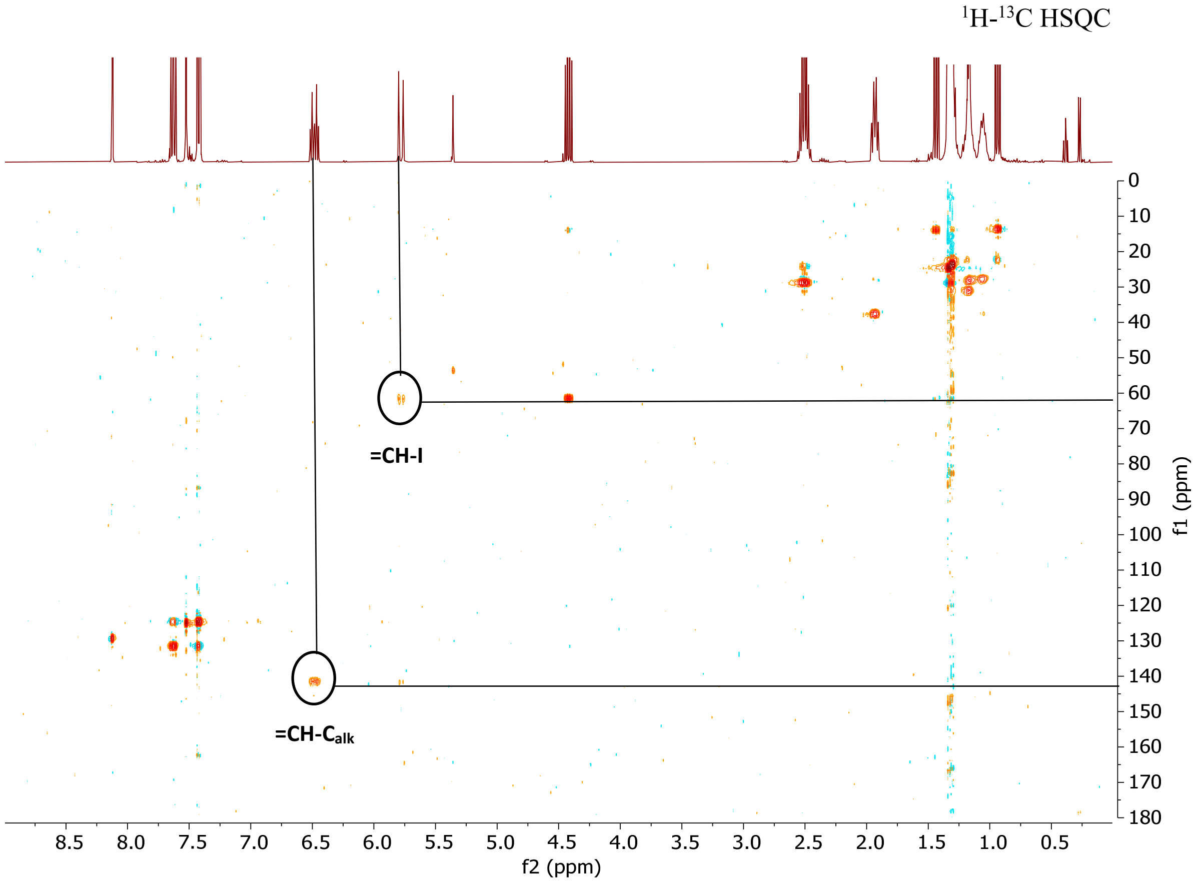

^{1}H-^{13}C HSQC
=CH-I
=CH-C_{alk}
f2 (ppm)
f1 (ppm)

2.2.2 Competition experiments

2.2.2.1 Competition reaction between (iodoethynyl)benzene and iodobenzene

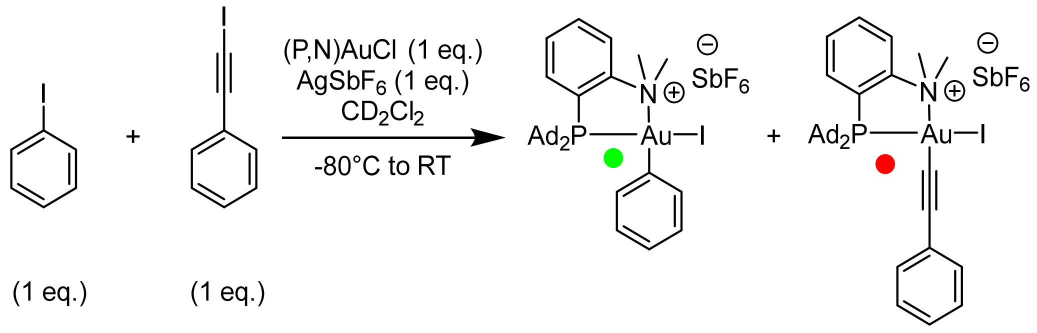

In a glovebox, a screw-cap NMR tube was charged with $AgSbF_6$ (8.0 mg, 0.023 mmol) in CD_2Cl_2 (0.3 mL). (P,N)AuCl complex (15 mg, 0.023 mmol) was transferred into a glass vial and dissolved in CD_2Cl_2 (0.3 mL). Iodobenzene (2.6 µL, 0.023 mmol) and (iodoethynyl)benzene (5.2 mg, 0.023 mmol) were added to the solution of (P,N)AuCl complex. The prepared solution was loaded into a plastic syringe equipped with stainless steel needle. The syringe was closed by blocking the needle with a septum. Outside the glovebox, the NMR tube was cooled down to -80°C (Ethanol/N_2 cold bath). At this T, the solution of (P,N)AuCl complex and the two iodides was added. The tube was gently shaken and allowed to warm to RT. The relative ratio of oxidative addition products was determined by ^{31}P and ^{1}H NMR spectroscopy. We observed 94% of the complex **3a** and 6% of the complex **6**.

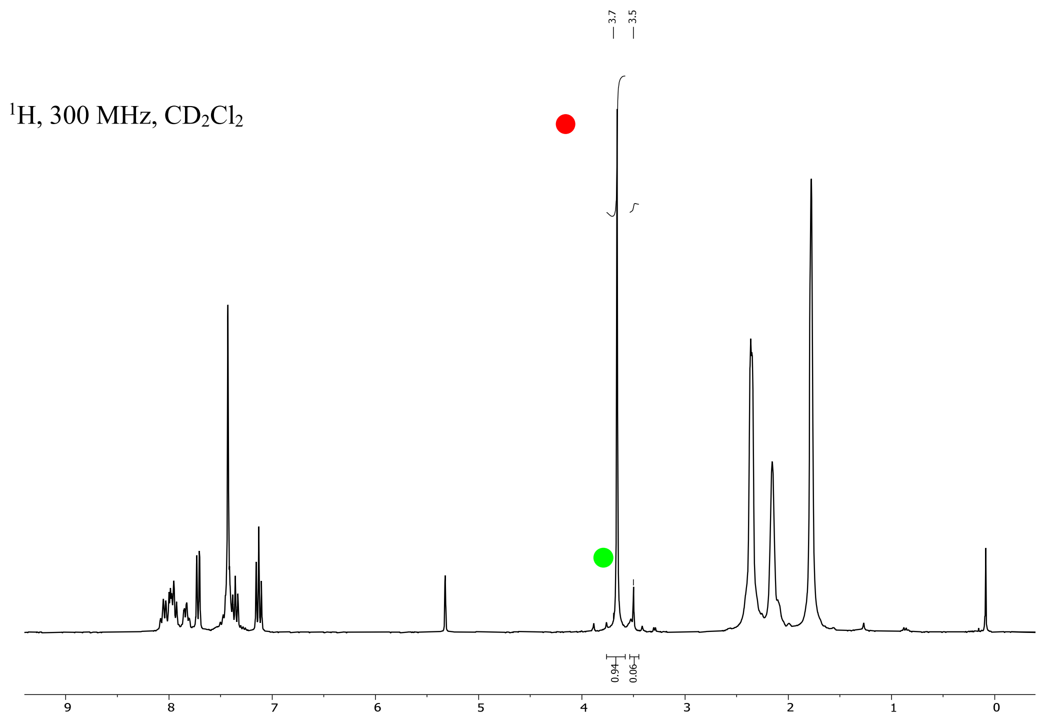

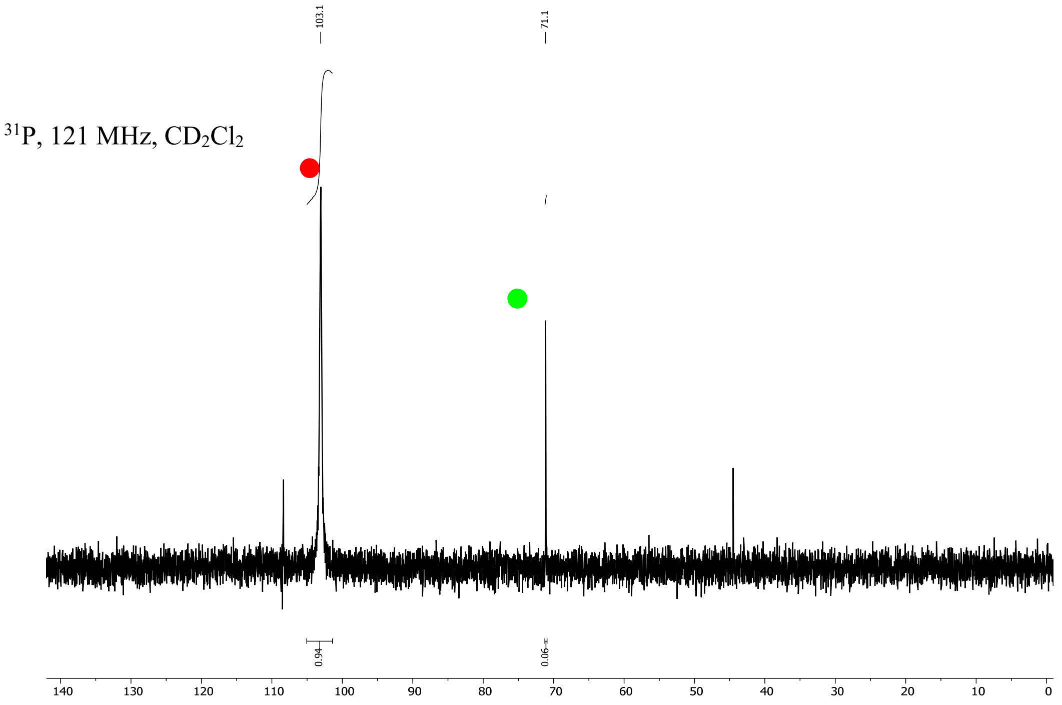

2.2.2.2 Competition reaction between trans-styryl iodide and iodobenzene

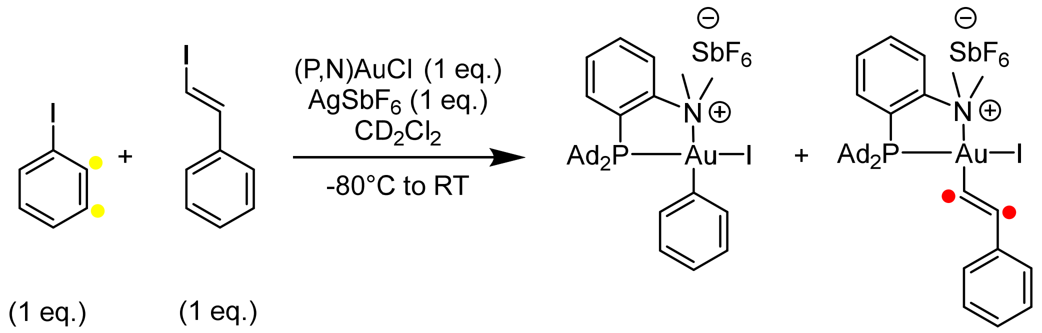

In a glovebox, a screw-cap NMR tube was charged with $AgSbF_6$ (8.0 mg, 0.023 mmol) in CD_2Cl_2 (0.3 mL). (P,N)AuCl complex (15 mg, 0.023 mmol) was transferred into a glass vial and dissolved in CD_2Cl_2 (0.3 mL). Iodobenzene (2.6 µL, 0.023 mmol) and *trans*-styryl iodide (5.3 mg, 0.023 mmol) were added to the solution of (P,N)AuCl complex. The prepared solution was loaded into a plastic syringe equipped with stainless steel needle. The syringe was closed by blocking the needle with a septum. Outside the glovebox, the NMR tube was cooled down to -80°C (Ethanol/N_2 cold bath). At this temperature, the solution of (P,N)AuCl complex and the two iodides was added. The tube was gently shaken and allowed to warm to RT. The relative ratio of oxidative addition products was determined by ^{31}P and ^{1}H NMR spectroscopy. Only the complex **5a** was observed and iodobenzene.

^{1}H, 300 MHz, CD_2Cl_2

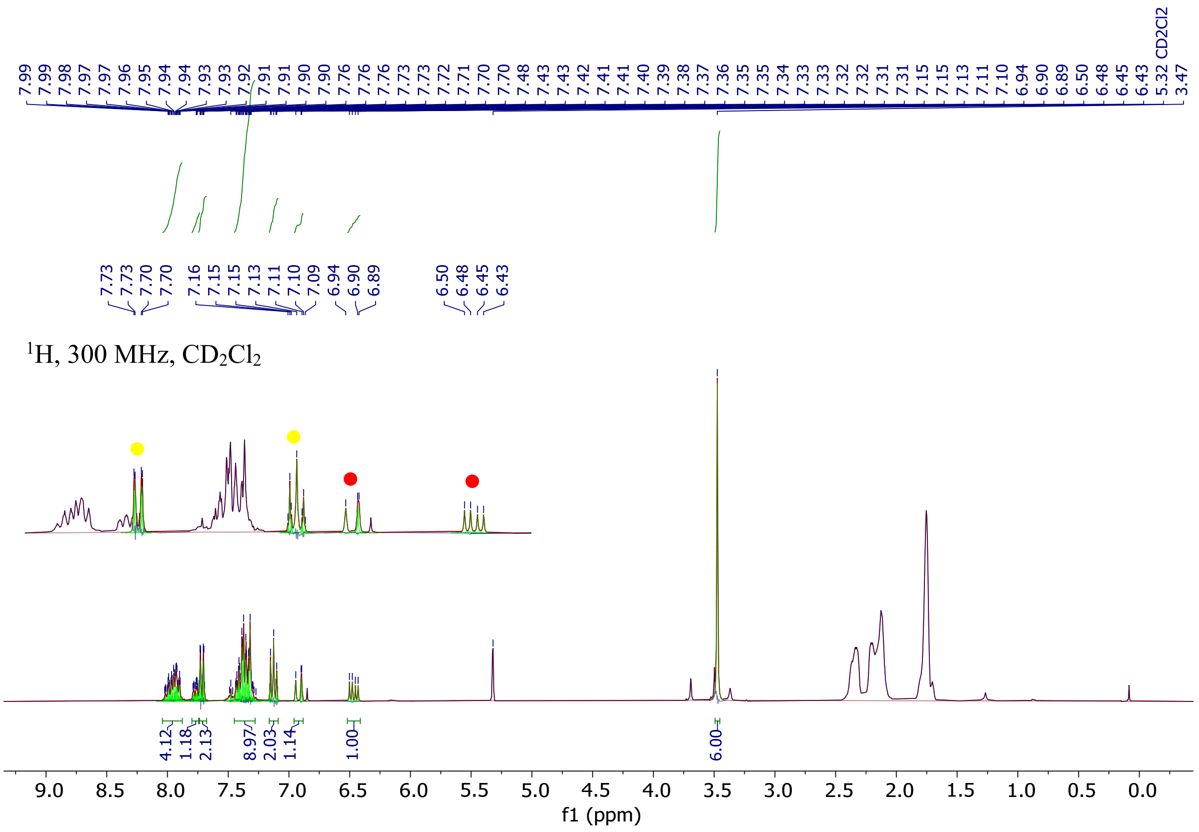

^{31}P, 121 MHz, CD_2Cl_2

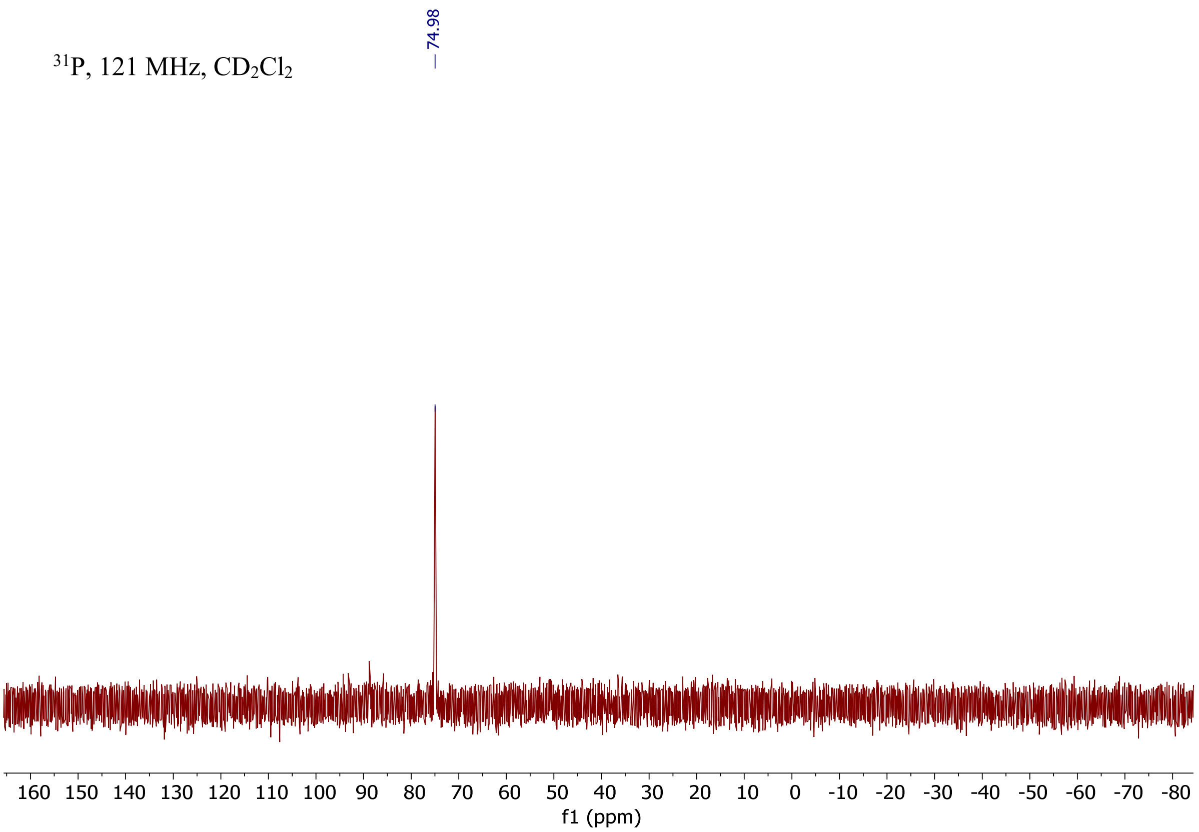

2.2.2.3 Competition reaction between trans-styryl iodide and (iodoethynyl)benzene

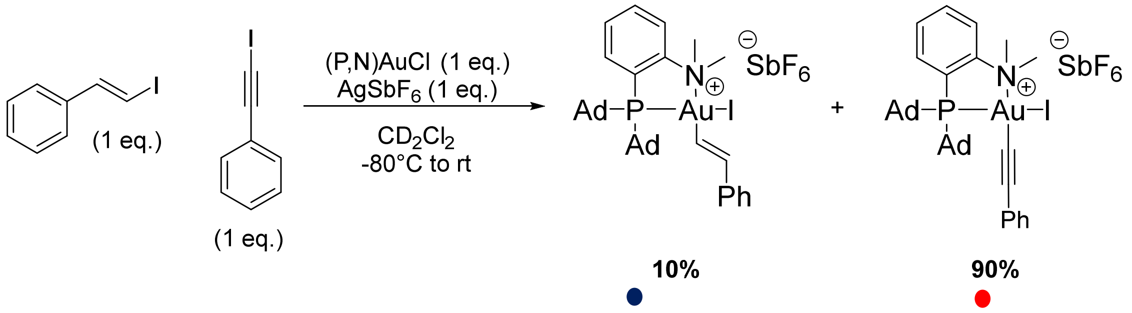

In a glovebox, a screw-cap NMR tube was charged with $AgSbF_6$ (16.0 mg, 0.046 mmol) in CD_2Cl_2 (0.6 mL). (P,N)AuCl complex (30 mg, 0.046 mmol) was transferred into a glass vial and dissolved in CD_2Cl_2 (0.6 mL). (iodoethynyl)benzene (10.4 mg, 0.046 mmol) and *trans*-styryl iodide (10.6 mg, 0.046 mmol) were added to the solution of (P,N)AuCl complex. The prepared solution was loaded into a plastic syringe equipped with stainless steel needle. The syringe was closed by blocking the needle with a septum. Outside the glovebox, the NMR tube was cooled down to -80°C (Ethanol/N_2 cold bath). At this temperature, the solution of (P,N)AuCl complex and the two iodides was added. The tube was gently shaken and allowed to warm to RT. The relative ratio of oxidative addition products was determined by ^{31}P and ^{1}H NMR spectroscopy.

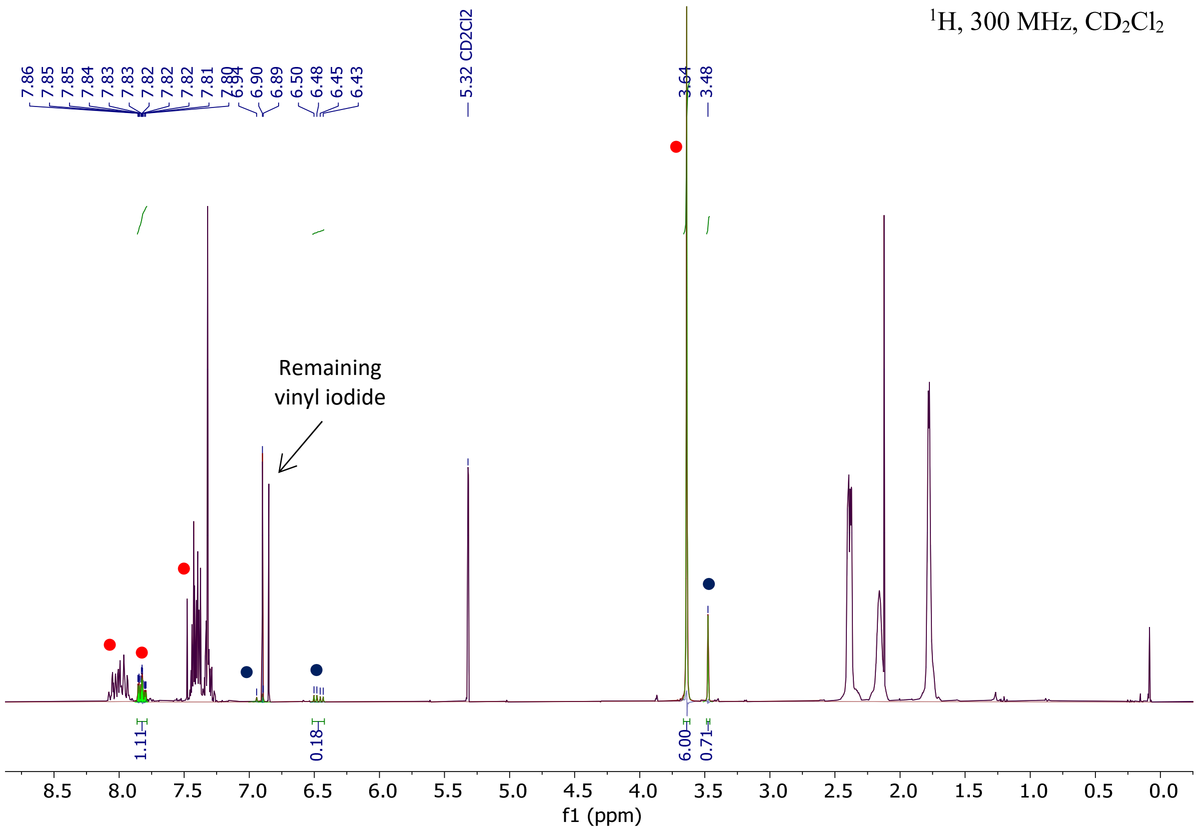

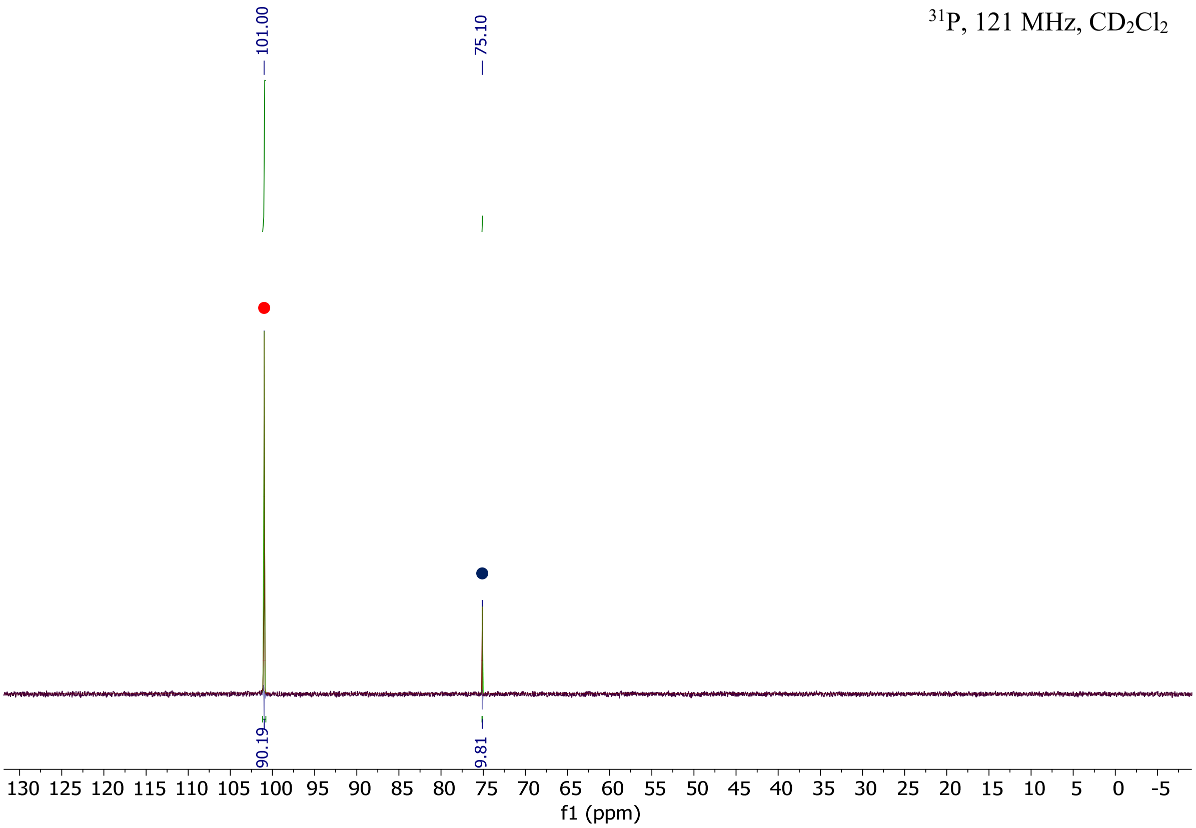

2.2.3 Oxidative addition of (iodoethynyl)benzene in the presence of 4-penten-1-ol

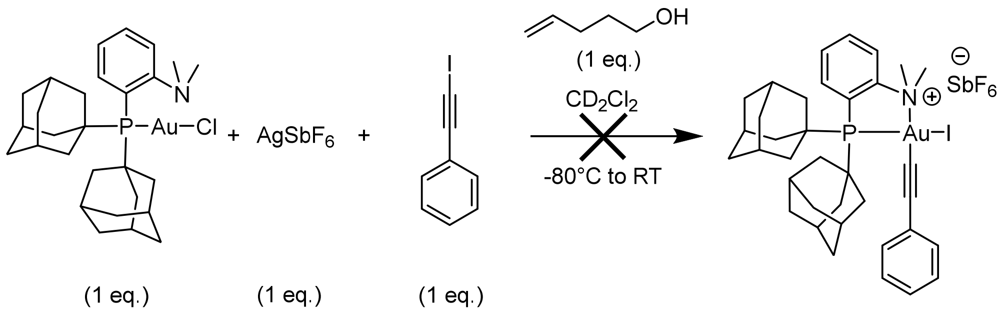

In a glovebox, a screw-cap NMR tube was charged with silver hexafluoroantimonate (8.0 mg, 0.023 mmol) in CD_2Cl_2 (0.3 mL). (MeDalphos)AuCl complex **1** (15 mg, 0.023 mmol, 1 eq.) was transferred into a small glass vial and dissolved in CD_2Cl_2 (0.3 mL). (iodoethynyl)benzene (0.023 mmol, 1 eq.) and 4-penten-1-ol (0.023 mmol, 1 eq.) were added to the solution of **1**. The prepared solution was loaded into a plastic syringe equipped with stainless steel needle. The syringe was closed by blocking the needle with a septum. Outside the glovebox, the NMR tube was cooled down to -80°C (Ethanol/N_2 cold bath). At this temperature, the solution of complex **1**, alkynyl iodide and 4-penten-1-ol was added. The tube was gently shaken and allowed to warm to RT. The reaction was monitored by ^{31}P NMR spectroscopy.

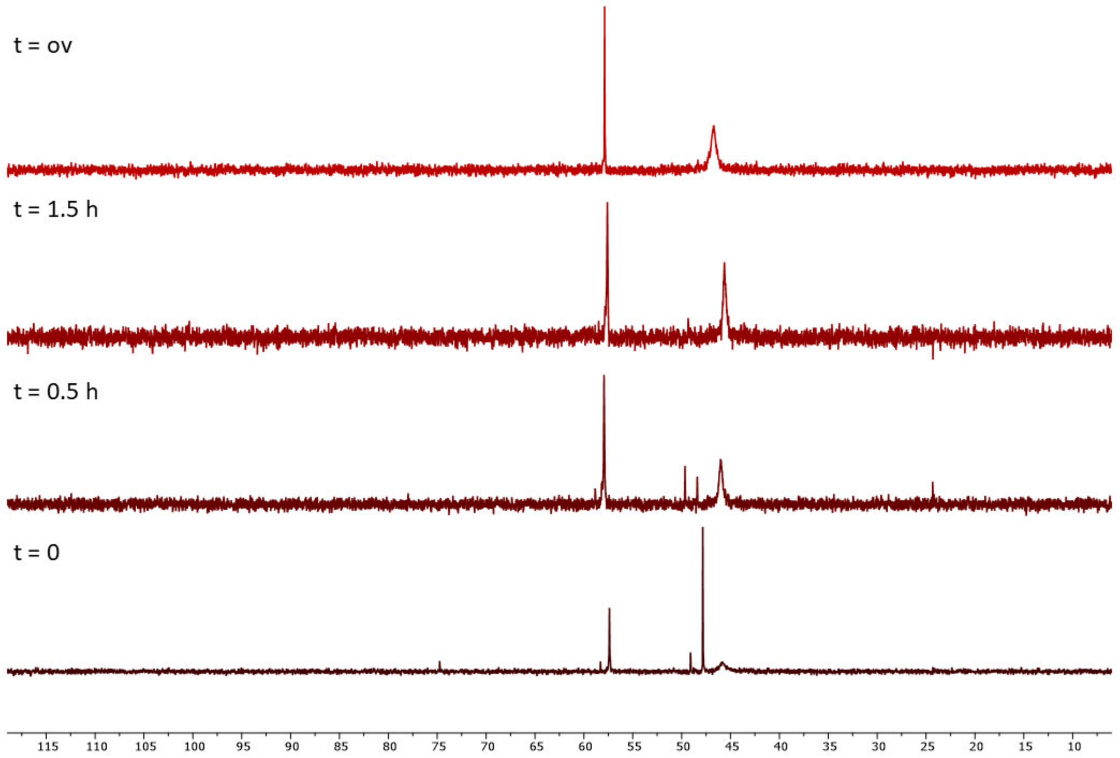

2.3 Gold-catalyzed coupling of vinyl iodide and 4-penten-1-ol

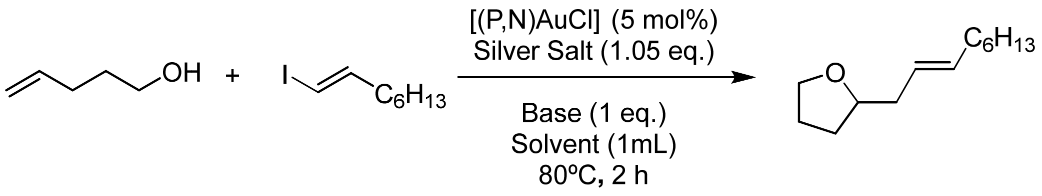

(procedure for optimization of the reaction conditions)

In a glovebox, a flame dried Schlenk equipped with a magnetic stirrer bar was charged with the silver salt (0.42 mmol) and base (0.40 mmol) in 0.4 mL of solvent. [(P,N)AuCl] (13 mg, 0.02 mmol), vinyl iodide (0.4 mmol), 4-penten-1-ol (0.4 mmol), and internal standard were transferred into a small glass vial dissolved in 0.6 mL of solvent. This solution was loaded into a plastic syringe equipped with stainless steel needle. The syringe was closed by blocking the needle with a septum. Outside the glovebox, the Schlenk was cooled down to –10 °C (acetone/N_2 cold bath). At this temperature, the solution of gold complex was added. The reaction mixture was then stirred at 80°C during 2 hours. The yield in heterovinylation product **7a** was determined by ^{1}H NMR using dimethyl terephthalate as internal standard.

2.4 Gold-catalyzed oxy-vinylation of alkenes

2.4.1 Synthesis of alkenol substrates

2,2-diphenyl-4-penten-1-ol[8], 2-benzylpent-4-en-1-ol[9], 1-but-3-enylcyclohexanol[10], 4-methyl-4-penten-1-ol[11], and heptenol[12] were prepared according to reported procedures.

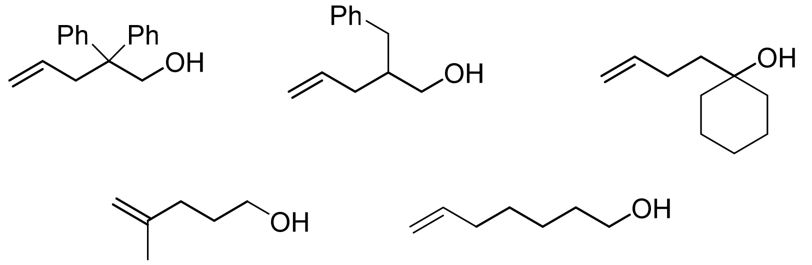

2.4.2 General procedure for the gold-catalyzed oxy-vinylation of alkenes

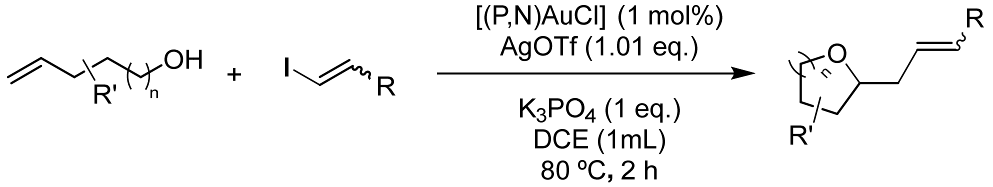

In a glovebox, a flame dried Schlenk equipped with a magnetic stirrer bar was charged with the silver trifluoromethanesulfonate (103 mg, 0.404 mmol) and potassium phosphate tribasic (85 mg, 0.40 mmol) in 0.4 mL of solvent. [(P,N)AuCl] (2.6 mg, 0.004 mmol), the vinyl iodide (0.4 mmol), the functionalized alkenol (0.4 mmol), and internal standard were transferred into a small glass vial dissolved in 0.6 mL of solvent. This solution was loaded into a plastic syringe equipped with stainless steel needle. The syringe was closed by blocking the needle with a septum. Outside the glovebox, the Schlenk was cooled down to –10 °C (acetone/N_2 cold bath). At this temperature, the solution of gold complex was added. The reaction mixture was then stirred at 80°C during 2 hours. The yield in heterovinylation product was determined by ^{1}H NMR using dimethyl terephthalate as internal standard. To isolate the product, silver salts were removed by filtration over celite and the sample was purified by column chromatography.

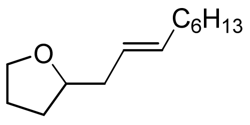

(*E*)-2-(non-2-en-1-yl)tetrahydrofuran (**7a**): The pure product was obtained after column chromatography (pentane/ethyl acetate: 96/4, R_f: 0.6) as a yellow oil (65 mg, 83%). **^{1}H NMR (300 MHz, $CDCl_3$): δ** 5.54-5.34 (m, 2H), 3.90-3.78 (m, 2H), 3.74-3.67 (m, 1H), 2.33-2.24 (m, 1H), 2.20-2.11 (m, 1H), 2.02-1.81 (m, 5H), 1.54-1.43 (m, 1H), 1.34-1.22 (m, 8H), 0.89-0.85 (m, 3H). **^{13}C NMR (75 MHz, $CDCl_3$): δ** 133.2, 126.3, 79.3, 69.0, 39.0, 32.8, 31.9, 30.9, 29.6, 29.0, 25.8, 22.8, 14.2. **EI-MS:** Calculated for $C_{13}H_{24}O_2$: 196.1827, Found: 196.2977.

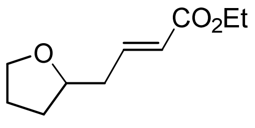

Ethyl (*E*)-4-(tetrahydrofuran-2-yl)but-2-enoate (**7b**): The pure product was obtained after column chromatography (pentane/diethyl ether: 9/1, R_f: 0.2) as a colorless oil (52 mg, 70%). **^{1}H NMR (300 MHz, $CDCl_3$): δ** 6.94 (dt, *J* = 15.7, 7.2 Hz, 1H), 5.88 (dt, *J* = 15.7, 1.5 Hz, 1H), 4.16 (q, *J* = 7.1 Hz, 2H), 3.99-3.83(m, 2H), 3.75-3.68 (m, 1H), 2.46-2.37 (m, 2H), 2.05-1.82 (m, 3H), 1.56-1.44 (m, 1H), 1.26 (t, J = 7.1 Hz, 3H). **^{13}C NMR (75 MHz, $CDCl_3$): δ** 166.5, 145.3, 123.4, 77.6, 68.1, 60.3, 38.4, 31.1, 25.7, 14.4. **EI-MS:** Calculated for $C_{10}H_{16}O_3$: 184.1099, Found: 184.1156.

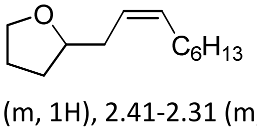

(*Z*)-2-(non-2-en-1-yl)tetrahydrofuran (**7c**): The pure product was obtained after column chromatography (pentane/ethyl acetate: 98/2, R_f: 0.5) as a yellow oil (51 mg, 65%). **^{1}H NMR (300 MHz, $CDCl_3$): δ** 5.52-5.33 (m, 2H), 3.91-3.79 (m, 2H), 3.75-3.68 (m, 1H), 2.41-2.31 (m, 1H), 2.25-2.16 (m, 1H), 2.07-1.98 (m, 2H), 1.96-1.79 (m, 3H), 1.54-1.43 (m, 1H), 1.36-1.25 (m, 8H), 0.90-0.85 (m, 3H). **^{13}C NMR (75 MHz, $CDCl_3$): δ** 132.2, 125.4, 79.2, 68.0, 33.5, 31.9, 31.0, 29.7, 29.1, 27.6, 25.8, 22.8, 14.2. **EI-MS:** Calculated for $C_{13}H_{24}O_2$: 196.1827, Found: 196.0474.

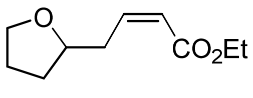

Ethyl (*Z*)-4-(tetrahydrofuran-2-yl)but-2-enoate (**7d**): The pure product was obtained after column chromatography (pentane/diethyl ether: 9/1, R_f: 0.3) as a colorless oil (58 mg, 78%). **^{1}H NMR (300 MHz, $CDCl_3$): δ** 6.32 (dt, *J* = 11.6, 7.3 Hz, 1H), 5.84 (dt, *J* = 11.6, 1.8 Hz, 1H), 4.15 (q, *J* = 7.1 Hz, 2H), 4.00-3.91(m, 1H), 3.90-3.83 (m, 1H), 3.76-3.69 (m, 1H), 2.99-2.79 (m, 2H), 2.04-1.82 (m, 3H), 1.59-1.48 (m, 1H), 1.27 (t, J = 7.1 Hz, 3H). **^{13}C NMR (75 MHz, $CDCl_3$): δ** 166.5, 146.5, 121.2, 78.4, 68.1, 60.0, 35.0, 31.1, 25.8, 14.4. **EI-MS:** Calculated for $C_{10}H_{16}O_3$: 184.1099, Found: 184.0023.

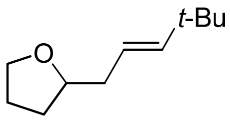

(*E*)-2-(4,4-dimethylpent-2-en-1-yl)tetrahydrofuran (**7e**): The pure product was obtained after column chromatography (pentane/ethyl acetate: 96/4, R_f: 0.6) as a yellow oil (49 mg, 73%). **^{1}H NMR (300 MHz, $CDCl_3$):** 5.52 (dt, *J* = 15.6, 1.2 Hz, 1H), 5.32 (dt, *J* = 15.6, 6.9 Hz, 1H), 3.90-3.81 (m, 2H), 3.79-3.68 (m, 1H), 2.34-2.25 (m, 1H), 2.18-2.09 (m, 1H), 1.98-1.80 (m, 3H), 1.55-1.46 (m, 1H), 0.99 (s, 9H). **δ ^{13}C NMR (75 MHz, $CDCl_3$): δ** 144.1, 120.8, 79.4, 68.1, 39.1, 33.1, 30.8, 29.9 (3C), 25.9. **EI-MS:** Calculated for $C_{11}H_{20}O$: 168.1514 Found: 168.2008.

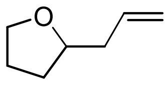

2-allyltetrahydrofuran (**7f**): The pure product was obtained after column chromatography (pentane/ethyl acetate: 96/4, R_f: 0.6) as a colorless oil (28 mg, 62%). Analytical data are consistent with those previously reported[13] **^{1}H NMR (300 MHz, $CDCl_3$): δ** 5.85 (ddt, J = 17.2, 10.2, 7.0 Hz, 1H), 5.16-5.05 (m, 2H), 3.95-3.86 (m, 2H), 3.78-3.71 (m, 1H), 2.37-2.26 (m, 2H), 2.04-1.84 (m, 3H), 1.56-1.49 (m, 1H).

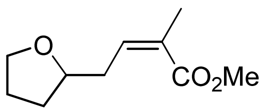

Ethyl (*Z*)-2-methyl-4-(tetrahydrofuran-2-yl)but-2-enoate (**7g**): The pure product was obtained after column chromatography (pentane/ethyl Acetate: 8/2, R_f: 0.6) as a yellow oil (64 mg, 87%). **^{1}H NMR (300 MHz, $CDCl_3$): δ** 6.04 (tq, *J* = 7.2, 1.5 Hz, 1H), 3.95-3.82 (m, 2H), 3.74-3.67 (m, 1H), 3.71 (s, 3H), 2.79-2.59 (m, 2H), 2.02-1.80 (m, 3H), 1.90 (q, *J* = 1.5 Hz, 3H), 1.56-1.44 (m, 1H). **^{13}C NMR (75 MHz, $CDCl_3$): δ** 168.4, 139.7, 128.4, 78.8, 68.0, 51.4, 35.7, 31.1, 25.8, 20.8. **EI-MS:** Calculated for $C_{10}H_{16}O_3$: 184.1099, Found: 184.1895.

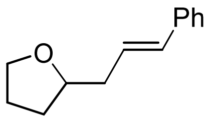

2-cinnamyltetrahydrofuran (**7h**): The pure product was obtained after column chromatography (pentane/ethyl acetate: 98/2, R_f: 0.5) as a colorless oil (59 mg, 75%). Analytical data are consistent with those previously reported[14] **^{1}H NMR (300 MHz, $CDCl_3$): δ** 7.38-7.26 (m, 4H), 7.22-7.17 (m, 1H), 6.46 (dt, *J* = 15.8, 1.4 Hz, 1H), 6.24 (dt, *J* = 15.9, 7.1 Hz, 1H), 4.01-3.87 (m, 2H), 3.79-3.72 (m, 1H), 2.51-2.41 (m, 2H), 2.04-1.84 (m, 3H), 1.61-1.54 (m, 1H).

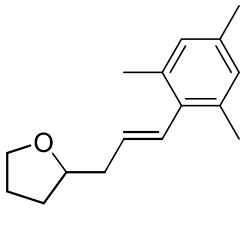

(*E*)-2-(3-mesitylallyl)tetrahydrofuran (**7i**): The pure product was obtained after column chromatography (pentane/ethyl acetate: 98/2, R_f: 0.3) as a yellow oil (69 mg, 75%). **^{1}H NMR (300 MHz, $CDCl_3$): δ** 6.87 (s, 2H), 6.39 (d, *J* = 16.1 Hz, 1H), 5.67 (dt, *J* = 16.2, 7.2 Hz, 1H), 4.03-3.89 (m, 2H), 3.81-3.74 (m, 1H), 2.64-2.55 (m, 1H), 2.49-2.37 (m, 1H), 2.28 (s, 9H), 2.08-1.83 (m, 3H), 1.69-1.58 (m, 1H). **^{13}C NMR (75 MHz, $CDCl_3$): δ** 135.9, 135.8, 134.6, 131.4, 129.8, 128.5 (3 Cq), 79.0, 68.1, 39.7, 30.8, 25.9, 21.0 (3 CH_3). **EI-MS:** Calculated for $C_{16}H_{22}O$: 230.1671, Found: 230.1373.

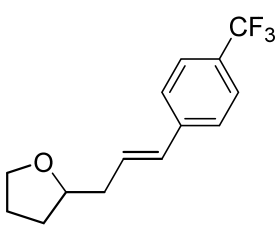

(*E*)-2-(3-(4-(trifluoromethyl)phenyl)allyl)tetrahydrofuran (**7j**): The pure product was obtained after column chromatography (pentane/ethyl acetate: 98/2, R_f: 0.5) as a yellow oil (80 mg, 78%). **^{1}H NMR (300 MHz, $CDCl_3$): δ** 7.53 (d, *J* = 8.2 Hz, 2H), 7.43 (d, *J* = 8.2 Hz, 2H), 6.49 (d, *J* = 16.0 Hz, 1H), 6.36 (dt, *J* = 15.9, 6.8 Hz, 1H), 4.02-3.87 (m, 2H), 3.79-3.72 (m, 1H), 2.56-2.39 (m, 2H), 2.06-1.83 (m, 3H), 1.62-1.50 (m, 1H). **^{13}C NMR (75 MHz, $CDCl_3$): δ** 141.2 (q, *J* = 1.5 Hz), 130.8, 130.0, 128.9 (q, *J* = 32.4 Hz), 126.3 (2 C_{ar}), 125.5 (q, *J* = 3.8 Hz, 2 C_{ar}), 124.4 (q, *J* = 271.7Hz), 78.6, 68.1, 39.3, 31.1, 25.8. **^{19}F NMR (282 MHz, $CDCl_3$): δ** -62.4. **EI-MS:** Calculated for $C_{14}H_{15}F_3O$: 256.1075, Found: 255.9676.

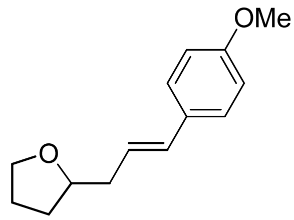

(E)-2-(3-(4-methoxyphenyl)allyl)tetrahydrofuran (**7k**): The pure product was obtained after column chromatography (pentane/ethyl acetate: 9/1, R_f: 0.5) as a yellow oil (45 mg, 52%). **^{1}H NMR (300 MHz, $CDCl_3$): δ** 7.29 (d, *J* = 8.4 Hz, 2H), 6.83 (d, *J* = 8.8 Hz, 2H), 6.40 (dt, *J* = 15.8, 1.5 Hz, 1H), 6.09 (dt, *J* = 15.8, 7.1 Hz, 1H), 3.99-3.87 (m, 2H), 3.80 (s, 3H), 3.80-3.71 (m, 1H), 2.51-2.35 (m, 2H), 2.03-1.83 (m, 3H), 1.62-1.53 (m, 1H). **^{13}C NMR (75 MHz, $CDCl_3$): δ** 158.9 (Cq), 131.4, 130.6 (Cq), 127.3 (2 C_{ar}), 124.7, 114.0 (2 C_{ar}), 79.1, 68.1, 55.4, 39.4, 31.0, 25.9. **EI-MS:** Calculated for $C_{14}H_{18}O_2$: 218.1307, Found: 218.1308.

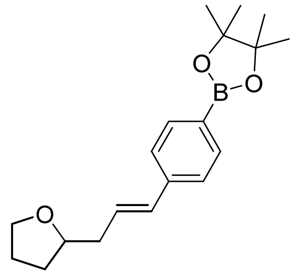

(*E*)-4,4,5,5-tetramethyl-2-(4-(3-(tetrahydrofuran-2-yl)prop-1-en-1-yl)-phenyl)-1,3,2-dioxaborolane (**7l**): The reaction was performed on 0.15 mmol scale. The pure product was obtained after column chromatography (pentane/ethyl acetate: 9/1, R_f: 0.5) as a yellow oil (32 mg, 73%). **^{1}H NMR (300 MHz, $CDCl_3$): δ** 7.73 (d, J = 8.1 Hz, 2H), 7.35 (d, *J* = 7.9 Hz, 2H), 6.46 (d, *J* = 16.0 Hz, 1H), 6.31 (dt, *J* = 15.9, 6.9 Hz, 1H), 4.01-3.87 (m, 2H), 3.79-3.71 (m, 1H), 2.56-2.36 (m, 2H), 2.05-1.82 (m, 3H), 1.62-1.53 (m, 1H), 1.34 (s, 12H). **^{13}C NMR (75 MHz, $CDCl_3$): δ** 140.4, 135.1 (2 C_{Ar}), 132.1, 128.2, 125.5 (2 C_{Ar}), 83.8, 78.9, 68.1, 39.4, 31.0, 25.9, 25.0 (4 CH_3). **EI-MS:** Calculated for $C_{19}H_{27}BO_3$: 314.2053, Found: 314.2723.

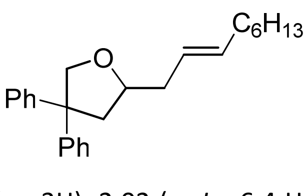

(*E*)-2-(non-2-en-1-yl)-4,4-diphenyltetrahydrofuran (**8a**): The pure product was obtained after column chromatography (pentane/ethyl acetate: 96/4, R_f: 0.5) as a yellow oil (104 mg, 75%). **^{1}H NMR (300 MHz, $CDCl_3$): δ** 7.35-7.27 (m, 6H), 7.25-7.17 (m, 4H), 5.58-5.37 (m, 2H), 4.64 (dd, *J* = 8.7, 1.2 Hz, 1H), 4.16 (d, *J* = 8.8 Hz, 1H), 4.14-4.04 (m, 1H), 2.60 (ddd, *J* = 12.2, 5.8, 1.2 Hz, 1H), 2.45-2.23 (m, 3H), 2.02 (q, *J* = 6.4 Hz, 2H), 1.42-1.24 (m, 8H), 0.93-0.89 (m, 3H). **^{13}C NMR (75 MHz, $CDCl_3$): δ** 146.5, 146.2, 133.6, 128.5 (2 C_{Ar}), 128.4 (2 C_{Ar}), 127.3 (2 C_{Ar}), 127.3 (2 C_{Ar}), 126.5, 126.3, 125.7, 78.7, 77.0, 56.1, 44.4, 39.2, 32.8, 31.8, 29.5, 29.0, 22.8, 14.2. **EI-MS:** Calculated for $C_{25}H_{32}O$: 348.2453, Found: 348.5099.

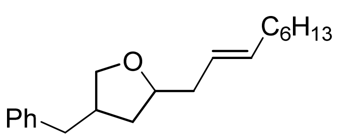

(*E*)-4-benzyl-2-(non-2-en-1-yl)tetrahydrofuran (**8b**): The pure product was obtained after column chromatography (pentane/ethyl acetate: 96/4, R_f: 0.4) as a yellow oil (79 mg, 69%). Data for the major diastereoisomer: **^{1}H NMR (500 MHz, $CDCl_3$): δ** 7.32-7.26 (m, 2H), 7.23-7.15 (m, 3H), 5.57-5.34 (m, 2H), 3.92-3.82 (m, 2H), 3.57 (dd, *J* = 8.4, 7.1 Hz, 1H), 2.27-2.65 (m, 2H), 2.63-2.53 (m, 2H), 2.40-2.34 (m, 1H), 2.28-2.22 (m, 1H), 2.09 (ddd, J = 12.7, 7.3, 5.8 Hz, 1H), 1.39-1.21 (m, 8H), 1.28 (dt, J = 12.3, 9.2 Hz, 2H), 0.92-0.87 (m, 3H). **^{13}C NMR (126 MHz, $CDCl_3$): δ** 140.9, 133.2, 128.7, 128.4 (2 C_{ar}), 126.1 (2 C_{ar}), 126.0, 79.8, 72.8, 32.7, 31.8, 41.6, 39.9, 39.1, 38.2, 29.5, 28.9, 22.7, 14.2. Data for the minor diastereoisomer: **^{1}H NMR (500 MHz, $CDCl_3$): δ** 7.32-7.26 (m, 2H), 7.23-7.15 (m, 3H), 5.57-5.34 (m, 2H), 4.05 (p, J = 6.7 Hz, 2H), 3.46 (dd, *J* = 8.4, 7.1 Hz, 1H), 2.27-2.65 (m, 2H), 2.63-2.53 (m, 2H), 2.40-2.34 (m, 1H), 2.28-2.22 (m, 1H), 2.09 (ddd, J = 12.7, 7.3, 5.8 Hz, 1H), 1.39-1.21 (m, 8H), 1.28 (dt, J = 12.3, 9.2 Hz, 2H), 0.92-0.87 (m, 3H). **^{13}C NMR (126 MHz, $CDCl_3$): δ** 140.8, 133.2, 128.7, 128.4 (2 C_{ar}), 126.1 (2 C_{ar}), 126.0, 78.6, 73.1, 40.6, 39.4, 39.2, 36.8, 32.7, 31.8, 29.5, 28.9, 22.7, 14.2. **EI-MS:** Calculated for $C_{20}H_{30}O$: 286.2297, Found: 286.3672.

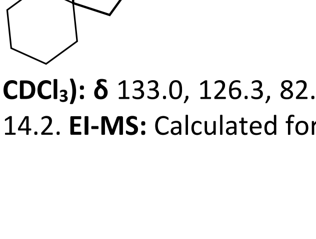

(*E*)-3-(non-2-en-1-yl)-2-oxaspiro[4.5]decane (**8c**): The pure product was obtained after column chromatography (pentane/ethyl acetate: 96/4, R_f: 0.7) as a orange-brown oil (55 mg, 52%). **^{1}H NMR (300 MHz, $CDCl_3$): δ** 5.51-5.32 (m, 2H), 3.98-3.89 (m, 1H), 2.36-2.27 (m, 1H), 2.15-2.06 (m, 1H), 2.01-1.87 (m, 3H), 1.71-1.46 (m, 9H), 1.38-1.25 (m, 12H), 0.90-0.85 (m, 3H). **^{13}C NMR (75 MHz, $CDCl_3$): δ** 133.0, 126.3, 82.7, 77.9, 39.8, 38.7, 37.8, 35.8, 32.8, 31.9, 30.7, 29.6, 29.0, 25.9, 24.3, 24.0, 22.8, 14.2. **EI-MS:** Calculated for $C_{18}H_{32}O$: 264.2453, Found: 264.3274.

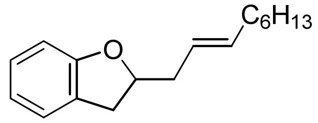

(*E*)-2-(non-2-en-1-yl)-2,3-dihydrobenzofuran (**8d**): The pure product was obtained after column chromatography (pentane/ethyl acetate: 98/2, R_f: 0.7) as a yellow oil (87 mg, 88%). **^{1}H NMR (300 MHz, $CDCl_3$):** 7.18-7.08 (m, 2H), 6.86-6.77 (m, 2H), 5.64-5.54 (m, 1H), 5.51-5.41 (m, 1H), 4.86-4.76 (m, 1H), 3.24 (dd, *J* = 15.6, 9.0 Hz, 1H), 2.93 (dd, *J* = 15.5, 7.5 Hz, 1H), 2.60-2.50 (m, 1H), 2.46-2.36 (m, 1H), 2.07-2.0 (m, 2H), 1.40-1.26 (m, 8H), 0.93-0.89 (m, 3H). **δ ^{13}C NMR (75 MHz, $CDCl_3$): δ** 159.7, 134.5, 128.0, 126.9, 125.0, 124.5, 120.3, 109.4, 82.9, 39.2, 34.8, 32.8, 31.9, 29.5, 28.9, 22.8, 14.2. **EI-MS:** Calculated for $C_{17}H_{24}O$: 244.1827, Found: 244.2720.

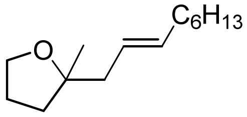

(*E*)-2-methyl-2-(non-2-en-1-yl)tetrahydrofuran (**8e**): The reaction was performed with 5 mol% of [(P,N)AuCl] and 1.05 mol% of AgOTf. The pure product was obtained after column chromatography (pentane/ethyl acetate: 96/4, R_f: 0.7) as a colorless oil (59 mg, 69%). **^{1}H NMR (300 MHz, $CDCl_3$): δ** 5.51-5.35 (m, 2H), 3.88-3.76 (m, 2H), 2.19-2.16 (m, 2H), 2.03-1.96 (m, 2H), 1.94-1.85 (m, 2H), 1.81-1.72 (m, 1H), 1.62-1.53 (m, 1H), 1.37-1.23 (m, 8H), 1.16 (s, 3H), 0.90-0.85 (m, 3H). **^{13}C NMR (75 MHz, $CDCl_3$): δ** 133.8, 126.2, 82.7, 67.4, 44.5, 36.2, 32.8, 31.9, 29.6, 29.0, 26.2, 26.1, 22.8, 14.2. **EI-MS:** Calculated for $C_{14}H_{26}O$: 210.1984, Found 210.1834

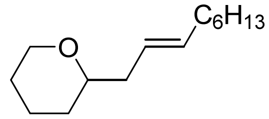

(*E*)-2-(non-2-en-1-yl)tetrahydro-2H-pyran (**8f**): The pure product was obtained after column chromatography (pentane/ethyl acetate: 98/2, R_f: 0.6) as a colorless oil (74 mg, 88%). **^{1}H NMR (300 MHz, $CDCl_3$): δ** 5.51-5.33 (m, 2H), 3.99-3.93 (m, 1H), 3.45-3.35 (m, 1H), 3.28-3.20 (m, 1H), 2.27-2.17 (m, 1H), 2.12-2.03 (m, 1H), 1.99-1.92 (m, 2H), 1.85-1.76 (m, 1H), 1.63-1.42 (m, 4H), 1.37-1.19 (m, 9H), 0.89-0.85 (m, 3H). **^{13}C NMR (75 MHz, $CDCl_3$): δ** 133.0, 126.1, 78.0, 68.7, 40.0, 32.8, 31.9, 31.5, 29.6, 29.0, 26.3, 23.7, 22.8, 14.2. **EI-MS:** Calculated for $C_{14}H_{26}O$: 210.1984, Found: 210.1850.

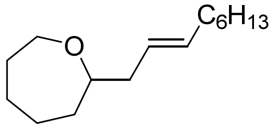

(*E*)-2-(non-2-en-1-yl)oxepane (**8g**): The pure product was obtained after column chromatography (pentane/ethyl acetate: 98/2, R_f: 0.5) as a yellow oil (49 mg, 55%). **^{1}H NMR (300 MHz, $CDCl_3$): δ** 5.50-5.35 (m, 2H), 3.94-3.81 (m, 1H), 3.56-3.36 (m, 2H), 2.25-2.16 (m, 1H), 2.11-2.04 (m, 1H), 2.03-1.95 (m, 2H), 1.77-1.42 (m, 7H), 1.33-1.23 (m, 9H), 0.90-0.85 (m, 3H). **^{13}C NMR (75 MHz, $CDCl_3$): δ** 132.7, 126.9, 79.9, 68.7, 40.3, 35.4, 32.8, 31.9, 31.2, 29.6, 29.0, 26.9, 25.8, 22.8, 14.2. **EI-MS:** Calculated for $C_{15}H_{28}O$: 224.2140, Found: 224.1956.

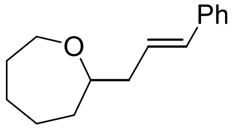

2-cinnamyloxepane (**8h**): The reaction was performed with 5 mol% of [(P,N)AuCl] and 1.05 mol% of AgOTf. The pure product was obtained after column chromatography (pentane/ethyl acetate: 98/2, R_f: 0.6) as a colorless oil (60 mg, 69%). **^{1}H NMR (300 MHz, $CDCl_3$): δ** 7.38-7.26 (m, 4H), 7.23-7.16 (m, 1H), 6.43 (d, *J* = 15.7 Hz, 1H), 6.26 (ddd, *J* = 15.8, 7.5, 6.5 Hz, 1H), 3.93-3.85 (m, 1H), 3.64-3.52 (m, 2H), 2.52-2.39 (m, 1H), 2.37-2.26 (m, 1H), 1.87-1.44 (m, 8H). **^{13}C NMR (75 MHz, $CDCl_3$): δ** 137.8 (C_q), 131.7, 128.6, 128.6, 127.7, 127.1, 126.2, 126.1, 79.7, 68.9, 40.7, 35.7, 31.1, 26.9, 25.9. **EI-MS:** Calculated for $C_{15}H_{20}O$: 216.1514, Found: 216.1956.

The stereochemistry of **8i, 8j** and **8k** was assigned by comparison with previous work. [11]

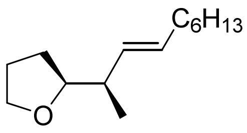

(*S*)-2-((*R*,*E*)-dec-3-en-2-yl)tetrahydrofuran (**8i**): The pure product was obtained from (*Z*)-hex-4-en-1-ol after column chromatography (pentane/ethyl acetate: 98/2, R_f: 0.5) as a yellow oil (73 mg, 85%). **^{1}H NMR (300 MHz, $CDCl_3$): δ** 5.49-5.34 (m, 2H), 3.87-3.80 (m, 1H), 3.75-3.61 (m, 2H), 2.21 (h, *J* = 6.6 Hz, 1H), 2.03-1.96 (m, 2H), 1.92-1.78 (m, 3H), 1.59-1.48 (m, 1H), 1.36-1.23 (m, 8H), 0.98 (d, *J* = 6.9 Hz, 3H), 0.89-0.85 (m, 3H). **^{13}C NMR (75 MHz, $CDCl_3$): δ** 132.6, 130.7, 83.5, 68.3, 41.7, 32.8, 31.9, 29.7, 29.0, 29.0, 26.0, 22.8, 17.0, 14.2. **EI-MS:** Calculated for $C_{14}H_{26}O$: 210.1984, Found: 210.1850. **GC:** t_R: 9.17 min.

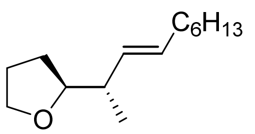

(*S*)-2-((*S*,*E*)-dec-3-en-2-yl)tetrahydrofuran (**8j**): The pure product was obtained from (*E*)-hex-4-en-1-ol after column chromatography (pentane/ethyl acetate: 98/2, R_f: 0.5) as a yellow oil (39 mg, 46%). Data for the major diastereoisomer: **^{1}H NMR (500 MHz, $CDCl_3$): δ** 5.47-5.41 (m, 1H), 5.30 (ddt, *J* = 15.3, 7.5, 1.2 Hz, 1H), 3.85-3.81 (m, 1H), 3.74-3.69 (m, 1H), 3.61-3.57 (m, 1H), 2.19 (h, J = 14.3, 7.0 Hz, 1H), 1.88-1.79 (m, 3H), 1.58-1.51 (m, 3H), 1.38-1.22 (m, 8H), 1.04 (d, *J* = 6.7 Hz, 3H), 0.89-0.86 (m, 3H). **^{13}C NMR (126 MHz, $CDCl_3$): δ** 132.2, 130.9, 83.6, 68.1, 42.1, 32.8, 31.9, 29.6, 29.3, 28.9, 26.0, 22.8, 17.4, 14.2. **EI-MS:** Calculated for $C_{14}H_{26}O$: 210.1984, Found: 210.1850. **GC:** t_R: 9.17 min.

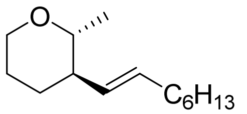

(2*R*,3*R*)-2-methyl-3-((*E*)-oct-1-en-1-yl)tetrahydro-2H-pyran (**8k**): The pure product was obtained from (*E*)-hex-4-en-1-ol after column chromatography (pentane/ethyl acetate: 98/2, R_f: 0.5) as a yellow oil (27 mg, 32%). **^{1}H NMR (500 MHz, $CDCl_3$): δ** 5.44 (dtd, *J* = 15.2, 6.8, 0.8 Hz, 1H), 5.15 (ddt, *J* = 15.3, 8.5, 1.5 Hz, 1H), 3.94 (ddt, *J* = 11.3, 4.6, 1.8 Hz, 1H), 3.38 (ddd, *J* = 12.3, 11.3, 2.5 Hz, 1H), 3.11 (dq, *J* = 9.5, 6.2 Hz, 1H), 1.99-1.94 (m, 1H), 1.85-1.73 (m, 1H), 1.68-1.59 (m, 3H), 1.56-1.52 (m, 1H), 1.33 (tdd, *J* = 13.0, 11.7, 4.2 Hz, 1H), 1.13 (d, *J* = 6.2 Hz, 3H), 1.39-1.23 (m, 8H), 0.89-0.85 (m, 3H). **^{13}C NMR (126 MHz, $CDCl_3$): δ** 131.8, 131.7, 77.8, 68.3, 47.3, 32.8, 31.8, 31.0, 29.6, 28.9, 26.2, 22.8, 20.3, 14.2. **EI-MS:** Calculated for $C_{14}H_{26}O$: 210.1984, Found: 210.1850. **GC:** t_R: 9.22 min.

2.4.3 Catalytic oxy-vinylation under air with reagent-grade chemicals

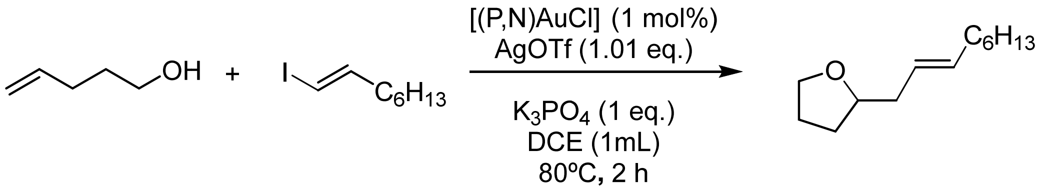

Under air, a flame dried Schlenk equipped with a magnetic stirrer bar was charged with the silver trifluoromethanesulfonate (103 mg, 0.42 mmol) and potassium phosphate tribasic (85 mg, 0.40 mmol) in 0.4 mL of DCE. [(P,N)AuCl] (2.6 mg, 0.004 mmol), the vinyl iodide (0.4 mmol), 4-penten-1-ol (0.4 mmol), and internal standard were transferred into a small glass vial dissolved in 0.6 mL of DCE (reagent grade from commercial bottle, not degassed or dried). This solution was loaded into a plastic syringe equipped with stainless steel needle. The Schlenk was cooled down to –10 °C (acetone/N_2 cold bath). At this temperature, the solution of gold complex was added. The reaction mixture was then stirred at 80°C during 2 hours. The yield in heterovinylation product **7a** was determined by ^{1}H NMR using dimethyl terephthalate as internal standard (NMR yield: 98%)

2.4.4 Catalytic oxy-vinylation catalyzed by gold(III) complex

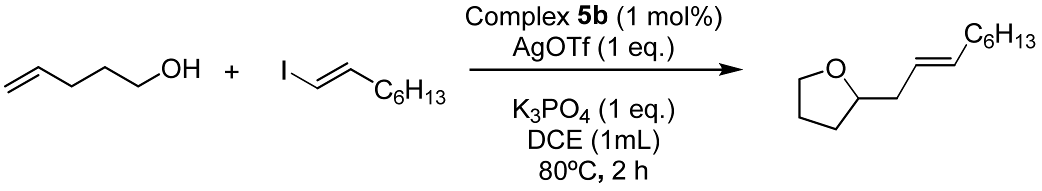

In a glovebox, a flame dried Schlenk equipped with a magnetic stirrer bar was charged with the silver trifluoromethanesulfonate (98 mg, 0.40 mmol) and potassium phosphate tribasic (85 mg, 0.40 mmol) in 0.4 mL of solvent. Gold(III) complex **X** (4.4 mg, 0.004 mmol), the vinyl iodide (0.4 mmol), the functionalized alkenol (0.4 mmol), and internal standard were transferred into a small glass vial dissolved in 0.6 mL of solvent. This solution was loaded into a plastic syringe equipped with stainless steel needle. The syringe was

closed by blocking the needle with a septum. Outside the glovebox, the Schlenk was cooled down to –10 °C (acetone/N_2 cold bath). At this temperature, the solution of gold complex was added. The reaction mixture was then stirred at 80°C during 2 hours. The yield in heterovinylation product was determined by ^{1}H NMR using dimethyl terephthalate as internal standard. (NMR yield: 98%, exactly as in the case of the catalytic run starting from the (P,N) gold(I) complex).

2.4.5 Monitoring catalytic oxy-vinylation catalyzed (MeDalphos)AuCl

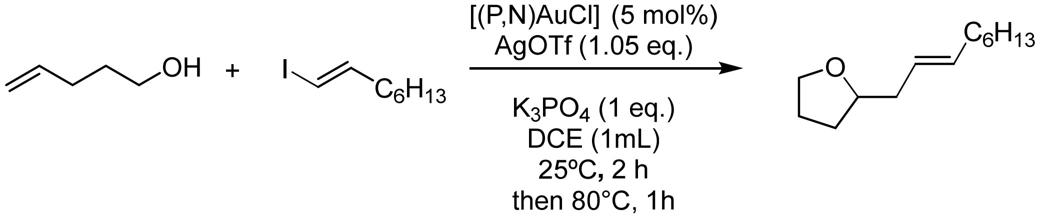

In a glovebox, a flame dried Schlenk equipped with a magnetic stirrer bar was charged with the silver trifluoromethanesulfonate (108 mg, 0.404 mmol) and potassium phosphate tribasic (85 mg, 0.40 mmol) in 0.4 mL of solvent. [(P,N)AuCl] (13 mg, 0.004 mmol), the vinyl iodide (95.4 mg, 0.4 mmol), the functionalized alkenol (34.2, 0.4 mmol), and DMT (internal standard) were transferred into a small glass vial dissolved in 0.6 mL of solvent. This solution was loaded into a plastic syringe equipped with stainless steel needle. The syringe was closed by blocking the needle with a septum. Outside the glovebox, the Schlenk was cooled down to –10 °C (acetone/N_2 cold bath). At this temperature, the solution of gold complex was added. The reaction mixture was then stirred at 25°C during 2 hours then 1 hour at 80°C. An aliquot was taken to perform ^{1}H NMR and ^{31}P NMR.

T_1 After 1h at 25°C: using DMT as IS for ^{1}H NMR, 40% conversion of the vinyl iodide and 70% of π-complex. NMR product yield: **30%.** ^{31}P NMR: 57.9 (π-complex confirm by ^{1}H NMR)
T_2 After 1h at 25°C: using DMT as IS for ^{1}H NMR, 66% conversion of the vinyl iodide and 30% of π-complex. NMR product yield: **55%.** ^{31}P NMR: 57.8 (π-complex confirm by ^{1}H NMR)
T_3 After 1h at 80°C: using DMT as IS for ^{1}H NMR, 100% conversion of the vinyl iodide. NMR product yield: **99%.** ^{31}P NMR: no peak of π-complex was observed.

2.5 Gold-catalyzed Amino-vinylation of alkenes

2.5.1 Synthesis of substrates

4-methyl-N-(pent-4-en-1-yl)benzenesulfonamide[11] and N-(2,2-dimethylpent-4-en-1-yl)-4-methylbenzenesulfonamide[15] were prepared according to reported procedures.

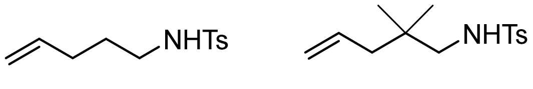

2.5.2 General procedure for the gold-catalyzed Amino-vinylation of alkenes

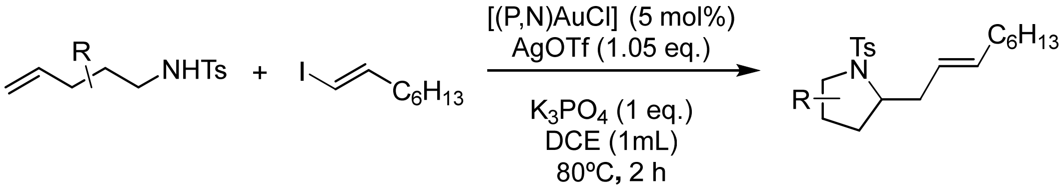

In a glovebox, a flame dried Schlenk equipped with a magnetic stirrer bar was charged with the silver trifluoromethanesulfonate (108 mg, 0.42 mmol) and potassium phosphate tribasic (85 mg, 0.40 mmol) in 0.4 mL of solvent. [(P,N)AuCl] (13 mg, 0.02 mmol), the vinyl iodide (0.4 mmol), the functionalized alkene

(0.4 mmol), and internal standard were transferred into a small glass vial dissolved in 0.6 mL of solvent. This solution was loaded into a plastic syringe equipped with stainless steel needle. The syringe was closed by blocking the needle with a septum. Outside the glovebox, the Schlenk was cooled down to –10 °C (acetone/N_2 cold bath). At this temperature, the solution of gold complex was added. The reaction mixture was then stirred at 80°C during 2 hours. The yield in heterovinylation product was determined by ^{1}H NMR using dimethyl terephthalate as internal standard. To isolate the product, silver salts were removed by filtration over celite and the sample was purified by column chromatography.

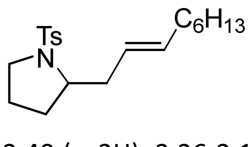

(*E*)-2-(non-2-en-1-yl)-1-tosylpyrrolidine (**8l**): The pure product was obtained after column chromatography (pentane/ethyl acetate: 9/1, R_f: 0.6) as a yellow oil (120 mg, 86%). **^{1}H NMR (300 MHz, CDCl$_3$): δ** 7.72-7.68 (m, 2H), 7.30-7.27 (m, 2H), 5.52-5.28 (m, 2H), 3.63-3.55 (m, 1H), 3.40-3.33 (m, 1H), 3.19-3.10 (m, 1H), 2.54-2.46 (m, 1H), 2.40 (s, 3H), 2.26-2.15 (m, 1H), 2.00-1.93 (m, 2H), 1.79-1.42 (m, 4H), 1.36-1.21 (m, 8H), 0.88-0.84 (m, 3H). **^{13}C NMR (75 MHz, CDCl$_3$): δ** 143.3 (C_q),135.0 (C_q), 134.0, 129.7 (2 C_{ar}), 127.5 (2 C_{ar}), 125.8, 60.2, 49.3, 39.7, 32.7, 31.8, 30.0, 29.4, 28.9, 24.0, 22.7, 21.6, 14.2. **EI-MS:** Calculated for $C_{20}H_{31}NO_2S$: 349.2075, Found: 349.1464.

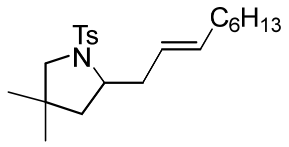

(*E*)-4,4-dimethyl-2-(non-2-en-1-yl)-1-tosylpyrrolidine (**8m**): The pure product was obtained after column chromatography (pentane/ethyl acetate: 9/1, R_f: 0.6) as a yellow oil (139 mg, 92%). **^{1}H NMR (300 MHz, CDCl$_3$): δ** 7.73-7.68 (m, 2H), 7.30-7.27 (m, 2H), 5.51-5.41 (m, 1H), 5.35-5.24 (m, 1H), 3.64-3.55 (m, 1H), 3.14-3.05 (m, 2H), 2.73-2.65 (m, 1H), 2.40 (s, 3H), 2.36-2.25 (m, 1H), 1.99-1.92 (m, 2H), 1.64-1.45 (m, 2H), 1.34-1.21 (m, 8H), 0.99 (s, 3H), 0.88-0.84 (m, 3H), 0.50 (s, 3H). **^{13}C NMR (75 MHz, CDCl$_3$): δ** 143.2 (C_q),135.7 (C_q), 134.1, 129.6 (2 C_{ar}), 127.5 (2 C_{ar}), 125.5, 61.7, 60.1, 45.6, 39.4, 37.3, 32.8, 31.8, 29.5, 28.9, 26.5, 26.0, 22.7, 21.6, 14.2. **EI-MS:** Calculated for $C_{22}H_{35}NO_2S$: 377.2389, Found: 377.3470

2.6 Gold-catalyzed 3-component oxyvinylation

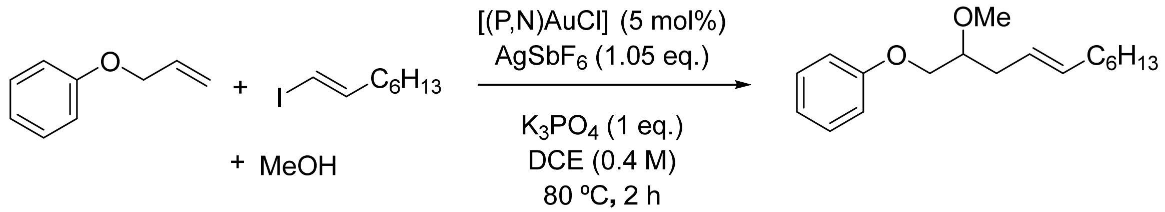

In a glovebox, a flame dried Schlenk equipped with a magnetic stirrer bar was charged with silver hexafluoroantimonate (144 mg, 0.42 mmol) and potassium phosphate tribasic (85 mg, 0.4 mmol) in dichloroethane (0.4 mL). (P,N)AuCl complex (13 mg, 0.02 mmol)), vinyl iodide (95.4 mg, 0.4 mmol, 1 eq.), allylphenylether (54 mg, 0.4 mmol, 1 eq.), methanol (128 mg, 4 mmol, 10 eq.) and internal standard were transferred into a small glass vial and dissolved in dichloroethane (0.6 mL). This solution was loaded into a plastic syringe equipped with stainless steel needle. The syringe was closed by blocking the needle with a septum. Outside the glovebox, the Schlenk was cooled down to -10 °C (Ethanol/N_2 cold bath). At this temperature, the solution was added. The reaction mixture was then stirred at 80 °C for 2h. The yield in 1,2-oxyvinylation product was determined by ^{1}H NMR using diethyl terephthalate as internal standard. To isolate the product, silver salts were removed by filtration over celite and the sample was purified by column chromatography.

(*E*)-((2-methoxyundec-4-en-1-yl)oxy)benzene (**8n**): The pure product was obtained after column chromatography (pentane, R_f: 0.4) as a yellow oil (73 mg, 66%). **^{1}H NMR (300 MHz, $CDCl_3$): δ** 7.32-7.25 (m, 2H), 6.98-6.90 (m, 3H), 5.59-5.39 (m, 2H), 4.03-3.94 (m, 2H), 3.62-3.54 (m, 2H), 3.49 (s, 3H), 2.40-2.35 (m, 2H), 2.01 (q, *J* = 6.6 Hz, 2H), 1.39-1.24 (m, 8H), 0.91-0.86 (m, 3H). **^{13}C NMR (75 MHz, $CDCl_3$): δ** 159.0 (C_q), 134.1, 129.5 (2 C_{ar}), 125.1, 120.9, 114.8 (2 C_{ar}), 79.6, 69.4, 57.8, 34.5, 32.8, 31.9, 29.5, 29.0, 22.8, 14.2. **EI-MS:** Calculated for $C_{18}H_{28}O_2$: 276.2089, Found: 276.1707.

2.7 Gold-catalyzed heterovinylation/heteroarylation of alkenes

2.7.1 General procedure for the gold-catalyzed heterovinylation from bifunctional substrate

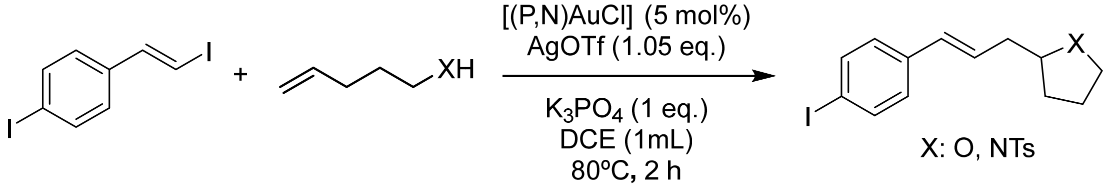

In a glovebox, a flame dried Schlenk equipped with a magnetic stirrer bar was charged with the silver trifluoromethanesulfonate (108 mg, 0.42 mmol) and potassium phosphate tribasic (85 mg, 0.40 mmol) in 0.4 mL of solvent. [(P,N)AuCl] (13 mg, 0.02 mmol), the vinyl iodide **4f** (0.4 mmol), the functionalized alkene (0.4 mmol), and internal standard were transferred into a small glass vial dissolved in 0.6 mL of solvent. This solution was loaded into a plastic syringe equipped with stainless steel needle. The syringe was closed by blocking the needle with a septum. Outside the glovebox, the Schlenk was cooled down to –10 °C (acetone/N_2 cold bath). At this temperature, the solution of gold complex was added. The reaction mixture was then stirred at 80°C during 2 hours. The yield in heterovinylation product was determined by ^{1}H NMR using dimethyl terephthalate as internal standard. To isolate the product, silver salts were removed by filtration over celite and the sample was purified by column chromatography.

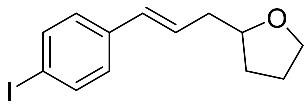

(*E*)-2-(3-(4-iodophenyl)allyl)tetrahydrofuran (**9a**): The pure product was obtained after column chromatography (pentane/ethyl acetate: 9/1, R_f: 0.6) as a yellow oil (70 mg, 56%). **^{1}H NMR (300 MHz, $CDCl_3$): δ** 7.60 (d, *J* = 8.4 Hz, 2H), 7.08 (d, *J* = 8.5 Hz, 2H), 6.37 (d, *J* = 15.9 Hz, 1H), 6.25 (dt, *J* = 15.9, 6.8 Hz, 1H), 3.99-3.89 (m, 2H), 3.78-3.73 (m, 1H), 2.47-2.39 (m, 2H), 2.03-1.84 (m, 3H), 1.61-1.51 (m, 1H). **^{13}C NMR (75 MHz, $CDCl_3$): δ** 137.6 (2 C_{ar}), 137.2 (C_q), 131.0, 128.1, 128.0 (2 C_{ar}), 92.2 (C_q), 78.7, 68.1, 39.3, 31.1, 25.8. **EI-MS:** Calculated for $C_{13}H_{15}IO$: 314.0168, Found: 313.9594.

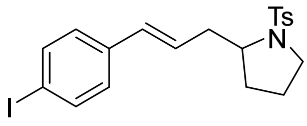

(*E*)-2-(3-(4-iodophenyl)allyl)-1-tosylpyrrolidine (**9b**): The pure product was obtained after column chromatography (pentane/ethyl acetate: 8/2, R_f: 0.5) as a yellow oil (96 mg, 52%). **^{1}H NMR (300 MHz, $CDCl_3$): δ** 7.73 (d, *J* = 8.2 Hz, 2H), 7.61 (d, *J* = 8.4 Hz, 2H), 7.30 (d, *J* = 7.9 Hz, 2H), 7.08 (d, *J* = 8.1 Hz, 2H), 6.36 (d, *J* = 15.9 Hz, 1H), 6.21 (dt, *J* = 15.9, 7.1 Hz, 1H), 3.73 (tt, *J* = 7.9, 4.0 Hz, 1H), 3.45-3.37 (m, 1H), 3.18 (dt, *J* = 10.2, 7.2 Hz, 1H), 2.73-2.65 (m, 1H), 2.53-2.45 (m, 1H), 2.42 (s, 3H), 1.86-1.46 (m, 4H). **^{13}C NMR (75 MHz, $CDCl_3$): δ** 143.4 (C_q), 137.7 (2 C_{ar}), 137.0 (C_q), 134.8 (C_q), 131.7, 129.77 (2 C_{ar}), 128.0 (2 C_{ar}), 127.6 (2 C_{ar}), 127.4, 92.4 (C_q), 59.9, 49.3, 40.1, 30.4, 24.1, 21.6. **EI-MS:** Calculated for $C_{20}H_{22}INO_2S$: 467.0416, Found: 466.9984.

2.7.2 Synthetic application from 9a

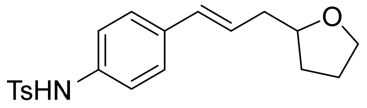

(*E*)-4-methyl-N-(4-(3-(tetrahydrofuran-2-yl)prop-1-en-1-yl)phenyl)-benzenesulfonamide (**10a**): Following the procedure,[16] the pure product was obtained after column chromatography (pentane/ethyl acetate: 7/3, R_f: 0.3) as a yellow oil (125 mg, 87%). **^{1}H NMR (300 MHz, $CDCl_3$): δ** 7.65 (d, *J* = 8.3 Hz, 2H), 7.30-7.28 (m, 1H), 7.21-7.15 (m, 4H), 6.99 (d, *J* = 8.5 Hz, 2H), 6.33 (d, *J* = 15.9 Hz, 1H), 6.12

(dt, J = 15.9, 7.1 Hz, 1H), 3.98-3.85 (m, 2H), 3.78-3.71 (m, 1H), 2.49-2.38 (m, 2H), 2.35 (s, 3H), 2.04-1.81 (m, 3H), 1.60-1.48 (m, 1H). **^{13}C NMR (75 MHz, CDCl$_3$): δ** 143.8 (C$_q$), 136.1 (C$_q$), 135.4 (C$_q$), 134.7 (C$_q$), 131.0, 129.7 (2 C$_{ar}$), 127.3 (2 C$_{ar}$), 126.9 (2 C$_{ar}$), 126.8, 121.7 (2 C$_{ar}$), 78.8, 68.0, 39.2, 31.0, 25.7, 21.6. **EI-MS:** Calculated for $C_{20}H_{23}NO_3S$: 357.1399, Found: 357.0088.

.

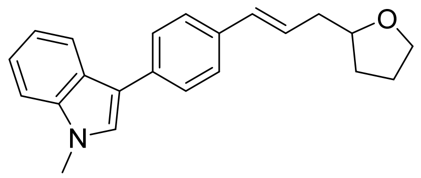

(*E*)-1-methyl-3-(4-(3-(tetrahydrofuran-2-yl)prop-1-en-1-yl)phe-nyl)-1H-indole (**10b**): Following the procedure, [17] the pure product was obtained after column chromatography (pentane/ethyl acetate: 9/1, R$_f$: 0.2) as a pink pale solid (47 mg, 62%). **^{1}H NMR (300 MHz, CDCl$_3$): δ** 7.97 (ddd, J = 7.9, 1.3, 0.8 Hz, 1H), 7.64-7.60 (m, 2H), 7.47-7.43 (m, 2H), 7.38 (ddd, J = 8.2, 1.3, 0.8 Hz, 1H), 7.30 (ddd, J = 8.2, 6.9, 1.2 Hz, 1H), 7.24-7.19 (m, 2H), 6.52 (d, J = 15.9 Hz, 1H), 6.28 (dt, J = 15.8, 7.1 Hz, 1H), 4.06-3.91 (m, 2H), 3.83 (s, 3H), 3.82-3.75 (m, 1H), 2.56-2.45 (m, 2H), 2.09-1.87 (m, 3H), 1.67-1.56 (m, 1H). **^{13}C NMR (75 MHz, CDCl$_3$): δ** 137.6 (C$_q$), 135.1 (C$_q$), 134.6 (C$_q$), 131.9, 127.3 (2 C$_{ar}$), 126.6 (2 C$_{ar}$), 126.6, 126.2 (C$_q$), 126.0, 122.0, 120.1, 120.0, 116.6 (C$_q$), 109.7, 79.0, 68.1, 39.4, 33.0, 31.0, 25.9. **EI-MS:** Calculated for $C_{22}H_{23}NO$: 317.1780, Found: 317.1160.

2.7.3 General procedure for the gold-catalyzed oxy-vinylation/oxy-arylation from bifunctional substrate

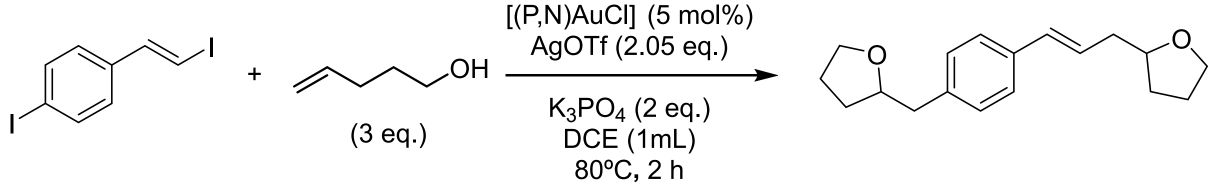

In a glovebox, a flame dried Schlenk equipped with a magnetic stirrer bar was charged with the silver trifluoromethanesulfonate (210 mg, 0.82 mmol) and potassium phosphate tribasic (170 mg, 0.80 mmol) in 0.2 mL of solvent. [(P,N)AuCl] (13 mg, 0.02 mmol), the vinyl iodide **4f** (142 mg, 0.4 mmol), the functionalized alkene (102.6 mg, 1.2 mmol), and internal standard were transferred into a small glass vial dissolved in 0.8 mL of solvent. This solution was loaded into a plastic syringe equipped with stainless steel needle. The syringe was closed by blocking the needle with a septum. Outside the glovebox, the Schlenk was cooled down to –10 °C (acetone/N_2 cold bath). At this temperature, the solution of gold complex was added. The reaction mixture was then stirred at 80°C during 2 hours. The yield in heterovinylation product was determined by ^{1}H NMR using dimethyl terephthalate as internal standard. To isolate the product, silver salts were removed by filtration over celite and the sample was purified by column chromatography.

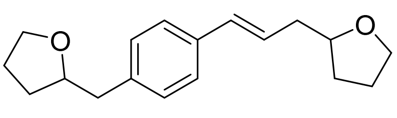

(*E*)-2-(3-(4-((tetrahydrofuran-2-yl)methyl)phenyl)allyl)tetrahydro furan (**11a**): The pure product was obtained after column chromatography (Pentane/Ethyl Acetate: 9/1, R$_f$: 0.3) as a viscous pale-yellow oil (94 mg, 85%). **^{1}H NMR (300 MHz, CDCl$_3$): δ** 7.31 (d, J = 8.2 Hz, 2H), 7.18 (d, J = 8.2 Hz, 2H), 6.46 (dt, J = 15.8, 1.4 Hz, 1H), 6.23 (dt, J = 15.9, 7.1 Hz, 1H), 4.12-4.03 (m, 1H), 4.01-3.88 (m, 3H), 3.81-3.71 (m, 2H), 2.92 (dd, J = 13.6, 6.4 Hz, 1H), 2.75 (dd, J = 13.6, 6.5 Hz, 1H), 2.55-2.40 (m, 2H), 2.05-1.82 (m, 6H), 1.65-1.52 (m, 2H). **^{13}C NMR (75 MHz, CDCl$_3$): δ** 137.9 (C$_q$), 135.7 (C$_q$), 131.9, 129.4 (2 C$_{ar}$), 126.2, 126.1 (2 C$_{ar}$), 80.1, 78.9, 68.1, 68.0, 41.7, 39.3, 31.0, 30.9, 25.8, 25.7. **EI-MS:** Calculated for $C_{18}H_{24}O_2$: 272.1776, Found: 272.1049.

2.7.4 General procedure for the gold-catalyzed heterovinylation/heteroarylation from bifunctional substrate

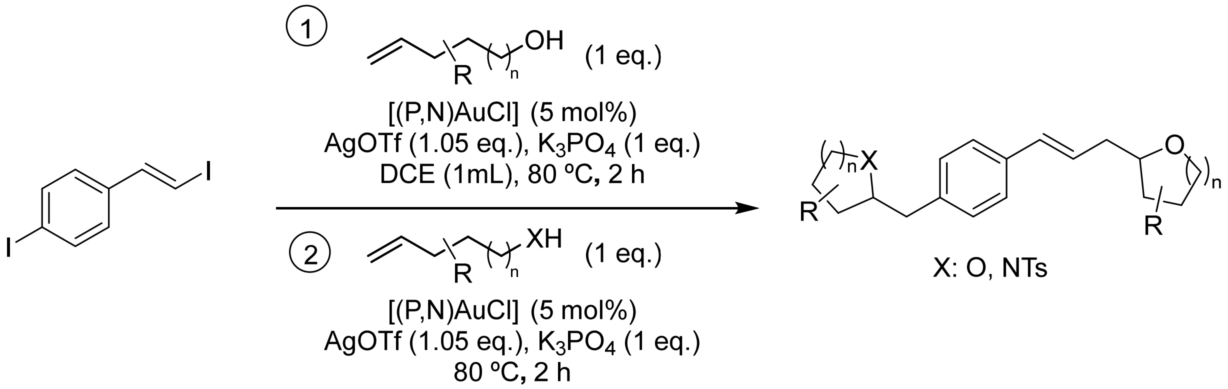

In a glovebox, two flame dried schlenks equipped with a magnetic stirrer bar were charged respectively with the silver trifluoromethanesulfonate (108 mg, 0.42 mmol) and potassium phosphate tribasic (85 mg, 0.80 mmol). 0.2 mL of DCE was added in one of two schlenk. A first small glass vial was charged with [(P,N)AuCl] (13 mg, 0.02 mmol), the vinyl iodide (142 mg, 0.4 mmol), the functionalized alkene (0.4 mmol), and internal standard and was dissolved in 0.8 mL of DCE. In a second small glass vial, [(P,N)AuCl] (13 mg, 0.02 mmol), and the functionalized alkene (0.4 mmol) was dissolved in 0.2 mL of DCE. These solutions were, respectively, loaded into a plastic syringe equipped with stainless steel needle. The syringes were closed by blocking the needles with a septum. Outside the glovebox, the schlenk containing the solvent was cooled down to -10°C (acetone/N_2 cold bath). At this temperature, the solution of gold complex, vinyl iodide, functionalized alkene and IS was added. The reaction mixture was then stirred at 80°C during 2 hours. Subsequently, under argon, the mixture was transferred directly in the second schlenk by cannula and was cooled down to -10°C (acetone/N_2 cold bath). At this temperature, the second solution of gold complex and functionalized alkene was added. The reaction mixture was then stirred at 80°C during 2 hours. The yield in heterovinylation/heteroarylation product over two steps was determined by ^{1}H NMR using dimethyl terephthalate as internal standard. To isolate the product, silver salts were removed by filtration over celite and the sample was purified by column chromatography.

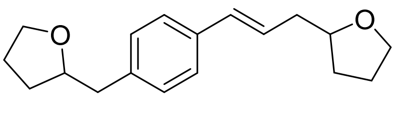

(*E*)-2-(3-(4-((tetrahydrofuran-2-yl)methyl)phenyl)allyl)tetrahydrofuran (**11a**): The pure product was obtained after column chromatography (pentane/ethyl acetate: 9/1, R_f: 0.5) as a viscous pale-yellow oil (60 mg, 55% over two steps). Analytical data are consistent with those previously example. **^{1}H NMR (300 MHz, CDCl$_3$): δ** 7.31 (d, *J* = 8.2 Hz, 2H), 7.18 (d, *J* = 8.2 Hz, 2H), 6.46 (dt, *J* = 15.8, 1.4 Hz, 1H), 6.23 (dt, *J* = 15.9, 7.1 Hz, 1H), 4.12-4.03 (m, 1H), 4.01-3.88 (m, 3H), 3.81-3.71 (m, 2H), 2.92 (dd, *J* = 13.6, 6.4 Hz, 1H), 2.75 (dd, *J* = 13.6, 6.5 Hz, 1H), 2.55-2.40 (m, 2H), 2.05-1.82 (m, 6H), 1.65-1.52 (m, 2H).

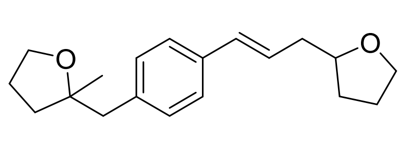

(*E*)-2-methyl-2-(4-(3-(tetrahydrofuran-2-yl)prop-1-en-1-yl)benzyl) tetrahydrofuran (**11b**): The pure product was obtained after column chromatography (pentane/ethyl acetate: 9/1, R_f: 0.5) as a colorless oil (50 mg, 44% over two steps). **^{1}H NMR (300 MHz, CDCl$_3$): δ** 7.29-7.25 (m, 2H), 7.17-7.13 (m, 2H), 6.43 (d, *J* = 16.0 Hz, 1H), 6.20 (dt, *J* = 15.8, 7.1 Hz, 1H), 3.99-3.70 (m, 5H), 2.75 (s, 2H), 2.54-2.34 (m, 2H), 2.04-1.76 (m, 6H), 1.62-1.53 (m, 2H), 1.17 (s, 3H). **^{13}C NMR (75 MHz, CDCl$_3$): δ** 137.6 (C_q), 135.6 (C_q), 131.9, 130.6 (2 C_{ar}), 126.1, 125.6 (2 C_{ar}), 83.0, 79.0, 68.1, 67.5, 46.7, 39.4, 36.3, 31.0, 26.6, 26.2, 25.8. **EI-MS:** Calculated for $C_{19}H_{26}O_2$: 286.1933, Found: 286.3047.

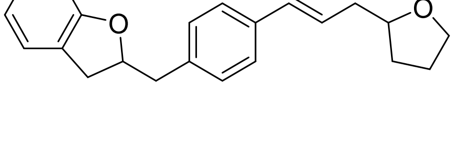

(*E*)-2-(4-(3-(tetrahydrofuran-2-yl)prop-1-en-1-yl)benzyl)-2,3-dihydrobenzofuran (**11c**): The pure product was obtained after column chromatography (pentane/ethyl acetate: 9/1, R_f: 0.4) as a

colorless oil (41mg, 29% over two steps). **^{1}H NMR (300 MHz, $CDCl_3$): δ** 7.33-7.30 (m, 2H), 7.21-7.18 (m, 2H), 7.15-7.08 (m, 2H), 6.88-6.77 (m, 2H), 6.44 (d, J = 15.8 Hz, 1H), 6.22 (dt, J = 15.9, 7.1 Hz, 1H), 5.04-4.94 (m, 1H), 4.02-3.87 (m, 2H), 3.79-3.72 (m, 1H), 3.24-3.13 (m, 2H), 2.98-2.87 (m, 2H), 2.55-2.35 (m, 2H), 2.05-1.83 (m, 3H), 1.63-1.51 (m, 1H). **^{13}C NMR (75 MHz, $CDCl_3$): δ** 159.4 (C_q), 136.3 (C_q), 136.1 (C_q), 131.6, 129.5 (2 C_{ar}), 128.0, 126.7 (C_q), 126.5, 126.3 (2 C_{ar}), 125.0, 120.3, 109.5, 83.5, 78.8, 68.0, 41.7, 39.3, 34.9, 30.9, 25.8. **EI-MS:** Calculated for $C_{22}H_{24}O_2$: 320.1776, Found: 320.0297.

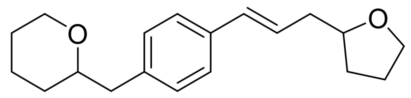

(*E*)-2-(4-(3-(tetrahydrofuran-2-yl)prop-1-en-1-yl)benzyl)tetrahy-dro-2H-pyran (**11d**): The pure product was obtained after column chromatography (pentane/ethyl acetate: 9/1, R_f: 0.5) as a colorless oil (55 mg, 48% over two steps). **^{1}H NMR (300 MHz, $CDCl_3$): δ** 7.29-7.25 (m, 2H), 7.14-7.12 (m, 2H), 6.43 (d, J = 15.8 Hz, 1H), 6.19 (dt, J = 15.8, 7.1 Hz, 1H), 4.00-3.86 (m, 3H), 3.78-3.71 (m, 1H), 3.50-3.35 (m, 2H), 2.84 (dd, J = 13.7, 6.6 Hz, 1H), 2.61 (dd, J = 12.9, 7.2 Hz, 1H), 2.56-2.36 (m, 2H), 2.04-1.76 (m, 4H), 1.68-1.20 (m, 6H). **^{13}C NMR (75 MHz, $CDCl_3$): δ** 137.8 (C_q), 135.6 (C_q), 131.9, 129.6 (2 C_{ar}), 126.2, 126.1 (2 C_{ar}), 79.0, 78.9, 68.8, 68.1, 43.0, 39.3, 31.6, 30.9, 26.2, 25.9, 23.6. **EI-MS:** Calculated for $C_{19}H_{26}O_2$: 286.1933, Found: 286.0544.

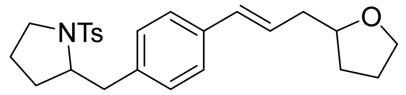

(*E*)-2-(4-(3-(tetrahydrofuran-2-yl)prop-1-en-1-yl)benzyl)-1-tosyl-pyrrolidine (**11e**): The pure product was obtained after column chromatography (pentane/ethyl acetate: 7/3, R_f: 0.5) as a viscous white oil (71 mg, 42% over two steps). **^{1}H NMR (300 MHz, $CDCl_3$): δ** 7.75 (d, J = 8.3 Hz, 2H), 7.30-7.27 (m, 4H), 7.16 (d, J = 8.1 Hz, 2H), 6.43 (d, J = 15.9 Hz, 1H), 6.21 (dt, J = 15.8, 7.0 Hz, 1H), 3.99-3.83 (m, 2H), 3.80-3.71 (m, 2H), 3.41-3.34 (m, 1H), 3.22-3.08 (m, 2H), 2.78-2.70 (m, 1H), 2.53-2.34 (m, 2H), 2.42 (s, 3H), 2.04-1.83 (m, 3H), 1.66-1.37 (m, 5H). **^{13}C NMR (75 MHz, $CDCl_3$): δ** 143.1 (C_q), 137.4 (C_q), 135.9 (C_q), 134.8 (C_q), 131.7, 129.9 (2 C_{ar}), 129.8 (2 C_{ar}), 127.6 (2 C_{ar}), 126.5, 126.2 (2 C_{ar}), 78.9, 68.1, 61.6, 49.3, 42.5, 39.3, 31.0, 30.0, 25.8, 23.9, 21.6. **EI-MS:** Calculated for $C_{25}H_{31}NO_3S$: 425.2025, Found: 425.1975.

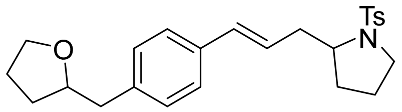

(*E*)-2-(3-(4-((tetrahydrofuran-2-yl)methyl)phenyl)allyl)-1-tosyl-pyrolidine (**11f**): The pure product was obtained after column chromatography (pentane/ethyl acetate: 7/3, R_f: 0.5) as a viscous white oil (82 mg, 48%). **^{1}H NMR (300 MHz, $CDCl_3$): δ** 7.74 (d, J = 8.3 Hz, 2H), 7.32-7.26 (m, 4H), 7.16 (d, J = 8.2 Hz, 2H), 6.41 (d, J = 15.8 Hz, 1H), 6.14 (dt, J = 15.8, 7.3 Hz, 1H), 4.09-4.00 (m, 1H), 3.92-3.85 (m, 1H), 3.77-3.67 (m, 2H), 3.45-3.38 (m, 1H), 3.22-3.14 (m, 1H), 2.89 (dd, J = 13.6, 6.5 Hz, 1H), 2.72 (dd, J = 13.6, 6.4 Hz, 1H), 2.75-2.66 (m, 1H), 2.49-2.44 (m, 1H), 2.42 (s, 3H), 1.94-1.48 (m, 8H). **^{13}C NMR (75 MHz, $CDCl_3$): δ** 143.4 (C_q), 138.2 (C_q), 135.5 (C_q), 134.9 (C_q), 132.7, 129.8 (2 C_{ar}), 129.5 (2 C_{ar}), 127.6 (2 C_{ar}), 126.2 (2 C_{ar}), 125.5, 80.1, 68.0, 60.1, 49.4, 41.7, 40.1, 31.1, 30.3, 25.7, 24.1, 21.6. **EI-MS:** Calculated for $C_{25}H_{31}NO_3S$: 425.2025, Found 425.2015.

3. Crystallographic Data

Crystallographic data were collected at 193(2) K on a Bruker-AXS Kappa APEX II Quazar diffractometer equipped with a 30W air-cooled microfocus source (**5c**, **5d**, **5e**) or on a Bruker-AXS D8-Venture equipped with a CMOS Area detector (**3a**, **5b**), using Mo K_α radiation (λ=0.71073 Å). Phi- and omega-scans were used. Space groups were determined on the basis of systematic absences and intensity statistics. Empirical absorption correction was employed.[18] The structures were solved using an intrinsic phasing method (SHELXT),[19] and refined using the least-squares method on F^2.[20] All non-H atoms were refined with anisotropic displacement parameters. Hydrogen atoms were refined isotropically at calculated positions using a riding model with their isotropic displacement parameters constrained to be equal to 1.5 times the equivalent isotropic displacement parameters of their pivot atoms for terminal sp^3 carbon and 1.2 times for all other carbon atoms. For 5e, the solvent the solvent molecule was disordered and difficult to model

correctly. Therefore the SQUEEZE function of PLATON[21] was used to eliminate the contribution of the electron density in the solvent region from the intensity data, and a solvent-free model was used for the final refinement. And for **5c**, the anisotropic displacements parameters and the residual electron density suggested a disorder of the gold atom, chlorine atom and carbon atoms (C1 to C8) over two positions in a ratio of 87:13 after refinement.

CCDC 2069847 (**3a**), 2069844 (**5b**), 2069846 (**5c**), 2069845 (**5d**) and 2069848 (**5e**) contain the supplementary crystallographic data for this paper. These data can be obtained free of charge from The Cambridge Crystallographic Data Centre via www.ccdc.cam.a-c.uk/data_request/cif.

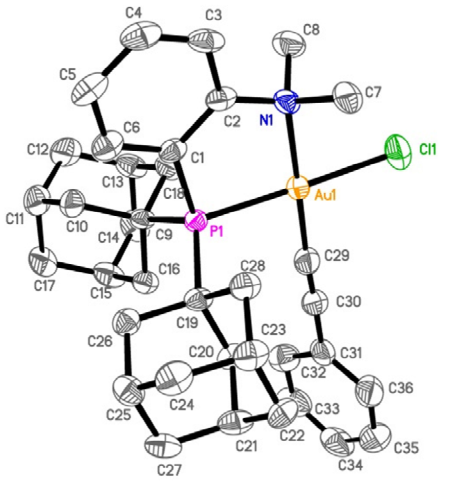

Molecular structure of 3a. Hydrogen atoms, counter anion are omitted for clarity; thermal ellipsoids are drawn with 50% probability. Selected bond lengths (Å) and angles (°): Au–P 2.289(2), Au–N 2.121(8), Au–Cl 2.337(3), Au–C29 1.971(11), P–Au–N 87.1(2).

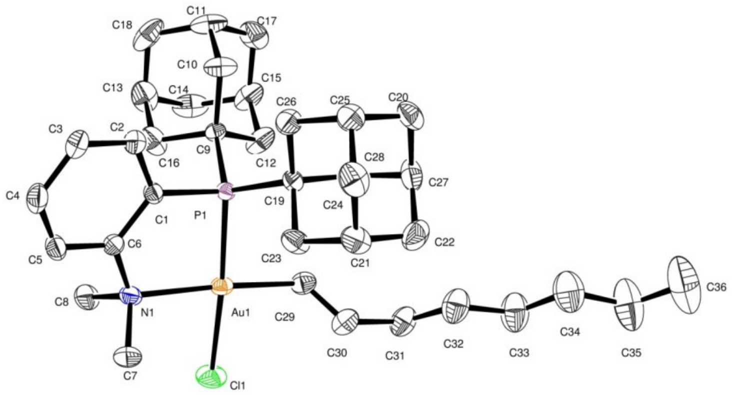

Molecular structure of 5b. Hydrogen atoms, counter anion and solvent molecule are omitted for clarity; thermal ellipsoids are drawn with 50% probability. Selected bond lengths (Å) and angles (°): Au–P 2.311(1), Au–N 2.213(2), Au–Cl 2.336(1), Au–C29 2.031(3), P–Au–N 85.84(6).

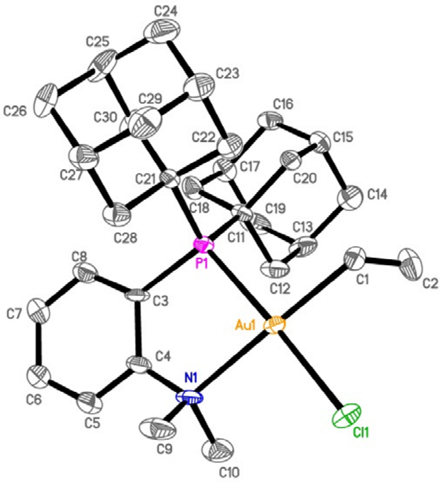

Molecular structure of 5c. Hydrogen atoms, counter anion and solvent molecule are omitted for clarity; thermal ellipsoids are drawn with 50% probability. Selected bond lengths (Å) and angles (°): Au–P 2.333(2), Au–N 2.268(6), Au–Cl 2.342(3), Au–C1 2.039(11), P–Au–N 84.18(16). Au1'–P 2.435(4), Au1'–N 2.323(6), Au1'–Cl1' 2.308(9), Au1'–C1' 2.015(14), P–Au1'–N 80.78(19).

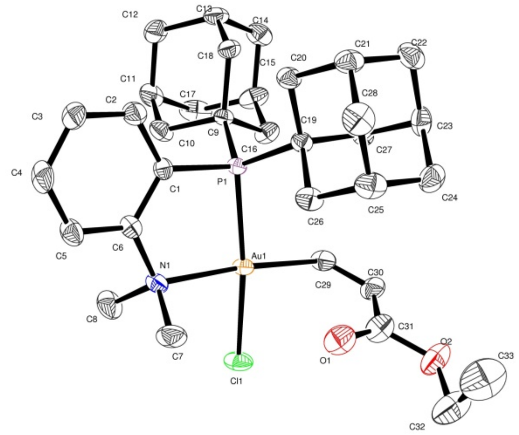

Molecular structure of 5d. Hydrogen atoms, counter anion and disordered atoms are omitted for clarity; thermal ellipsoids are drawn with 50% probability. Selected bond lengths (Å) and angles (°): Au–P 2.3152(9), Au–N 2.173(3), Au–Cl 2.355(1), Au–C29 2.020(4), P–Au–N 85.87(8).

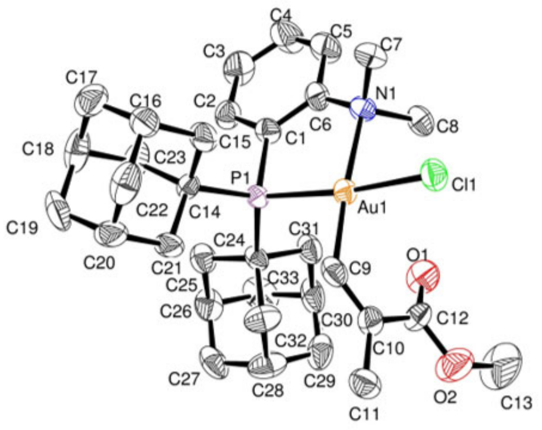

Molecular structure of 5e. Hydrogen atoms and counter anion are omitted for clarity; thermal ellipsoids are drawn with 50% probability. Selected bond lengths (Å) and angles (°): Au–P 2.319(2), Au–N 2.186(6), Au–Cl 2.361(2), Au–C9 1.998(8), P–Au–N 86.29(18).

Table S1. Crystal Data, Data Collection, and Structure Refinement for **3a** and **5b-e**.

ID	**3a**	**5b**	**5c**	**5d**	**5e**
formula	$C_{36}H_{47}AuClNP$, Sb F_6	$C_{36}H_{55}AuClNP$, SbF_6, CH_2Cl_2	$C_{30}H_{43}AuCl_2NP$, SbF_6	$C_{33}H_{47}AuClNO_2P$, SbF_6	$C_{33}H_{47}AuClNO_2P$, SbF_6
M_r	990.86	1085.89	959.25	988.87	988.87
crystal system	monoclinic	triclinic	triclinic	monoclinic	triclinic
space group	*C2/c*	$P\bar{1}$	$P\bar{1}$	$P\,2_1/n$	$P\bar{1}$
a (Å)	29.910(4)	10.1636(12)	10.3795(8)	9.7524(11)	10.1052(9)
b (Å)	9.8604(14)	11.2723(15)	11.7433(10)	25.856(3)	14.6709(12)
c (Å)	28.710(4)	20.036(5)	16.0874(13)	14.5514(17)	14.9416(12)
α (°)	90	79.592(8)	80.093(3)	90	109.750(3)
β (°)	119.225(4)	86.479(7)	77.477(3)	106.770(3)	101.005(3)
γ (°)	90	65.966(4)	65.482(3)	90	104.616()
V (Å^3)	7389.5(17)	2061.8(6)	1734.2(2)	3513.2(7)	1922.4(3)
Z	8	2	2	4	2
ρ_{calc} (g cm^{-3})	1.781	1.749	1.837	1.870	1.708
μ (mm^{-1})	4.869	4.496	5.258	5.125	4.683
F(000)	3872	1072	934	1936	968
crystal size (mm^3)	0.16 x 0.12 x 0.06	0.14 x 0.12 x 0.06	0.12 x 0.10 x 0.08	0.18 x 0.04 x 0.04	0.2 x 0.04 x 0.02
T/K	193(2)	193(2)	193(2)	193(2)	193(2)
measd reflns	80974	80664	50848	152578	50261
Unique reflns (Rint)	7548 (0.0544)	15578 (0.0509)	7049 (0.0883)	14636 (0.1249)	8537 (0.1011)
Data/restraints/parameters	7548 / 255 / 523	15578/79/483	7049/569/543	14636/77/448	8537/231/483
GOF on F^2	1.257	1.016	1.091	1.030	1.052
R_1[a] [I>2σ(I)]	0.0573	0.0329	0.0582	0.0395	0.0525
wR_2[b] [all data]	0.1384	0.0758	0.1531	0.0805	0.1385

[a] $R_1 = \Sigma||F_o| - |F_c|| / \Sigma |F_o|$. [b] $wR_2 = [\Sigma[w(F_o^2 - F_c^2)^2] / \Sigma [w(F_o^2)^2]]^{1/2}$.

4. References

[1] J. A. Bull, J. J. Mousseau and A. B. Charette *Org. Lett.*, 2008, **10**, 5485.
[2] R. Dakarapu and J. R. Falck *J. Org. Chem.*, 2018, **83**, 1241.
[3] J. A. Cadge, H. A. Sparkes, J. Bower, C. A. Russell, *Angew. Chem., Int. Ed*. 2020, **59**, 6617.
[4] K. Kepski, C. R. Rice and W. J. Moran *Org. Lett.*, 2019, **21**, 6936.
[5] M. J. Doty, X. Han and R. C. Larock, *J. Org. Chem.*, 1999, **64**, 8770.
[6] H. Ren, A. Krasovskiy and P. Knochel *Org. Lett.*, 2004, **6**, 4215.
[7] C. Gao, S. Nakao and S. A. Blum *J. Org. Chem.*, 2020, **85**, 10350.
[8] S. Fujita, M. Abe, M. Shibuya and Y. Yamamoto, *Org. Lett.*, 2015, **17**, 3822.
[9] G. Zhang, L. Cui, Y. Wang and L. Zhang, *J. Am. Chem. Soc.*, 2010, **132**, 1474.
[10] Y.-S. Hon, Y.-W. Liu and C.-H. Hsieh, *Tetrahedron* 2004, **60**, 4837.
[11] M. Rigoulet, O. Thillaye du Boullay, A. Amgoune, D. Bourissou, *Angew. Chem. Int. Ed.*, 2020, **59**, 16625.
[12] A. Verma, S. Jana, C. D. Prasad, A. Yadav and S. Kumar, *Chem. Commun.*, 2016, **52**, 4179.
[13] T. Saito, Y. Nishimoto, M. Yasuda and A. Baba *J. Org. Chem.*, 2006, **71**, 8516.
[14] M. B. Hay, A. R. Hardin and J. P. Wolfe *J. Org. Chem.*, 2005, **70**, 3099.
[15] Z. Zhang, X. Tang, C. S. Thomoson and W. R. Dolbier, *Org. Lett.*, 2015, **17**, 3528.
[16] J. Rodriguez, N. Adet, N. Saffon-Merceron, D. Bourissou, *Chem. Commun*., 2020, **56**, 94.
[17] J. Rodriguez, A. Zeineddine, E. D. Sosa Carrizo, K. Miqueu, N. Saffon-Merceron, A. Amgoune, D. Bourissou, *Chem. Sci*., 2019, **10**, 7183.
[18] Bruker, *SADABS*, Bruker AXS Inc., Madison, Wisconsin, USA, 2008.
[19] ShelXT, G. M. Sheldrick, University of Göttingen, *Acta Crystallogr. Sect. A*, 2015, **71**, 3.
[20] ShelXL, G. M. Sheldrick, University of Göttingen, *Acta Crystallogr. Sect. C*, 2015, **71**, 3.
[21] A. L. Spek, *Acta Crystallogr. Sect. C*, 2015, **71**, 9.

5. NMR spectra

5.1 NMR spectra of complexes

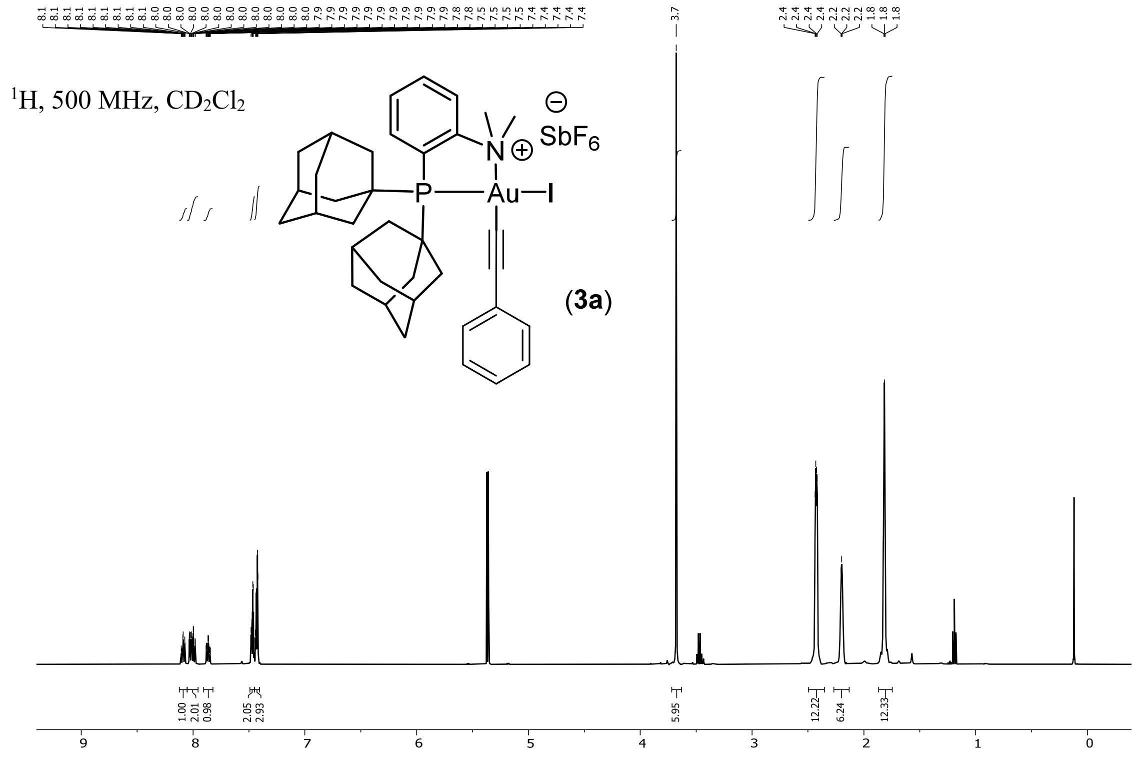

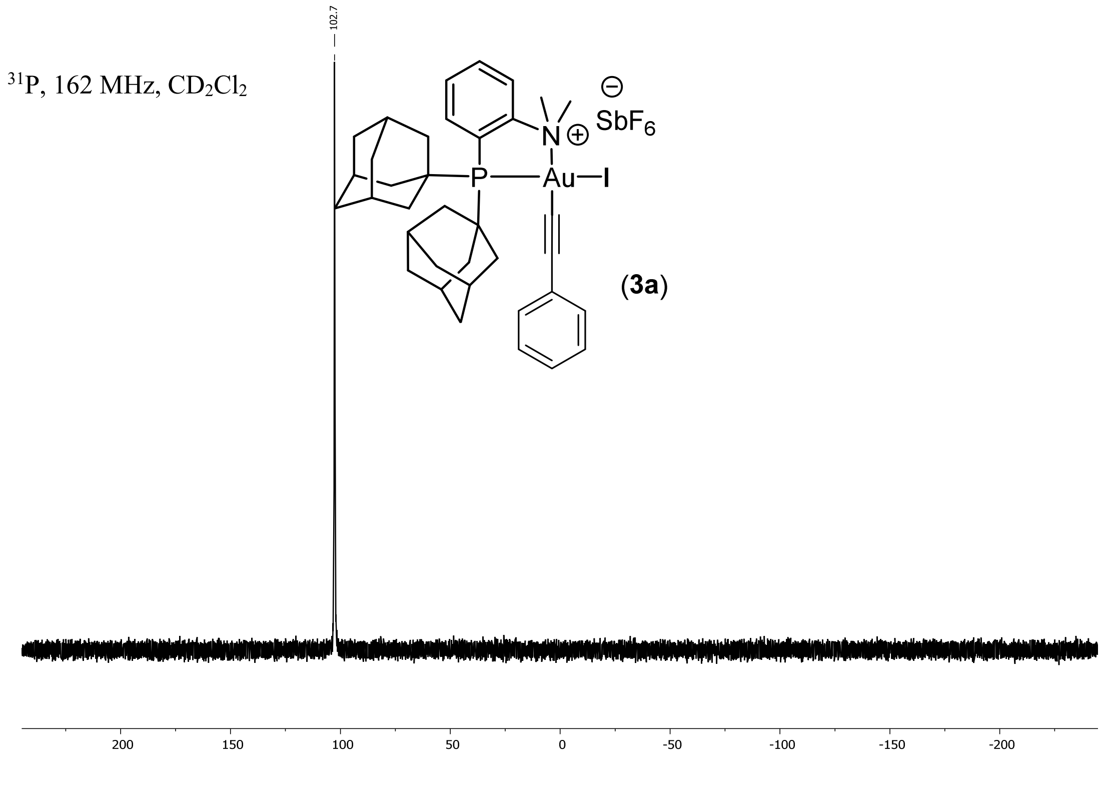

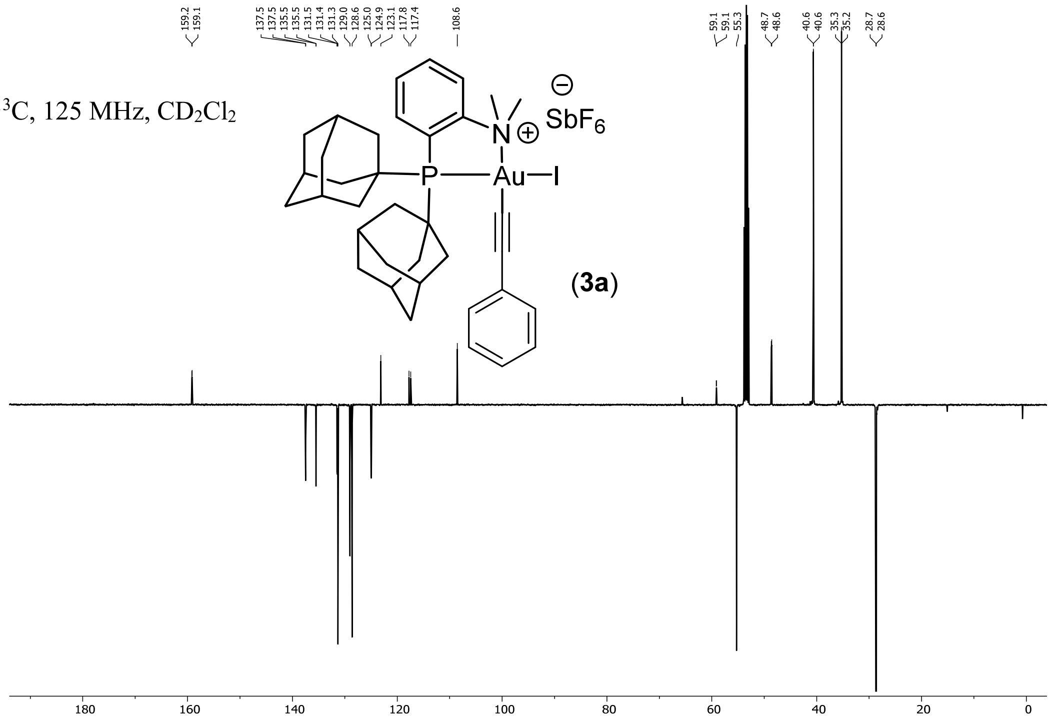
^{13}C, 125 MHz, CD_2Cl_2
159.2
159.1
137.5
137.5
135.5
135.5
131.5
131.4
131.3
129.0
128.6
125.0
124.9
123.1
117.8
117.4
108.6
59.1
59.1
55.3
48.7
48.6
40.6
40.6
35.3
35.2
28.7
28.6
SbF6
P
Au
I
N
(3a)

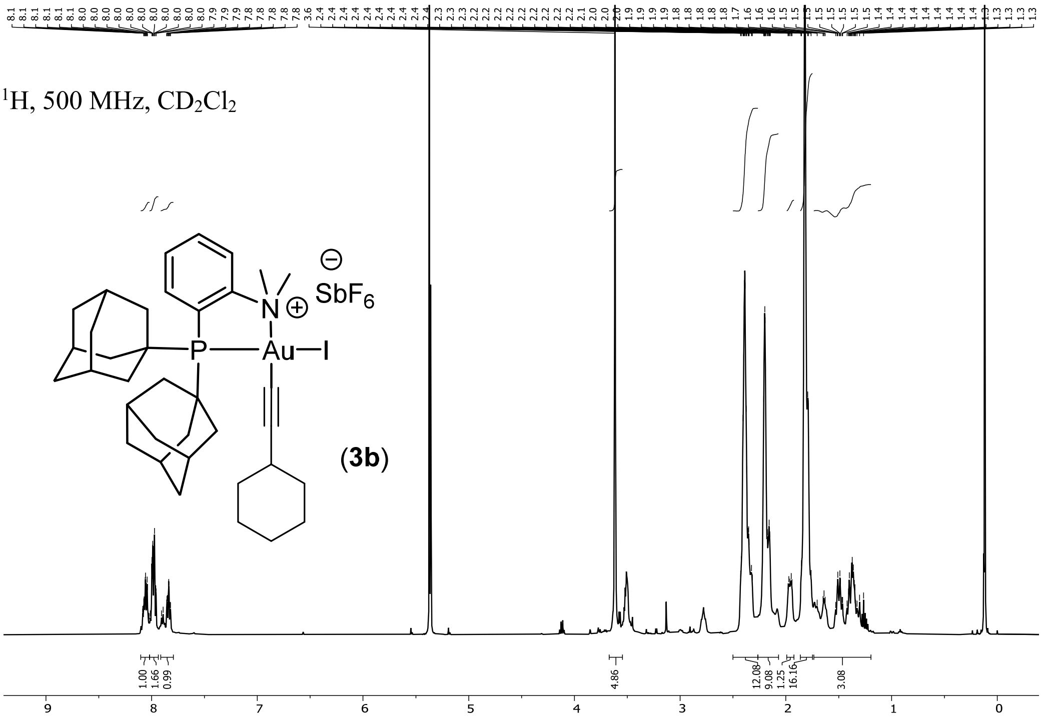
^1H, 500 MHz, CD_2Cl_2
SbF6
P
Au
I
N
(3b)
1.00
1.66
0.99
4.86
12.08
9.08
1.25
16.16
3.08

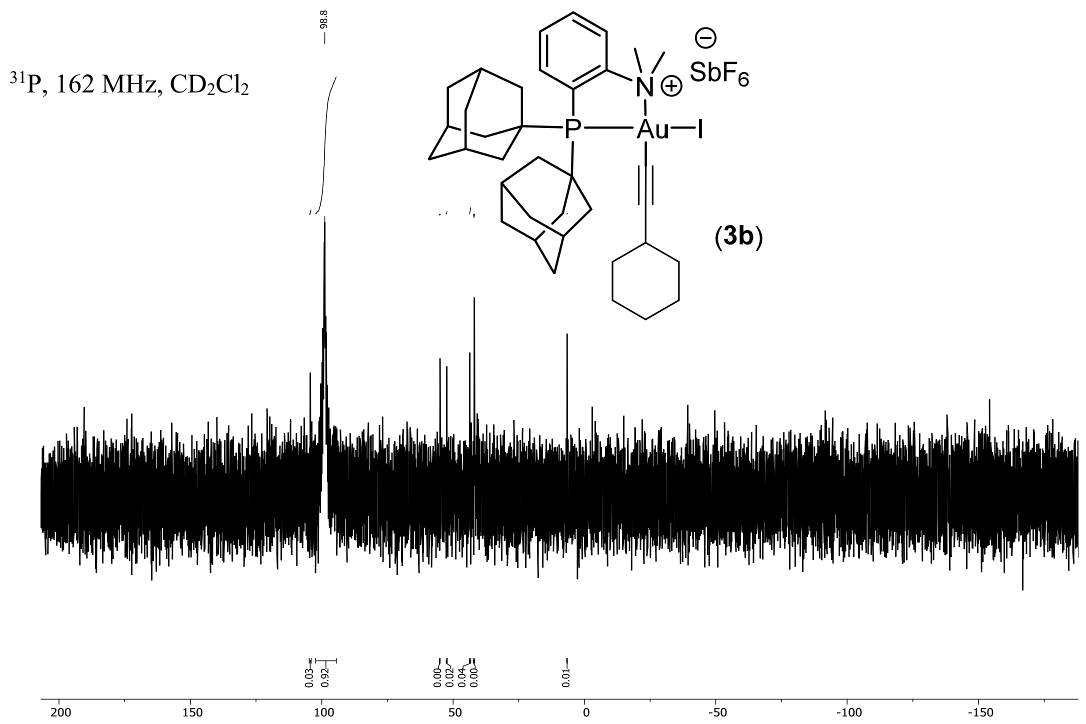

31P, 162 MHz, CD2Cl2
98.8
SbF6
N
P
Au
I
(3b)
0.03
0.92
0.00
0.02
0.04
0.00
0.01
200
150
100
50
0
-50
-100
-150

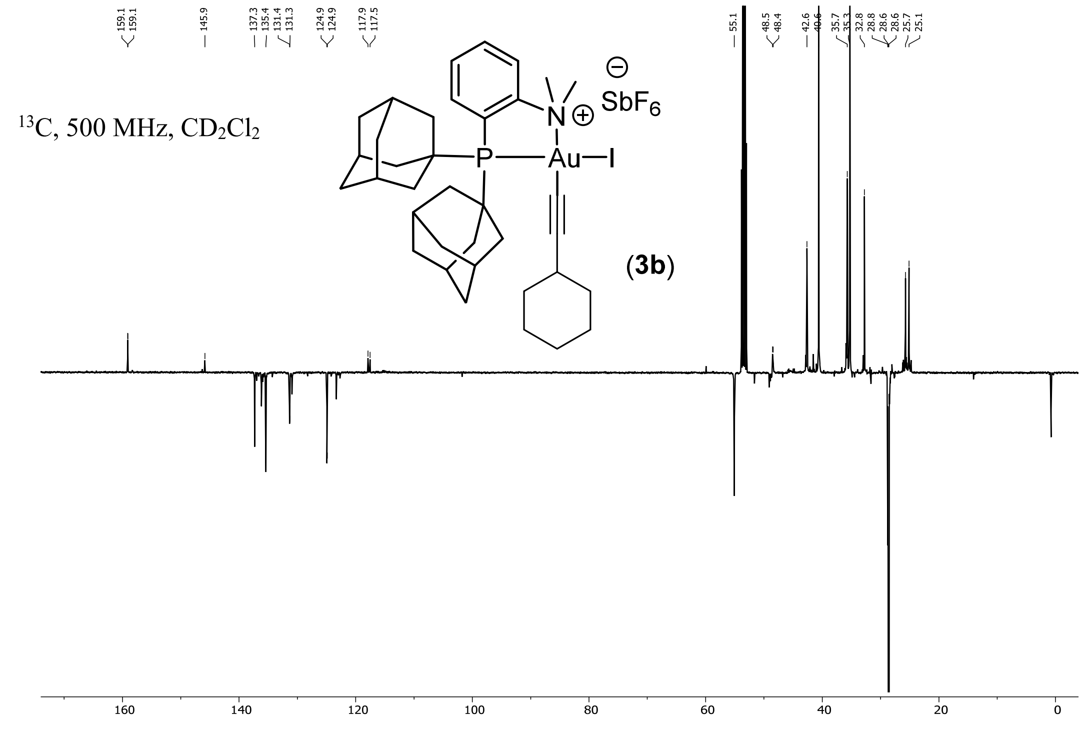

13C, 500 MHz, CD2Cl2
159.1
159.1
145.9
137.3
135.4
131.4
131.3
124.9
124.9
117.9
117.5
55.1
48.5
48.4
42.6
40.6
35.7
35.3
32.8
28.8
28.6
28.6
25.7
25.1
SbF6
N
P
Au
I
(3b)
160
140
120
100
80
60
40
20
0

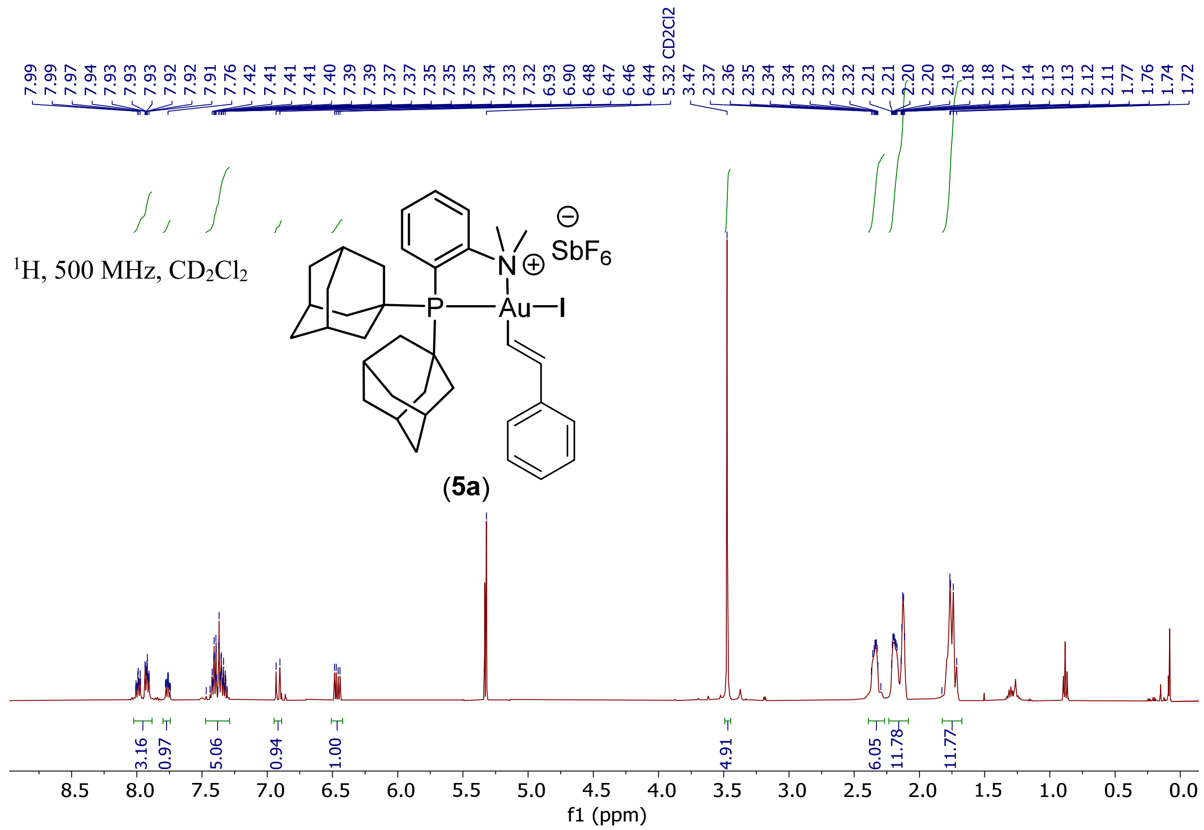
7.99 7.99 7.97 7.94 7.93 7.93 7.93 7.92 7.92 7.91 7.76 7.42 7.41 7.41 7.41 7.40 7.39 7.39 7.37 7.37 7.35 7.35 7.35 7.34 7.33 7.32 6.93 6.90 6.48 6.47 6.46 6.44 5.32 CD2Cl2 3.47 2.37 2.36 2.35 2.34 2.34 2.33 2.32 2.32 2.21 2.21 2.20 2.20 2.19 2.18 2.18 2.17 2.14 2.13 2.13 2.12 2.11 1.77 1.76 1.74 1.72
^{1}H, 500 MHz, CD2Cl2
SbF6
N
P
Au
I
(5a)
3.16
0.97
5.06
0.94
1.00
4.91
6.05
11.78
11.77
f1 (ppm)

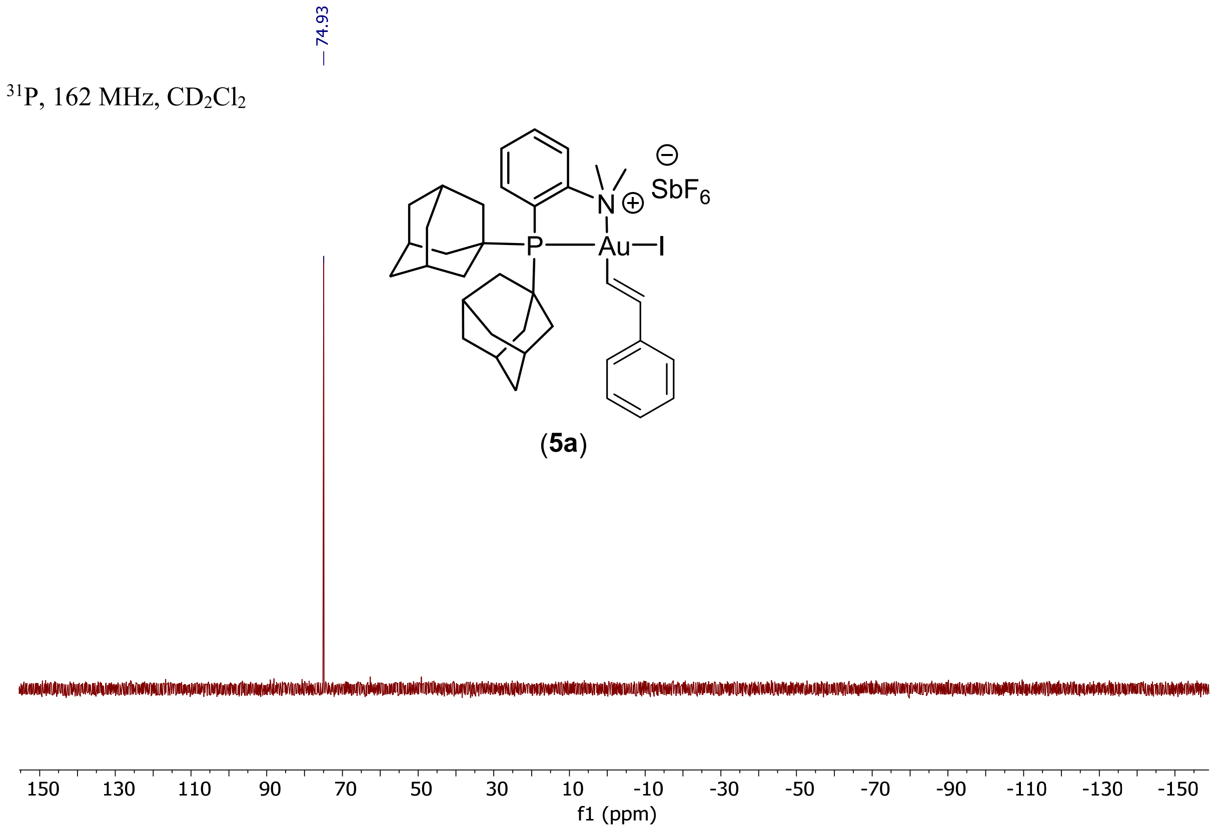
74.93
^{31}P, 162 MHz, CD2Cl2
SbF6
N
P
Au
I
(5a)
f1 (ppm)

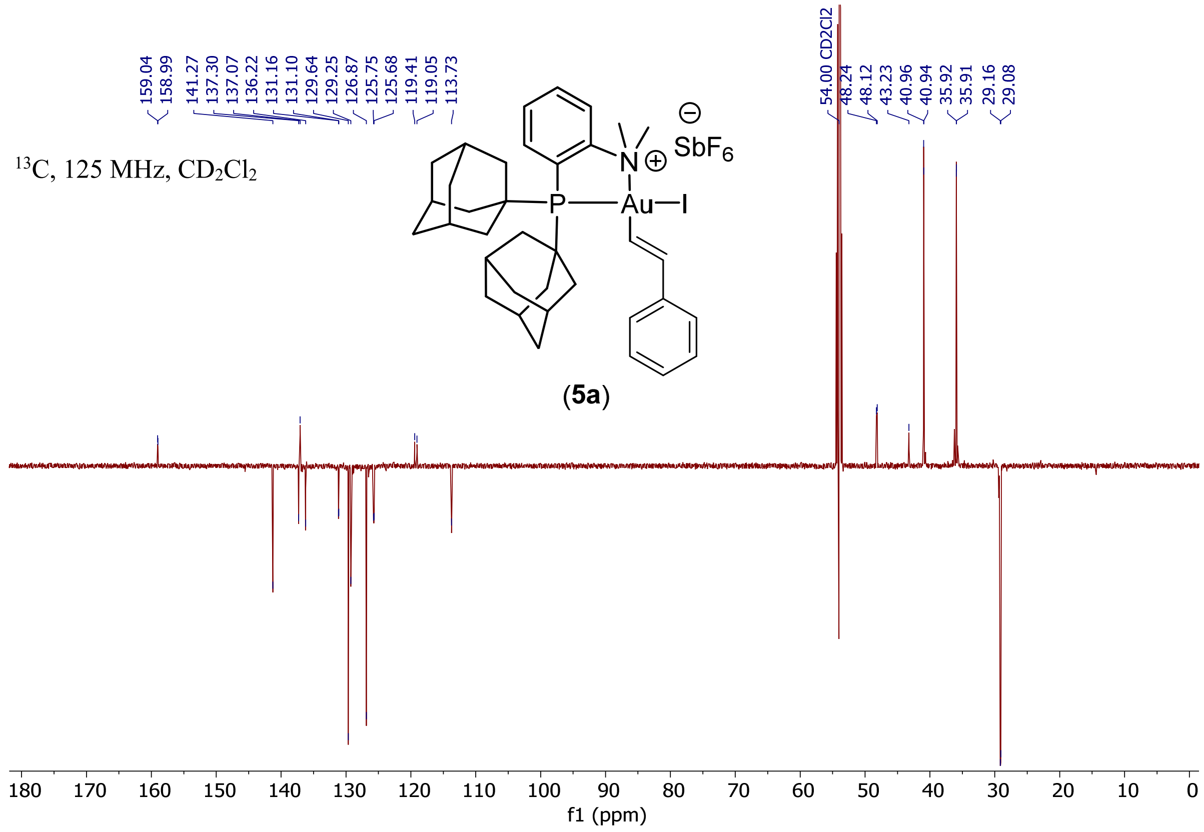

^{13}C, 125 MHz, CD_2Cl_2
159.04
158.99
141.27
137.30
137.07
136.22
131.16
131.10
129.64
129.25
126.87
125.75
125.68
119.41
119.05
113.73
54.00 CD2Cl2
48.24
48.12
43.23
40.96
40.94
35.92
35.91
29.16
29.08
N
SbF6
P
Au
I
(5a)
180 170 160 150 140 130 120 110 100 90 80 70 60 50 40 30 20 10 0
f1 (ppm)

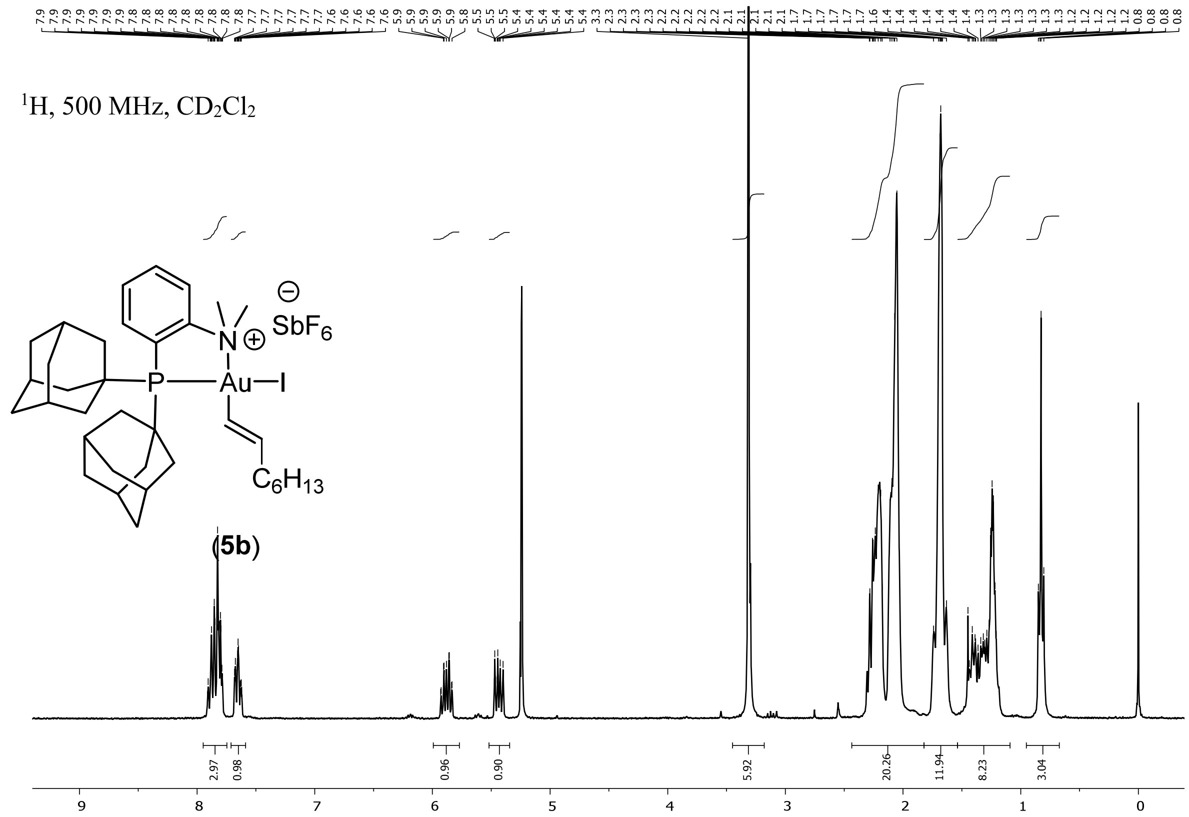

^{1}H, 500 MHz, CD_2Cl_2
N
SbF6
P
Au
I
C6H13
(5b)
2.97
0.98
0.96
0.90
5.92
20.26
11.94
8.23
3.04
9 8 7 6 5 4 3 2 1 0

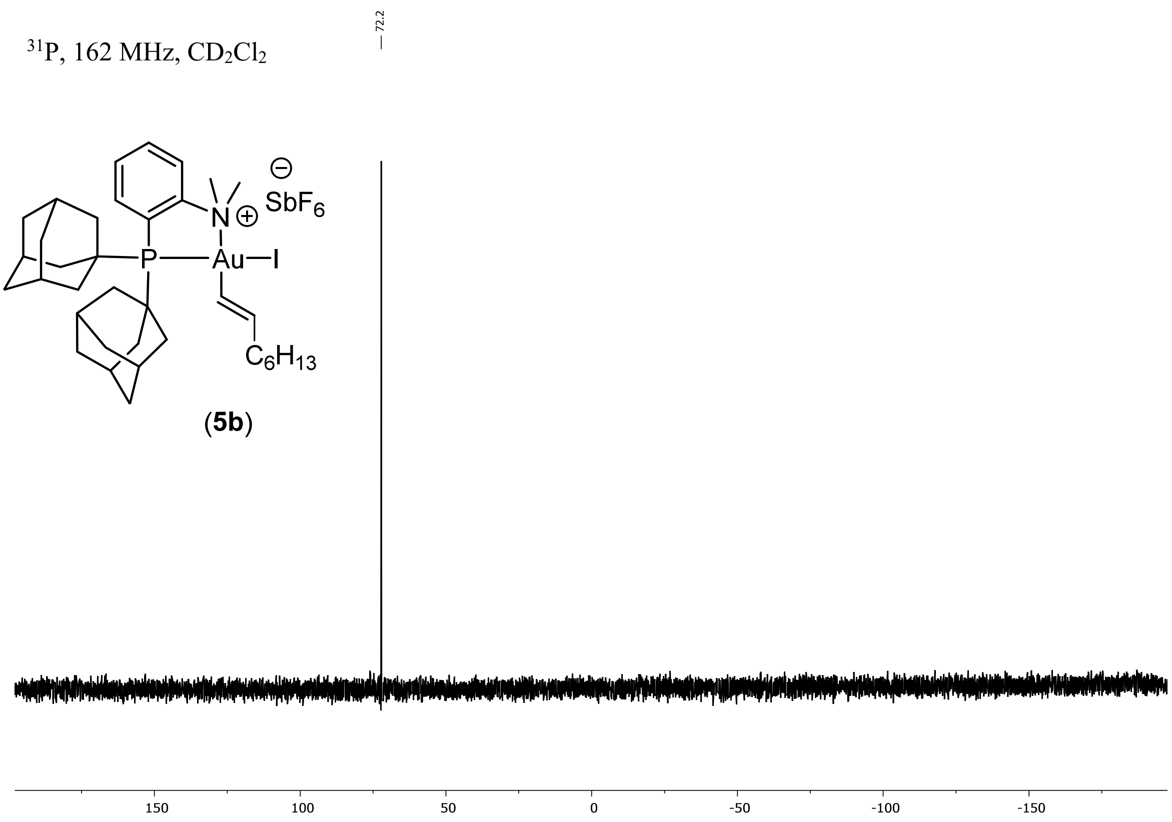

^{31}P, 162 MHz, CD_2Cl_2
72.2
SbF_6
N
P
Au
I
C_6H_{13}
(**5b**)
150
100
50
0
-50
-100
-150

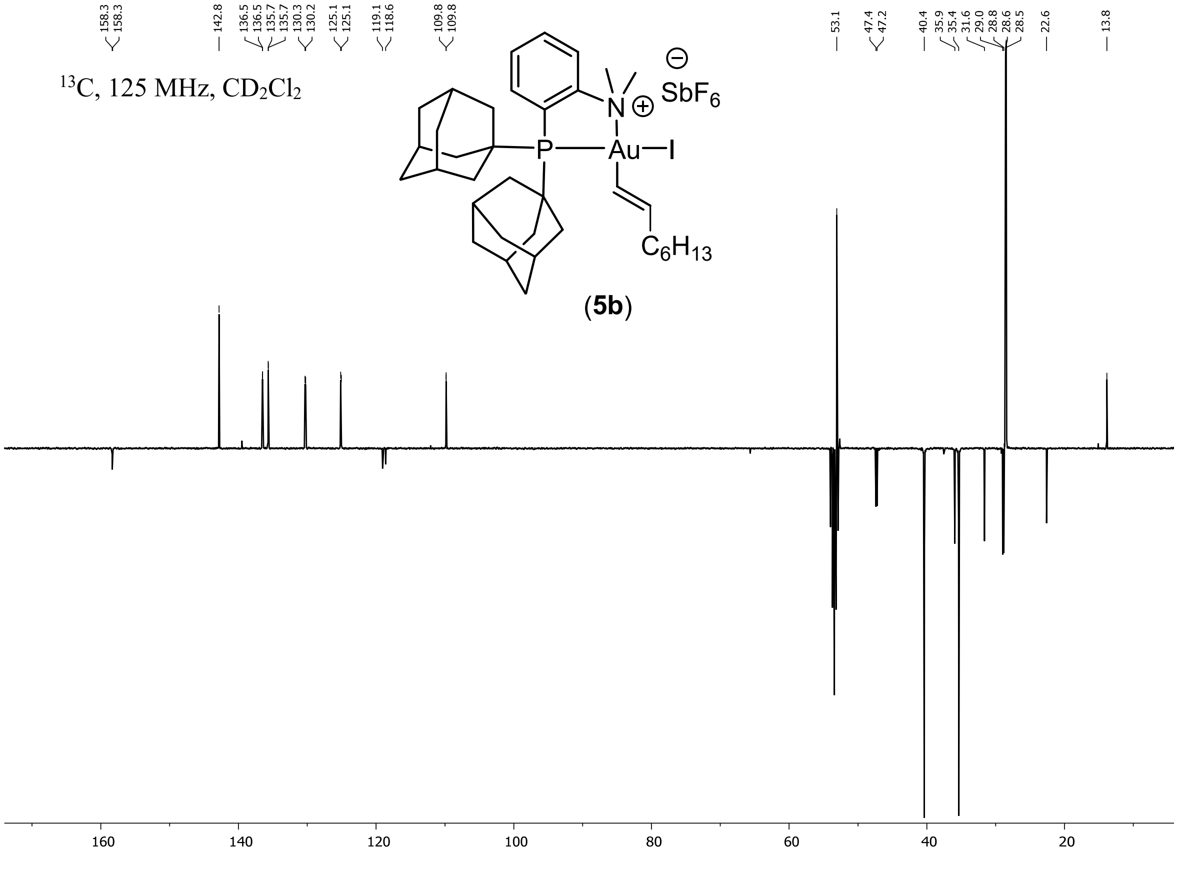

^{13}C, 125 MHz, CD_2Cl_2
158.3
158.3
142.8
136.5
136.5
135.7
135.7
130.3
130.2
125.1
125.1
119.1
118.6
109.8
109.8
53.1
47.4
47.2
40.4
35.9
35.4
31.6
29.0
28.8
28.6
28.5
22.6
13.8
SbF_6
N
P
Au
I
C_6H_{13}
(**5b**)
160
140
120
100
80
60
40
20

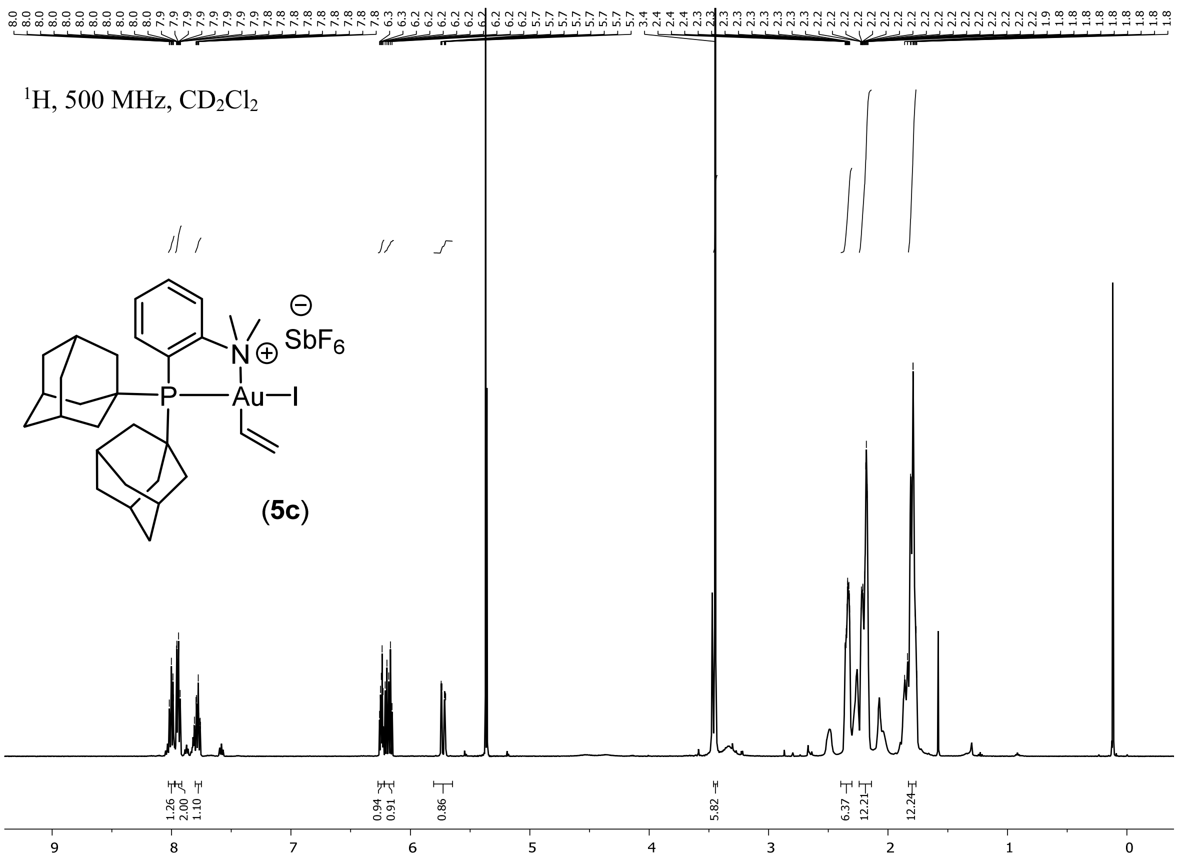

1H, 500 MHz, CD2Cl2
SbF6
N
P
Au
I
(5c)
1.26
2.00
1.10
0.94
0.91
0.86
5.82
6.37
12.21
12.24
9
8
7
6
5
4
3
2
1
0

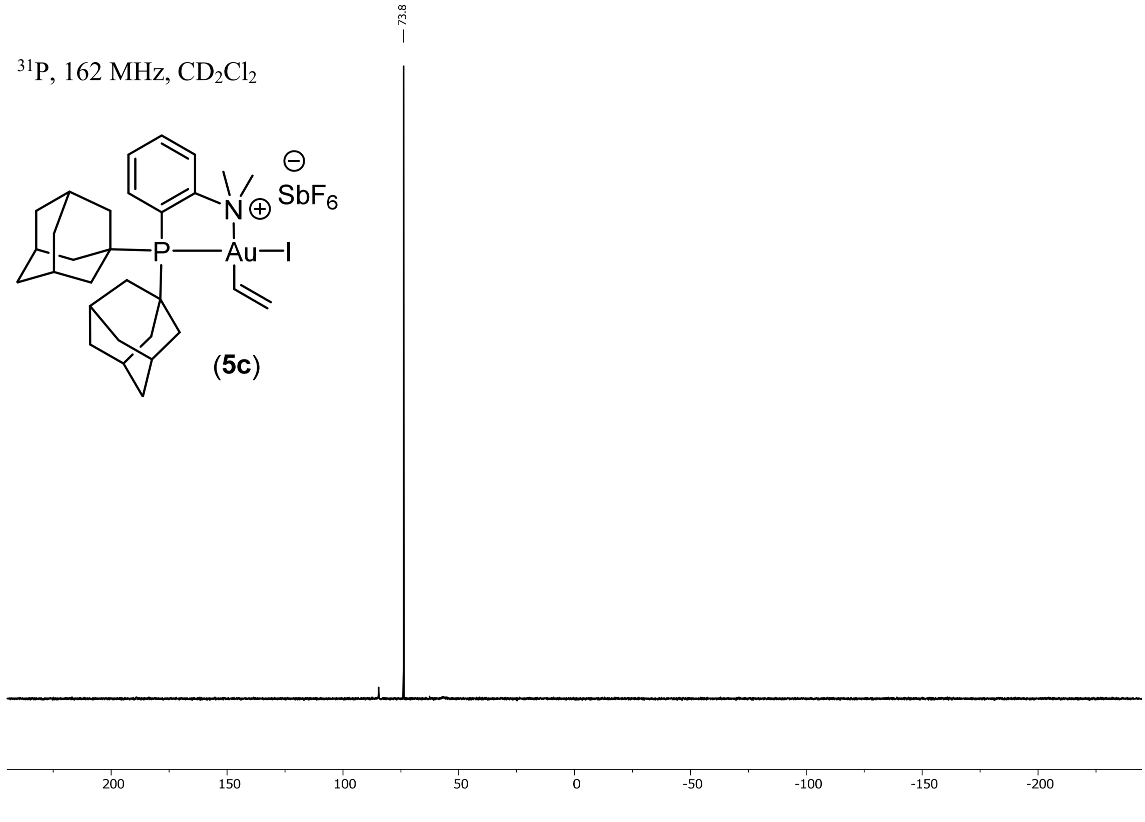

73.8
31P, 162 MHz, CD2Cl2
SbF6
N
P
Au
I
(5c)
200
150
100
50
0
-50
-100
-150
-200

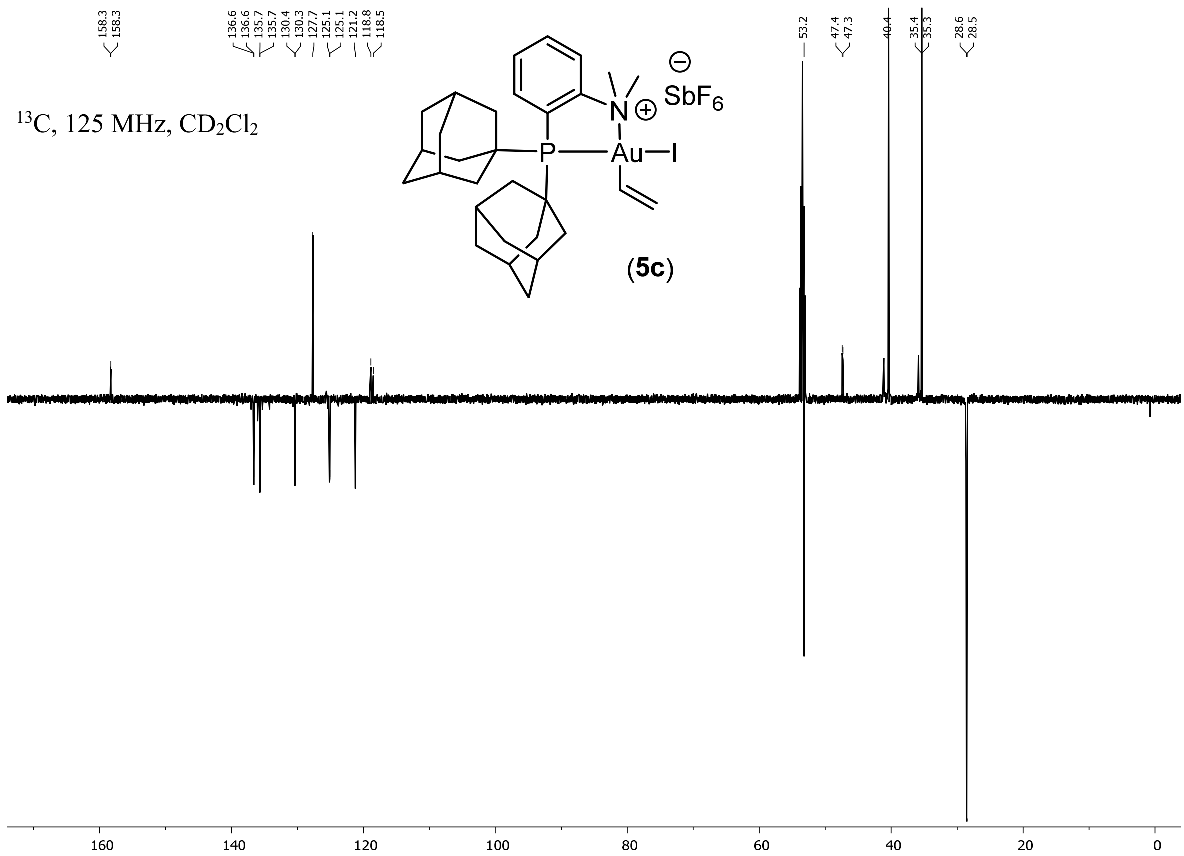
13C, 125 MHz, CD2Cl2
158.3
158.3
136.6
136.6
135.7
135.7
130.4
130.3
127.7
125.1
125.1
121.2
118.8
118.5
53.2
47.4
47.3
40.4
35.4
35.3
28.6
28.5
SbF6
N
P
Au
I
(5c)
160
140
120
100
80
60
40
20
0

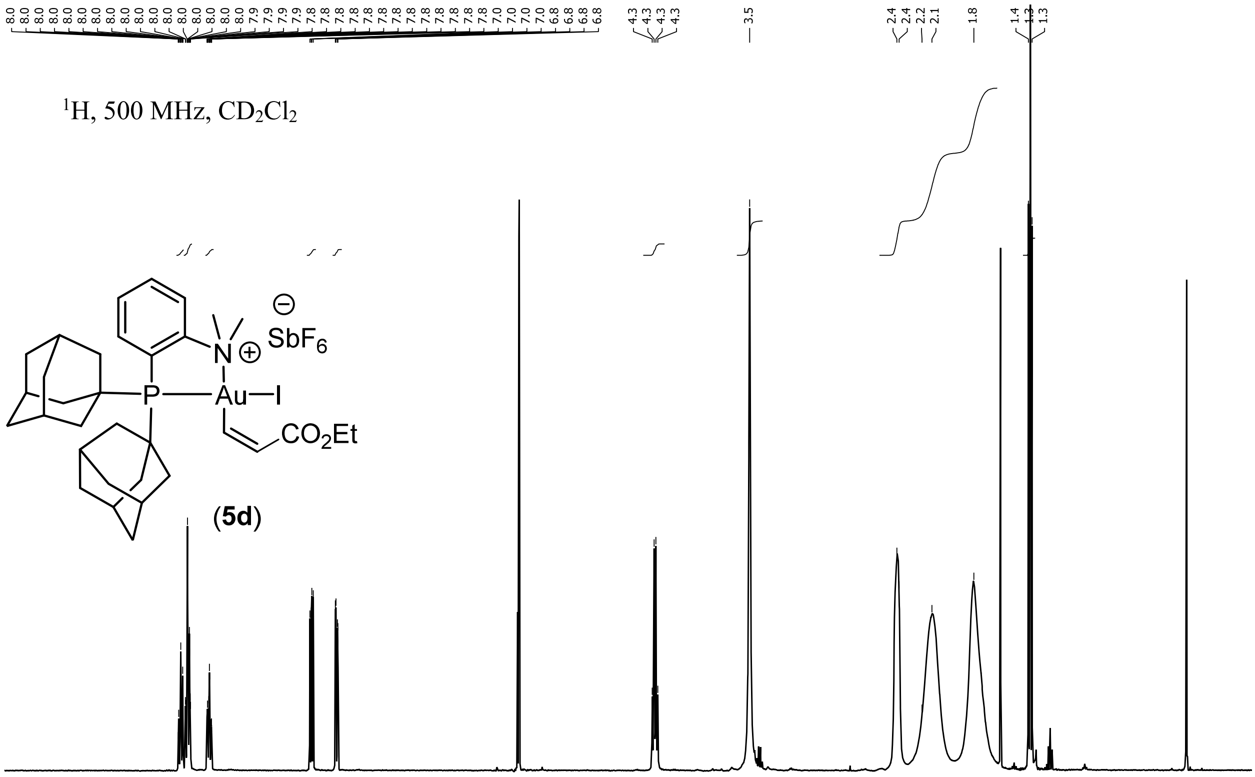
1H, 500 MHz, CD2Cl2
4.3
4.3
4.3
4.3
3.5
2.4
2.4
2.2
2.1
1.8
1.4
1.3
1.3
SbF6
N
P
Au
I
CO2Et
(5d)
0.95
1.95
1.01
1.00
1.01
1.98
5.96
28.97
2.97
9
8
7
6
5
4
3
2
1
0

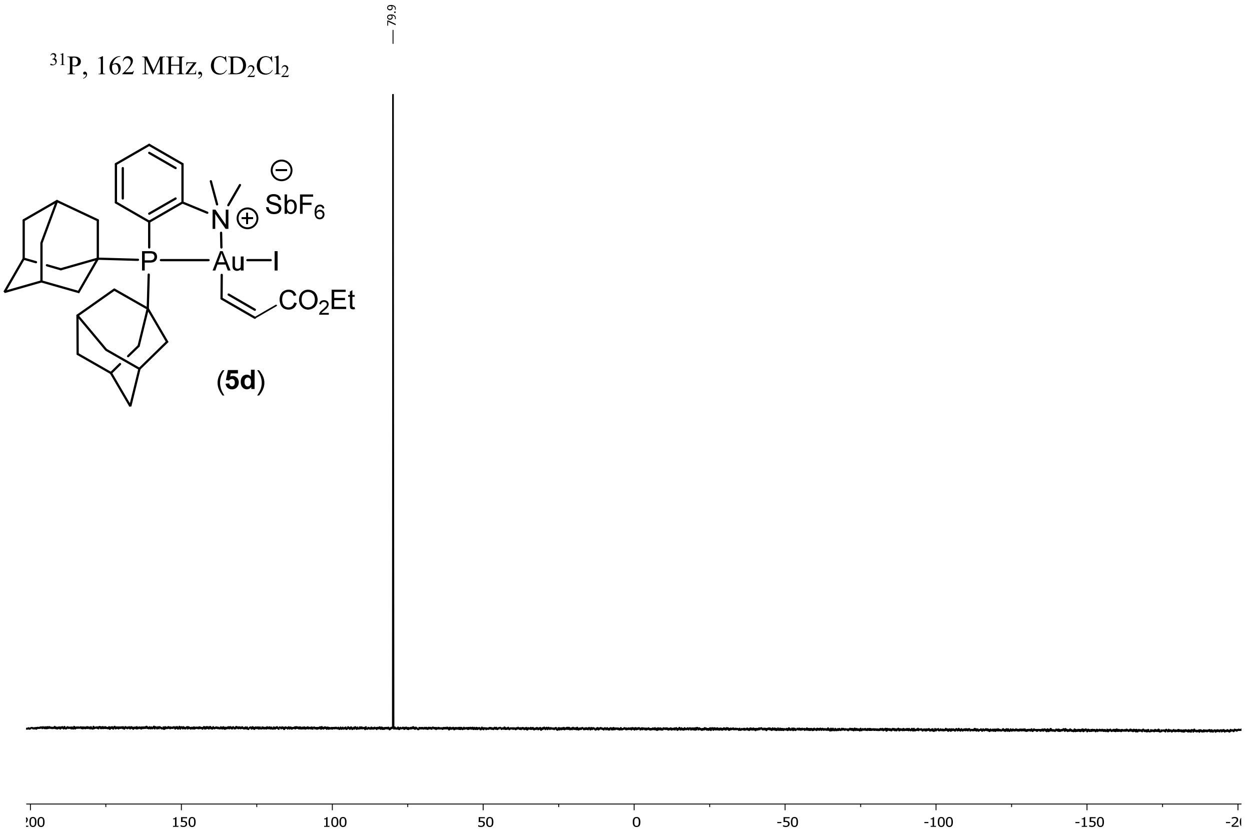
79.9
^{31}P, 162 MHz, CD_2Cl_2
SbF6
N
P
Au—I
CO2Et
(5d)
200
150
100
50
0
-50
-100
-150
-200

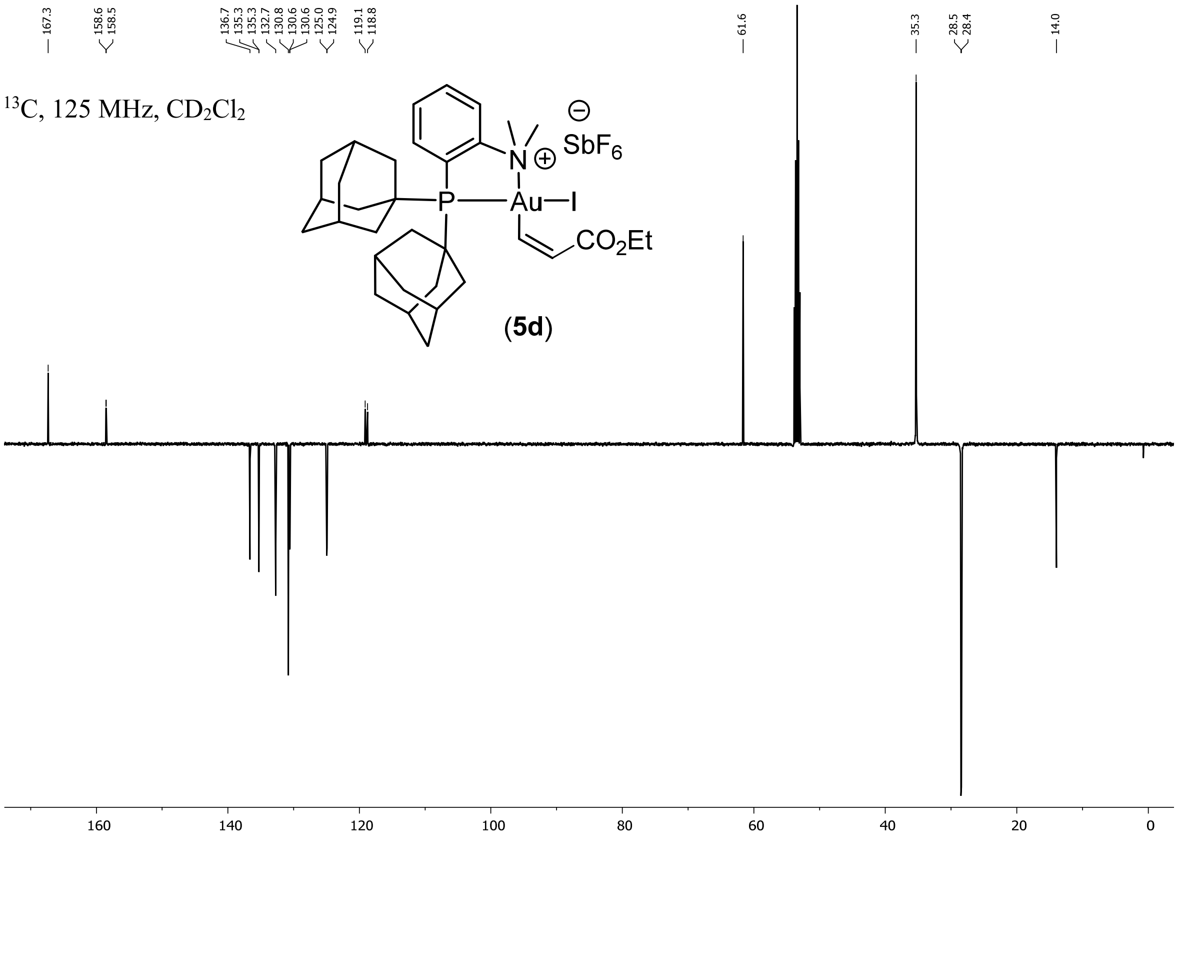
167.3
158.6
158.5
136.7
135.3
135.3
132.7
130.8
130.6
130.6
125.0
124.9
119.1
118.8
61.6
35.3
28.5
28.4
14.0
^{13}C, 125 MHz, CD_2Cl_2
SbF6
N
P
Au—I
CO2Et
(5d)
160
140
120
100
80
60
40
20
0

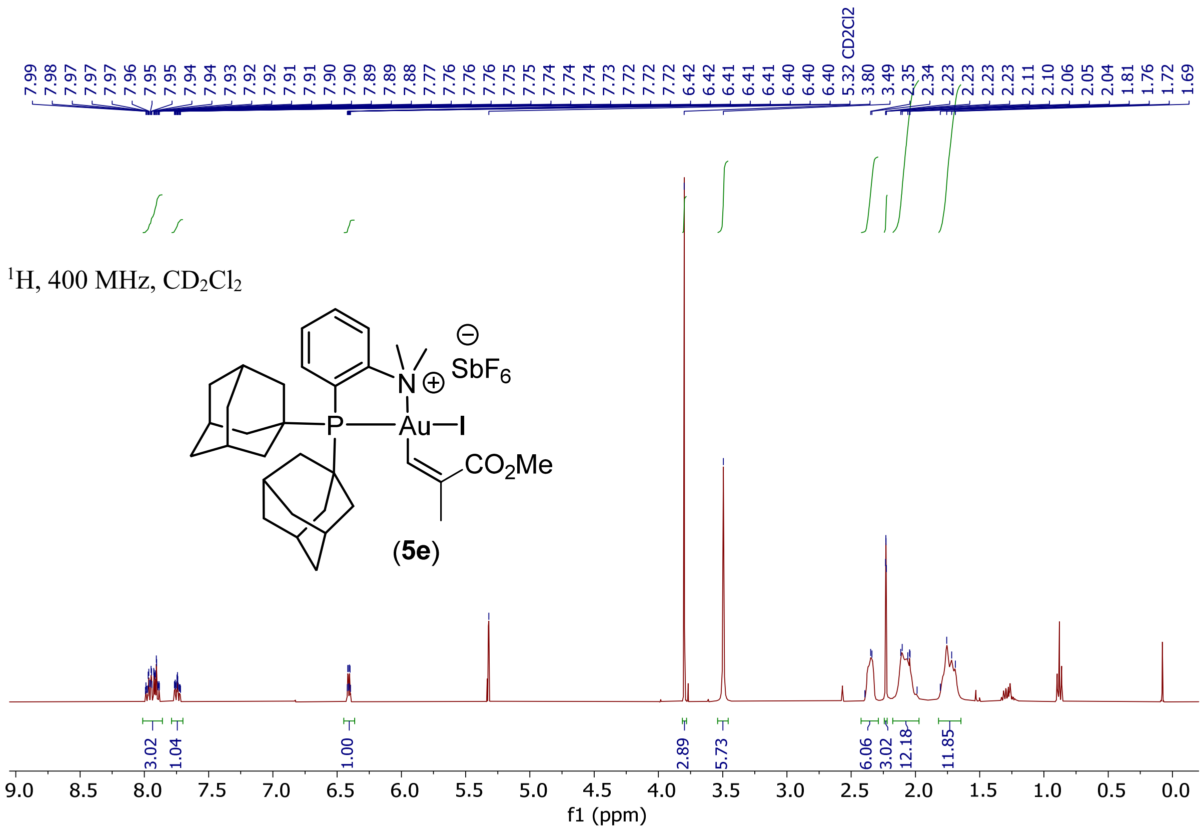

^{1}H, 400 MHz, CD_2Cl_2
SbF6
Au—I
CO2Me
(5e)
f1 (ppm)

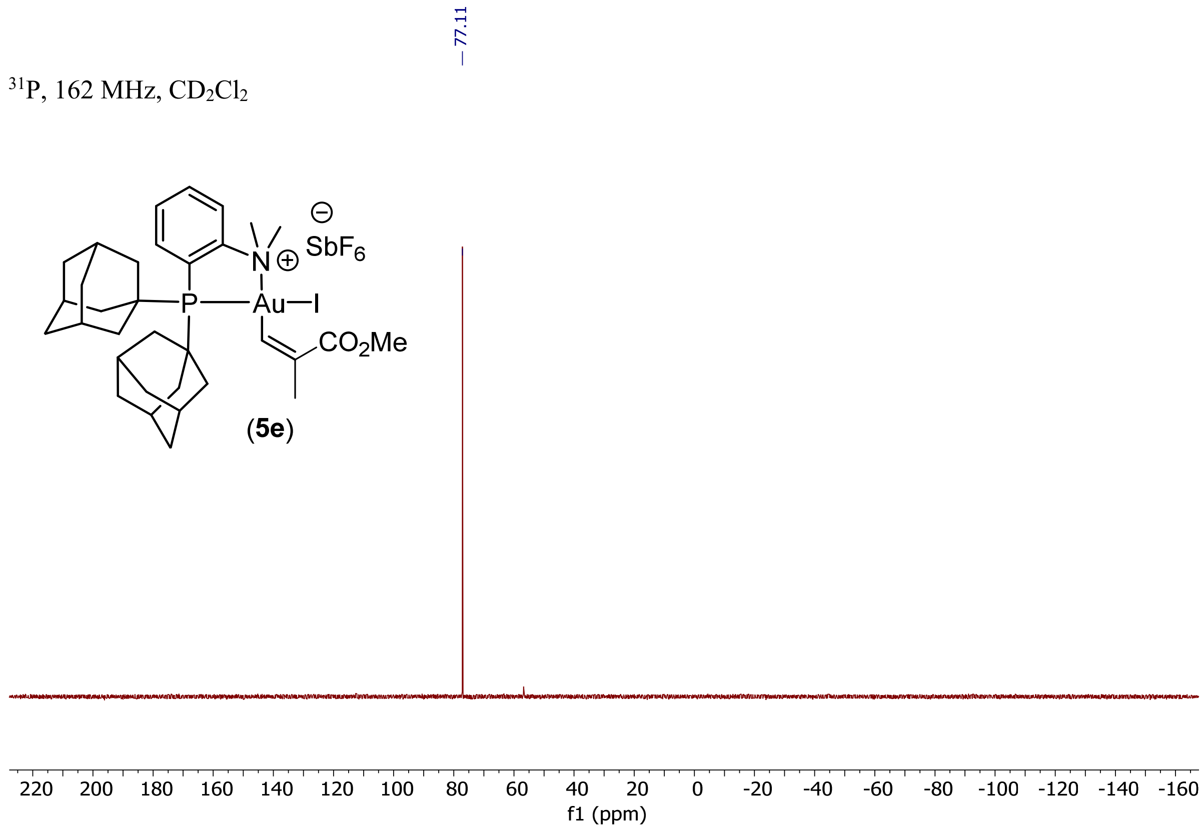

— 77.11
^{31}P, 162 MHz, CD_2Cl_2
SbF6
Au—I
CO2Me
(5e)
f1 (ppm)

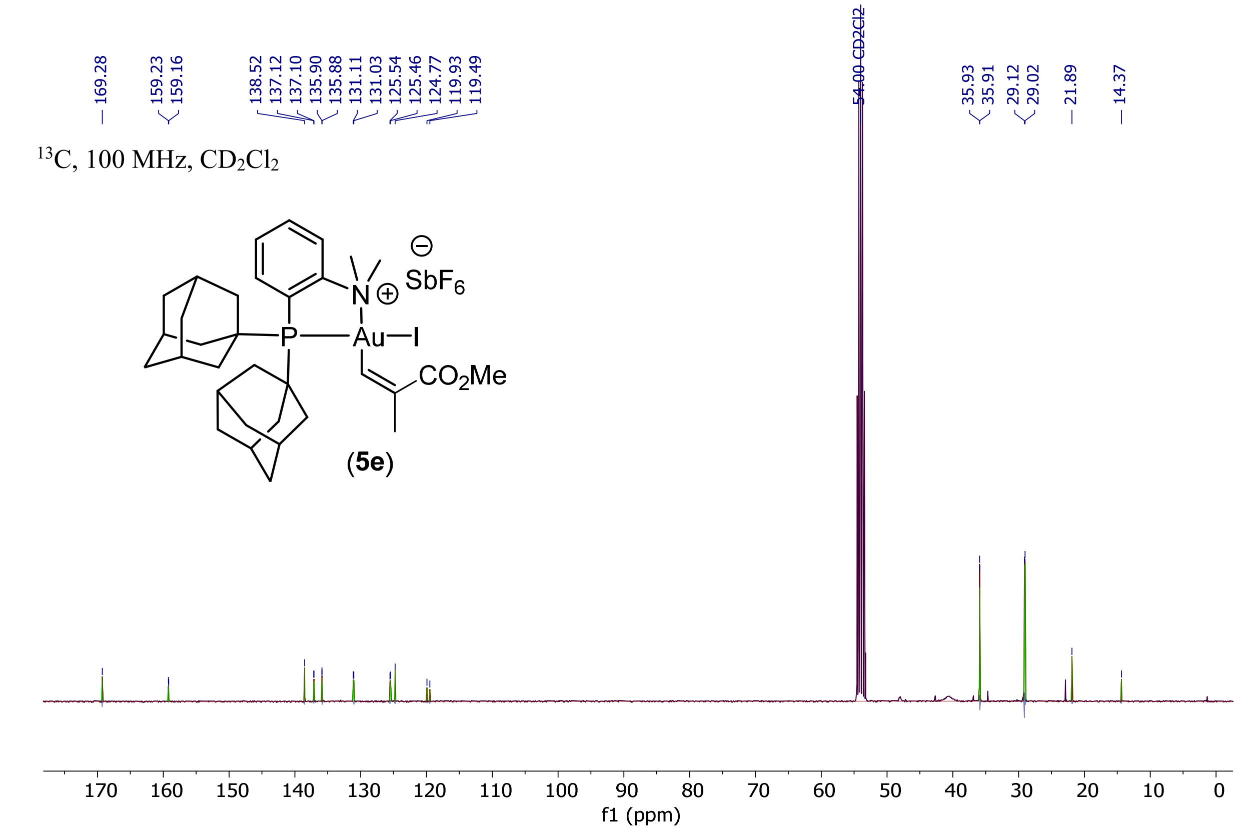

5.2 NMR spectra of vinyl iodides

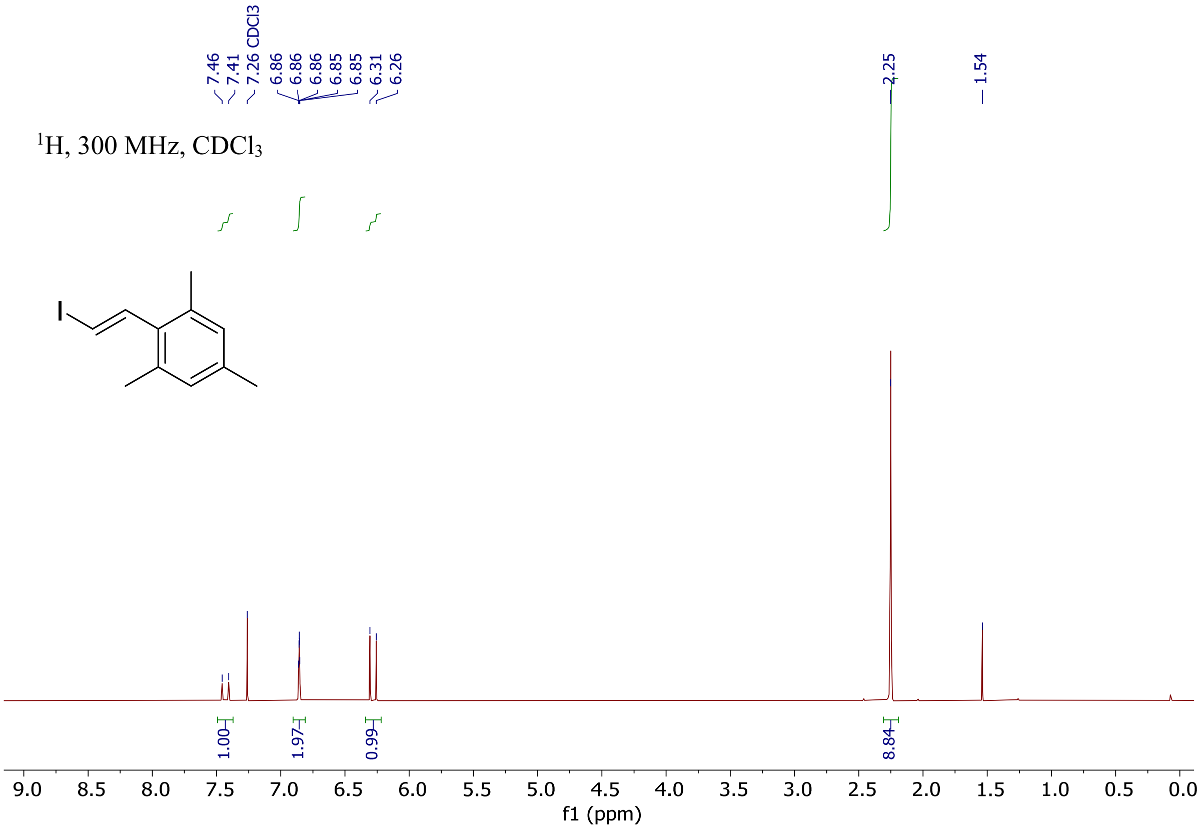

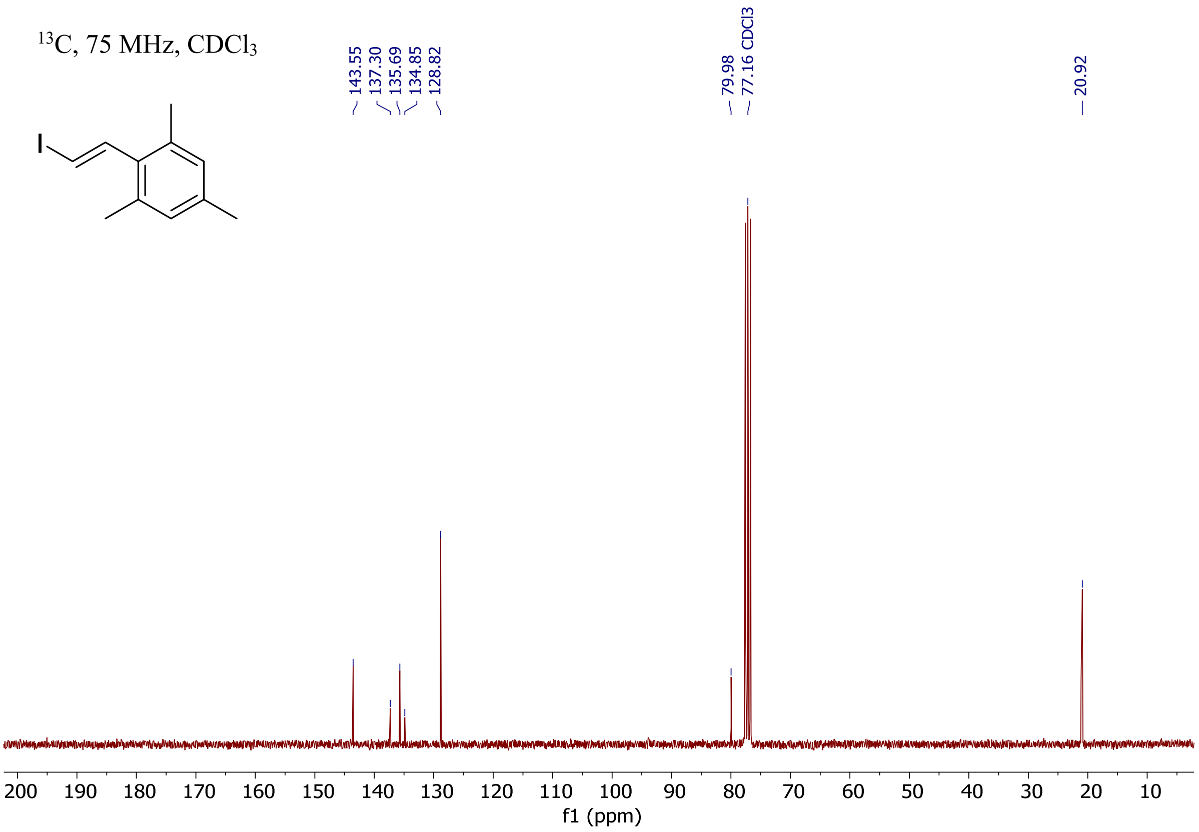
13C, 75 MHz, CDCl3
143.55
137.30
135.69
134.85
128.82
79.98
77.16 CDCl3
20.92
I
200 190 180 170 160 150 140 130 120 110 100 90 80 70 60 50 40 30 20 10
f1 (ppm)

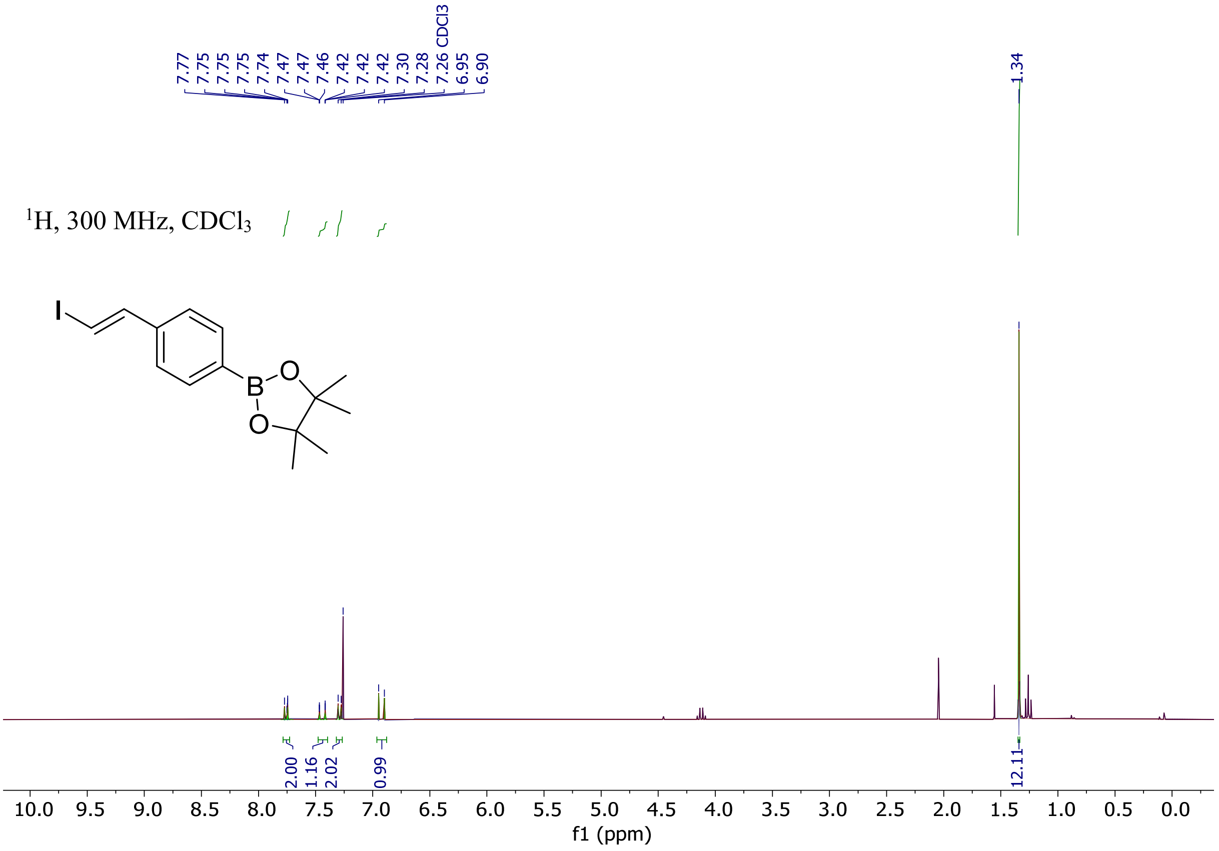
7.77
7.75
7.75
7.75
7.74
7.47
7.47
7.46
7.42
7.42
7.42
7.42
7.30
7.28
7.26 CDCl3
6.95
6.90
1.34
1H, 300 MHz, CDCl3
I
B
O
O
2.00
1.16
2.02
0.99
12.11
10.0 9.5 9.0 8.5 8.0 7.5 7.0 6.5 6.0 5.5 5.0 4.5 4.0 3.5 3.0 2.5 2.0 1.5 1.0 0.5 0.0
f1 (ppm)

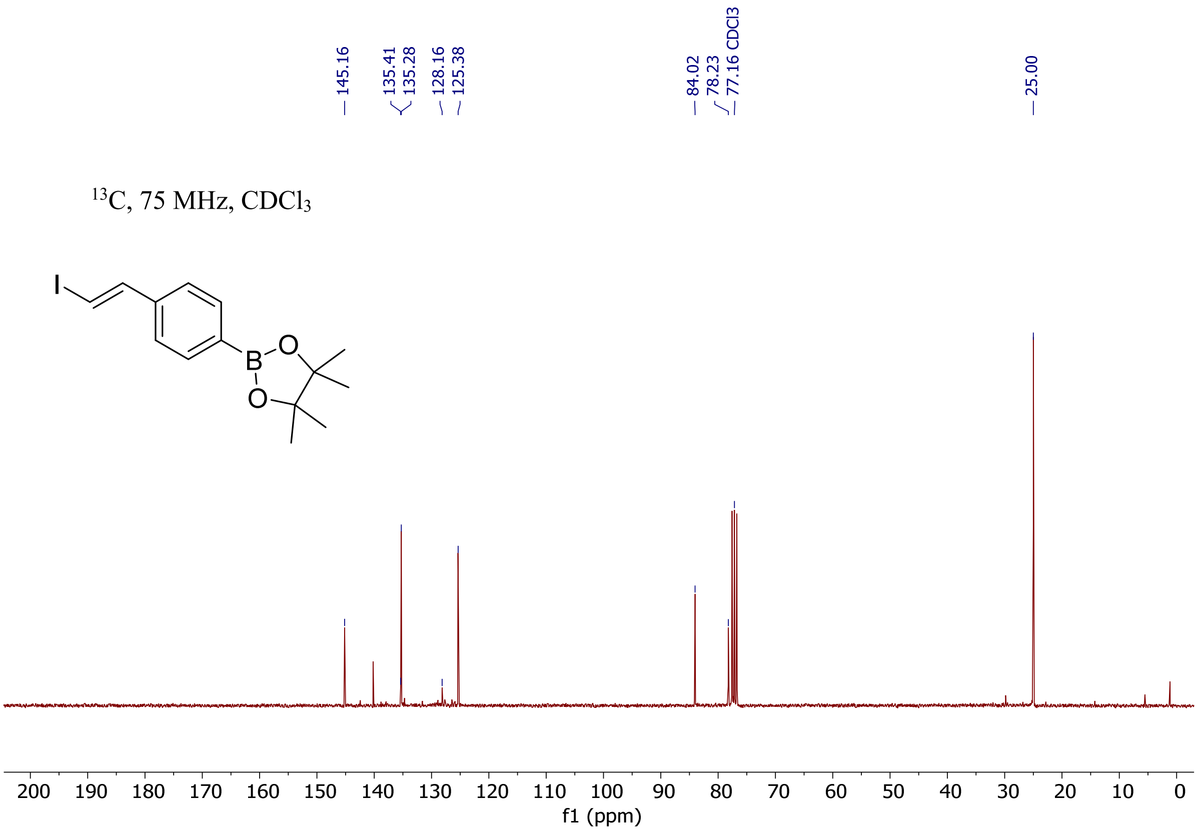

5.3 NMR spectra of hetero-vinylation products

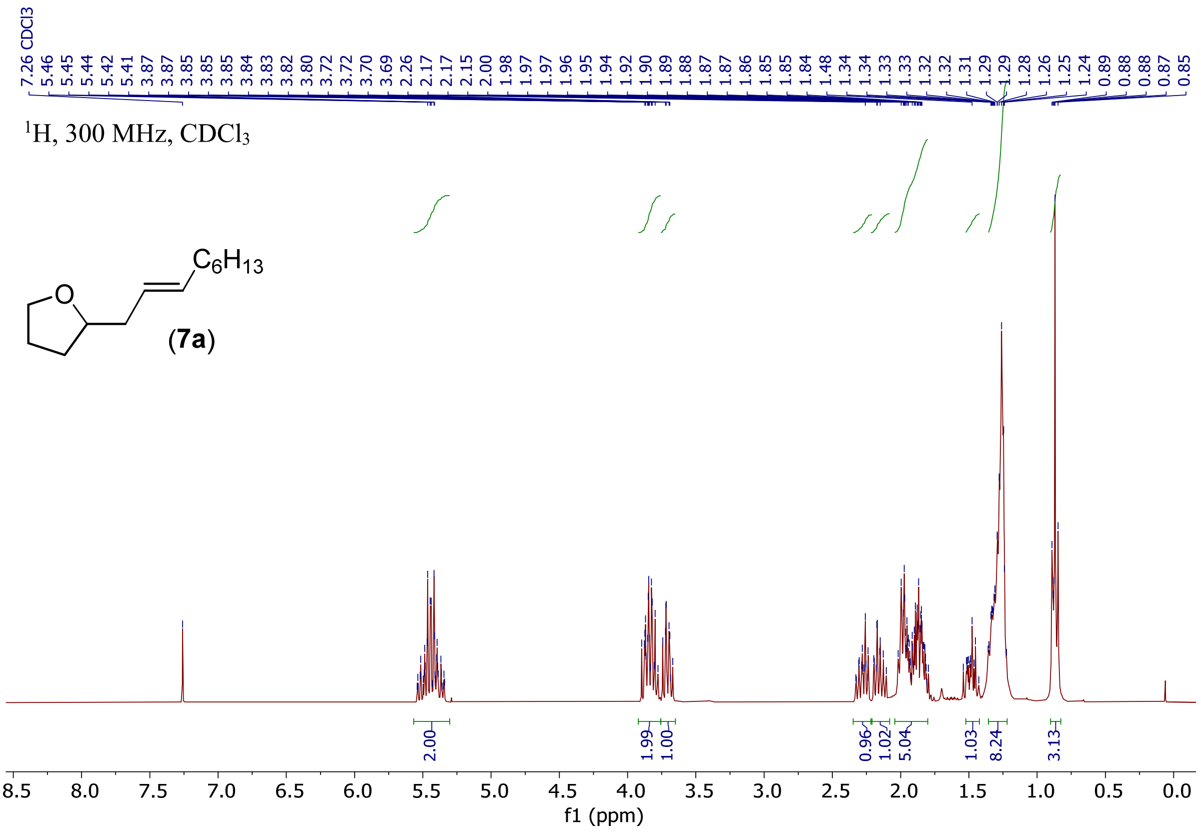

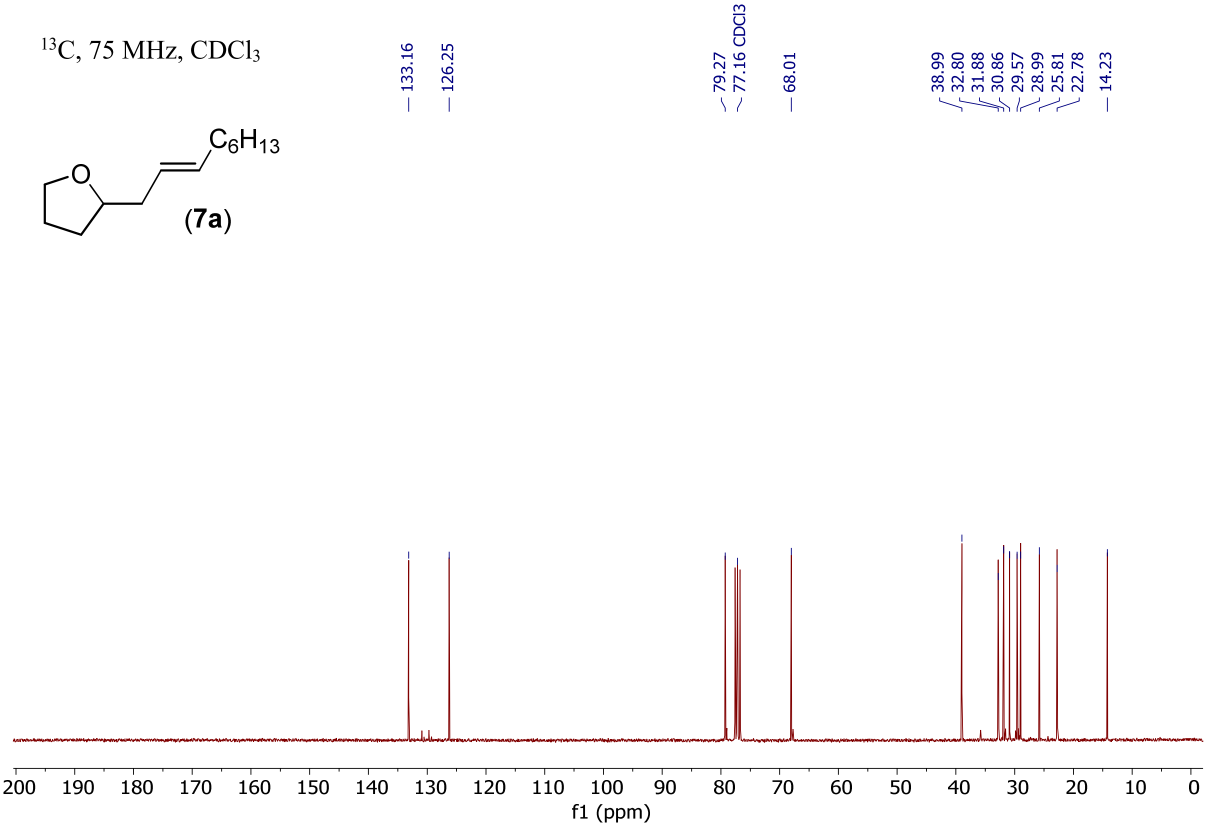
13C, 75 MHz, CDCl3
C6H13
O
(7a)
133.16
126.25
79.27
77.16 CDCl3
68.01
38.99
32.80
31.88
30.86
29.57
28.99
25.81
22.78
14.23
f1 (ppm)

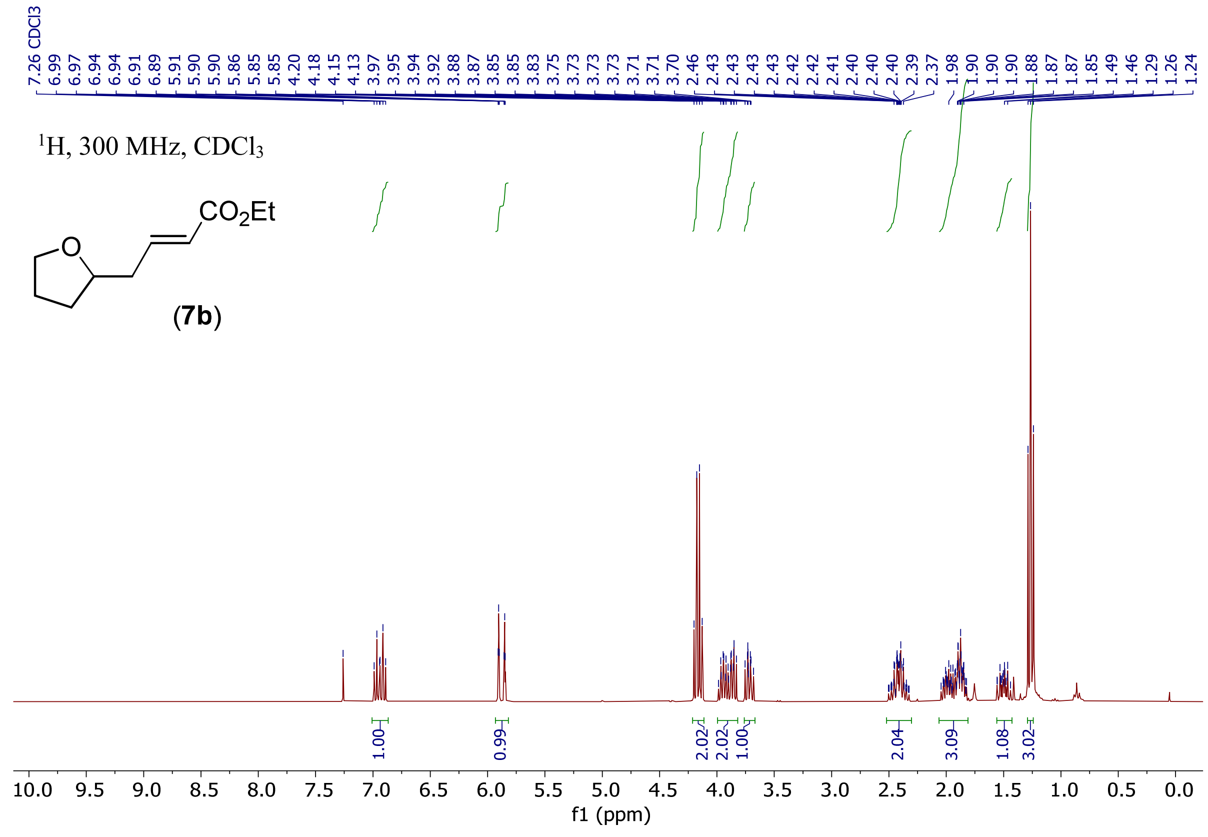
1H, 300 MHz, CDCl3
CO2Et
O
(7b)
1.00
0.99
2.02
2.02
1.00
2.04
3.09
1.08
3.02
f1 (ppm)

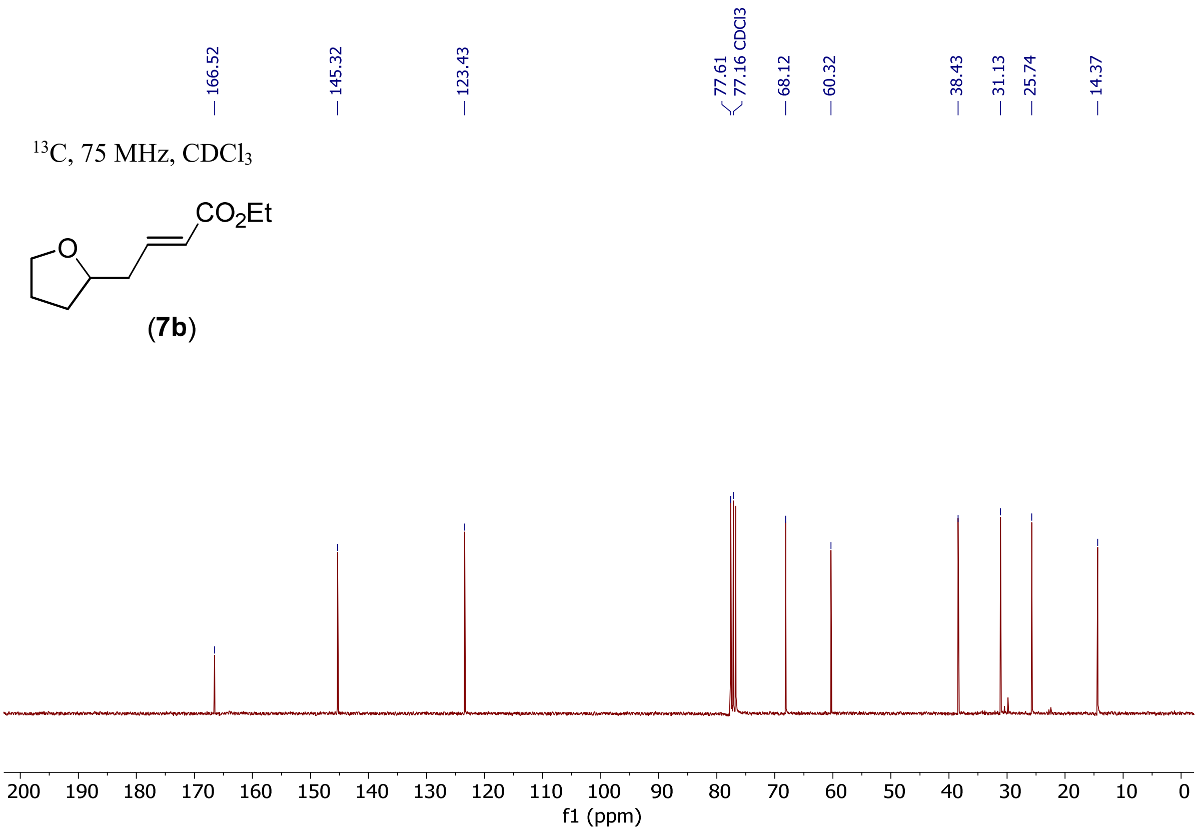
166.52
145.32
123.43
77.61
77.16 CDCl3
68.12
60.32
38.43
31.13
25.74
14.37
13C, 75 MHz, CDCl3
CO2Et
O
(7b)
200 190 180 170 160 150 140 130 120 110 100 90 80 70 60 50 40 30 20 10 0
f1 (ppm)

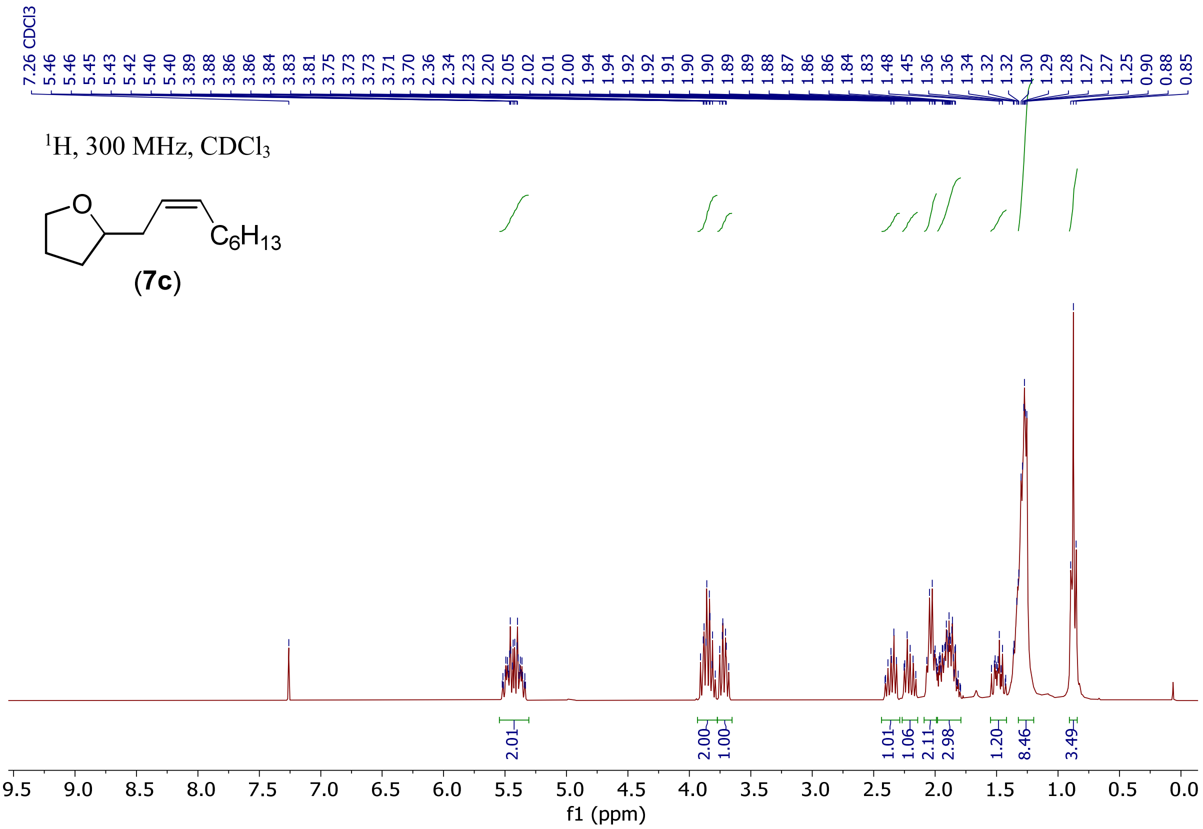
7.26 CDCl3
5.46 5.46 5.45 5.43 5.42 5.40 5.40 3.89 3.88 3.86 3.86 3.84 3.83 3.81 3.75 3.73 3.73 3.71 3.70 2.36 2.34 2.23 2.20 2.05 2.02 2.01 2.00 1.94 1.94 1.92 1.92 1.91 1.90 1.90 1.89 1.89 1.88 1.87 1.86 1.86 1.84 1.83 1.48 1.45 1.36 1.36 1.34 1.32 1.32 1.30 1.29 1.28 1.27 1.27 1.25 0.90 0.88 0.85
1H, 300 MHz, CDCl3
O
C6H13
(7c)
2.01
2.00
1.00
1.01
1.06
2.11
2.98
1.20
8.46
3.49
9.5 9.0 8.5 8.0 7.5 7.0 6.5 6.0 5.5 5.0 4.5 4.0 3.5 3.0 2.5 2.0 1.5 1.0 0.5 0.0
f1 (ppm)

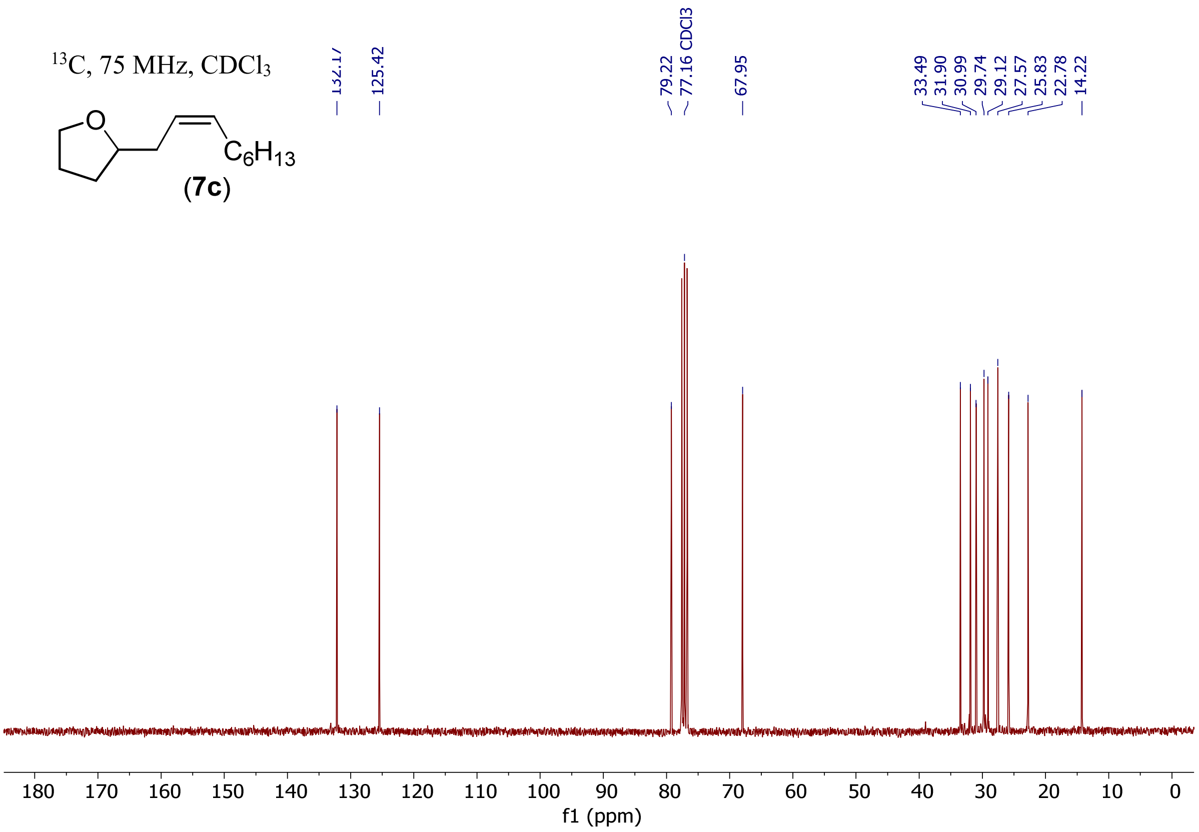

13C, 75 MHz, CDCl3
(7c)
132.17
125.42
79.22
77.16 CDCl3
67.95
33.49
31.90
30.99
29.74
29.12
27.57
25.83
22.78
14.22
f1 (ppm)

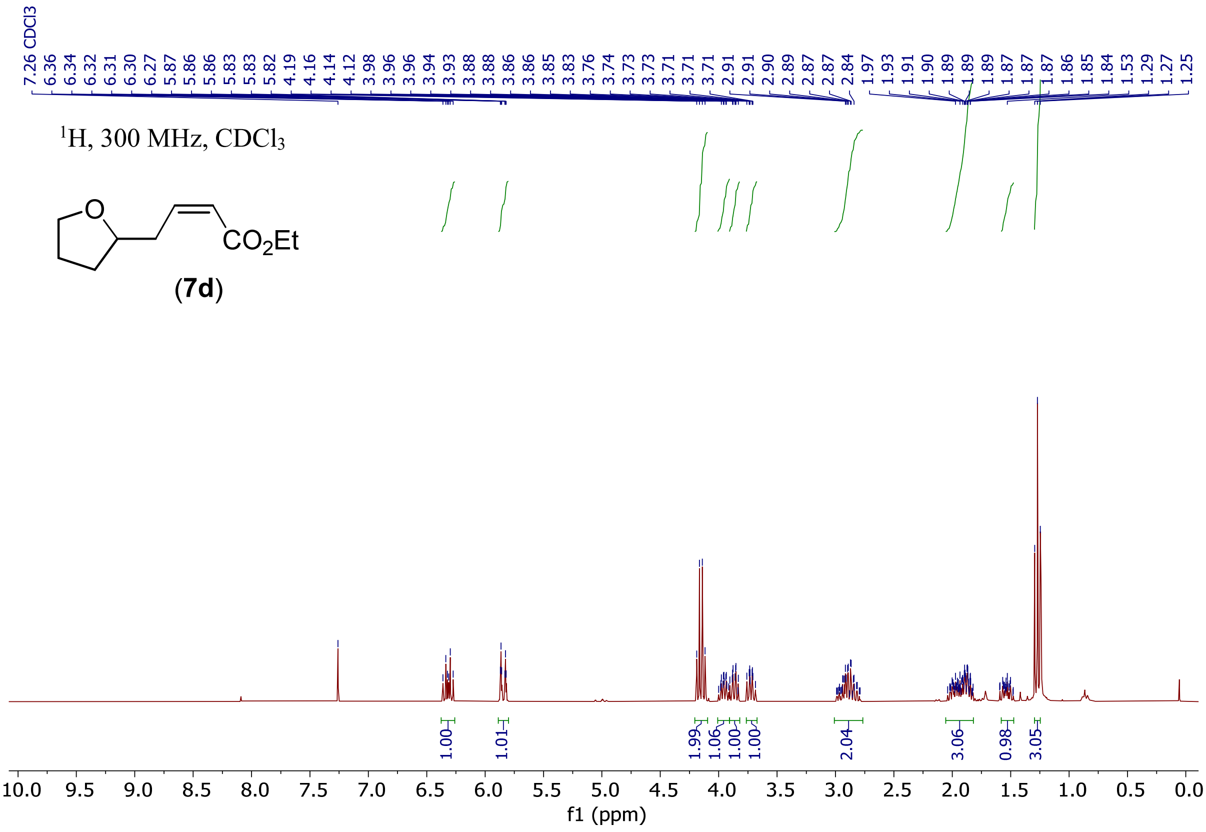

1H, 300 MHz, CDCl3
(7d)
f1 (ppm)

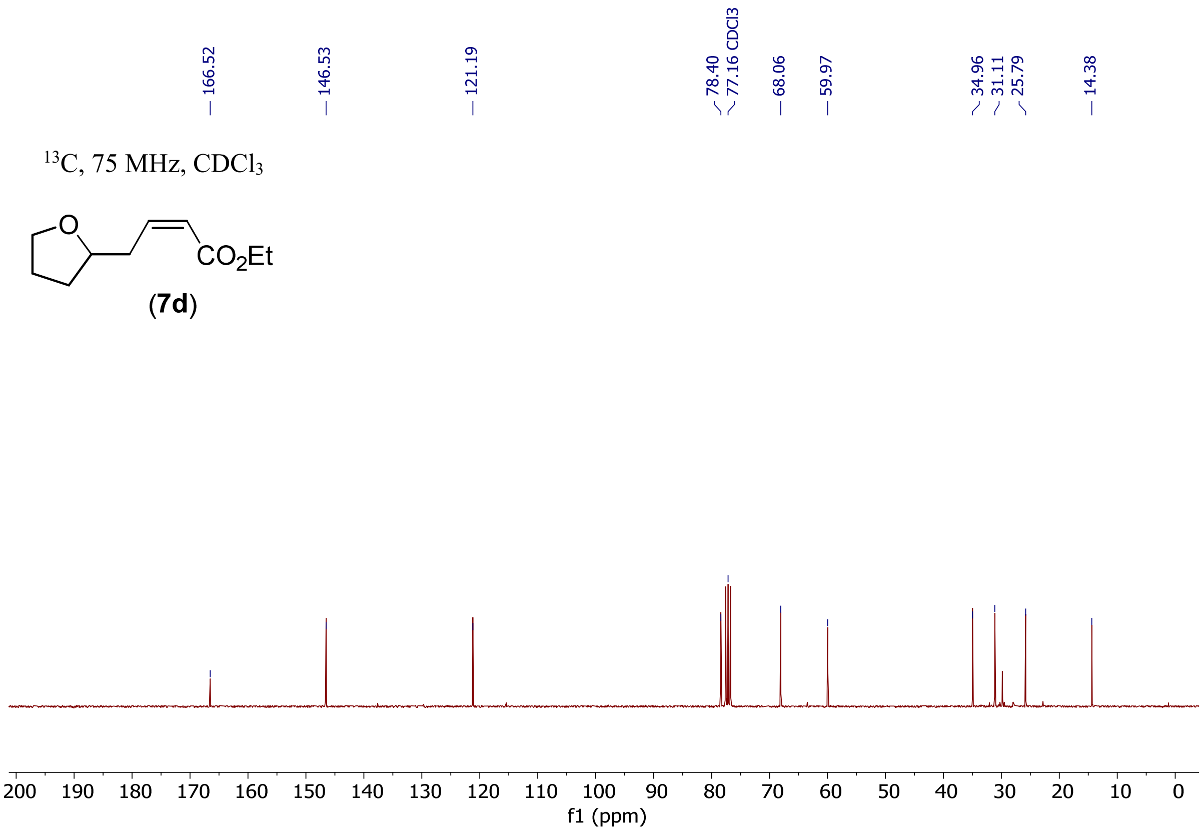

166.52
146.53
121.19
78.40
77.16 CDCl3
68.06
59.97
34.96
31.11
25.79
14.38
^{13}C, 75 MHz, $CDCl_3$
O
CO_2Et
(7d)
200 190 180 170 160 150 140 130 120 110 100 90 80 70 60 50 40 30 20 10 0
f1 (ppm)

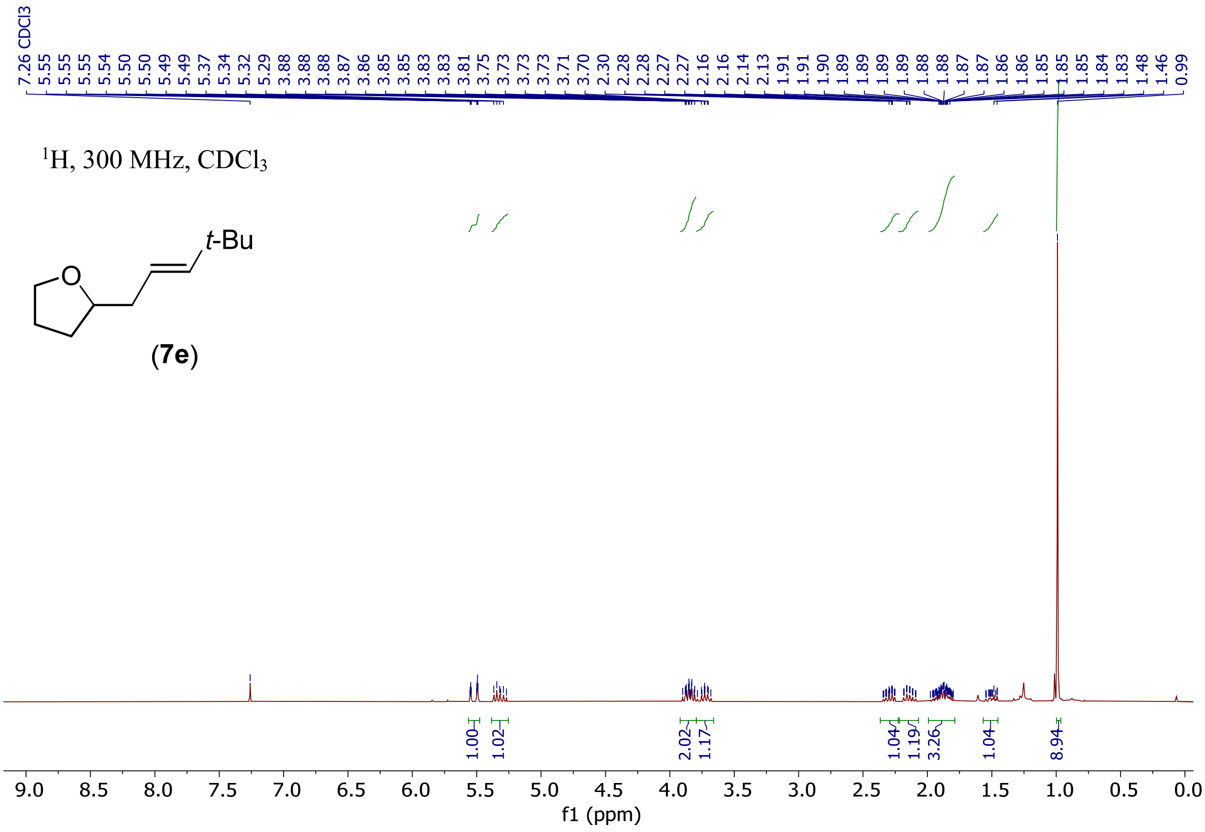

7.26 CDCl3
5.55 5.55 5.55 5.54 5.50 5.50 5.49 5.49 5.37 5.34 5.32 5.29 3.88 3.88 3.88 3.87 3.86 3.85 3.85 3.83 3.83 3.81 3.75 3.73 3.73 3.73 3.71 3.70 2.30 2.28 2.28 2.27 2.27 2.16 2.16 2.14 2.13 1.91 1.91 1.90 1.89 1.89 1.89 1.89 1.88 1.88 1.87 1.87 1.86 1.86 1.85 1.85 1.85 1.84 1.83 1.48 1.46 0.99
^{1}H, 300 MHz, $CDCl_3$
t-Bu
O
(7e)
1.00 1.02 2.02 1.17 1.04 1.19 3.26 1.04 8.94
9.0 8.5 8.0 7.5 7.0 6.5 6.0 5.5 5.0 4.5 4.0 3.5 3.0 2.5 2.0 1.5 1.0 0.5 0.0
f1 (ppm)

^{13}C, 75 MHz, $CDCl_3$

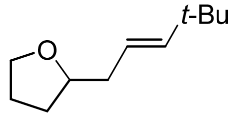

(**7e**)

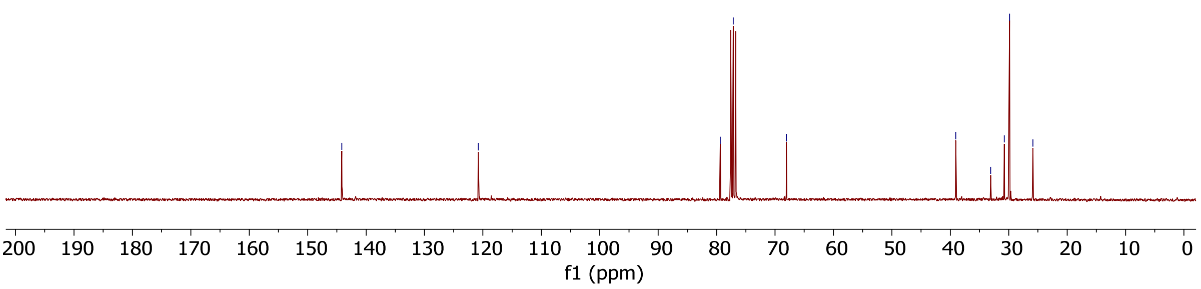

^{1}H, 300 MHz, $CDCl_3$

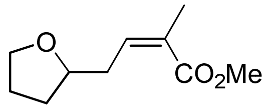

(**7g**)

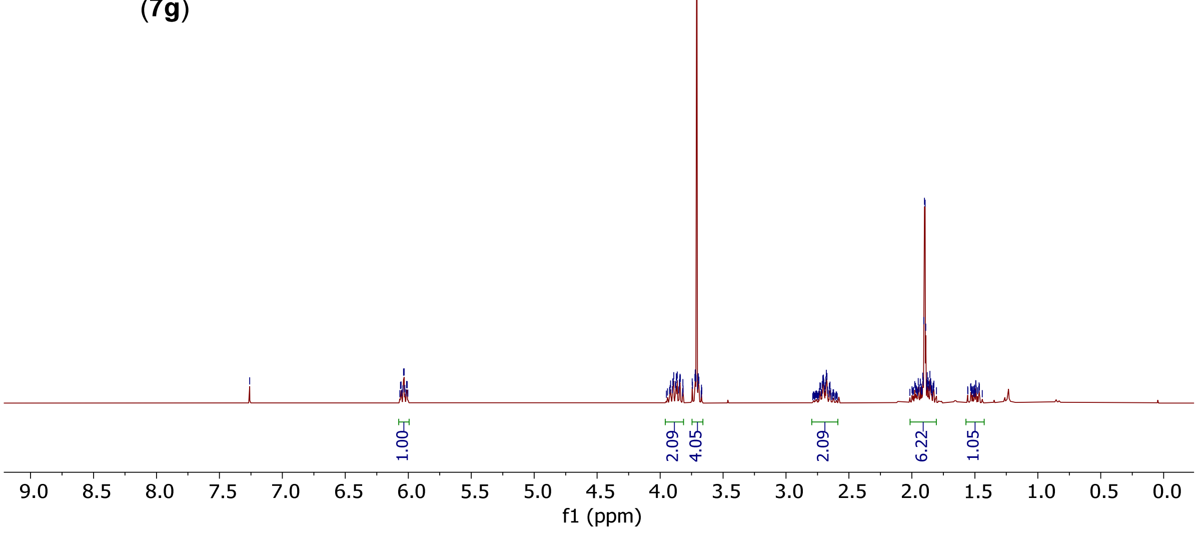

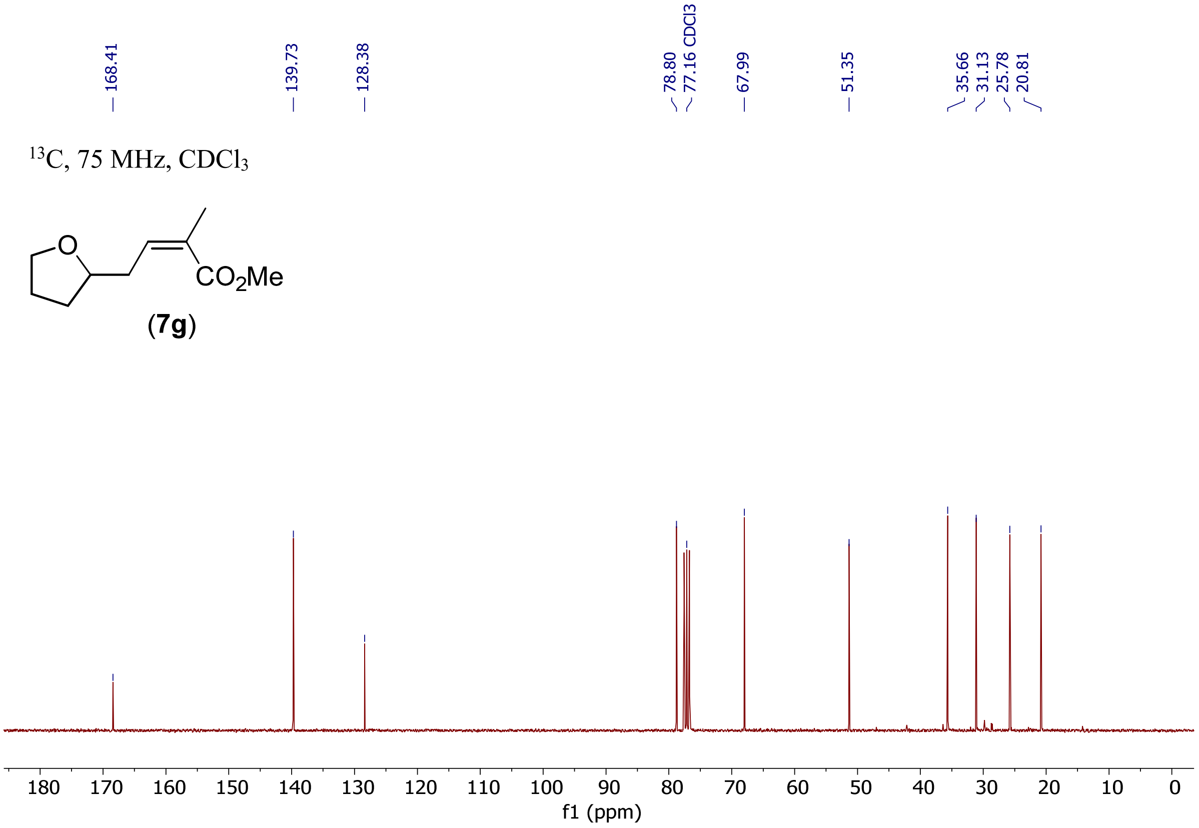
168.41
139.73
128.38
78.80
77.16 CDCl3
67.99
51.35
35.66
31.13
25.78
20.81
13C, 75 MHz, CDCl3
O
CO2Me
(7g)
180 170 160 150 140 130 120 110 100 90 80 70 60 50 40 30 20 10 0
f1 (ppm)

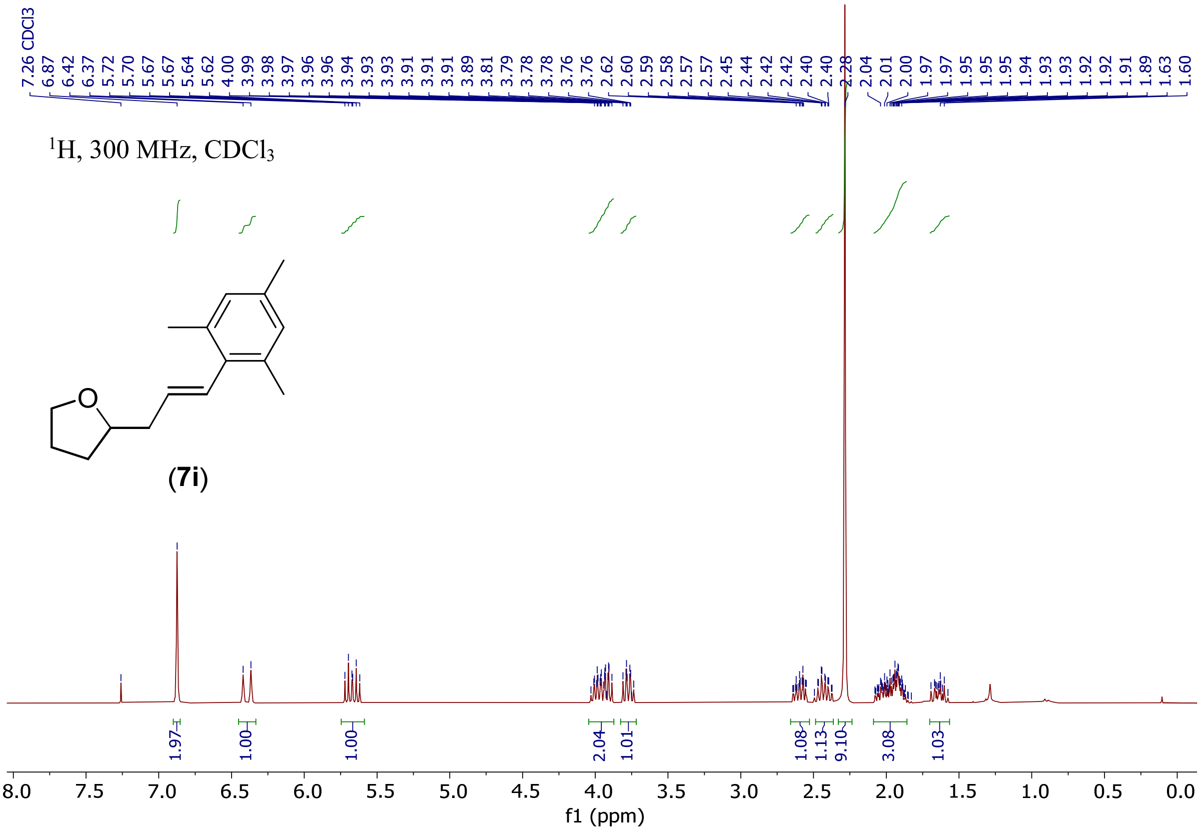
7.26 CDCl3
6.87
6.42
6.37
5.72
5.70
5.67
5.67
5.64
5.62
4.00
3.99
3.98
3.97
3.96
3.96
3.94
3.93
3.93
3.91
3.91
3.91
3.89
3.81
3.79
3.78
3.78
3.76
3.76
2.62
2.60
2.59
2.58
2.57
2.57
2.45
2.44
2.42
2.42
2.40
2.40
2.28
2.04
2.01
2.00
1.97
1.97
1.95
1.95
1.95
1.94
1.93
1.93
1.92
1.92
1.91
1.89
1.63
1.60
1H, 300 MHz, CDCl3
O
(7i)
1.97
1.00
1.00
2.04
1.01
1.08
1.13
9.10
3.08
1.03
8.0 7.5 7.0 6.5 6.0 5.5 5.0 4.5 4.0 3.5 3.0 2.5 2.0 1.5 1.0 0.5 0.0
f1 (ppm)

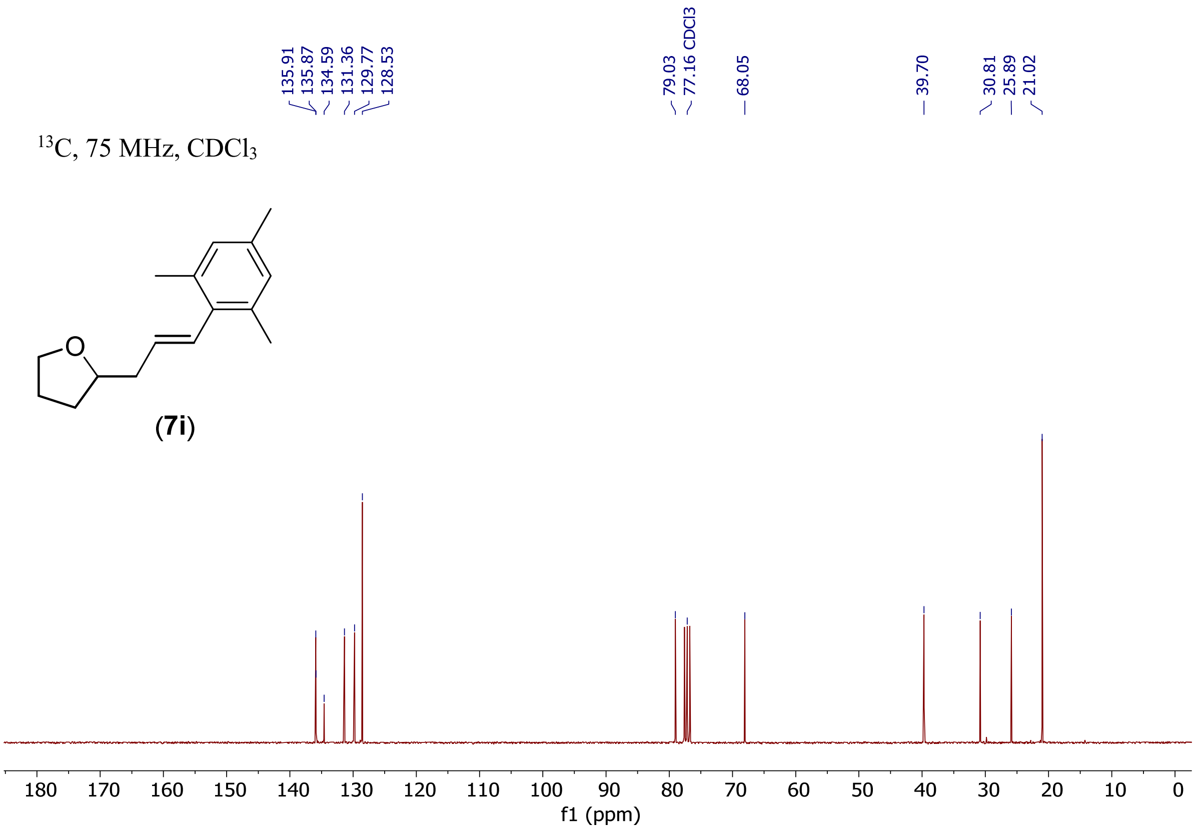
135.91
135.87
134.59
131.36
129.77
128.53
79.03
77.16 CDCl3
68.05
39.70
30.81
25.89
21.02
^{13}C, 75 MHz, $CDCl_3$
O
(7i)
180 170 160 150 140 130 120 110 100 90 80 70 60 50 40 30 20 10 0
f1 (ppm)

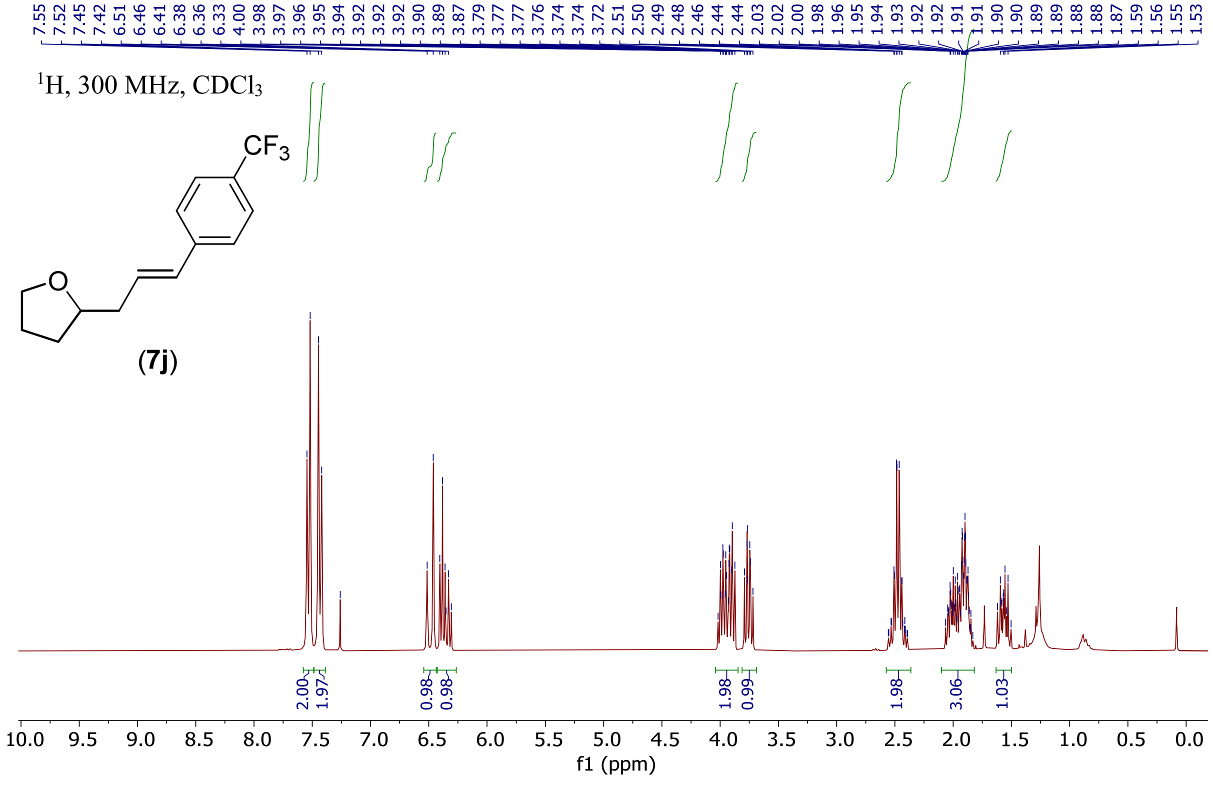
7.55 7.52 7.45 7.42 6.51 6.46 6.41 6.38 6.36 6.33 4.00 3.98 3.97 3.96 3.95 3.94 3.92 3.92 3.92 3.90 3.89 3.87 3.79 3.77 3.77 3.76 3.74 3.74 3.72 2.51 2.50 2.49 2.48 2.46 2.44 2.44 2.03 2.02 2.00 1.98 1.96 1.95 1.94 1.93 1.92 1.92 1.91 1.91 1.90 1.90 1.89 1.89 1.88 1.87 1.59 1.56 1.55 1.53
^{1}H, 300 MHz, $CDCl_3$
CF_3
O
(7j)
2.00 1.97 0.98 0.98 1.98 0.99 1.98 3.06 1.03
10.0 9.5 9.0 8.5 8.0 7.5 7.0 6.5 6.0 5.5 5.0 4.5 4.0 3.5 3.0 2.5 2.0 1.5 1.0 0.5 0.0
f1 (ppm)

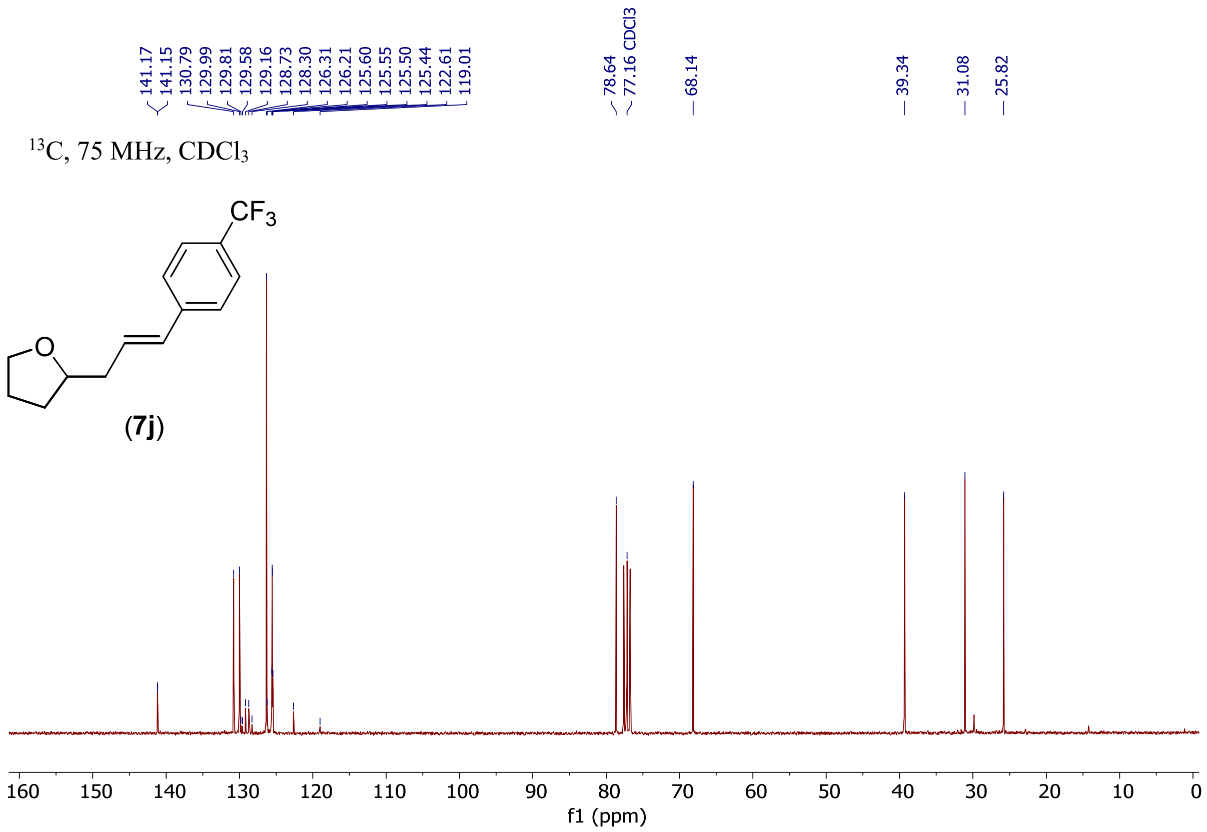
141.17
141.15
130.79
129.99
129.81
129.58
129.16
128.73
128.30
126.31
126.21
125.60
125.55
125.50
125.44
122.61
119.01
78.64
77.16 CDCl3
68.14
39.34
31.08
25.82
13C, 75 MHz, CDCl3
CF3
O
(7j)
160 150 140 130 120 110 100 90 80 70 60 50 40 30 20 10 0
f1 (ppm)

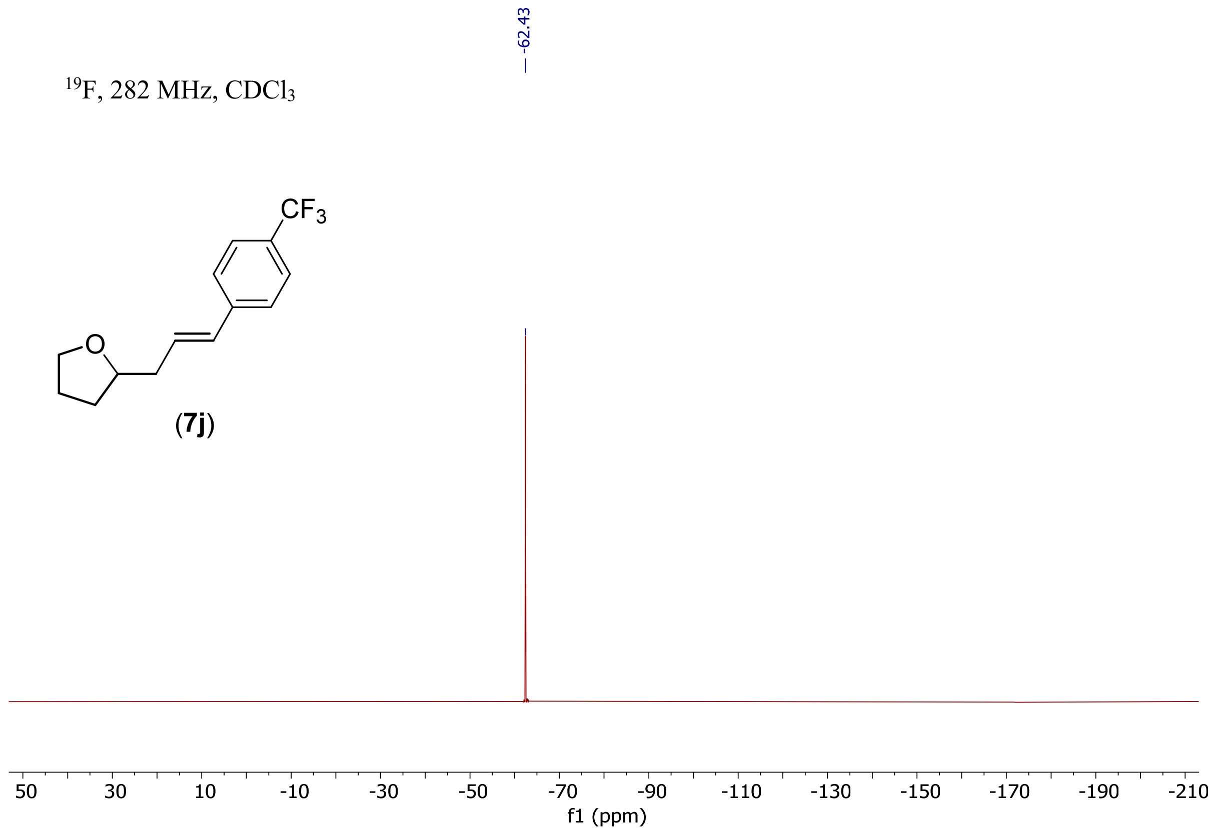
-62.43
19F, 282 MHz, CDCl3
CF3
O
(7j)
50 30 10 -10 -30 -50 -70 -90 -110 -130 -150 -170 -190 -210
f1 (ppm)

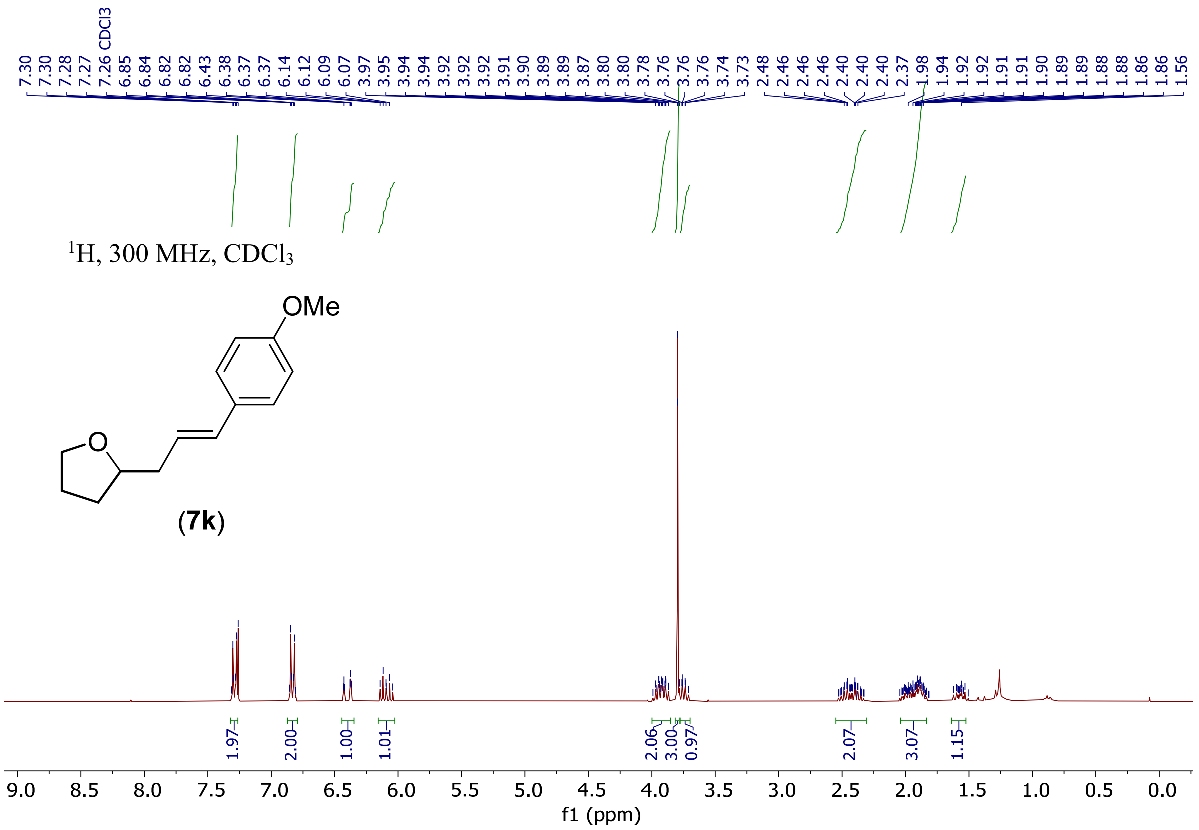

^{1}H, 300 MHz, CDCl$_3$
OMe
O
(**7k**)
f1 (ppm)

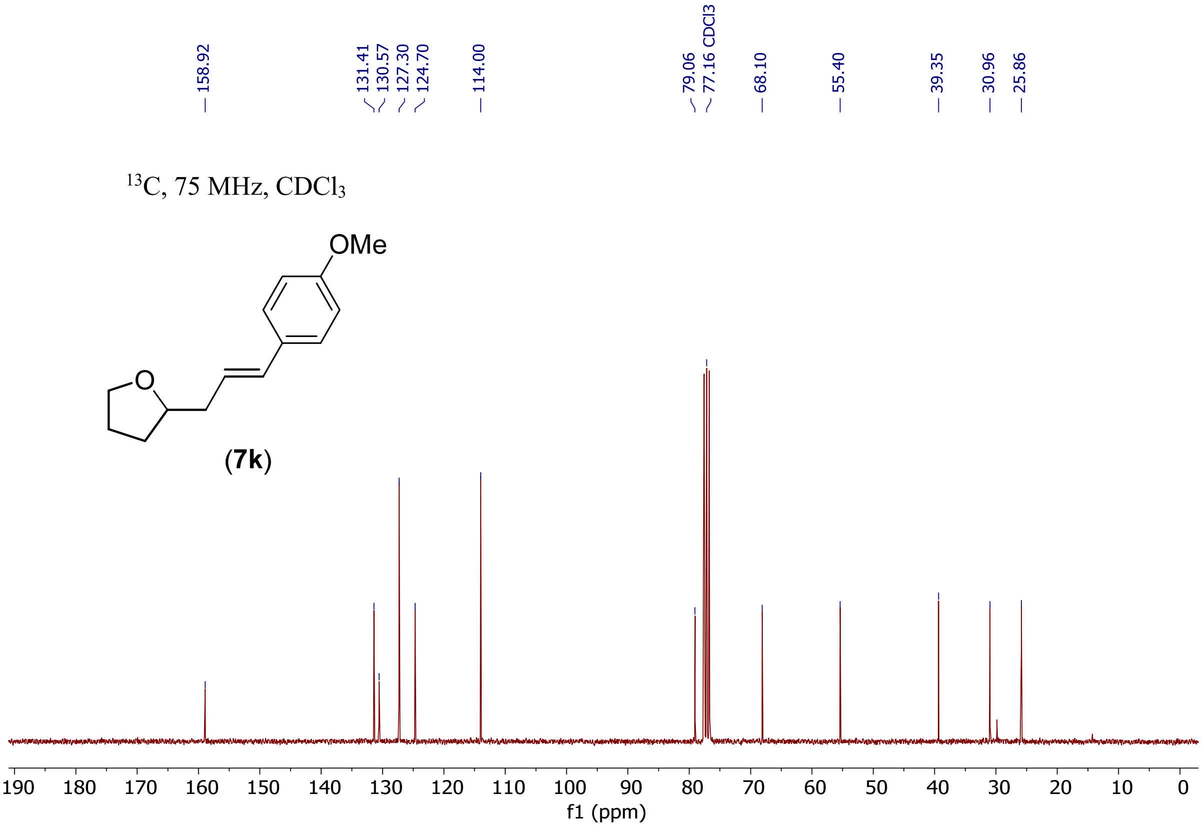

^{13}C, 75 MHz, CDCl$_3$
OMe
O
(**7k**)
158.92
131.41
130.57
127.30
124.70
114.00
79.06
77.16 CDCl3
68.10
55.40
39.35
30.96
25.86
f1 (ppm)

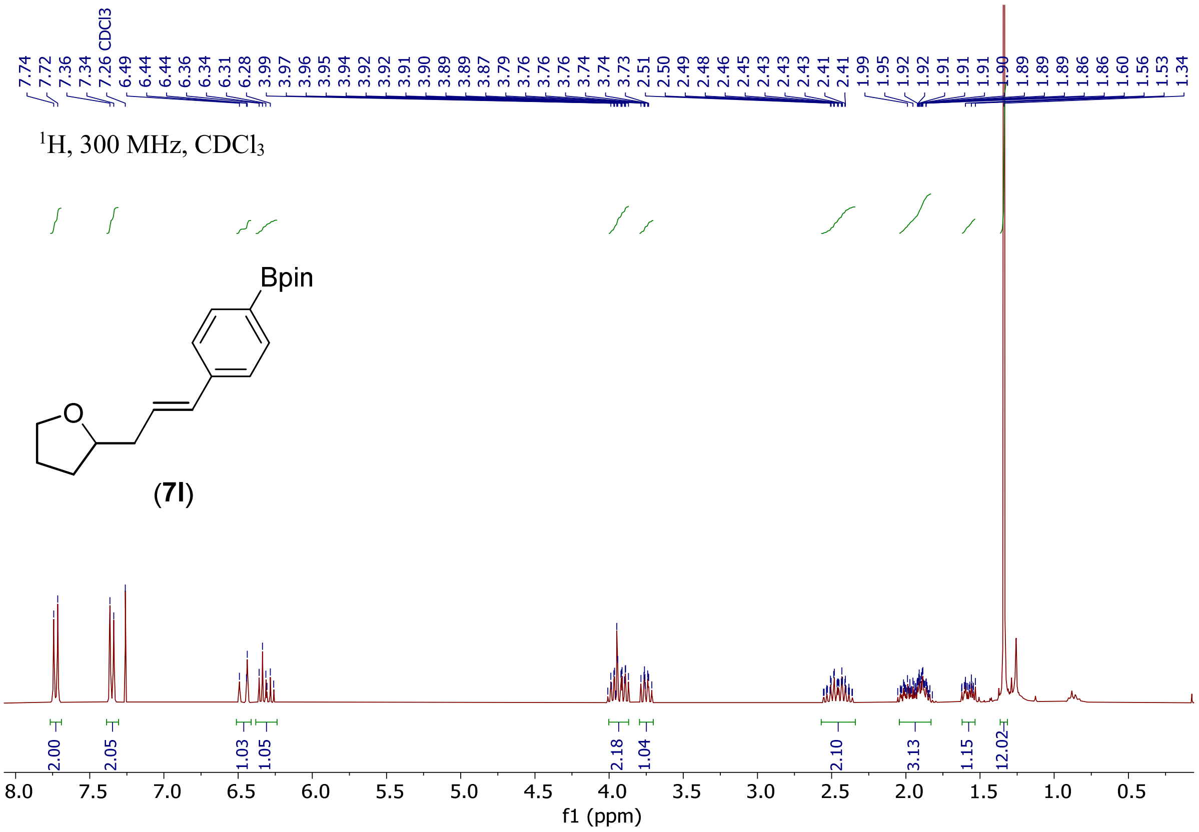
7.74 7.72 7.36 7.34 7.26 CDCl3 6.49 6.44 6.44 6.36 6.34 6.31 6.28 3.99 3.97 3.96 3.95 3.94 3.92 3.92 3.91 3.90 3.89 3.89 3.87 3.79 3.76 3.76 3.74 3.74 3.73 2.51 2.50 2.49 2.48 2.46 2.45 2.43 2.43 2.43 2.41 2.41 1.99 1.95 1.92 1.92 1.91 1.91 1.91 1.90 1.89 1.89 1.89 1.86 1.86 1.60 1.56 1.53 1.34
1H, 300 MHz, CDCl3
Bpin
O
(7l)
2.00 2.05 1.03 1.05 2.18 1.04 2.10 3.13 1.15 12.02
8.0 7.5 7.0 6.5 6.0 5.5 5.0 4.5 4.0 3.5 3.0 2.5 2.0 1.5 1.0 0.5
f1 (ppm)

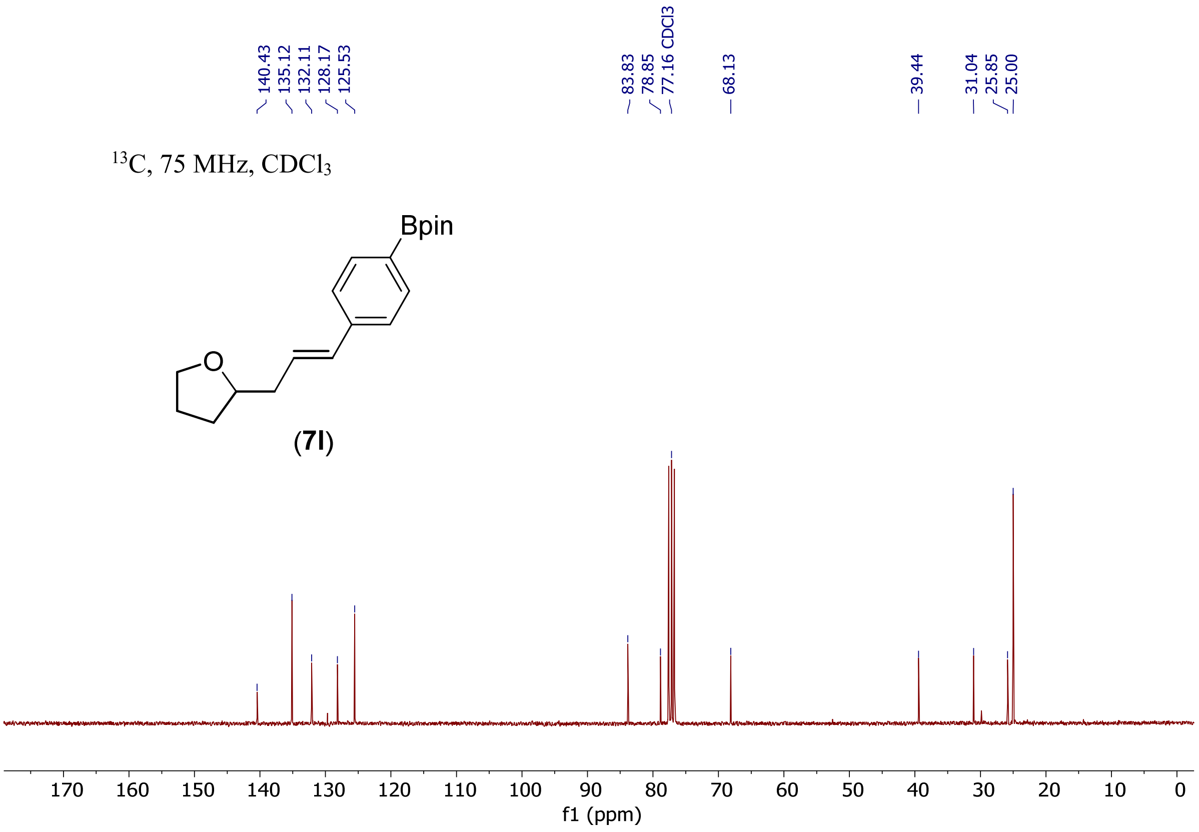
140.43 135.12 132.11 128.17 125.53 83.83 78.85 77.16 CDCl3 68.13 39.44 31.04 25.85 25.00
13C, 75 MHz, CDCl3
Bpin
O
(7l)
170 160 150 140 130 120 110 100 90 80 70 60 50 40 30 20 10 0
f1 (ppm)

^{1}H, 300 MHz, $CDCl_3$

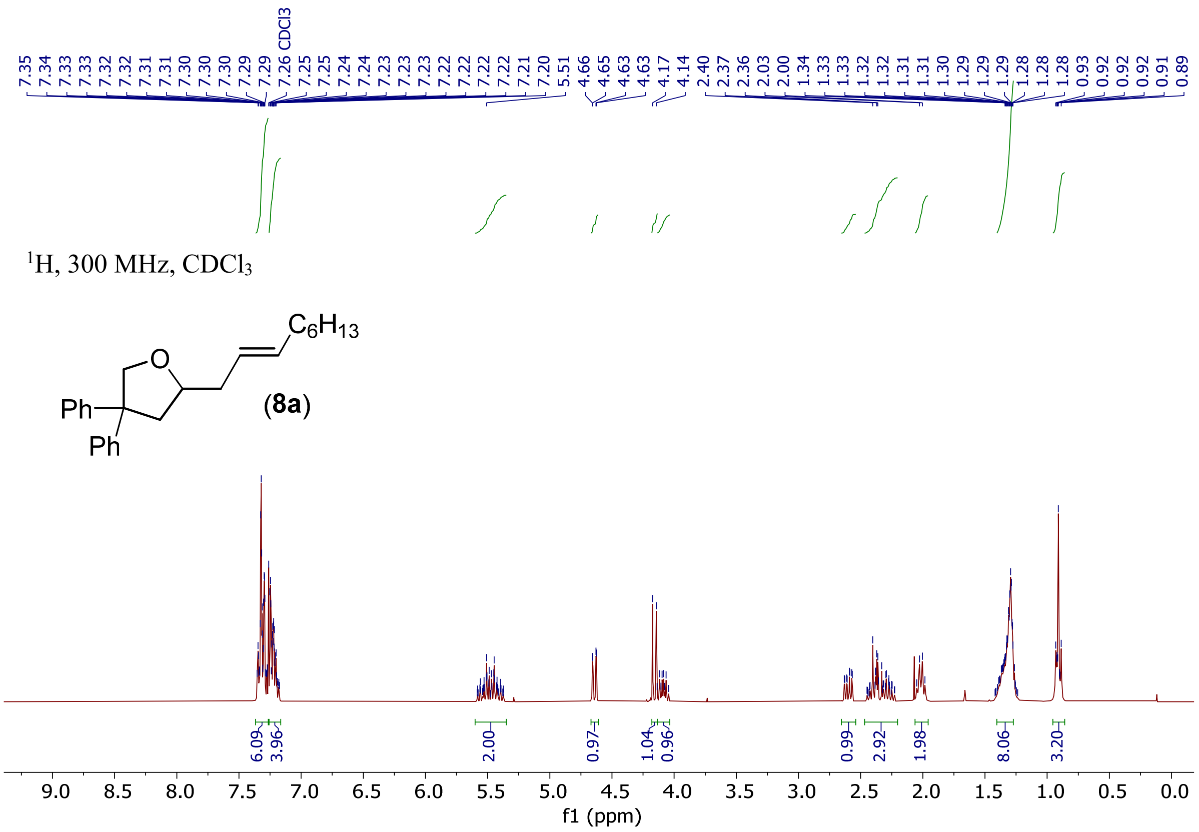

^{13}C, 75 MHz, $CDCl_3$

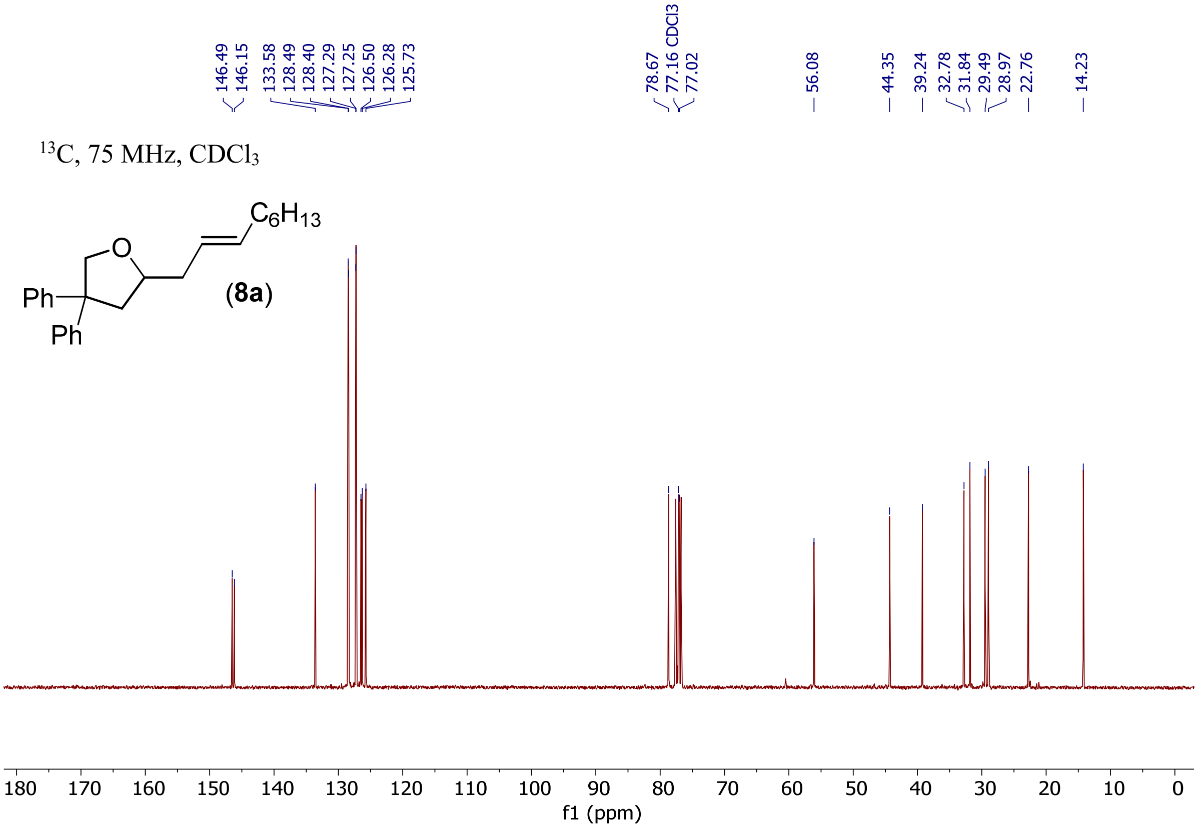

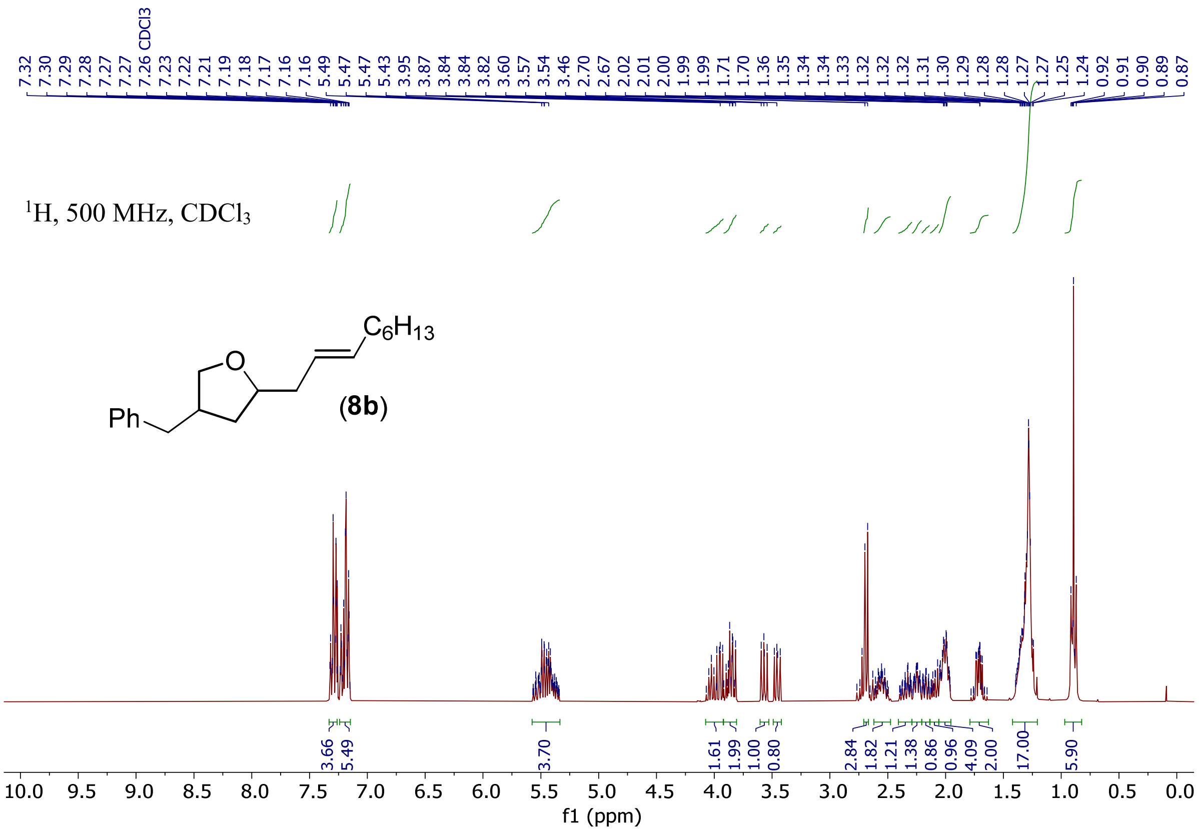
7.32 7.30 7.29 7.28 7.27 7.27 7.26 CDCl3 7.23 7.22 7.21 7.19 7.18 7.17 7.16 7.16 5.49 5.47 5.47 5.43 3.95 3.87 3.84 3.84 3.82 3.60 3.57 3.54 3.46 2.70 2.67 2.02 2.01 2.00 1.99 1.99 1.71 1.70 1.36 1.35 1.34 1.34 1.33 1.32 1.32 1.32 1.31 1.30 1.29 1.28 1.28 1.27 1.27 1.25 1.24 0.92 0.91 0.90 0.89 0.87
¹H, 500 MHz, CDCl₃
C6H13
O
Ph
(8b)
3.66 5.49 3.70 1.61 1.99 1.00 0.80 2.84 1.82 1.21 1.38 0.86 0.96 4.09 2.00 17.00 5.90
10.0 9.5 9.0 8.5 8.0 7.5 7.0 6.5 6.0 5.5 5.0 4.5 4.0 3.5 3.0 2.5 2.0 1.5 1.0 0.5 0.0
f1 (ppm)

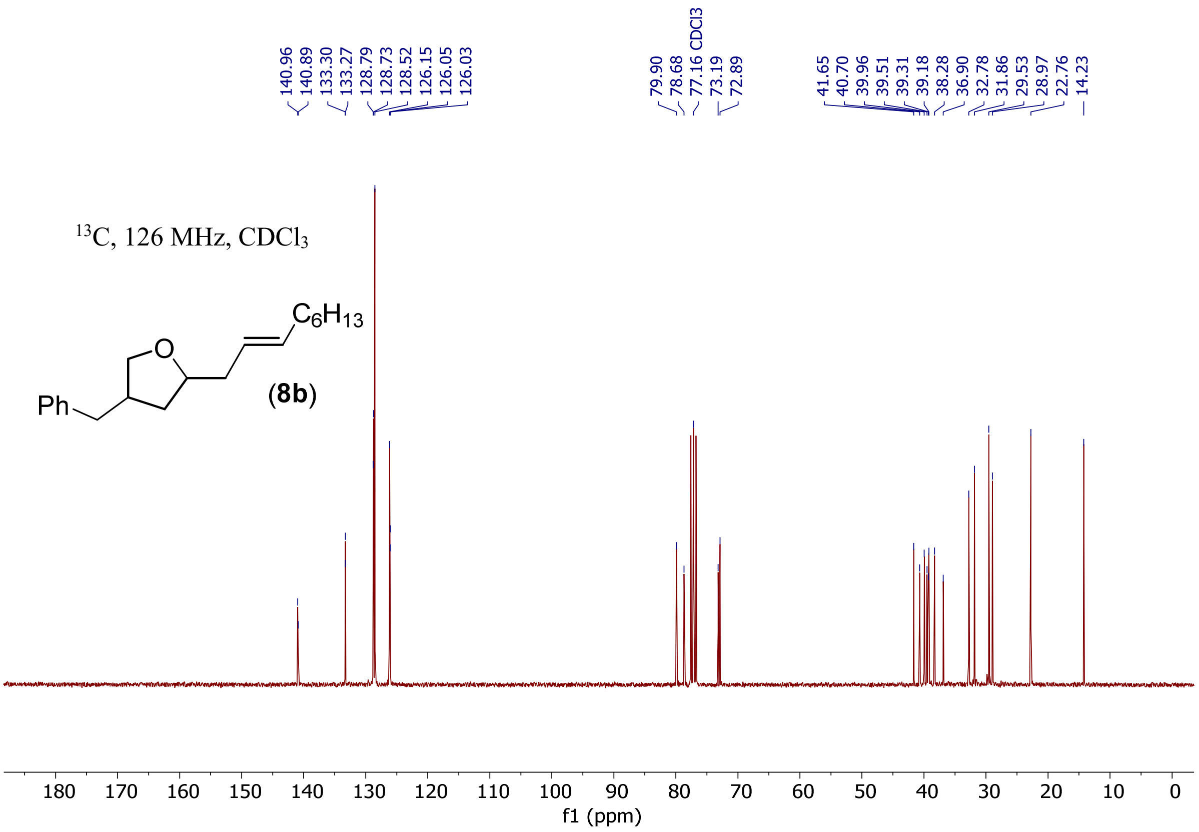
140.96 140.89 133.30 133.27 128.79 128.73 128.52 126.15 126.05 126.03
79.90 78.68 77.16 CDCl3 73.19 72.89
41.65 40.70 39.96 39.51 39.31 39.18 38.28 36.90 32.78 31.86 29.53 28.97 22.76 14.23
¹³C, 126 MHz, CDCl₃
C6H13
O
Ph
(8b)
180 170 160 150 140 130 120 110 100 90 80 70 60 50 40 30 20 10 0
f1 (ppm)

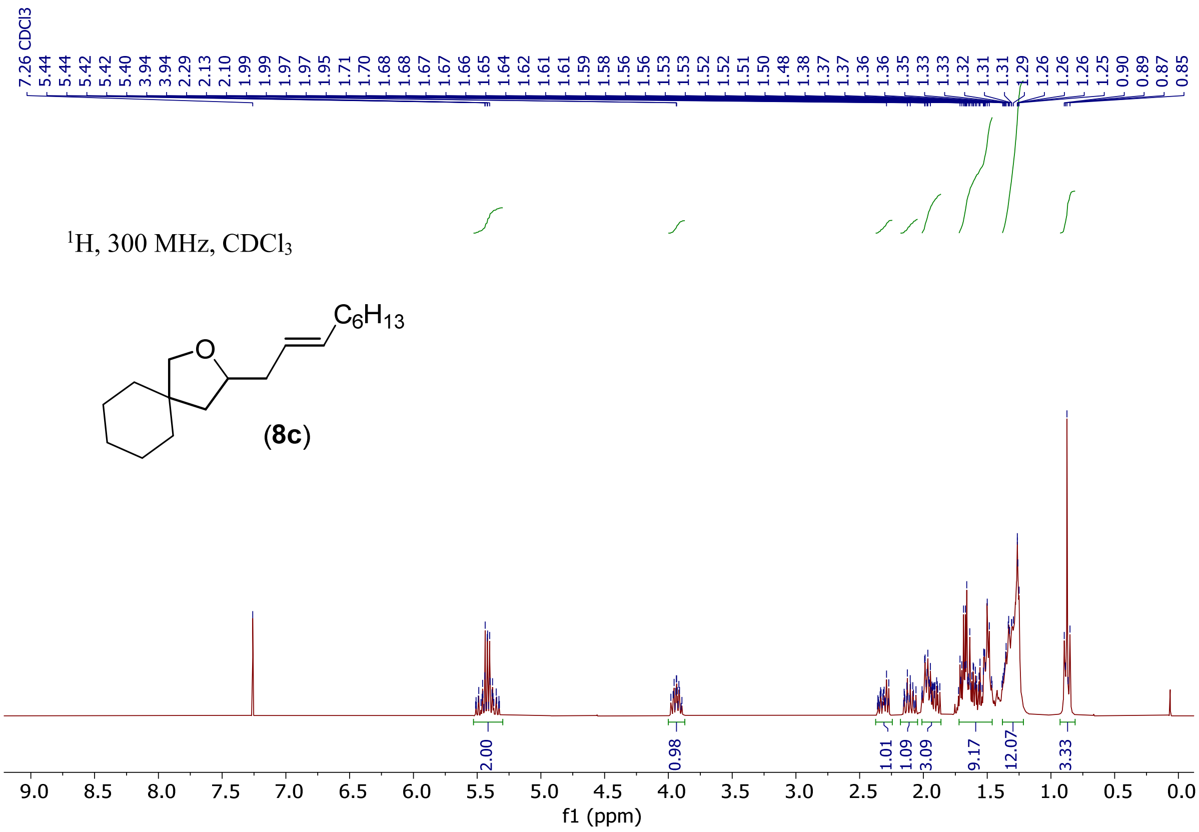
7.26 CDCl3 5.44 5.44 5.42 5.42 5.40 3.94 3.94 2.29 2.13 2.10 1.99 1.99 1.97 1.97 1.95 1.71 1.70 1.68 1.68 1.67 1.67 1.66 1.65 1.64 1.62 1.61 1.61 1.59 1.58 1.56 1.56 1.53 1.53 1.52 1.52 1.51 1.50 1.48 1.38 1.37 1.37 1.36 1.36 1.35 1.33 1.33 1.32 1.31 1.31 1.29 1.26 1.26 1.26 1.25 0.90 0.89 0.87 0.85
¹H, 300 MHz, CDCl₃
C6H13
O
(8c)
2.00
0.98
1.01
1.09
3.09
9.17
12.07
3.33
9.0 8.5 8.0 7.5 7.0 6.5 6.0 5.5 5.0 4.5 4.0 3.5 3.0 2.5 2.0 1.5 1.0 0.5 0.0
f1 (ppm)

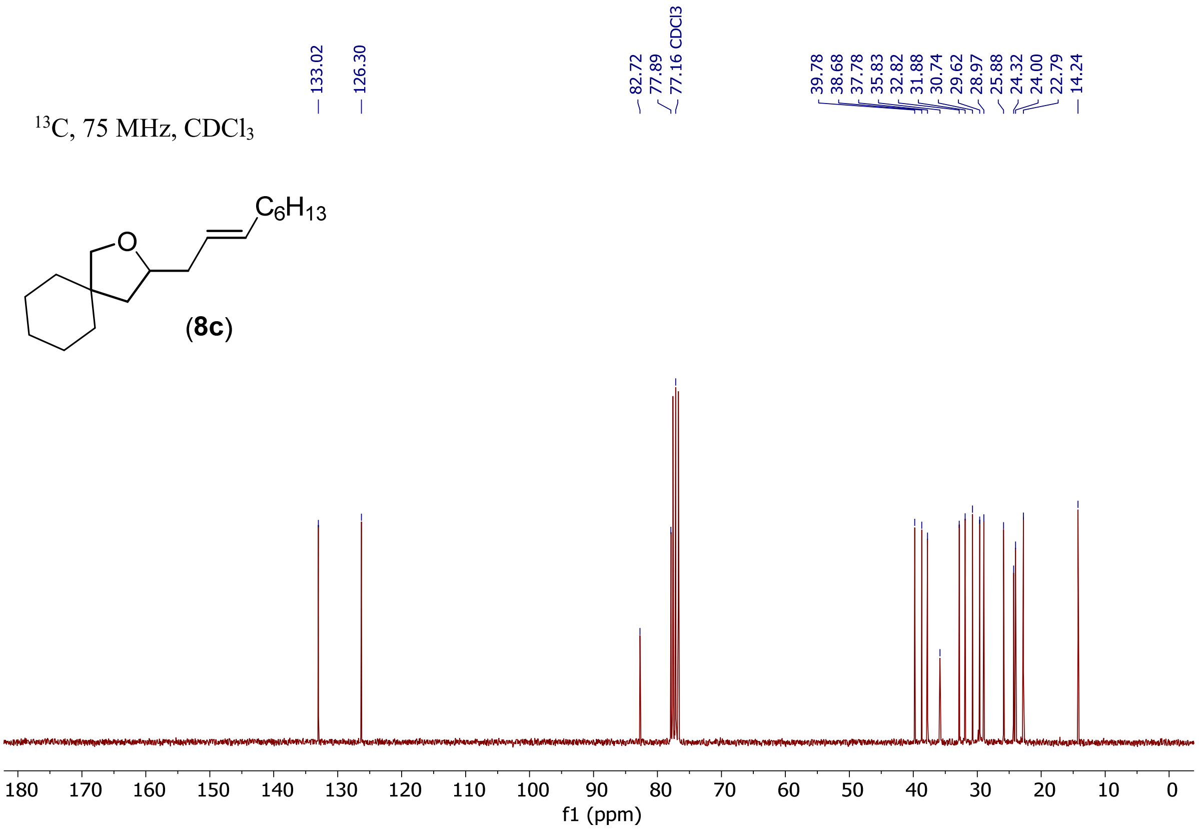
133.02
126.30
82.72 77.89 77.16 CDCl3
39.78 38.68 37.78 35.83 32.82 31.88 30.74 29.62 28.97 25.88 24.32 24.00 22.79
14.24
¹³C, 75 MHz, CDCl₃
C6H13
O
(8c)
180 170 160 150 140 130 120 110 100 90 80 70 60 50 40 30 20 10 0
f1 (ppm)

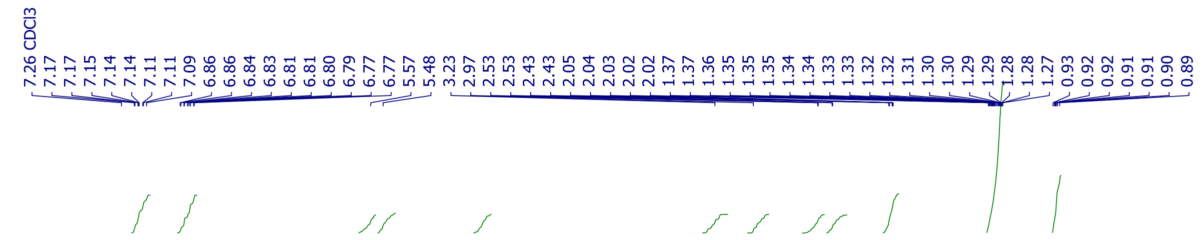

^{1}H, 300 MHz, $CDCl_3$

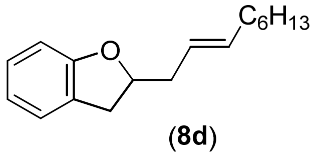

(**8d**)

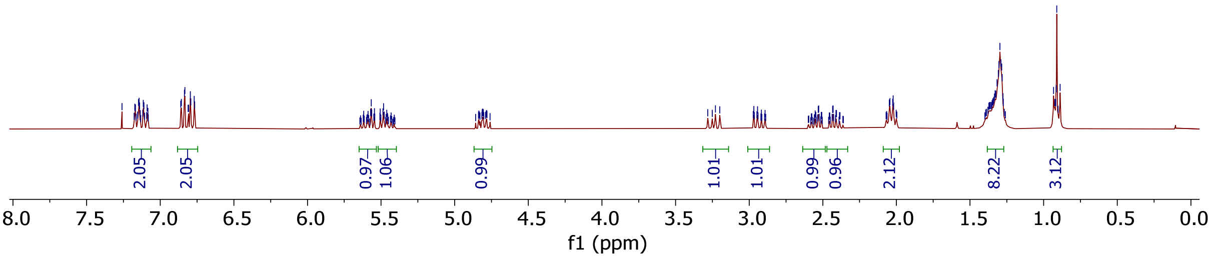

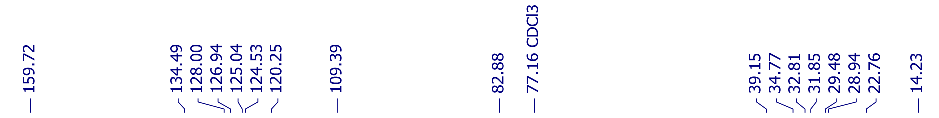

^{13}C, 75 MHz, $CDCl_3$

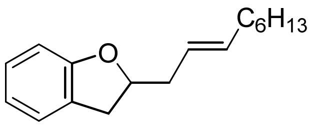

(**8d**)

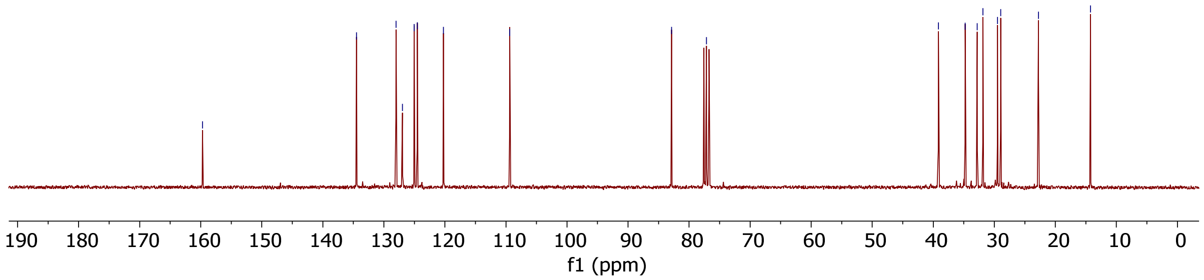

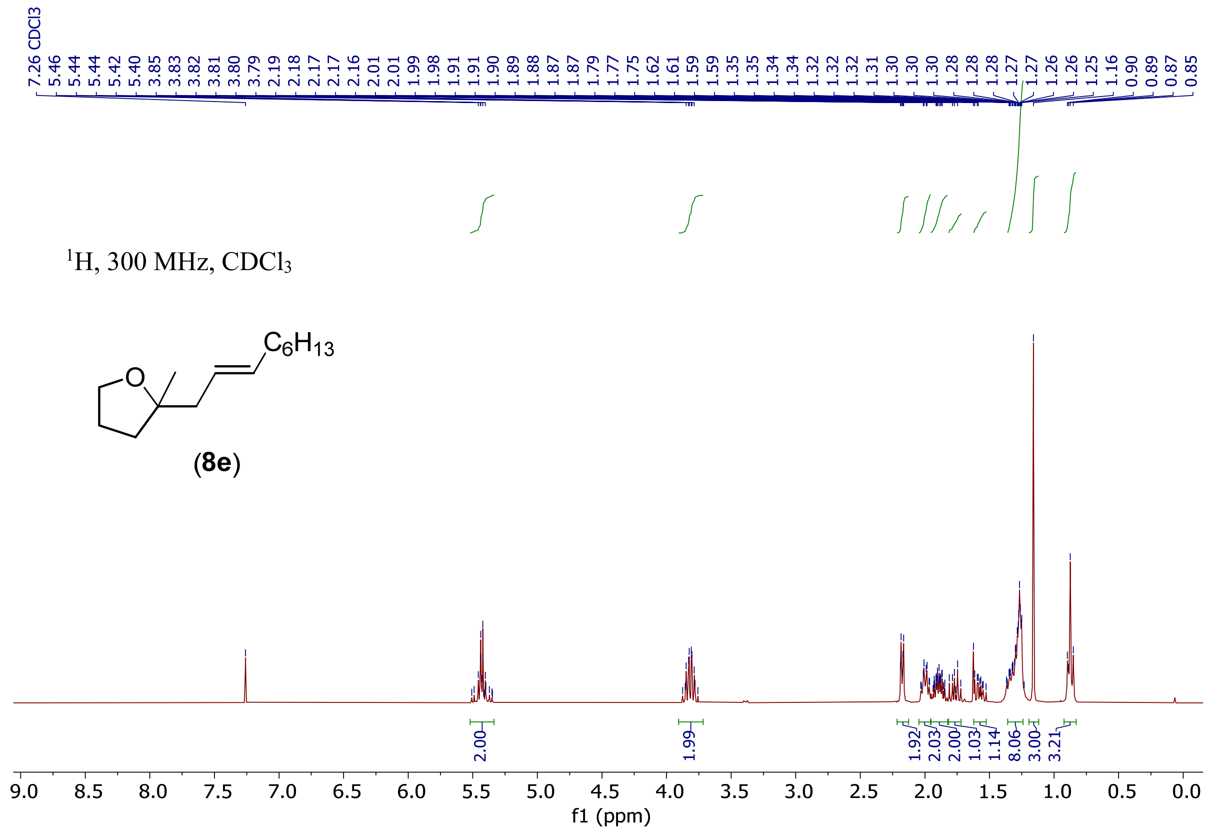

7.26 CDCl3
5.46
5.44
5.44
5.42
5.40
3.85
3.83
3.82
3.81
3.80
3.79
2.19
2.18
2.17
2.17
2.16
2.01
2.01
1.99
1.98
1.91
1.91
1.90
1.89
1.88
1.87
1.87
1.79
1.77
1.75
1.62
1.61
1.59
1.59
1.35
1.35
1.34
1.34
1.32
1.32
1.32
1.31
1.30
1.30
1.30
1.28
1.28
1.28
1.27
1.27
1.26
1.26
1.25
1.16
0.90
0.89
0.87
0.85
¹H, 300 MHz, CDCl₃
C6H13
O
(8e)
2.00
1.99
1.92
2.03
2.00
1.03
1.14
8.06
3.00
3.21
9.0
8.5
8.0
7.5
7.0
6.5
6.0
5.5
5.0
4.5
4.0
3.5
3.0
2.5
2.0
1.5
1.0
0.5
0.0
f1 (ppm)

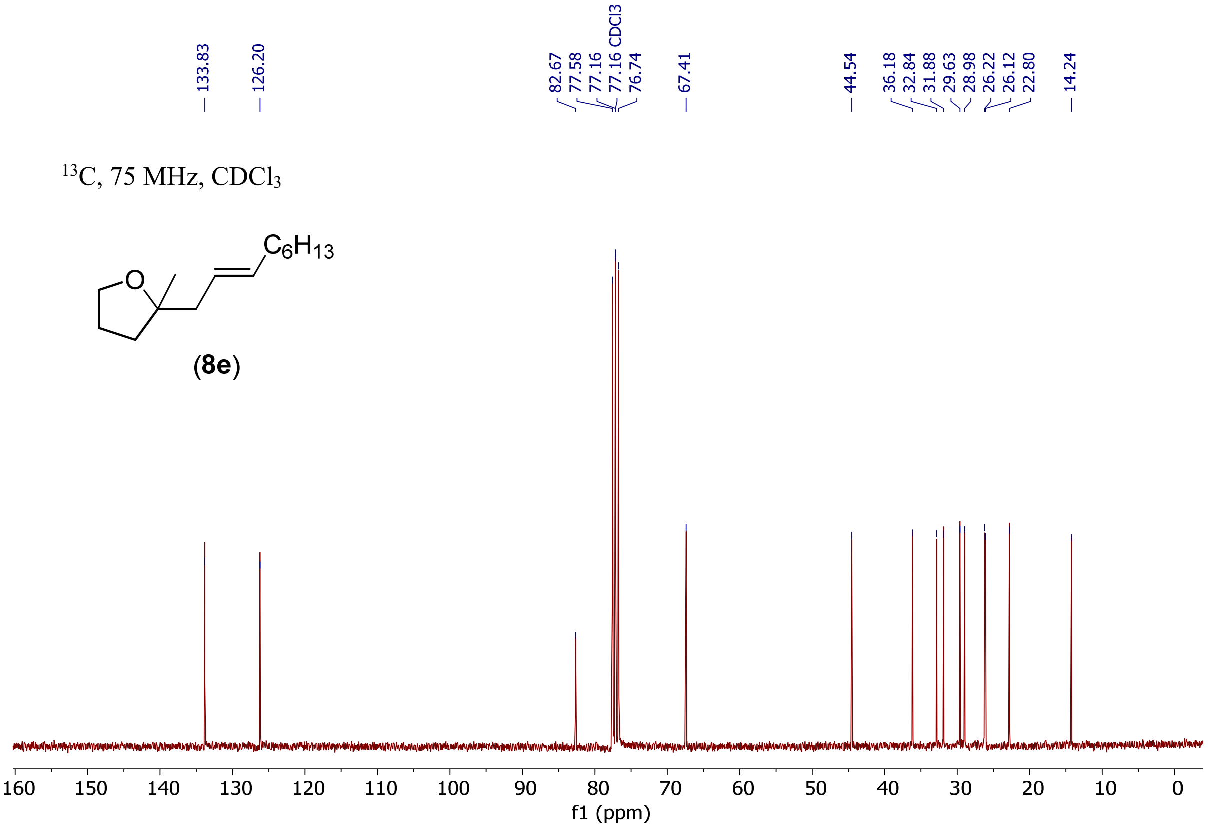

133.83
126.20
82.67
77.58
77.16
77.16 CDCl3
76.74
67.41
44.54
36.18
32.84
31.88
29.63
28.98
26.22
26.12
22.80
14.24
¹³C, 75 MHz, CDCl₃
C6H13
O
(8e)
160
150
140
130
120
110
100
90
80
70
60
50
40
30
20
10
0
f1 (ppm)

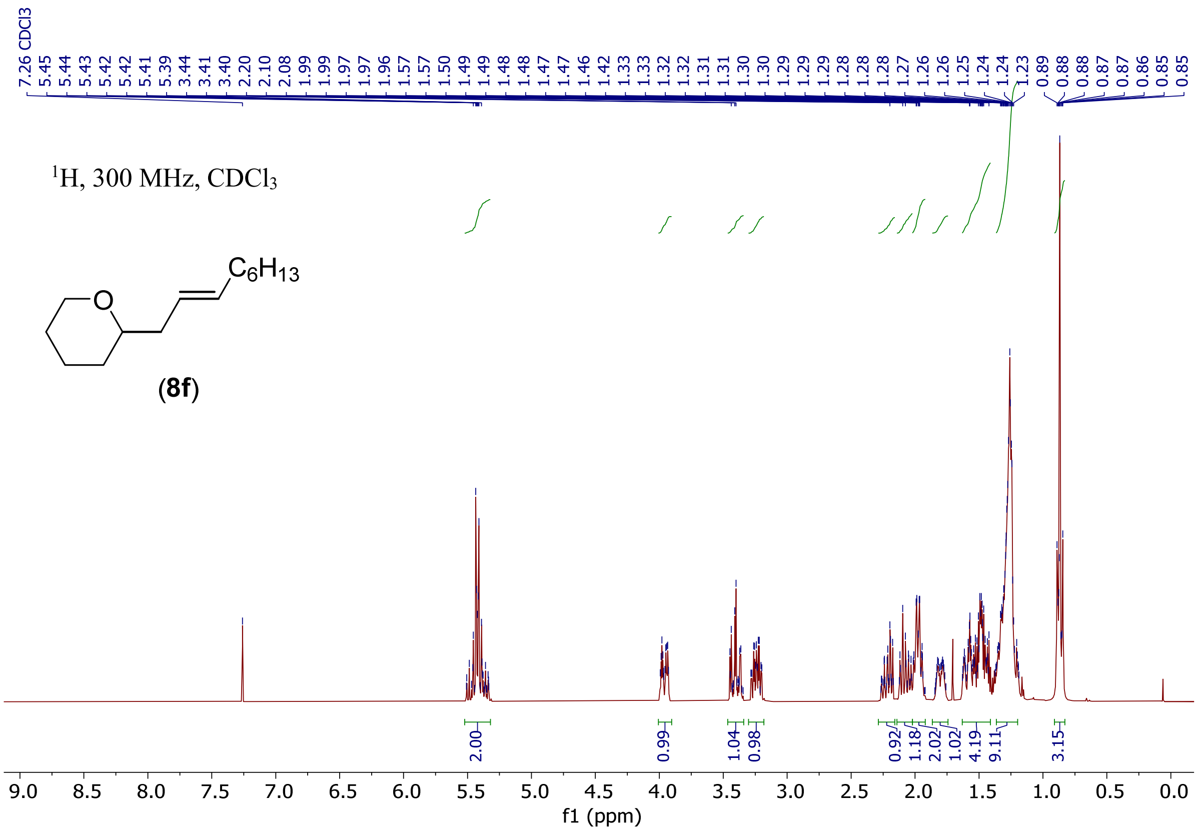
7.26 CDCl3
5.45
5.44
5.43
5.42
5.42
5.41
5.39
3.44
3.41
3.40
2.20
2.10
2.08
1.99
1.99
1.97
1.97
1.96
1.57
1.57
1.50
1.49
1.49
1.48
1.48
1.47
1.47
1.46
1.42
1.33
1.33
1.32
1.32
1.31
1.31
1.30
1.30
1.29
1.29
1.29
1.28
1.28
1.28
1.27
1.26
1.26
1.25
1.24
1.24
1.23
0.89
0.88
0.88
0.87
0.87
0.86
0.85
0.85
^{1}H, 300 MHz, CDCl$_3$
C_6H_{13}
O
(**8f**)
2.00
0.99
1.04
0.98
0.92
1.18
2.02
1.02
4.19
9.11
3.15
9.0 8.5 8.0 7.5 7.0 6.5 6.0 5.5 5.0 4.5 4.0 3.5 3.0 2.5 2.0 1.5 1.0 0.5 0.0
f1 (ppm)

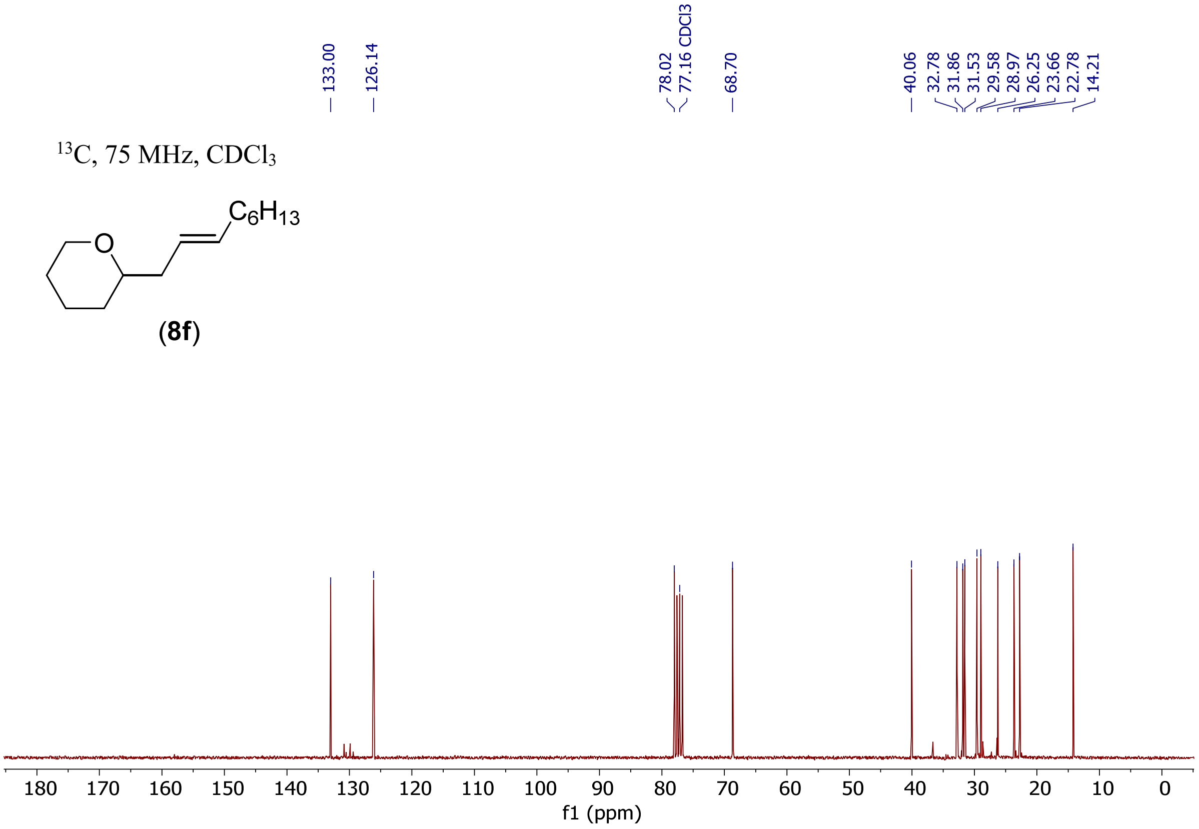
133.00
126.14
78.02
77.16 CDCl3
68.70
40.06
32.78
31.86
31.53
29.58
28.97
26.25
23.66
22.78
14.21
^{13}C, 75 MHz, CDCl$_3$
C_6H_{13}
O
(**8f**)
180 170 160 150 140 130 120 110 100 90 80 70 60 50 40 30 20 10 0
f1 (ppm)

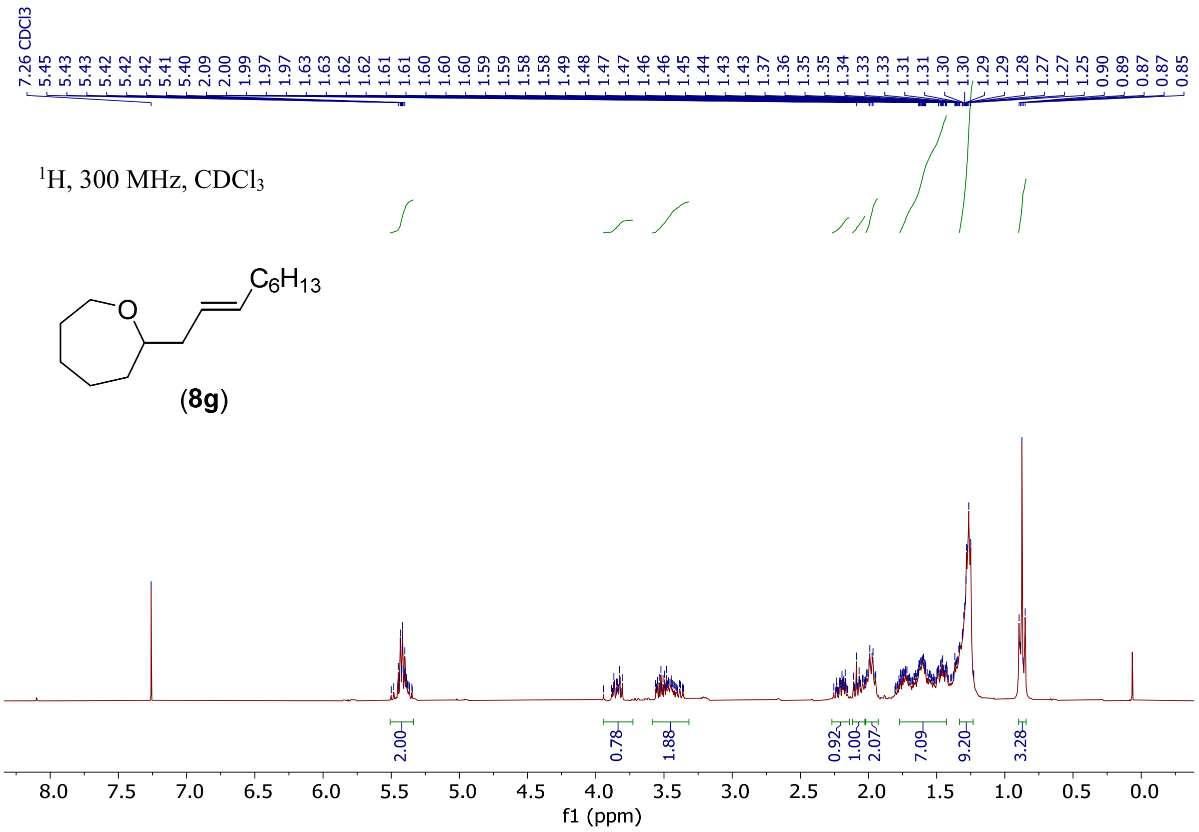
7.26 CDCl3
5.45
5.43
5.43
5.42
5.42
5.42
5.41
5.40
2.09
2.00
1.99
1.97
1.97
1.63
1.63
1.62
1.62
1.61
1.61
1.60
1.60
1.60
1.59
1.59
1.58
1.58
1.49
1.48
1.47
1.47
1.46
1.46
1.45
1.44
1.43
1.43
1.37
1.36
1.35
1.35
1.34
1.33
1.33
1.31
1.31
1.30
1.30
1.29
1.29
1.28
1.27
1.27
1.25
0.90
0.89
0.87
0.87
0.85
^{1}H, 300 MHz, CDCl$_3$
C$_6$H$_{13}$
O
(8g)
2.00
0.78
1.88
0.92
1.00
2.07
7.09
9.20
3.28
8.0
7.5
7.0
6.5
6.0
5.5
5.0
4.5
4.0
3.5
3.0
2.5
2.0
1.5
1.0
0.5
0.0
f1 (ppm)

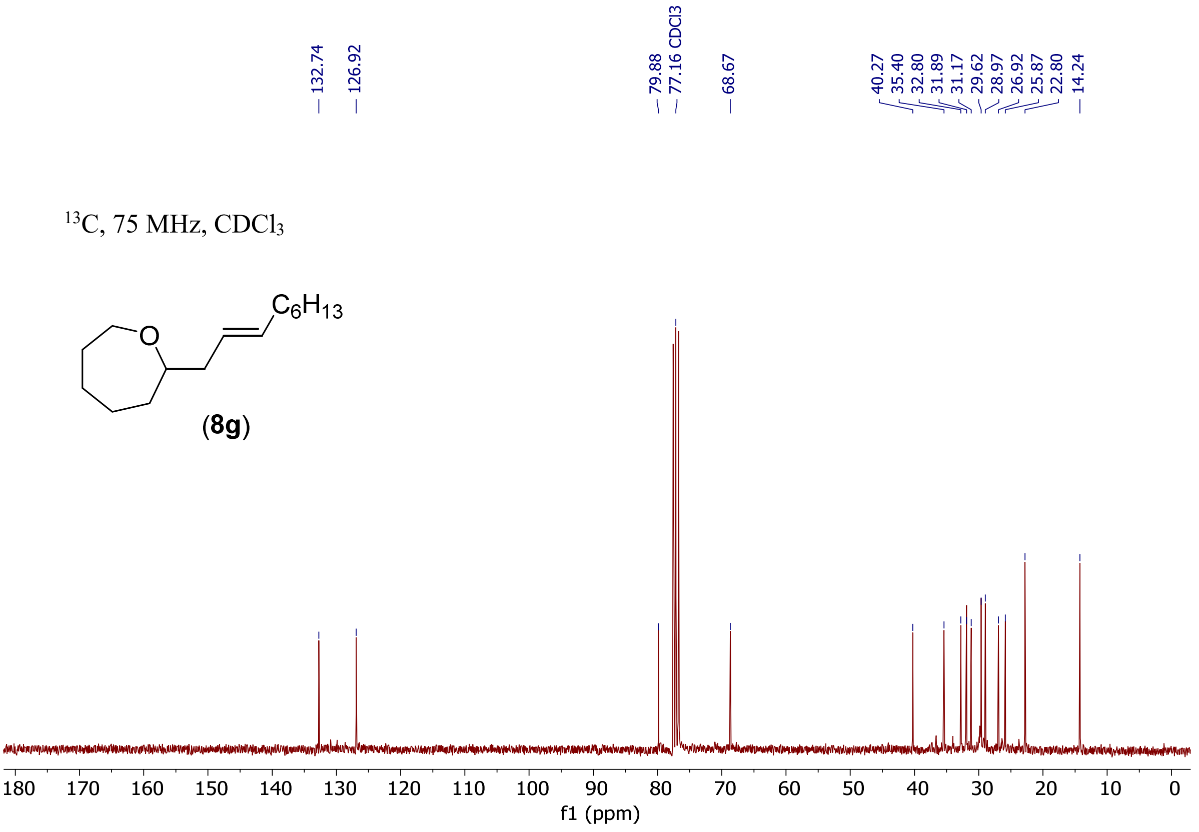
132.74
126.92
79.88
77.16 CDCl3
68.67
40.27
35.40
32.80
31.89
31.17
29.62
28.97
26.92
25.87
22.80
14.24
^{13}C, 75 MHz, CDCl$_3$
C$_6$H$_{13}$
O
(8g)
180
170
160
150
140
130
120
110
100
90
80
70
60
50
40
30
20
10
0
f1 (ppm)

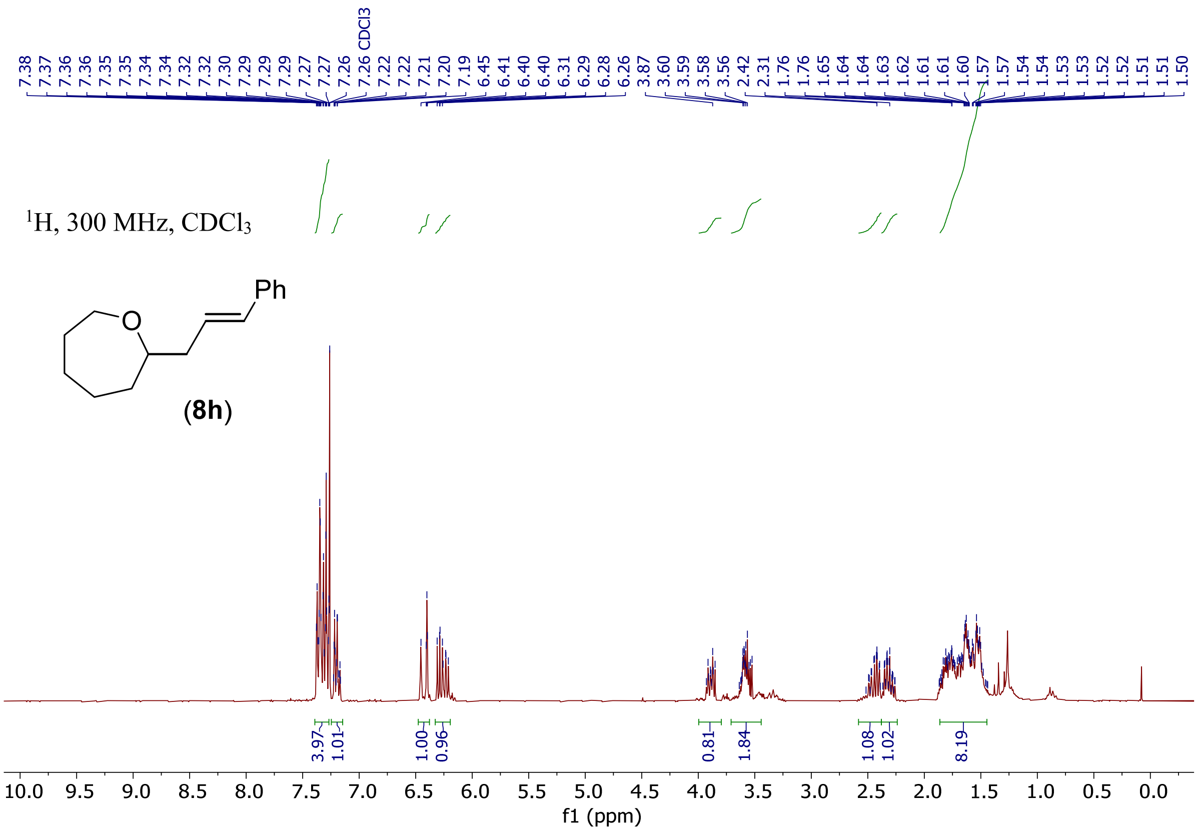
7.38 7.37 7.36 7.36 7.35 7.35 7.34 7.34 7.32 7.32 7.30 7.29 7.29 7.29 7.27 7.27 7.26 7.26 CDCl3 7.22 7.22 7.21 7.20 7.19 6.45 6.41 6.40 6.40 6.31 6.29 6.28 6.26 3.87 3.60 3.59 3.58 3.56 2.42 2.31 1.76 1.76 1.65 1.64 1.64 1.63 1.62 1.61 1.61 1.60 1.57 1.57 1.54 1.54 1.53 1.53 1.52 1.52 1.51 1.51 1.50
1H, 300 MHz, CDCl3
Ph
O
(8h)
3.97 1.01 1.00 0.96 0.81 1.84 1.08 1.02 8.19
10.0 9.5 9.0 8.5 8.0 7.5 7.0 6.5 6.0 5.5 5.0 4.5 4.0 3.5 3.0 2.5 2.0 1.5 1.0 0.5 0.0
f1 (ppm)

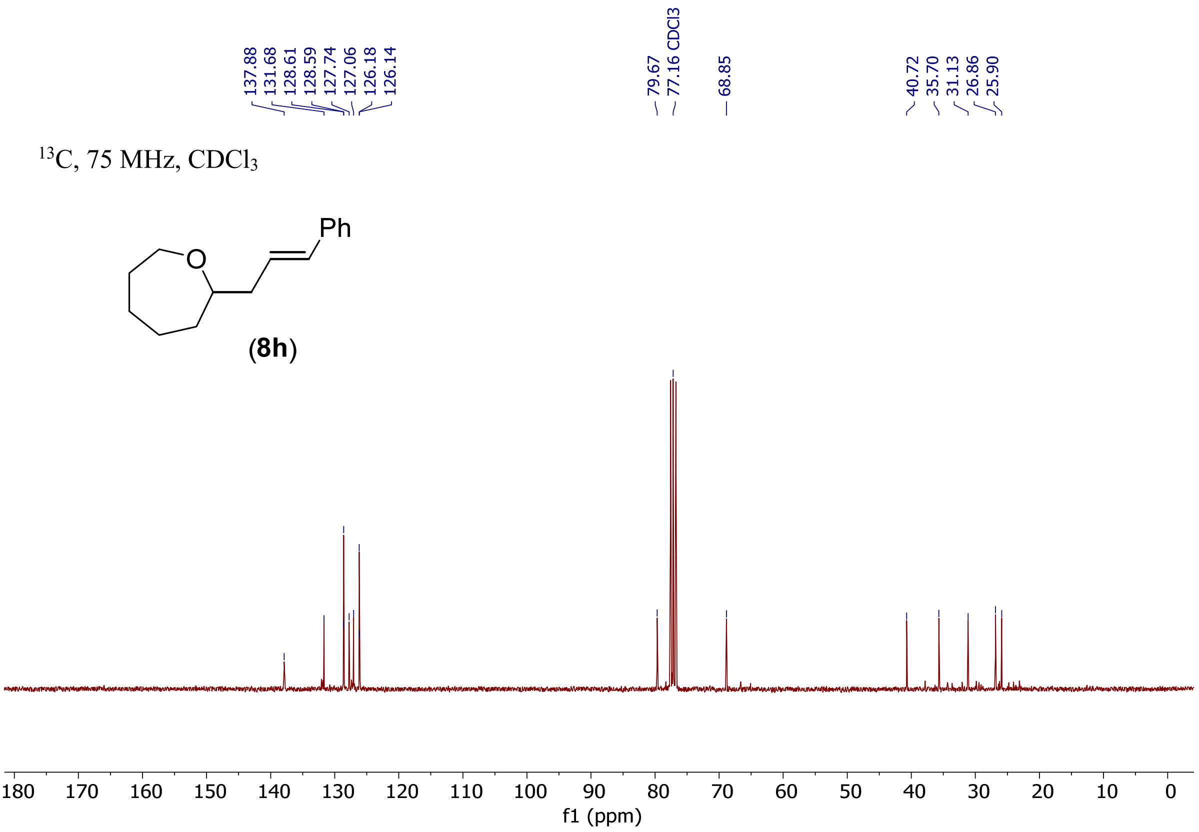
137.88 131.68 128.61 128.59 127.74 127.06 126.18 126.14
79.67 77.16 CDCl3
68.85
40.72 35.70 31.13 26.86 25.90
13C, 75 MHz, CDCl3
Ph
O
(8h)
180 170 160 150 140 130 120 110 100 90 80 70 60 50 40 30 20 10 0
f1 (ppm)

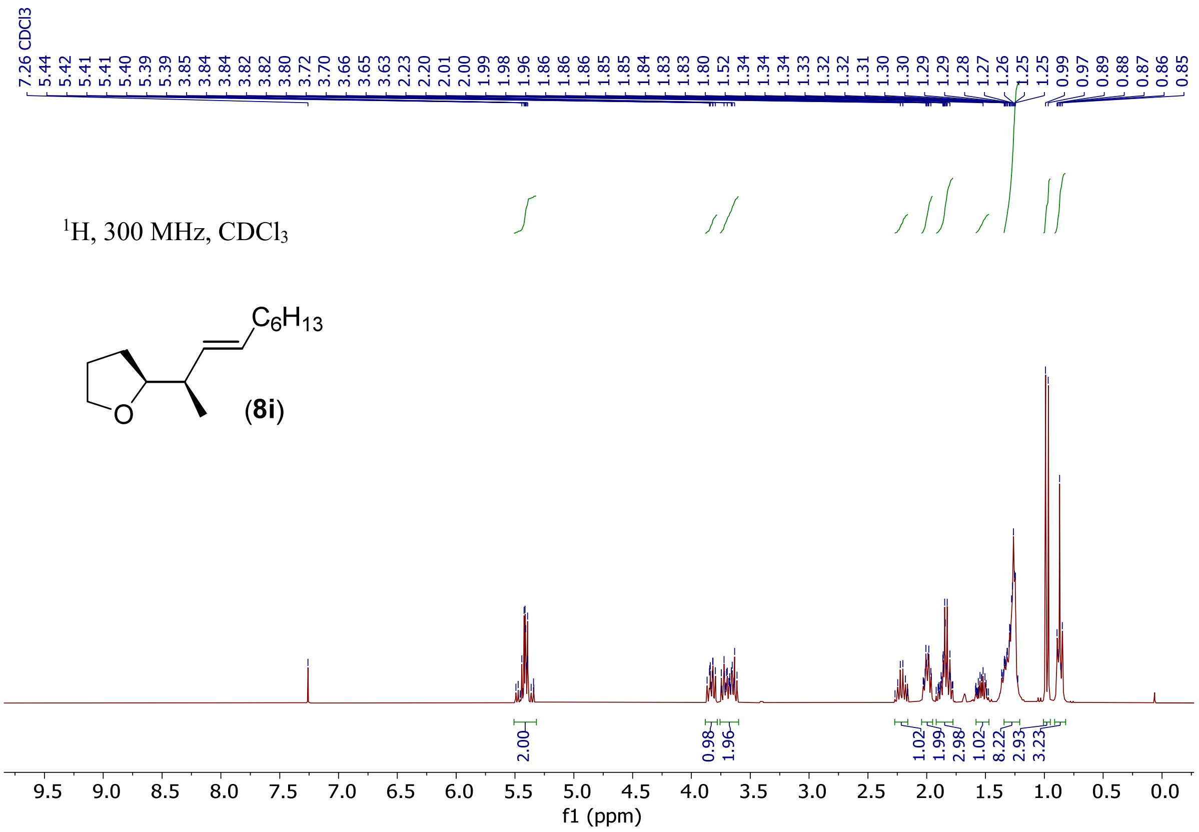
7.26 CDCl3
5.44
5.42
5.41
5.41
5.40
5.39
5.39
3.85
3.84
3.84
3.82
3.82
3.80
3.72
3.70
3.66
3.65
3.63
2.23
2.20
2.01
2.00
1.99
1.98
1.96
1.86
1.86
1.86
1.85
1.85
1.84
1.83
1.83
1.80
1.52
1.34
1.34
1.34
1.33
1.32
1.32
1.31
1.30
1.30
1.29
1.29
1.28
1.27
1.26
1.25
1.25
0.99
0.97
0.89
0.88
0.87
0.86
0.85
^{1}H, 300 MHz, CDCl$_3$
C$_6$H$_{13}$
O
(**8i**)
2.00
0.98
1.96
1.02
1.99
2.98
1.02
8.22
2.93
3.23
9.5 9.0 8.5 8.0 7.5 7.0 6.5 6.0 5.5 5.0 4.5 4.0 3.5 3.0 2.5 2.0 1.5 1.0 0.5 0.0
f1 (ppm)

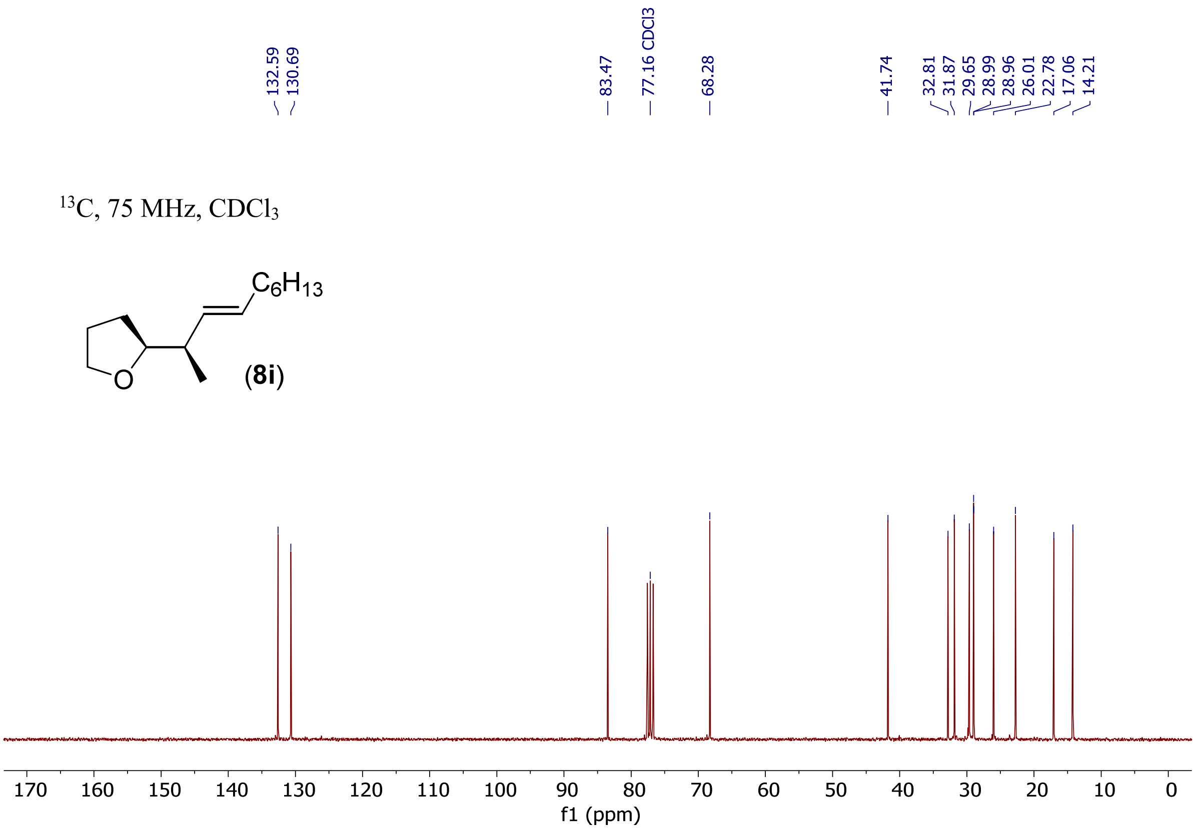
132.59
130.69
83.47
77.16 CDCl3
68.28
41.74
32.81
31.87
29.65
28.99
28.96
26.01
22.78
17.06
14.21
^{13}C, 75 MHz, CDCl$_3$
C$_6$H$_{13}$
O
(**8i**)
170 160 150 140 130 120 110 100 90 80 70 60 50 40 30 20 10 0
f1 (ppm)

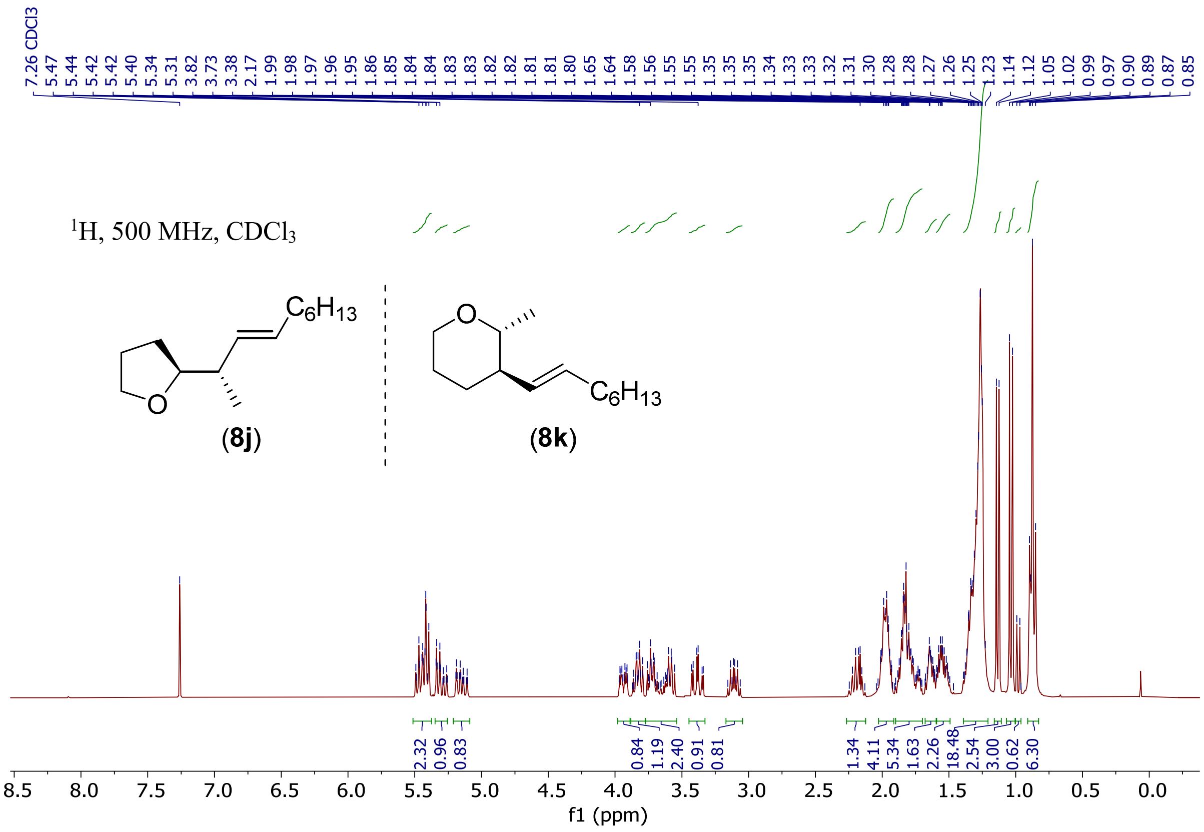
7.26 CDCl3
5.47 5.44 5.42 5.42 5.40 5.34 5.31 3.82 3.73 3.38 2.17 1.99 1.98 1.97 1.96 1.95 1.86 1.85 1.84 1.84 1.83 1.83 1.82 1.82 1.81 1.81 1.80 1.65 1.64 1.58 1.56 1.55 1.55 1.35 1.35 1.35 1.34 1.33 1.33 1.32 1.31 1.30 1.28 1.28 1.27 1.26 1.25 1.23 1.14 1.12 1.05 1.02 0.99 0.97 0.90 0.89 0.87 0.85
1H, 500 MHz, CDCl3
C6H13
(8j)
O
C6H13
(8k)
2.32 0.96 0.83 0.84 1.19 2.40 0.91 0.81 1.34 4.11 5.34 1.63 2.26 18.48 2.54 3.00 0.62 6.30
8.5 8.0 7.5 7.0 6.5 6.0 5.5 5.0 4.5 4.0 3.5 3.0 2.5 2.0 1.5 1.0 0.5 0.0
f1 (ppm)

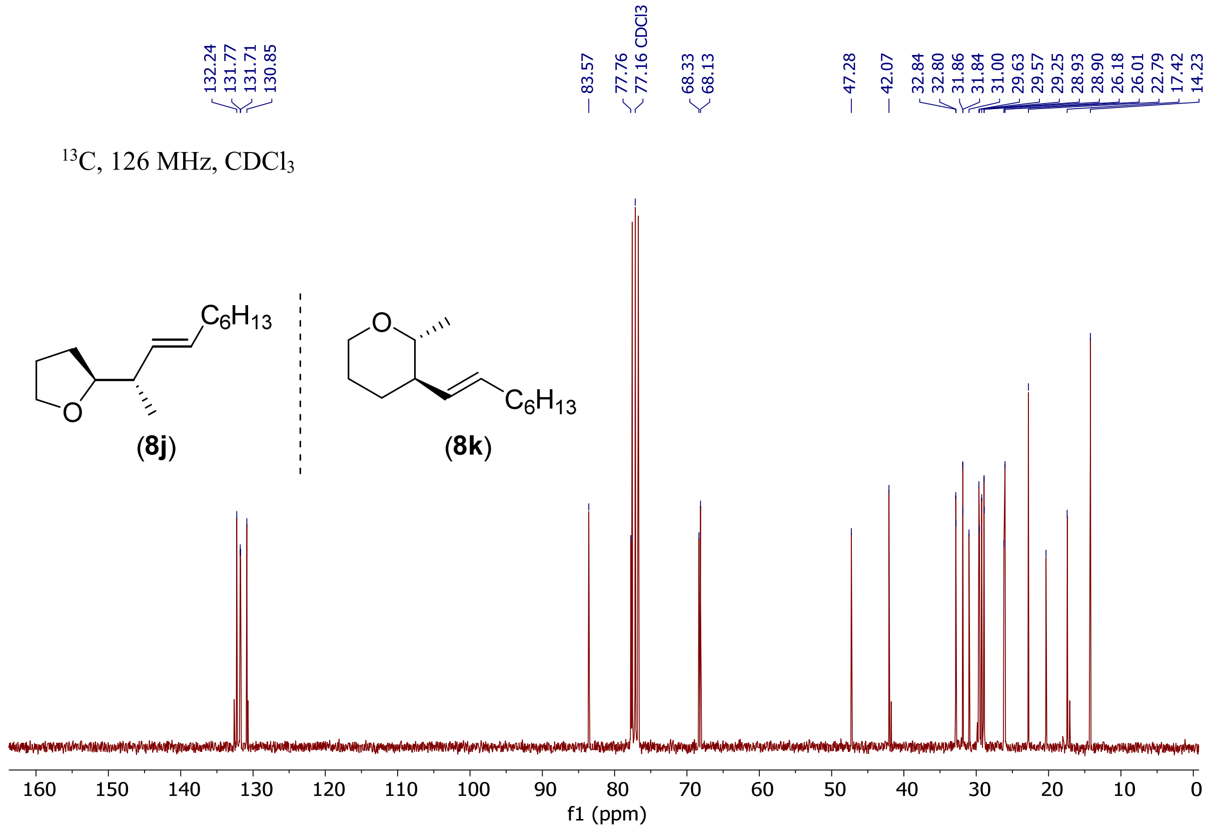
132.24 131.77 131.71 130.85
83.57
77.76 77.16 CDCl3
68.33 68.13
47.28
42.07
32.84 32.80 31.86 31.84 31.00 29.63 29.57 29.25 28.93 28.90 26.18 26.01 22.79 17.42 14.23
13C, 126 MHz, CDCl3
C6H13
(8j)
O
C6H13
(8k)
160 150 140 130 120 110 100 90 80 70 60 50 40 30 20 10 0
f1 (ppm)

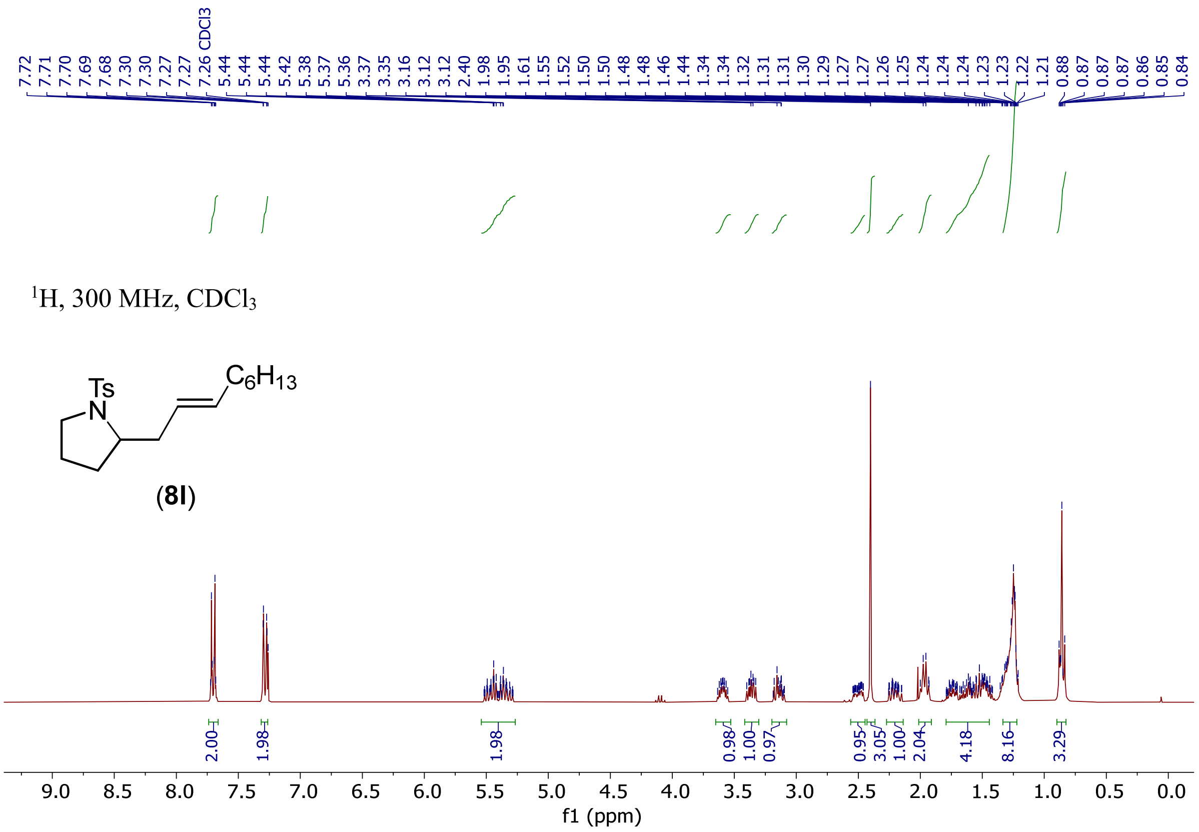
7.72 7.71 7.70 7.69 7.68 7.30 7.30 7.27 7.27 7.26 CDCl3 5.44 5.44 5.44 5.42 5.38 5.37 5.36 3.37 3.35 3.16 3.12 3.12 2.40 1.98 1.95 1.61 1.55 1.52 1.50 1.50 1.48 1.48 1.46 1.44 1.34 1.34 1.32 1.31 1.31 1.30 1.29 1.27 1.27 1.26 1.25 1.24 1.24 1.24 1.23 1.23 1.22 1.21 0.88 0.87 0.87 0.86 0.85 0.84
^{1}H, 300 MHz, $CDCl_3$
Ts
N
C_6H_{13}
(8l)
2.00 1.98 1.98 0.98 1.00 0.97 0.95 3.05 1.00 2.04 4.18 8.16 3.29
9.0 8.5 8.0 7.5 7.0 6.5 6.0 5.5 5.0 4.5 4.0 3.5 3.0 2.5 2.0 1.5 1.0 0.5 0.0
f1 (ppm)

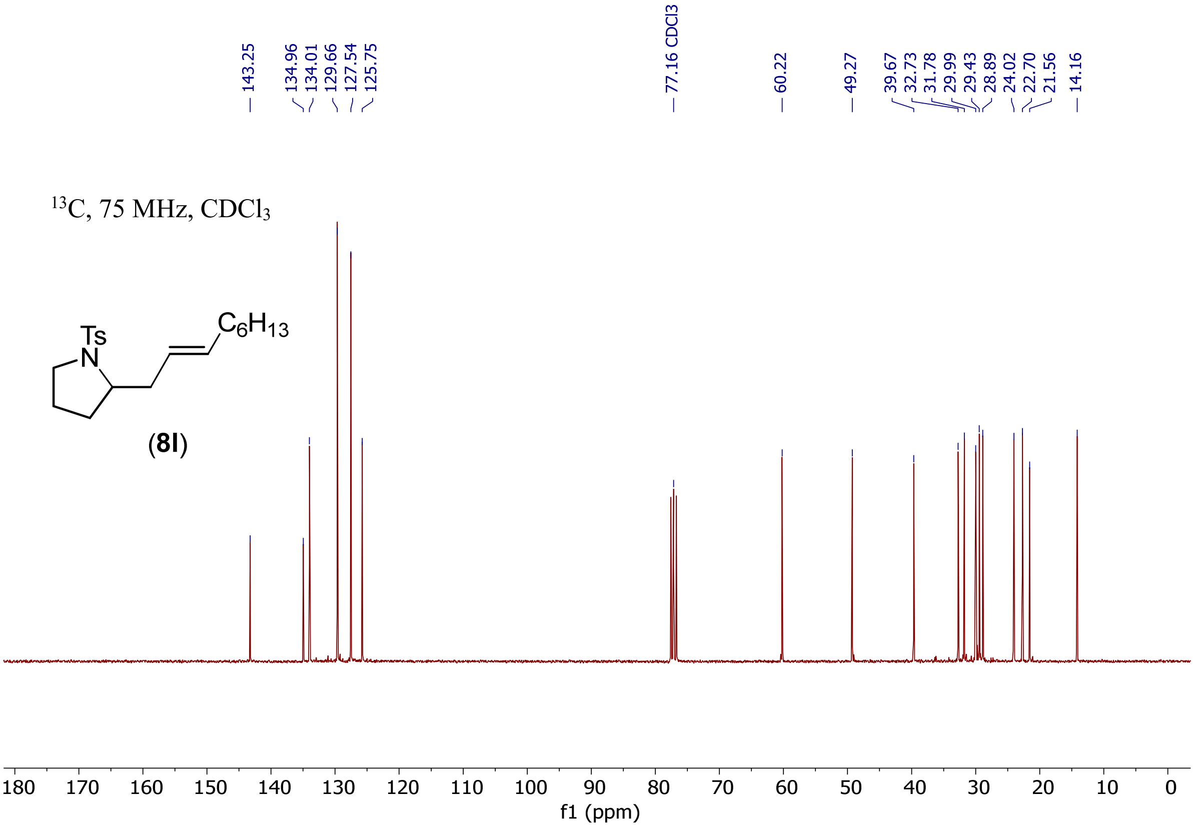
143.25 134.96 134.01 129.66 127.54 125.75 77.16 CDCl3 60.22 49.27 39.67 32.73 31.78 29.99 29.43 28.89 24.02 22.70 21.56 14.16
^{13}C, 75 MHz, $CDCl_3$
Ts
N
C_6H_{13}
(8l)
180 170 160 150 140 130 120 110 100 90 80 70 60 50 40 30 20 10 0
f1 (ppm)

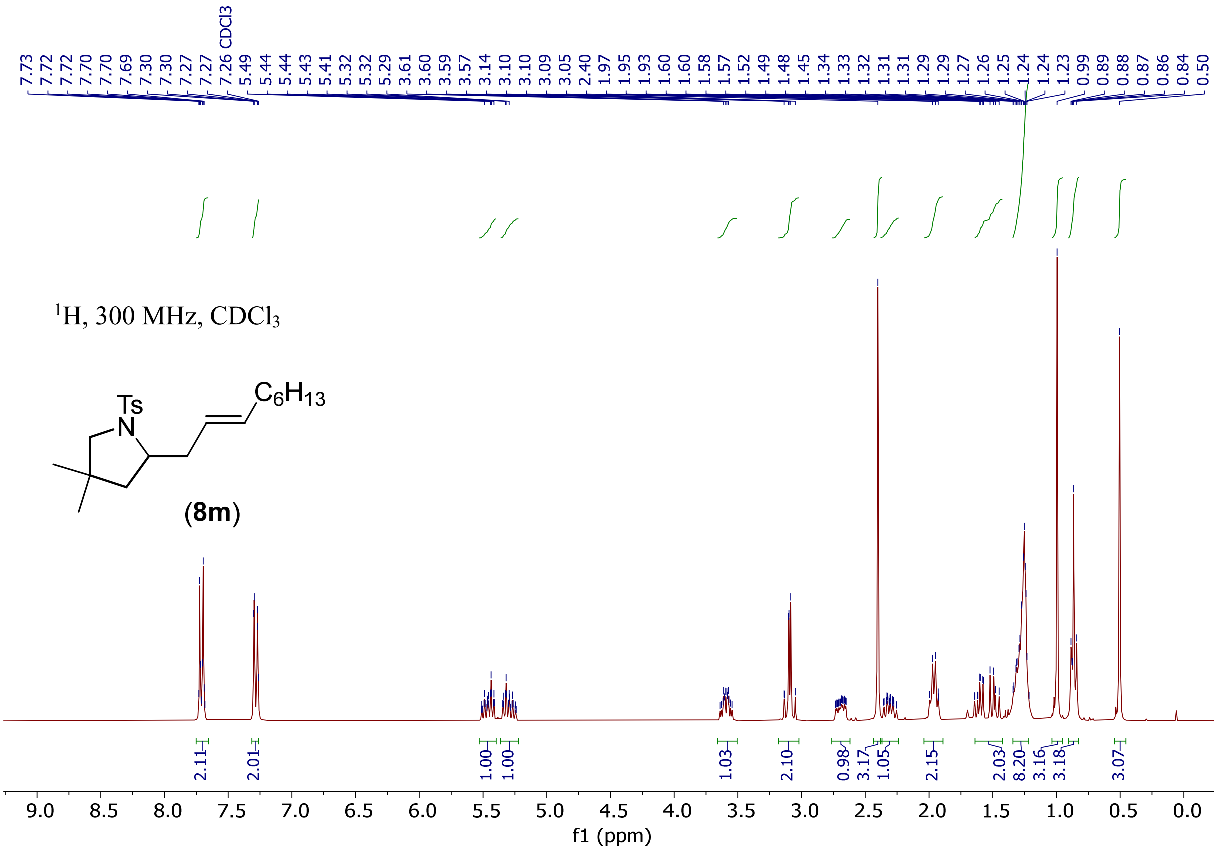
7.73 7.72 7.72 7.70 7.70 7.69 7.30 7.30 7.27 7.27 7.26 CDCl3 5.49 5.44 5.44 5.43 5.41 5.32 5.32 5.29 3.61 3.60 3.59 3.57 3.14 3.10 3.09 3.05 2.40 1.97 1.95 1.93 1.60 1.60 1.58 1.57 1.52 1.49 1.48 1.45 1.34 1.33 1.32 1.31 1.31 1.29 1.29 1.27 1.26 1.25 1.24 1.24 1.23 0.99 0.89 0.88 0.87 0.86 0.84 0.50
1H, 300 MHz, CDCl3
Ts
N
C6H13
(8m)
2.11 2.01 1.00 1.00 1.03 2.10 0.98 3.17 1.05 2.15 2.03 8.20 3.16 3.18 3.07
9.0 8.5 8.0 7.5 7.0 6.5 6.0 5.5 5.0 4.5 4.0 3.5 3.0 2.5 2.0 1.5 1.0 0.5 0.0
f1 (ppm)

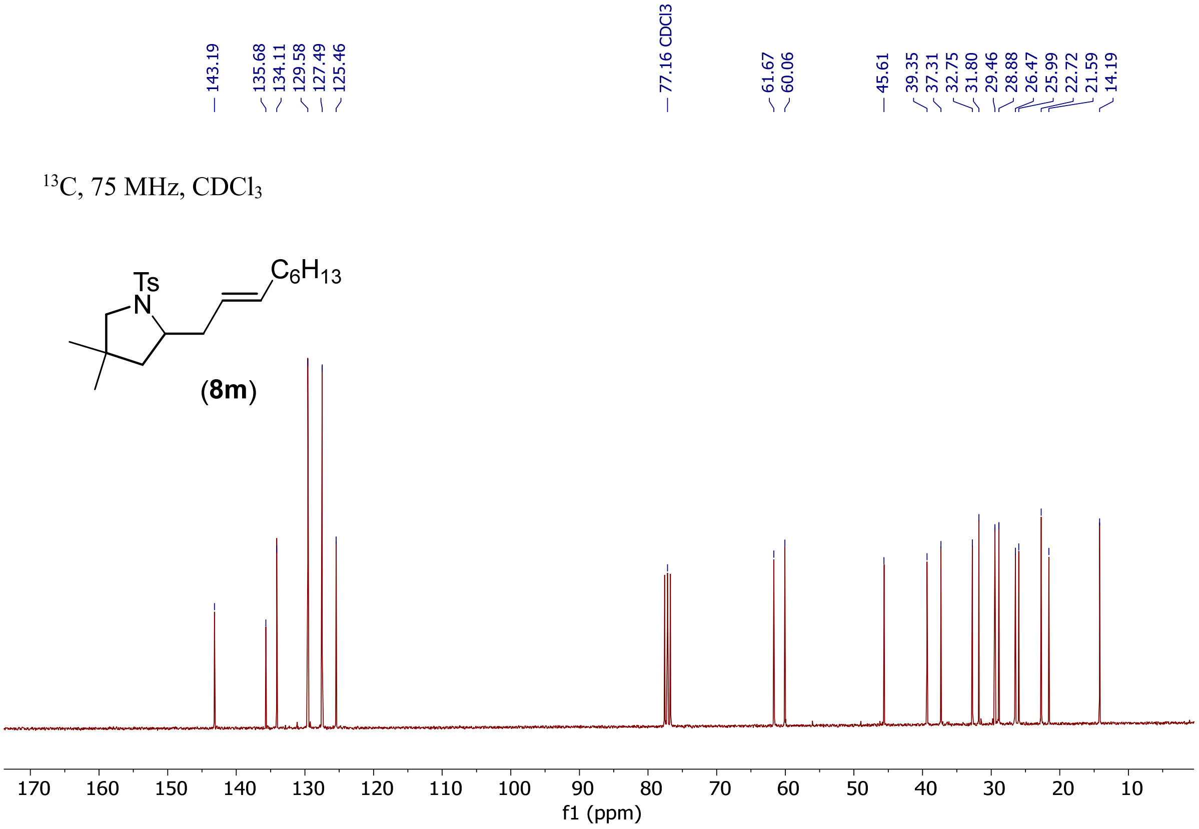
143.19 135.68 134.11 129.58 127.49 125.46 77.16 CDCl3 61.67 60.06 45.61 39.35 37.31 32.75 31.80 29.46 28.88 26.47 25.99 22.72 21.59 14.19
13C, 75 MHz, CDCl3
Ts
N
C6H13
(8m)
170 160 150 140 130 120 110 100 90 80 70 60 50 40 30 20 10
f1 (ppm)

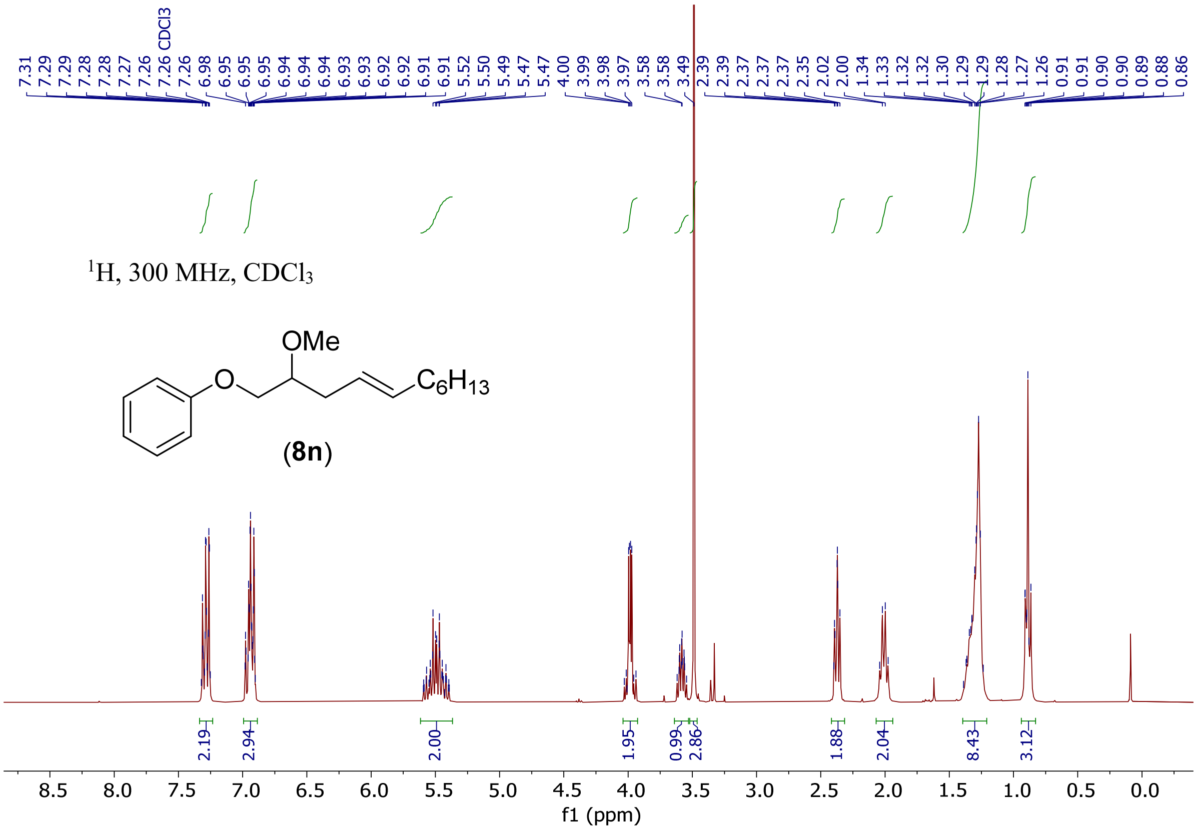
7.31 7.29 7.29 7.28 7.28 7.27 7.26 CDCl3 7.26 6.98 6.95 6.95 6.94 6.94 6.94 6.93 6.93 6.92 6.92 6.91 6.91 5.52 5.50 5.49 5.47 5.47 4.00 3.99 3.98 3.97 3.58 3.58 3.49 2.39 2.39 2.37 2.37 2.37 2.35 2.02 2.00 1.34 1.33 1.32 1.30 1.29 1.29 1.28 1.27 1.26 0.91 0.90 0.90 0.89 0.88 0.86
¹H, 300 MHz, CDCl3
OMe
O
C6H13
(8n)
2.19 2.94 2.00 1.95 0.99 2.86 1.88 2.04 8.43 3.12
8.5 8.0 7.5 7.0 6.5 6.0 5.5 5.0 4.5 4.0 3.5 3.0 2.5 2.0 1.5 1.0 0.5 0.0
f1 (ppm)

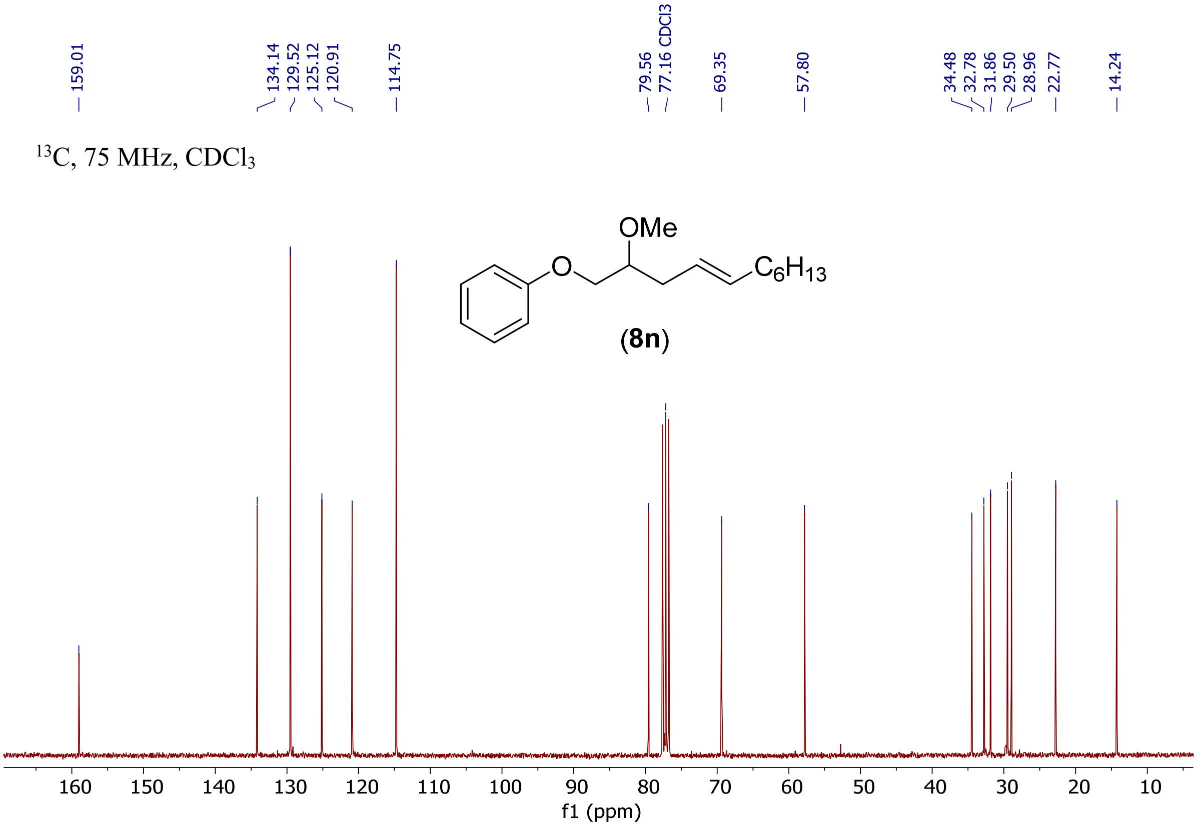
159.01 134.14 129.52 125.12 120.91 114.75 79.56 77.16 CDCl3 69.35 57.80 34.48 32.78 31.86 29.50 28.96 22.77 14.24
¹³C, 75 MHz, CDCl3
OMe
O
C6H13
(8n)
160 150 140 130 120 110 100 90 80 70 60 50 40 30 20 10
f1 (ppm)

5.4 NMR spectra of heterovinylation/heteroarylation products

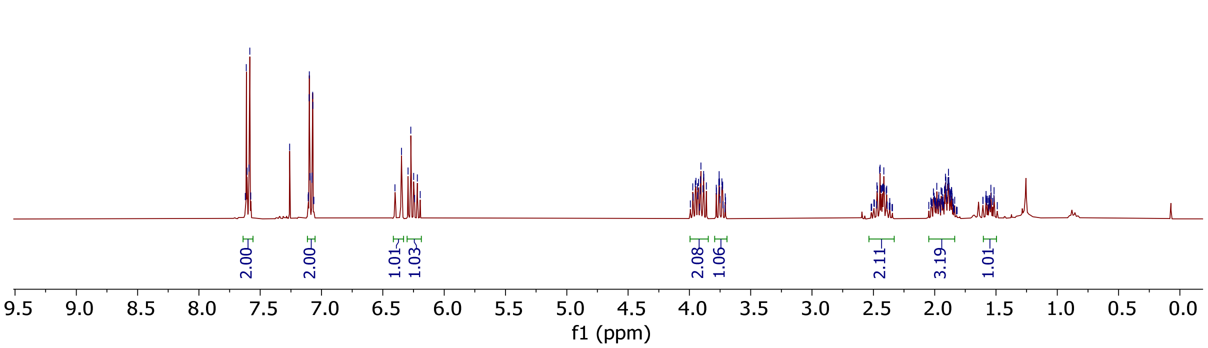

^{1}H, 300 MHz, $CDCl_3$

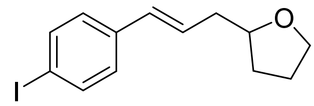

(**9a**)

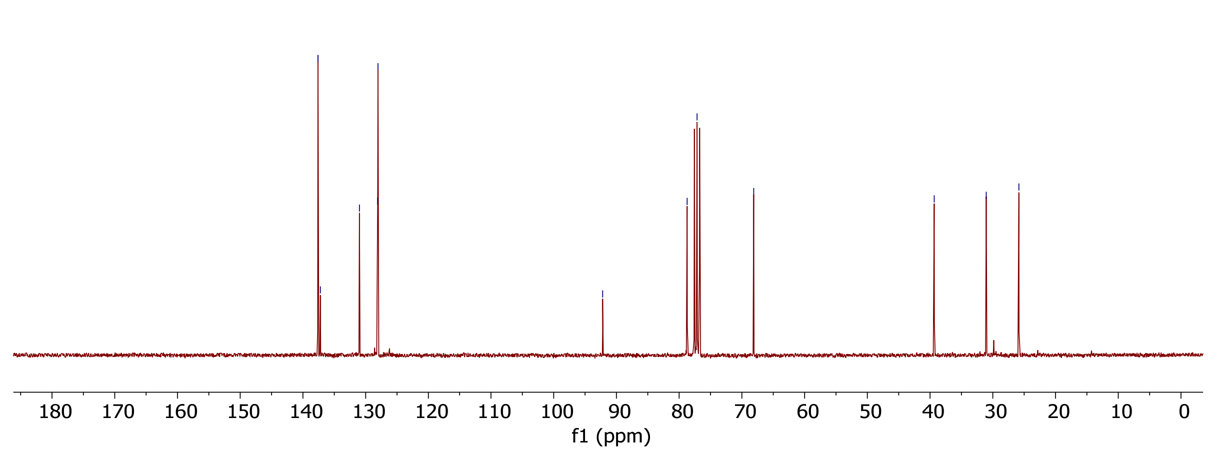

^{13}C, 75 MHz, $CDCl_3$

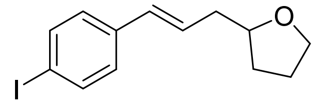

(**9a**)

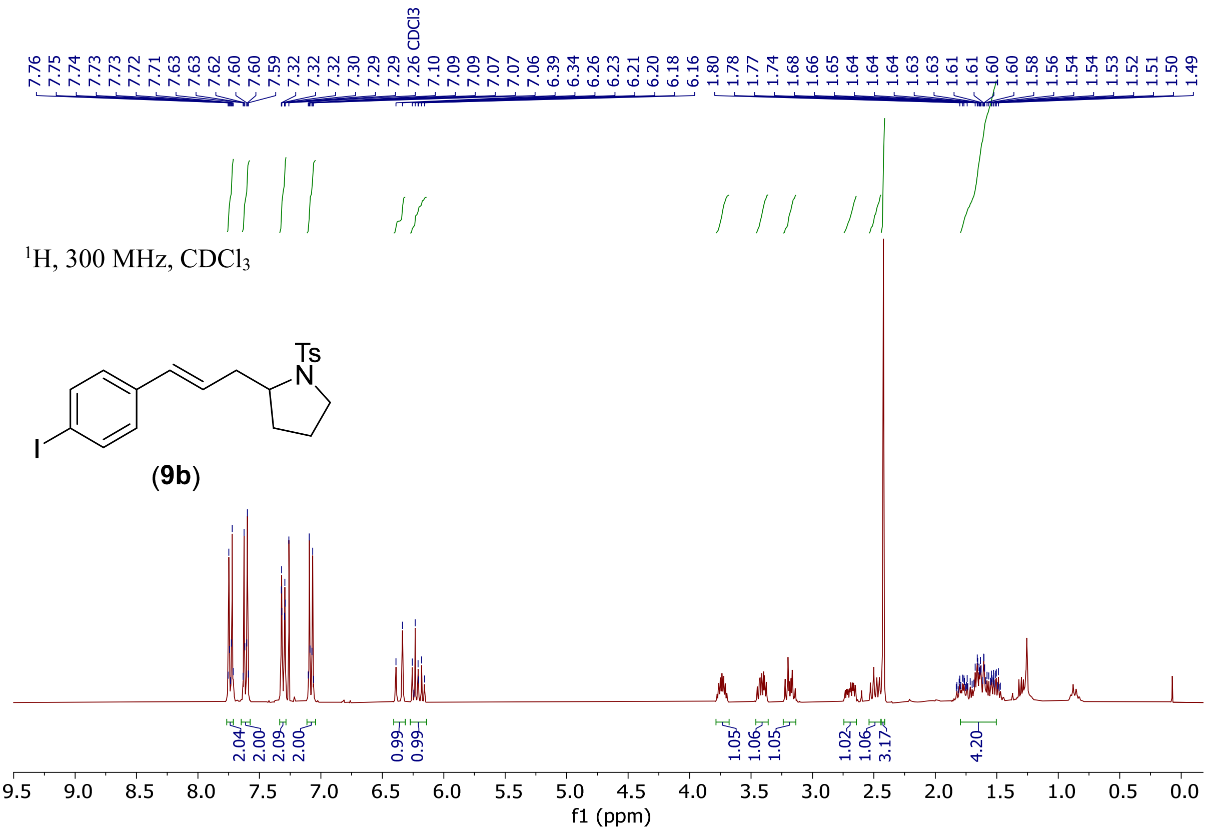
7.76 7.75 7.74 7.73 7.73 7.72 7.71 7.63 7.63 7.62 7.60 7.60 7.59 7.32 7.32 7.32 7.30 7.29 7.29 7.26 CDCl3 7.10 7.09 7.09 7.07 7.07 7.06 6.39 6.34 6.26 6.23 6.21 6.20 6.18 6.16 1.80 1.78 1.77 1.74 1.68 1.66 1.65 1.64 1.64 1.64 1.63 1.63 1.61 1.61 1.60 1.60 1.58 1.56 1.54 1.54 1.53 1.52 1.51 1.50 1.49
1H, 300 MHz, CDCl3
Ts
N
I
(9b)
2.04 2.00 2.09 2.00 0.99 0.99 1.05 1.06 1.05 1.02 1.06 3.17 4.20
9.5 9.0 8.5 8.0 7.5 7.0 6.5 6.0 5.5 5.0 4.5 4.0 3.5 3.0 2.5 2.0 1.5 1.0 0.5 0.0
f1 (ppm)

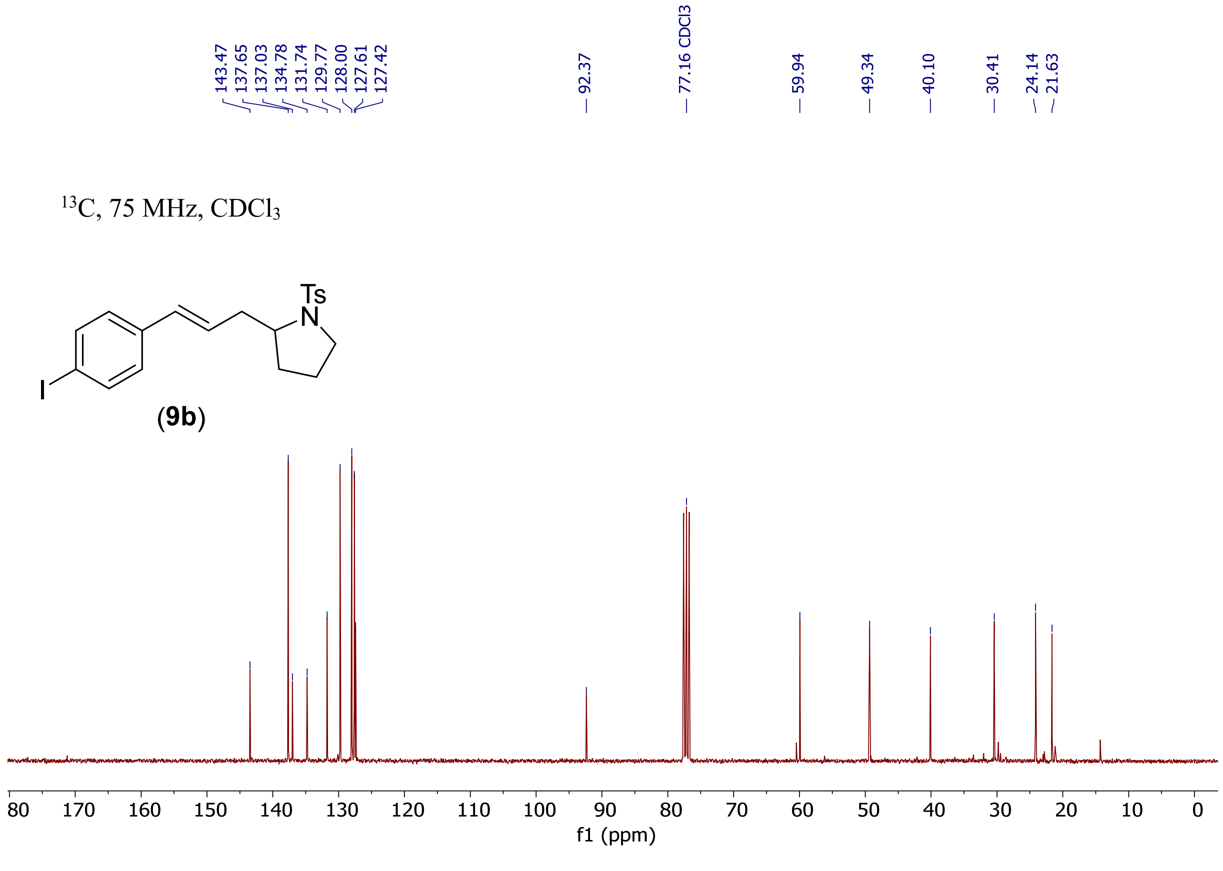
143.47 137.65 137.03 134.78 131.74 129.77 128.00 127.61 127.42
92.37
77.16 CDCl3
59.94
49.34
40.10
30.41
24.14 21.63
13C, 75 MHz, CDCl3
Ts
N
I
(9b)
80 170 160 150 140 130 120 110 100 90 80 70 60 50 40 30 20 10 0
f1 (ppm)

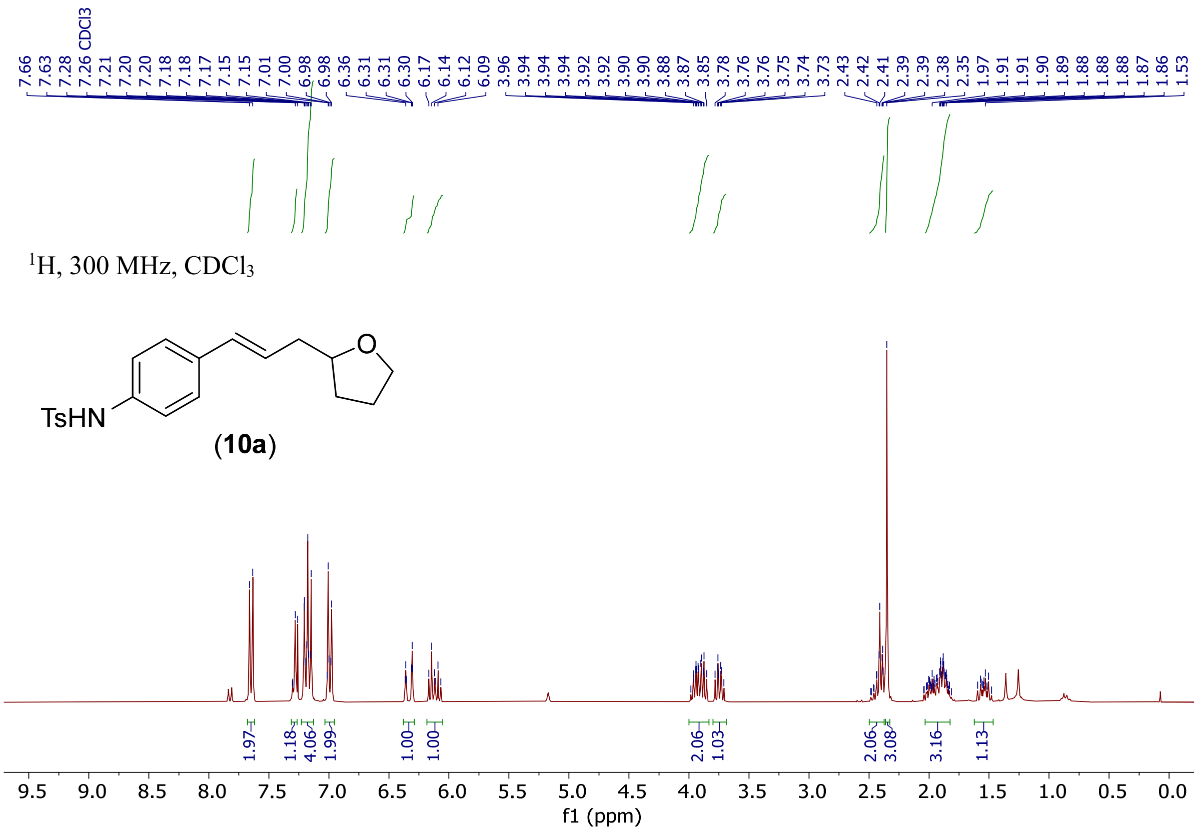

^{1}H, 300 MHz, $CDCl_3$

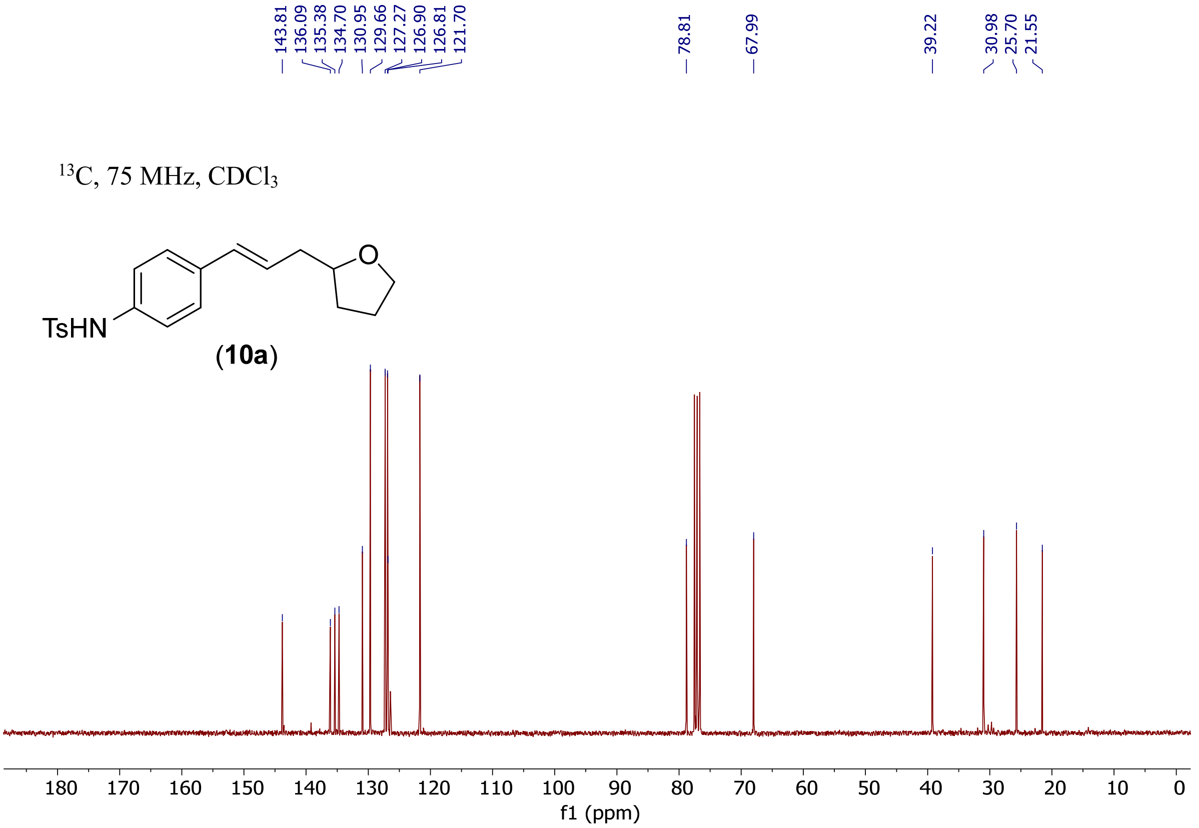

^{13}C, 75 MHz, $CDCl_3$

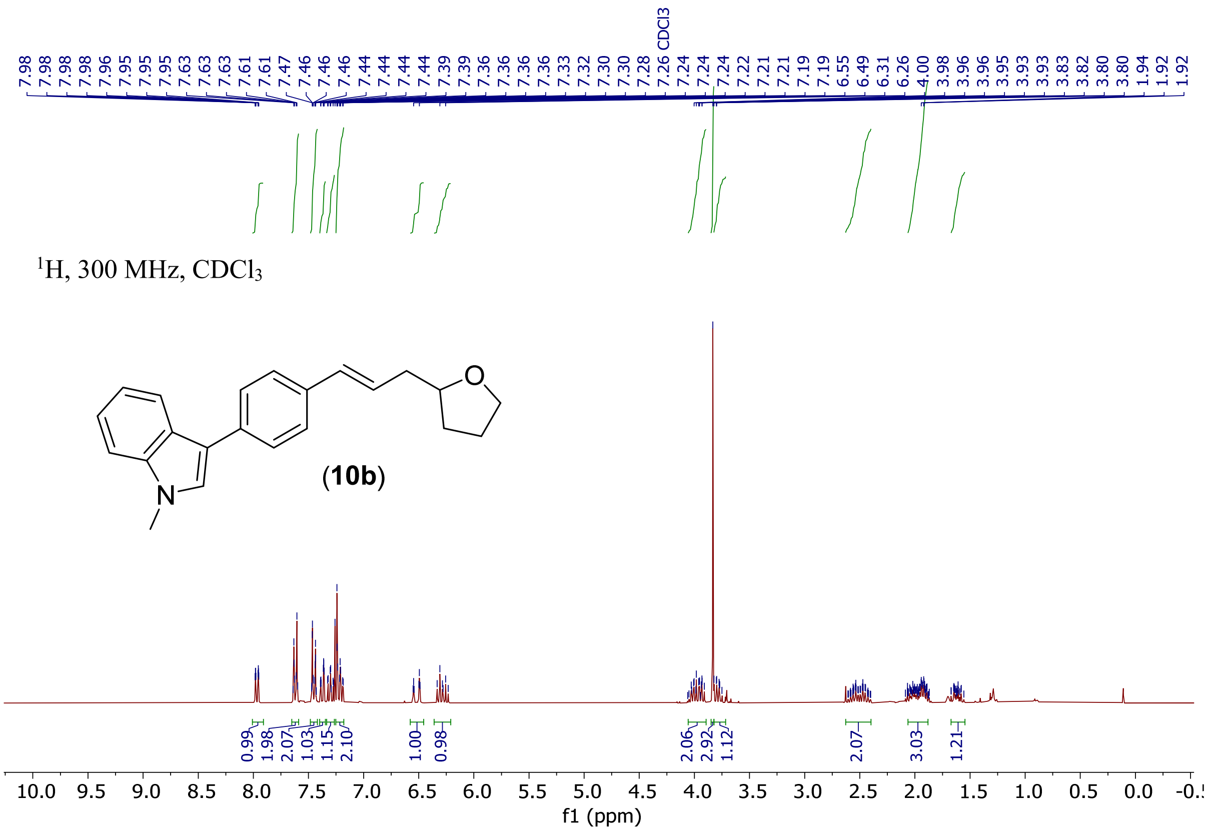
7.98 7.98 7.98 7.98 7.96 7.95 7.95 7.95 7.63 7.63 7.61 7.61 7.47 7.46 7.46 7.46 7.44 7.44 7.44 7.39 7.39 7.36 7.36 7.36 7.33 7.32 7.30 7.28 7.26 CDCl3 7.24 7.24 7.24 7.22 7.21 7.21 7.19 7.19 6.55 6.49 6.31 6.26 4.00 3.98 3.96 3.96 3.95 3.93 3.93 3.83 3.82 3.80 3.80 1.94 1.92 1.92
1H, 300 MHz, CDCl3
N
O
(10b)
0.99 1.98 2.07 1.03 1.15 2.10 1.00 0.98 2.06 2.92 1.12 2.07 3.03 1.21
10.0 9.5 9.0 8.5 8.0 7.5 7.0 6.5 6.0 5.5 5.0 4.5 4.0 3.5 3.0 2.5 2.0 1.5 1.0 0.5 0.0
f1 (ppm)

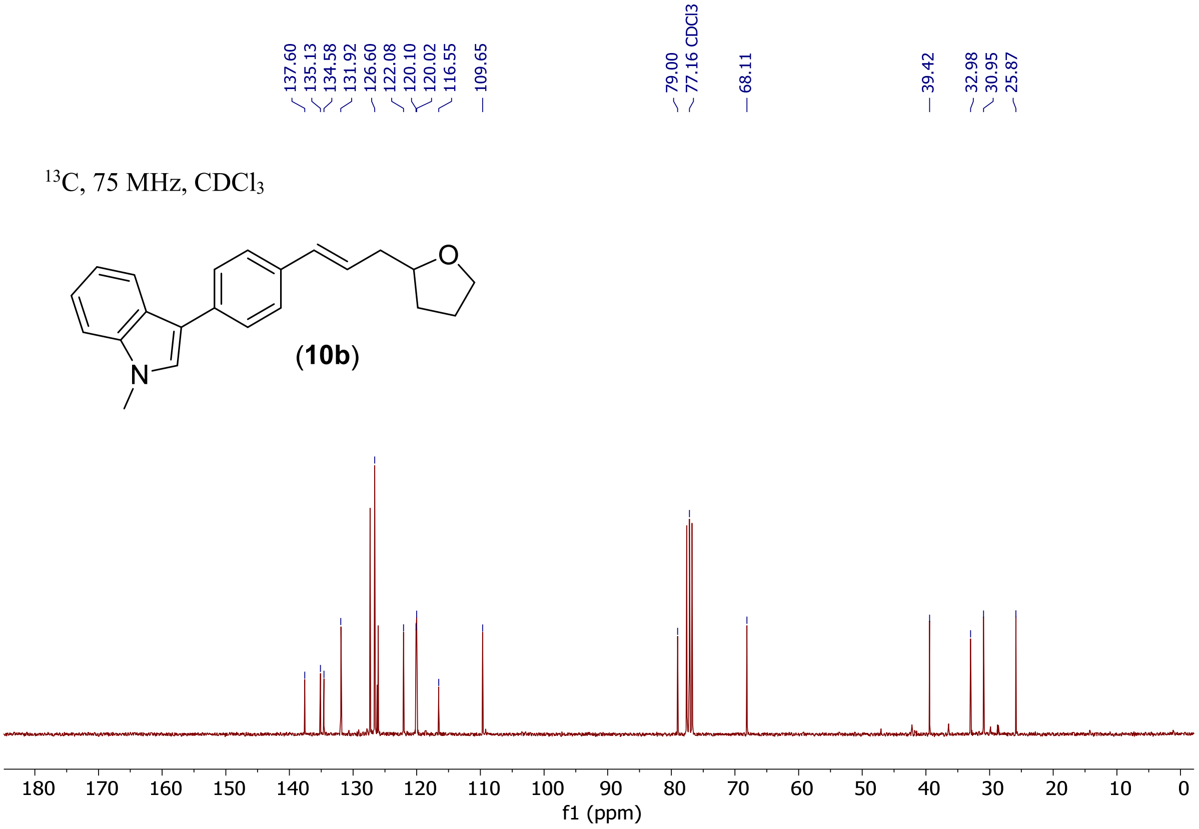
137.60 135.13 134.58 131.92 126.60 122.08 120.10 120.02 116.55 109.65 79.00 77.16 CDCl3 68.11 39.42 32.98 30.95 25.87
13C, 75 MHz, CDCl3
N
O
(10b)
180 170 160 150 140 130 120 110 100 90 80 70 60 50 40 30 20 10 0
f1 (ppm)

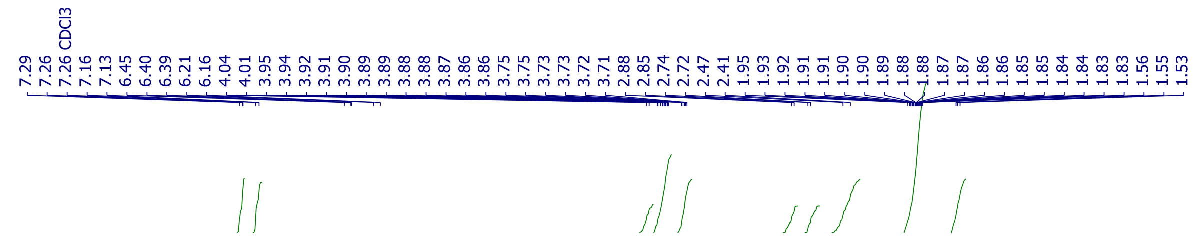

^{1}H, 300 MHz, $CDCl_3$

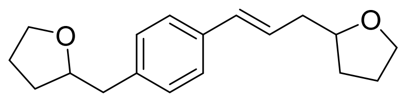

(**11a**)

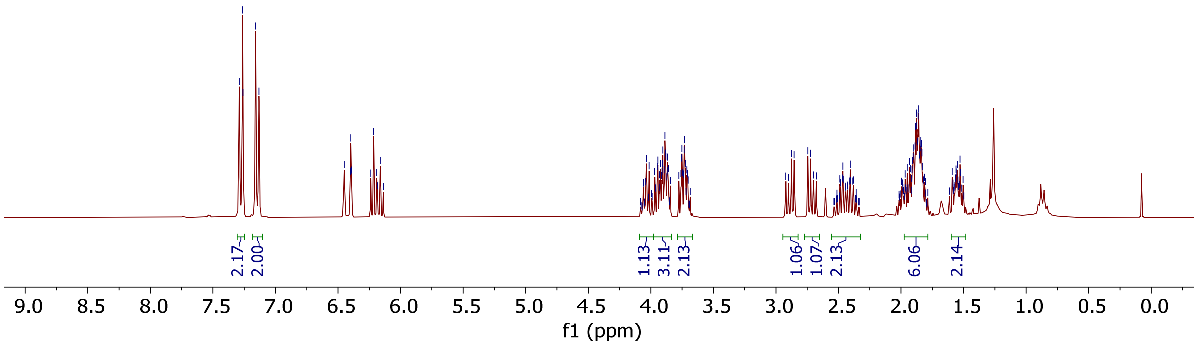

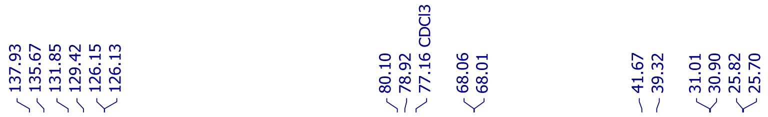

^{13}C, 75 MHz, $CDCl_3$

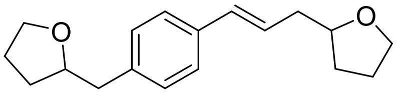

(**11a**)

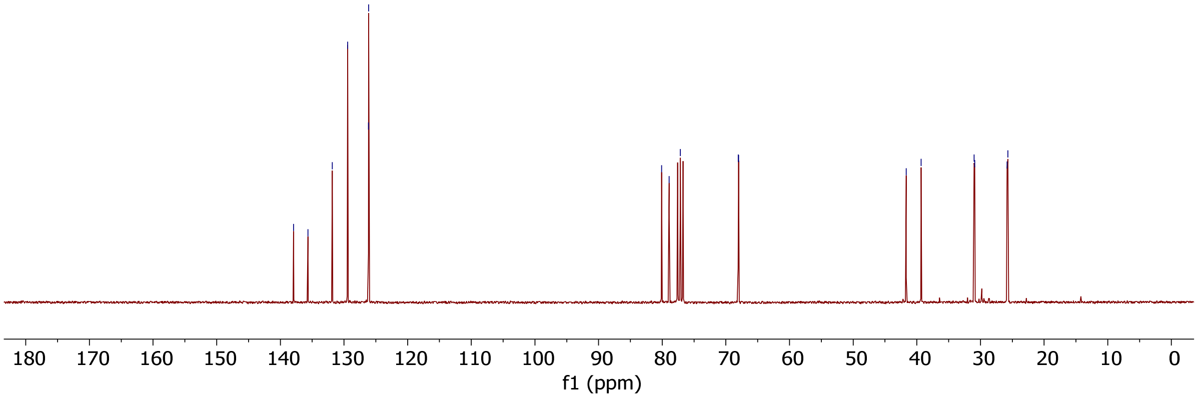

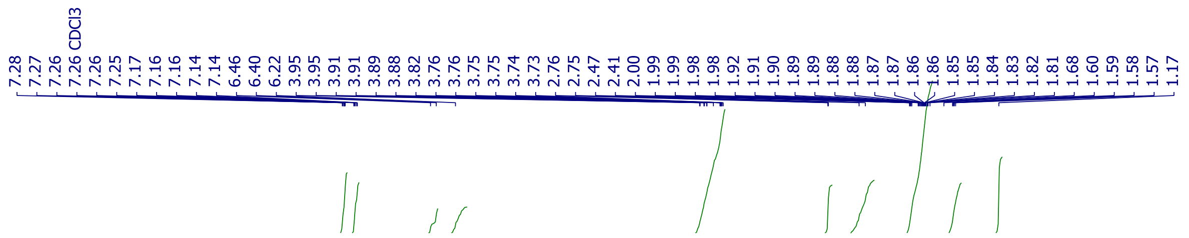

^{1}H, 300 MHz, $CDCl_3$

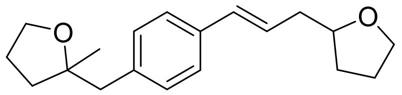

(**11b**)

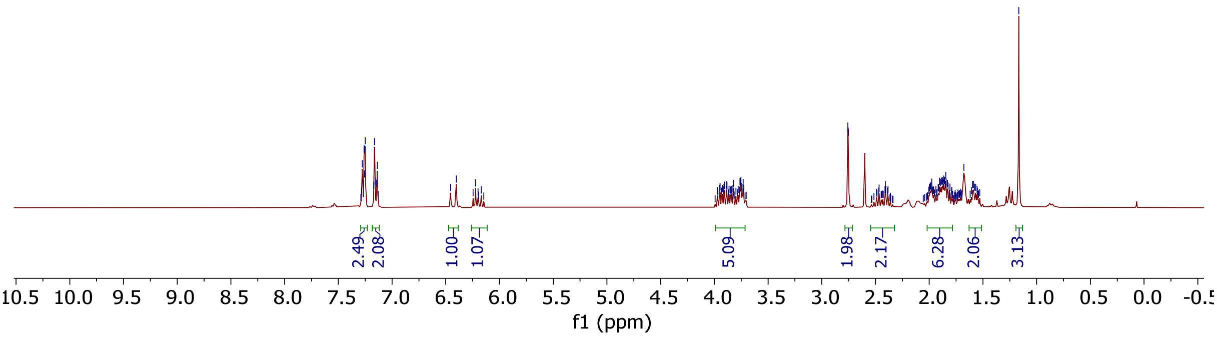

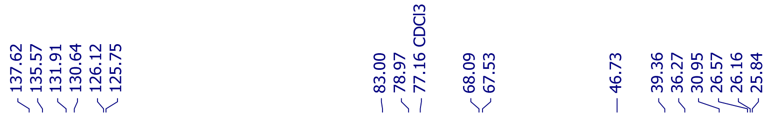

^{13}C, 75 MHz, $CDCl_3$

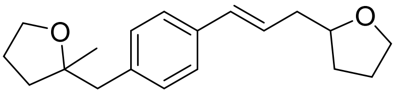

(**11b**)

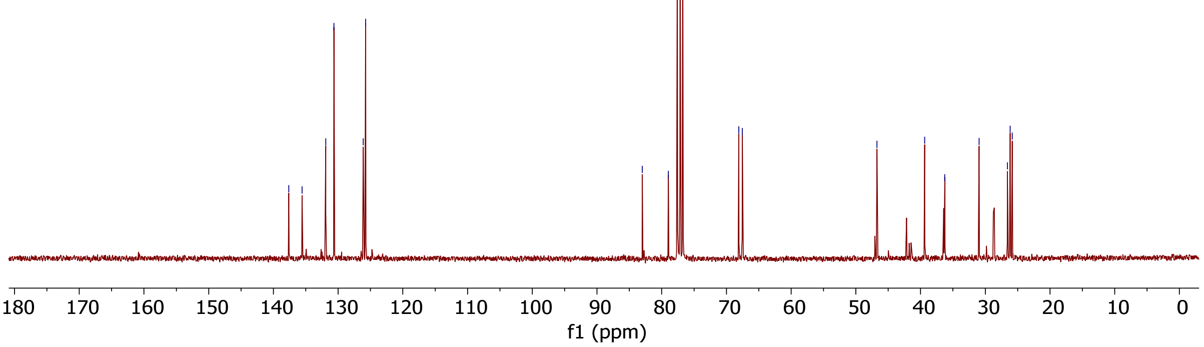

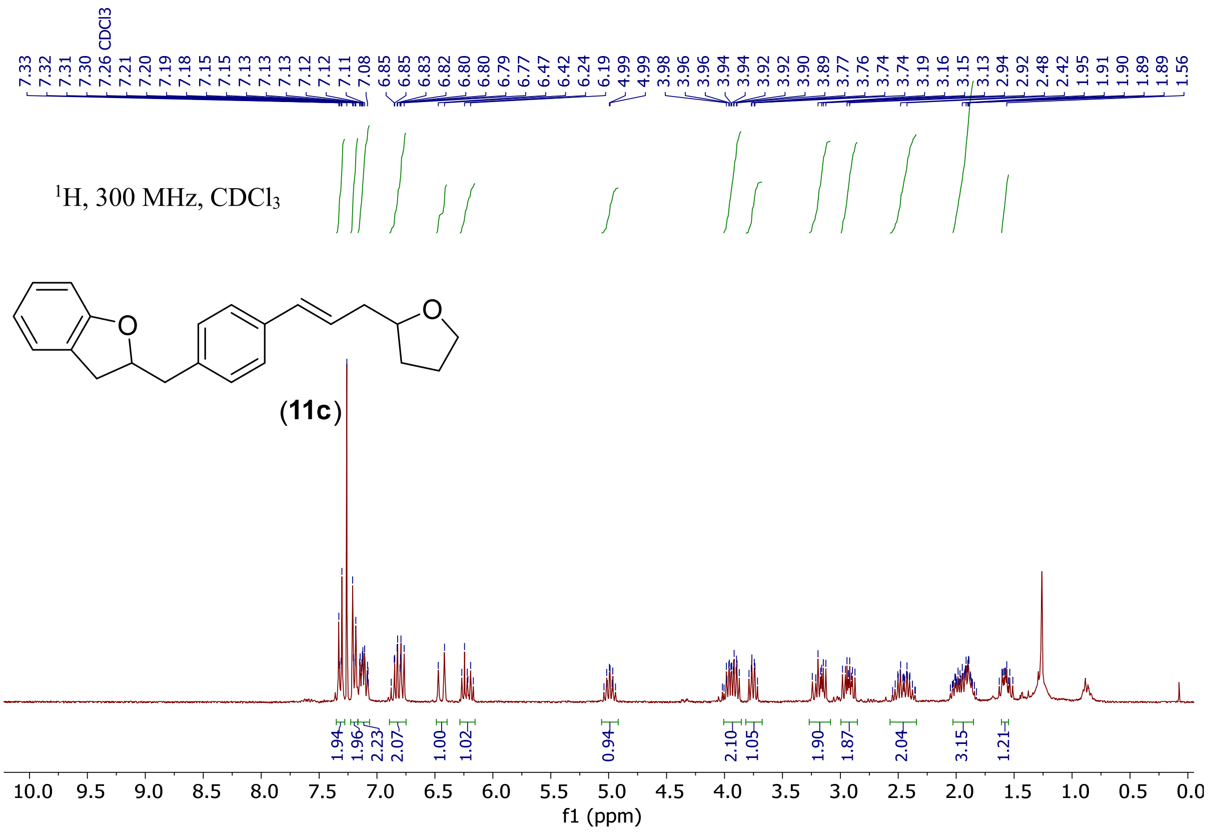
7.33 7.32 7.31 7.30 7.26 CDCl3 7.21 7.20 7.19 7.18 7.15 7.15 7.13 7.13 7.13 7.12 7.12 7.11 7.08 6.85 6.85 6.83 6.82 6.80 6.80 6.79 6.77 6.47 6.42 6.24 6.19 4.99 4.99 3.98 3.96 3.96 3.94 3.94 3.92 3.92 3.90 3.89 3.77 3.76 3.74 3.74 3.19 3.16 3.15 3.13 2.94 2.92 2.48 2.42 1.95 1.91 1.90 1.89 1.89 1.56
1H, 300 MHz, CDCl3
O
O
(11c)
1.94 1.96 2.23 2.07 1.00 1.02 0.94 2.10 1.05 1.90 1.87 2.04 3.15 1.21
10.0 9.5 9.0 8.5 8.0 7.5 7.0 6.5 6.0 5.5 5.0 4.5 4.0 3.5 3.0 2.5 2.0 1.5 1.0 0.5 0.0
f1 (ppm)

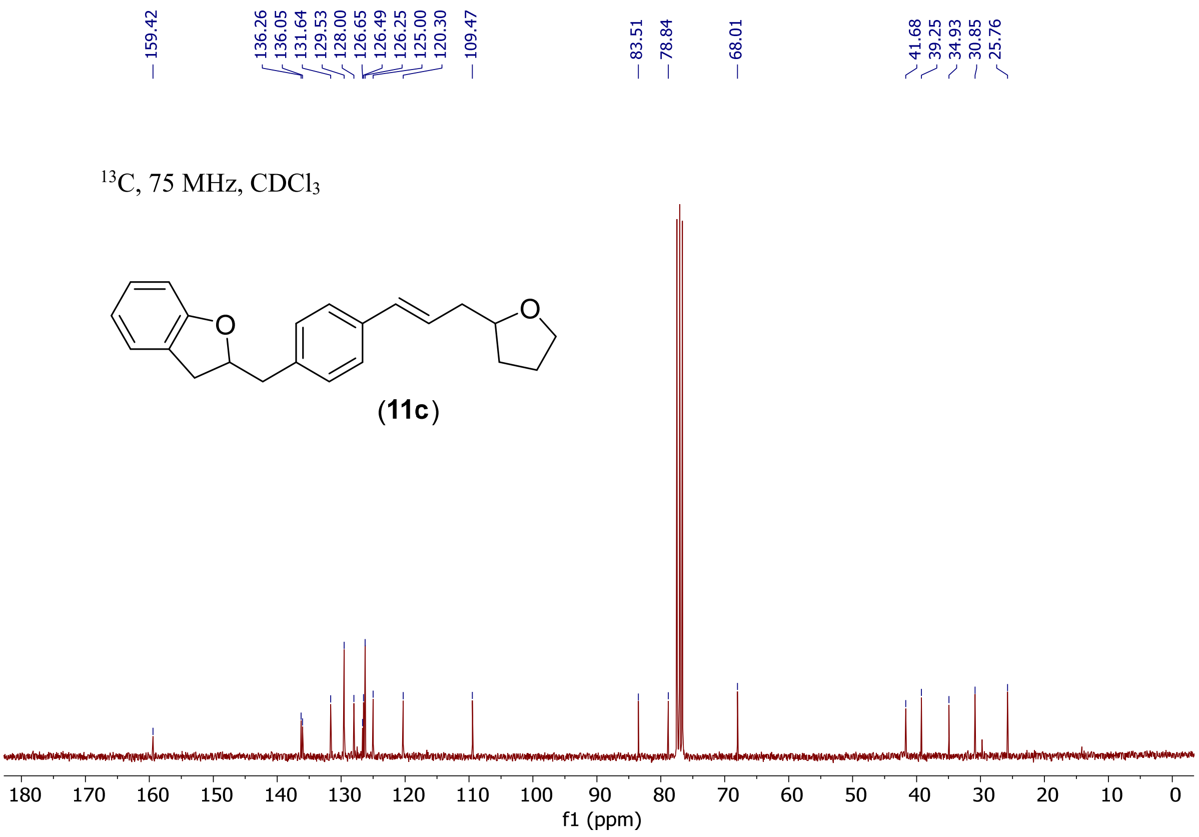
159.42
136.26 136.05 131.64 129.53 128.00 126.65 126.49 126.25 125.00 120.30 109.47
83.51 78.84 68.01
41.68 39.25 34.93 30.85 25.76
13C, 75 MHz, CDCl3
O
O
(11c)
180 170 160 150 140 130 120 110 100 90 80 70 60 50 40 30 20 10 0
f1 (ppm)

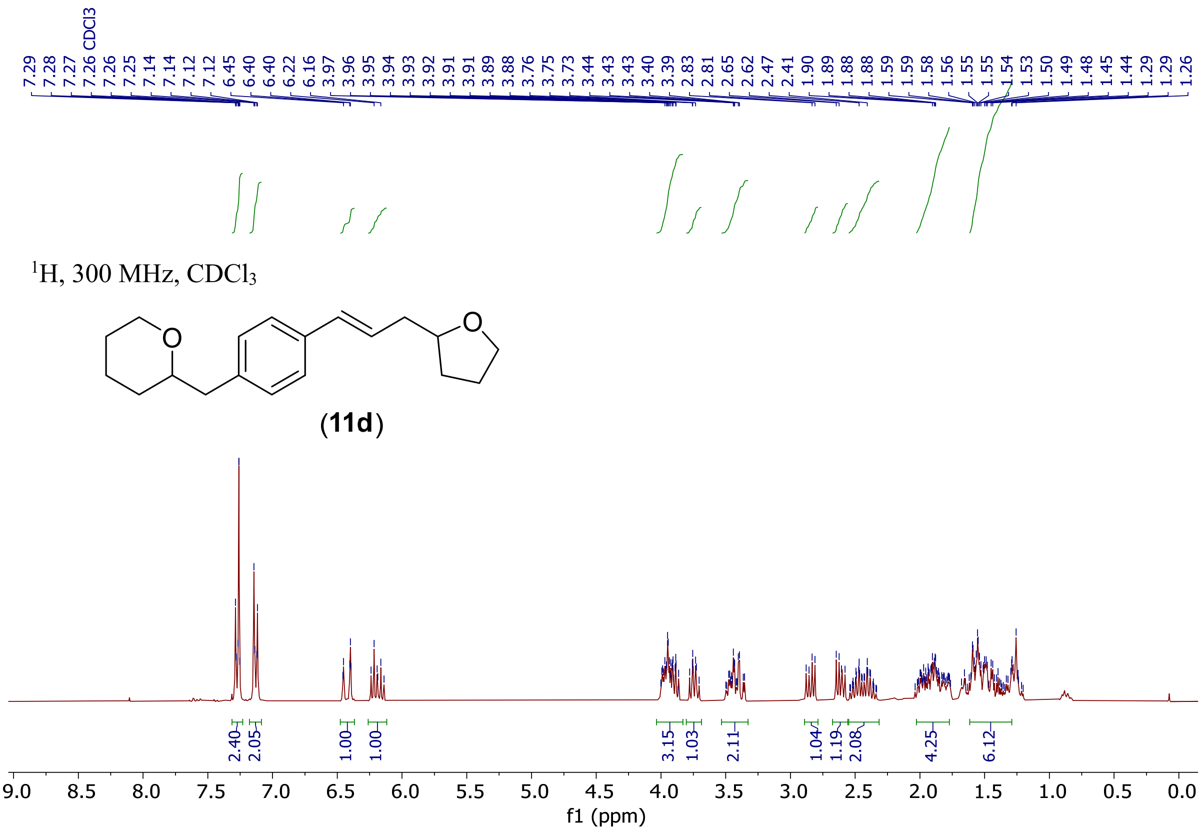
7.29 7.28 7.27 7.26 CDCl3 7.26 7.25 7.14 7.14 7.12 7.12 7.12 6.45 6.40 6.22 6.16 3.97 3.96 3.95 3.94 3.93 3.92 3.91 3.91 3.89 3.88 3.76 3.75 3.73 3.44 3.43 3.43 3.40 3.39 2.83 2.81 2.65 2.62 2.47 2.41 1.90 1.89 1.88 1.88 1.59 1.59 1.58 1.56 1.55 1.55 1.54 1.53 1.50 1.49 1.48 1.45 1.44 1.29 1.29 1.26
1H, 300 MHz, CDCl3
(11d)
2.40 2.05 1.00 1.00 3.15 1.03 2.11 1.04 1.19 2.08 4.25 6.12
f1 (ppm)

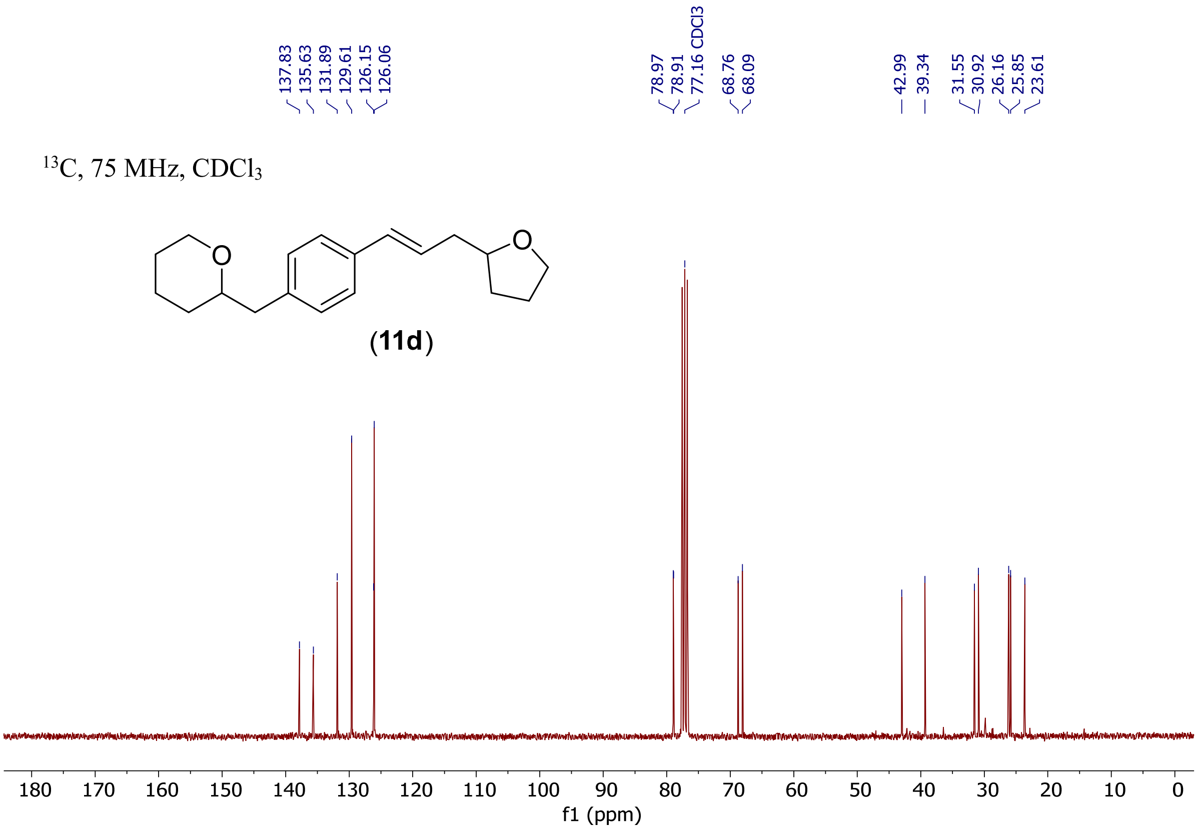
137.83 135.63 131.89 129.61 126.15 126.06 78.97 78.91 77.16 CDCl3 68.76 68.09 42.99 39.34 31.55 30.92 26.16 25.85 23.61
13C, 75 MHz, CDCl3
(11d)
f1 (ppm)

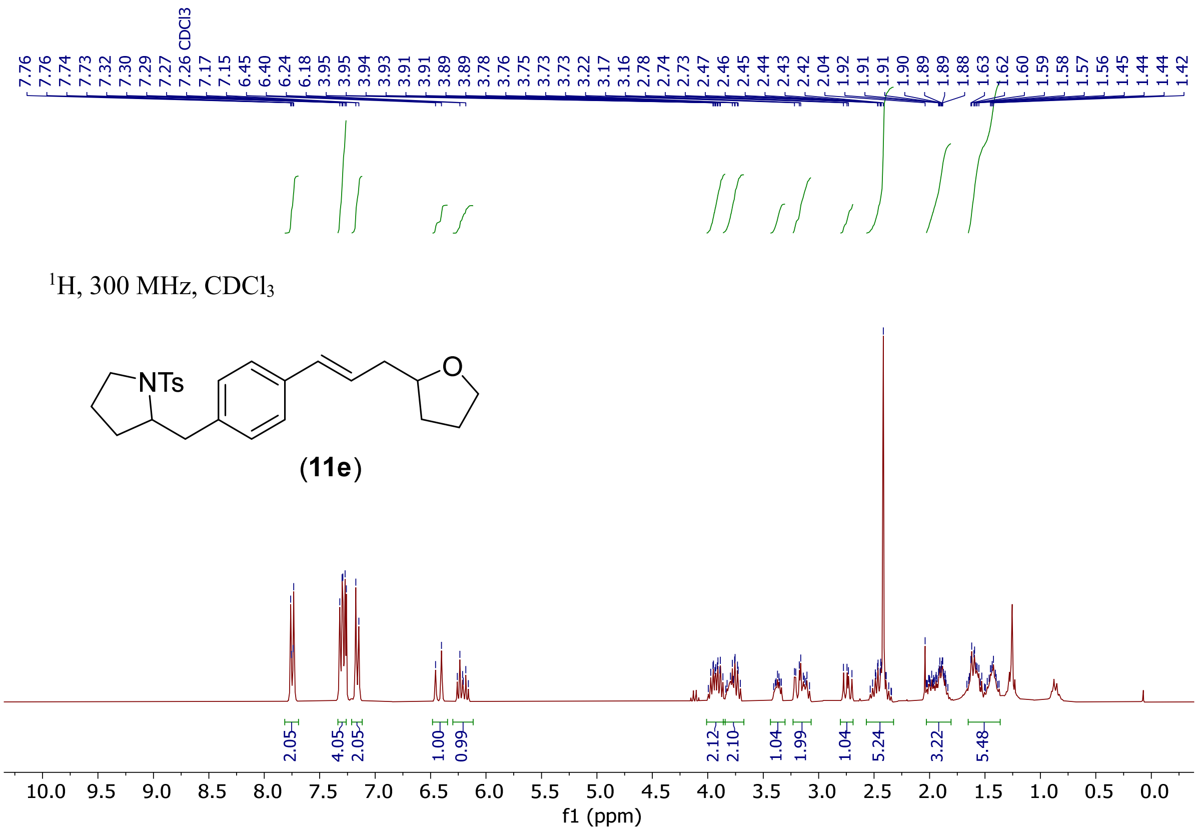
7.76 7.76 7.74 7.73 7.32 7.30 7.29 7.27 7.26 CDCl3 7.17 7.15 6.45 6.40 6.24 6.18 3.95 3.95 3.94 3.93 3.91 3.91 3.89 3.89 3.78 3.76 3.75 3.73 3.73 3.22 3.17 3.16 2.78 2.74 2.73 2.47 2.46 2.45 2.44 2.43 2.42 2.04 1.92 1.91 1.91 1.90 1.89 1.89 1.88 1.63 1.62 1.60 1.59 1.58 1.57 1.56 1.45 1.44 1.44 1.42
¹H, 300 MHz, CDCl₃
NTs
O
(11e)
2.05 4.05 2.05 1.00 0.99 2.12 2.10 1.04 1.99 1.04 5.24 3.22 5.48
10.0 9.5 9.0 8.5 8.0 7.5 7.0 6.5 6.0 5.5 5.0 4.5 4.0 3.5 3.0 2.5 2.0 1.5 1.0 0.5 0.0
f1 (ppm)

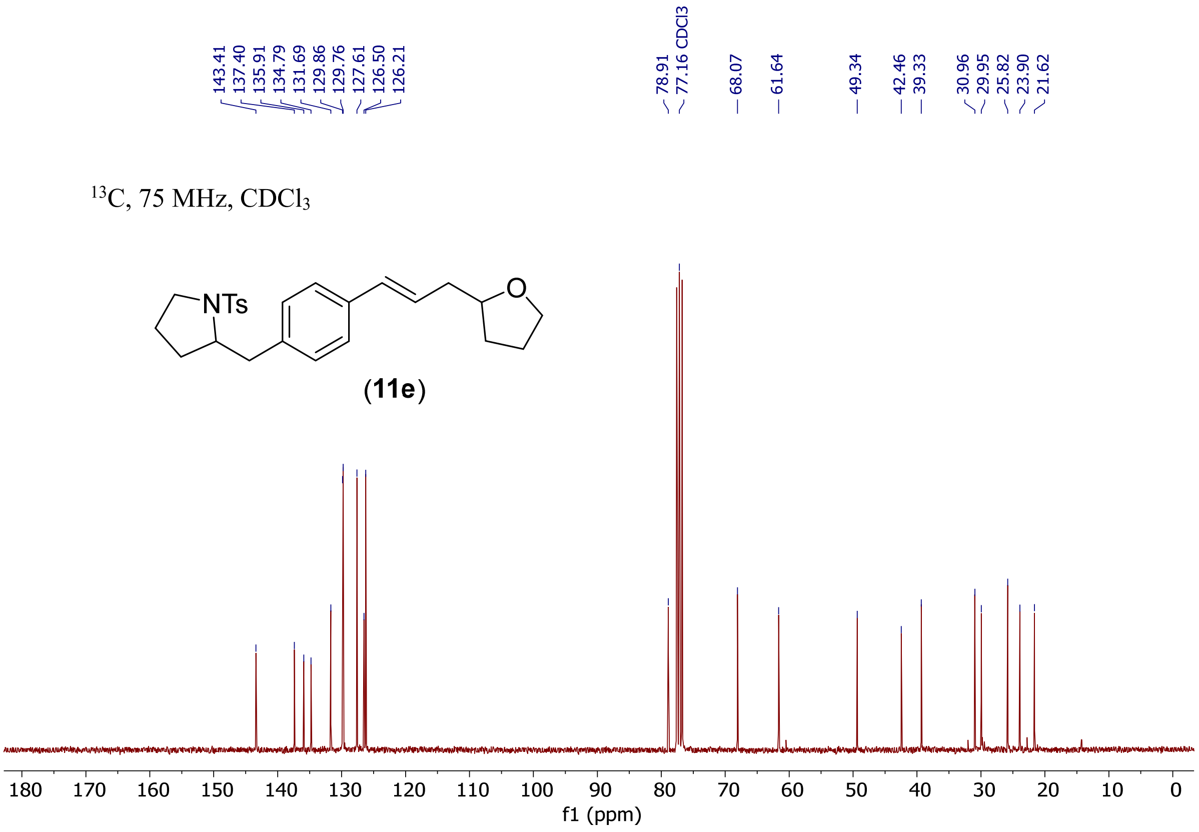
143.41 137.40 135.91 134.79 131.69 129.86 129.76 127.61 126.50 126.21
78.91 77.16 CDCl3 68.07 61.64 49.34 42.46 39.33 30.96 29.95 25.82 23.90 21.62
¹³C, 75 MHz, CDCl₃
NTs
O
(11e)
180 170 160 150 140 130 120 110 100 90 80 70 60 50 40 30 20 10 0
f1 (ppm)

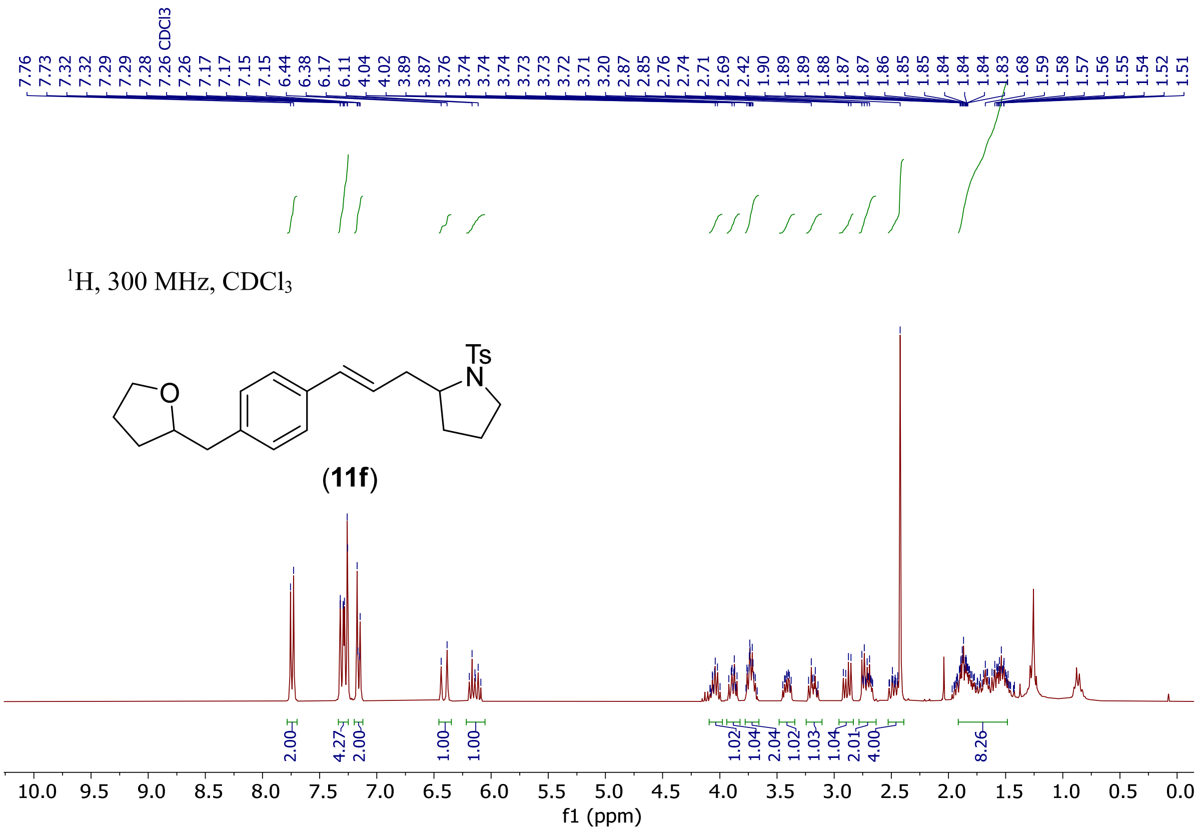

^{1}H, 300 MHz, $CDCl_3$

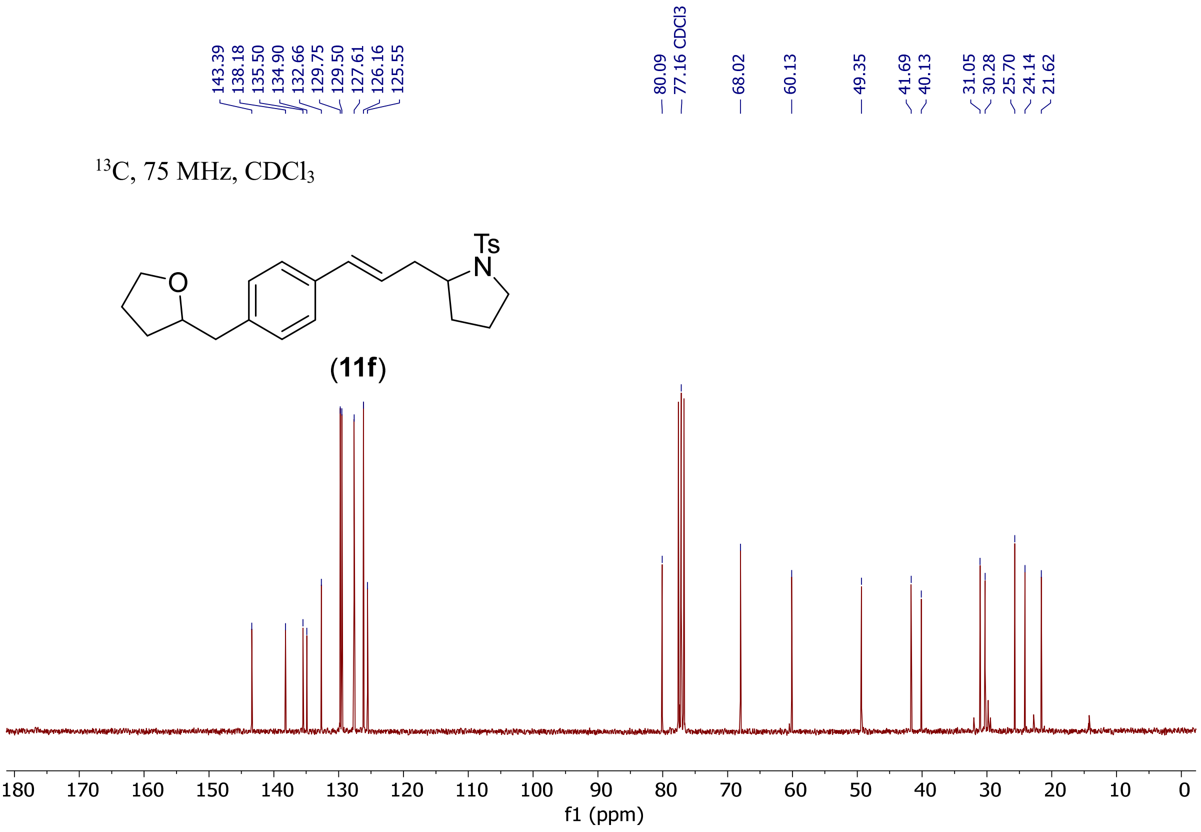

^{13}C, 75 MHz, $CDCl_3$
